# A machine-learning-based alloy design platform that enables both forward and inverse predictions for thermo-mechanically controlled processed (TMCP) steel alloys

**DOI:** 10.1038/s41598-021-90237-z

**Published:** 2021-05-26

**Authors:** Jin-Woong Lee, Chaewon Park, Byung Do Lee, Joonseo Park, Nam Hoon Goo, Kee-Sun Sohn

**Affiliations:** 1 Nanotechnology & Advanced Materials Engineering, Sejong University, 209 Neungdong-ro, Gwangjin-gu, Seoul, 143-747 South Korea; 2 Advanced Research Team, Hyundai Steel DangJin Works, DangJin, Chungnam 31719 South Korea

**Keywords:** Materials science, Structural materials, Theory and computation

## Abstract

Predicting mechanical properties such as yield strength (YS) and ultimate tensile strength (UTS) is an intricate undertaking in practice, notwithstanding a plethora of well-established theoretical and empirical models. A data-driven approach should be a fundamental exercise when making YS/UTS predictions. For this study, we collected 16 descriptors (attributes) that implicate the compositional and processing information and the corresponding YS/UTS values for 5473 thermo-mechanically controlled processed (TMCP) steel alloys. We set up an integrated machine-learning (ML) platform consisting of 16 ML algorithms to predict the YS/UTS based on the descriptors. The integrated ML platform involved regularization-based linear regression algorithms, ensemble ML algorithms, and some non-linear ML algorithms. Despite the dirty nature of most real-world industry data, we obtained acceptable holdout dataset test results such as R^2^ > 0.6 and MSE < 0.01 for seven non-linear ML algorithms. The seven fully trained non-linear ML models were used for the ensuing ‘inverse design (prediction)’ based on an elitist-reinforced, non-dominated sorting genetic algorithm (NSGA-II). The NSGA-II enabled us to predict solutions that exhibit desirable YS/UTS values for each ML algorithm. In addition, the NSGA-II-driven solutions in the 16-dimensional input feature space were visualized using holographic research strategy (HRS) in order to systematically compare and analyze the inverse-predicted solutions for each ML algorithm.

## Introduction

To facilitate discovery and understanding, machine learning (ML) approaches have become mainstays in the field of steel alloy design research^1–18^. Sha et al.^1^ were among the first to use an artificial neural network (ANN) model to connect the composition, processing parameters, working conditions, and mechanical properties for maraging steels. Now, ML-related work is both more advanced and almost ubiquitous. There are many examples of this phenomenon: Xiong et al.^2^ used a random forest (RF) algorithm chosen out of the WEKA library^3^ to develop a ML model that describes the glass-forming ability and elastic moduli of bulk metallic glasses; Möller et al.^4^ used a support vector machine (SVM) to develop hard magnetic materials; Shen et al.^5^ successfully established a physical metallurgy-guided ML model (SVM) and a genetic algorithm (GA)-based design process to produce ultrahigh-strength stainless steels; Zhang et al.^6^ incorporated GAs for the model and descriptor selection for high entropy alloys (HEAs) and incorporated several ML algorithms involving ANN, RF, SVM, etc.; Kaufmann et al.^7^ also used RF to manipulate HEAs; Wang et al.^8^ employed a group of ML algorithms to develop Fe-based soft magnetic materials; Khatavkar et al.^9^ employed Gaussian process regression (GPR) and SVR to advance Co- and Ni-based superalloys; Wen et al.^10^ employed ML models that include simple linear regression, SVM with various kernels, ANN, and K-nearest neighbors (KNN) to fabricate HEAs with a high degree of hardness; Feng et al.^11^ utilized a deep neural network (DNN) to predict the defects in stainless steel; Sun et al.^12^ used SVM models to predict the glass-forming ability of binary metallic alloys; Ward et al.^13^ constructed an RF model to design metallic glasses and validated them via commercially viable fabrication methods; Ren et al.^14^ employed a so-called general-purpose ML framework^15^ and high-throughput experiments to predict glass-forming likelihood; and, several other metallic glass alloys have also been studied by employing ML approaches^16,17^.

Herein, we report an integrated ML platform, which is a much-improved version of our previous ML strategy^19^. Most ML-based studies of metallic alloy design adopt, at best, a few ML algorithms. Although Xiong et al.^2^ has argued that the 20 ML algorithms available in the WEKA library^3^ were tested before the RF was finally selected, no substantial back-up data for the other ML algorithms were presented. In addition, Zhang et al.^6^, Wang et al.^8^, and Wen et al.^10^ employed several ML algorithms for a single problem. We incorporated 16 ML algorithms and provided full details for each algorithm involving a systematic hyper-parameter optimization and NSGA-II-driven inverse design (prediction) as well as visualization in the input feature space. Note that ANN, which was a major ML algorithm in our previous report^19^, is not mentioned in our account of the present investigation.

The integrated ML platform suggested here consisted of three ML algorithm groups. The first group included regularized linear regression algorithms such as Ridge^20^, Lasso^21^, Elastic net^22^, Kernel Ridge regression (KRR)^23^, Least-angle regression (LARS) Lasso^24^, Bayesian ridge regression (BRR)^25^, and automatic relevance determination (ARD)^26^. Of course, a basic linear regression algorithm was also included in this group. We also adopted ensemble algorithms such as random forest (RF)^27^, Ada Boost^28^, gradient Boost^29^, and XG Boost^30^. The third group included non-linear regression ML algorithms such as k-nearest neighbor (KNN)^31^, support vector machine (SVM)^32^, Gaussian process regression (GPR)^33^, and partial least square (PLS)^34^. PLS is not a non-linear regression ML algorithm, but it was categorized into this group just for the sake of convenience.

Xue et al.^35,36^ developed ML models along with a heuristic referred to as efficient global optimization (EGO) to search for shape-memory alloys. In addition, a GA-driven inverse design (or inverse prediction) was achieved based on SVM, RF, and XG Boost algorithms to design multicomponent β-Ti alloys with a low Young’s modulus^37^. Shen et al.^5^ have also used GAs successfully for inverse design. We used a deep neural networks (DNNs)-based inverse design^19^ in association with an elitist-reinforced non-dominated sorting genetic algorithm (NSGA-II)^38^. Our NSGA-II-related inverse design approach has a lucid distinction from others based on the fact that we deal with more than one objective function, and two output features are simultaneously optimized with our approach. In this regard, we employed the NSGA-II algorithm to systematically sort through inverse design problems in order to predict solutions (optimal sets of 16 input features) from non-dominated output features (YS and UTS). This approach takes advantage of the multi-objective optimization to deal with the multi-output-feature ML models. The NSGA-II algorithm has proven to be versatile and we have been reporting various successful materials discoveries based on use of the NSGA-II and -III^39–41^.

Of the 16 ML algorithms, seven showed promise and deserved to be used for inverse design (or prediction). As clearly described above, an NSGA-II was used to optimize the seven fully trained ML models. Using the NSGA-II algorithm for inverse prediction problems for the seven multi-output-feature ML models is an unprecedented approach, although conventional single-objective genetic algorithms in association with a particular ML algorithm have more often been used for the same purpose^5,19,35–37,42–44^. Since two objective functions (= two output features, YS and UTS) were of concern in the present investigation, both had to be simultaneously optimized, and the NSGA-II was the best option for that optimization process.

A dataset involving 5473 TMCP steel alloys provided by Hyundai Steel Co. was used for the integrated ML platform. This industry-produced, real-world data was compromised by the effect of the so-called ‘dirty nature’ of data due to human intervention during the data production, which leads to data that is non-identically and independently distributed (Non-IID). In our previous report, the nature of the data was described well and included conventional data analysis based on both a pair-wise scatter plot and a Pearson correlation coefficient matrix^19^. For this study, we adopted an alternative, more comprehensive data visualization strategy, a so-called holographic research strategy (HRS)^45–48^, that is capable of a simpler visualization of multi-dimensional data in contrast to the well-known data dimension reduction and visualization methodologies such as the principal component analysis (PCA) that accompanies considerable amounts of information loss.

## Results and discussion

### Regression results for 16 ML algorithms

The 6- and 5-fold cross validation had a similar goodness of fit, viz., the mean square error (MSE) and coefficient of determination (R^2^), which led to the conclusion that the regression fitting quality was acceptable irrespective of the data-splitting option. The validation MSE and R^2^ values were slightly worse than those for the training of both data-splitting schemes, and the holdout dataset test results were slightly worse than the validation, so that the holdout dataset test results for the 5-fold cross-validation were accepted as a baseline in the present investigation. The reason for the slightly lower fitting quality of the holdout dataset test by comparison with those for validation was ascribed to our policy that the worst hold-out dataset should be adopted as the final decision out of many different hold-out datasets. The validation MSE and R^2^ were averaged among 6 or 5 validation data subsets, some of which had a better fit than those for the training dataset, while some others exhibited MSE and R^2^ values far below the average. The selected test MSE and R^2^ values were equivalent to the under-averaged validation MSE and R^2^ values.

**Figure 1:**
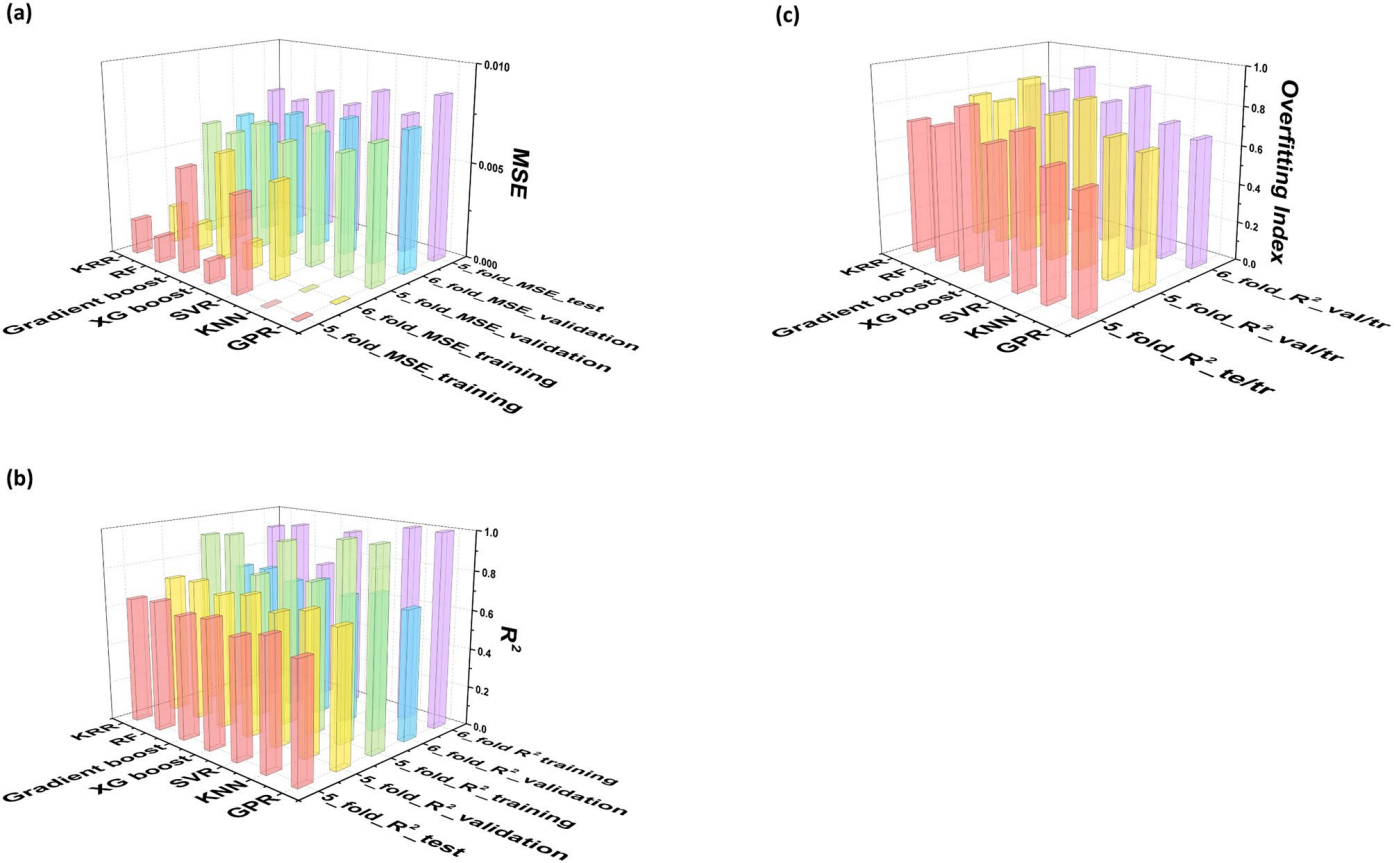
The training, validation and hold-out dataset test results for the seven non-linear ML algorithms in terms of (**a**) MSE and (**b**) R^2^ for 6- and 5-fold cross validation with a holdout dataset test, (**c**) the over-fitting index defined as Validation_R^2^/Training_R^2^ and Test_R^2^/Training_R^2^.

Table 1 shows the MSE and R^2^ results from the training, validation, and holdout dataset testing for the 6- and 5-fold cross-validations. The goodness of fit remained almost identical for both the 6- and 5-fold cross-validations. Despite the superior fitting quality for training and validation (i.e., lower MSE and higher R^2^ for training), we placed greater emphasis on the holdout test MSE and R^2^ results since these were considered the baseline. While the overall MSE level was approximately 10^–3^–10^–2^ for training, the validation MSE was increased and the holdout dataset test MSE results were slightly further increased. The validation and test MSE values greater than 10^–2^ would not be acceptable but the MSE for several algorithms was in an acceptable range that was far below 10^–2^. The overall R^2^ levels for the training were approximately 0.5–1 (mostly around 0.9), and the R^2^ levels for both the validation and the holdout dataset test were approximately 0.5–0.7. Also, several algorithms showed validation and test R^2^ values of approximately 0.7. If R^2^ for the validation (or the holdout dataset test) exceeds 0.7 for physical science (lower in social science), then the statistics research society considers the regression results to be robust^49^, although a conventional standard for the R^2^ level has yet to be clearly defined. It should be noted that in the present study the training MSE and R^2^ for KNN and GPR reached the perfect level, i.e., 0 and 1, respectively. This was due to the inherent non-parametric (or model-free) trait of the KNN and GPR algorithms. Only the seven non-linear ML algorithms (KRR, RF, Gradient boost, XG boost, SVR, KNN and GPR) led to acceptable validation and test results by exhibiting almost similar MSE and R^2^ levels, while the regression fitting quality for the others, in particular the basic linear regression algorithm and its regularized versions, was far behind those of the aforementioned seven algorithms. It is evident that all linear regression gave rise to an atrocious fitting quality. This means that the data status seemed highly non-linear since only the non-linear regression algorithms were successful. Figure 1 graphically summarizes the MSE and R^2^ values for only the seven algorithms.

**Table 1:** The training, validation and hold-out dataset test results in terms of MSE and R^2^ for two data-splitting schemes: 6-fold cross-validation (with no holdout dataset test) and 5-fold cross-validation with a holdout test dataset.

	6-Fold cross validation				5-Fold cross validation with hold-out dataset test					
	MSE (training)	R^2^ (training)	MSE (validation)	R^2^ (validation)	MSE (training)	R^2^ (training)	MSE (validation)	R^2^ (validation)	MSE (test)	R^2^ (test)
Basic linear	0.01054	0.50619	0.01062	0.50194	0.01042	0.51559	0.01053	0.51013	0.01120	0.45445
Ridge	0.01054	0.50616	0.01062	0.50196	0.01042	0.51553	0.01053	0.51016	0.01118	0.45520
Lasso	0.01054	0.50618	0.01062	0.50194	0.01042	0.51557	0.01053	0.51013	0.01119	0.45482
LARS	0.01117	0.47690	0.01120	0.47468	0.01101	0.48821	0.01106	0.48538	0.01133	0.44781
ENR	0.01066	0.50039	0.01073	0.49697	0.01056	0.50903	0.01064	0.50481	0.01114	0.45728
KRR	0.00196	0.90795	0.00617	0.71054	0.00179	0.91684	0.00610	0.71586	0.00728	0.64537
BRR	0.01054	0.50607	0.01062	0.50190	0.01042	0.51542	0.01053	0.51009	0.01117	0.45567
ARD	0.01054	0.50609	0.01062	0.50183	0.01042	0.51545	0.01053	0.50999	0.01118	0.45536
RF	0.00142	0.93353	0.00591	0.72277	0.00133	0.93827	0.00587	0.72657	0.00692	0.66267
Ada Boost	0.00960	0.55009	0.00987	0.53697	0.00950	0.55828	0.00983	0.54263	0.01017	0.50440
Gradient Boost	0.00561	0.73716	0.00682	0.68001	0.00532	0.75283	0.00670	0.68811	0.00768	0.62585
XG Boost	0.00136	0.93624	0.00608	0.71474	0.00114	0.94720	0.00604	0.71883	0.00723	0.64757
SVR	0.00493	0.76910	0.00716	0.66437	0.00485	0.77447	0.00717	0.66626	0.00821	0.60003
KNN	0.00000	1.00000	0.00624	0.70733	0.00000	1.00000	0.00622	0.71028	0.00727	0.64595
PLS	0.01171	0.45137	0.01174	0.44938	0.01165	0.45821	0.01170	0.45573	0.01198	0.41626
GPR	0.00001	0.99976	0.00724	0.66042	5.06E-06	0.99976	0.00702	0.67268	0.00805	0.60791

**Figure 2:**
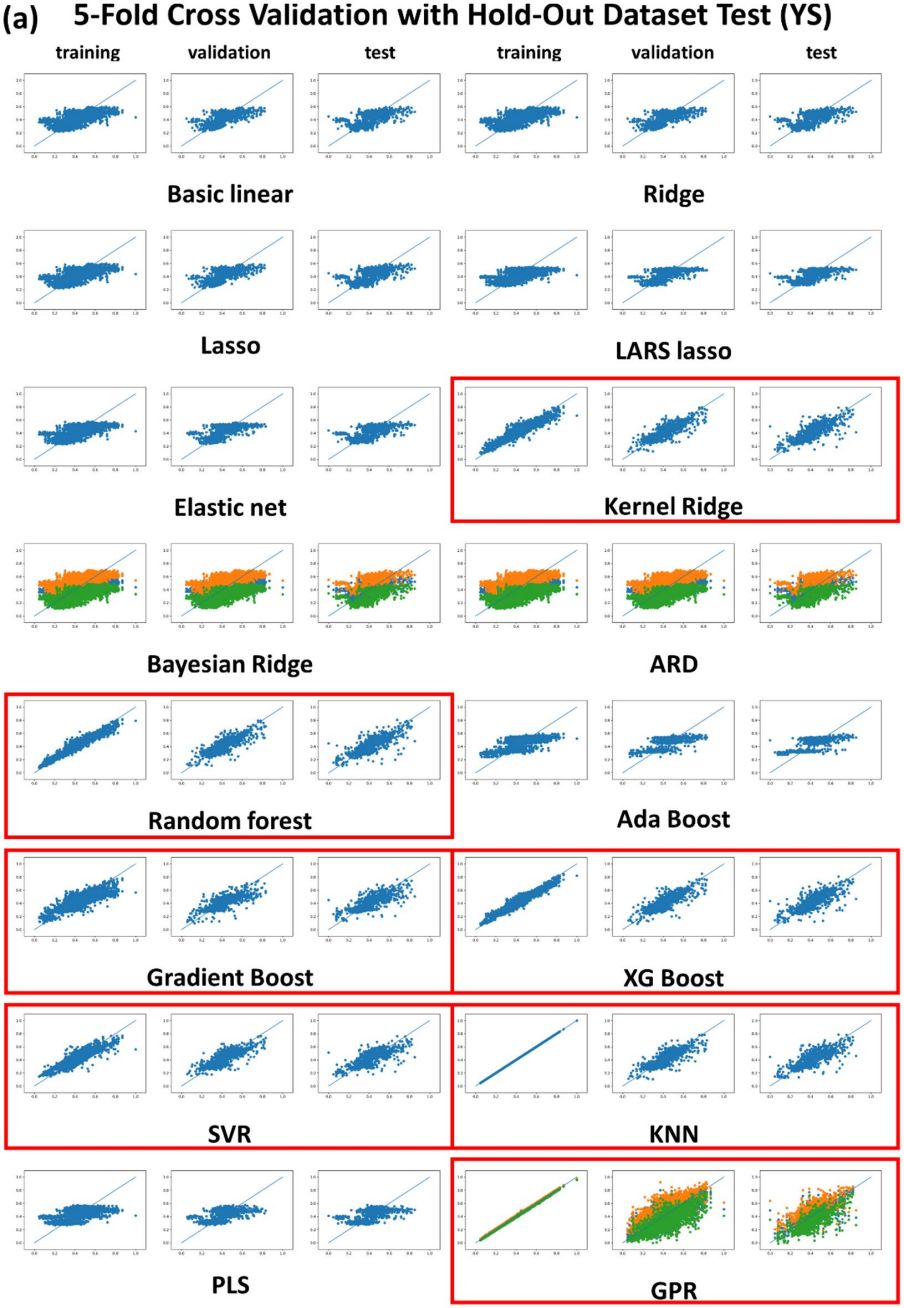

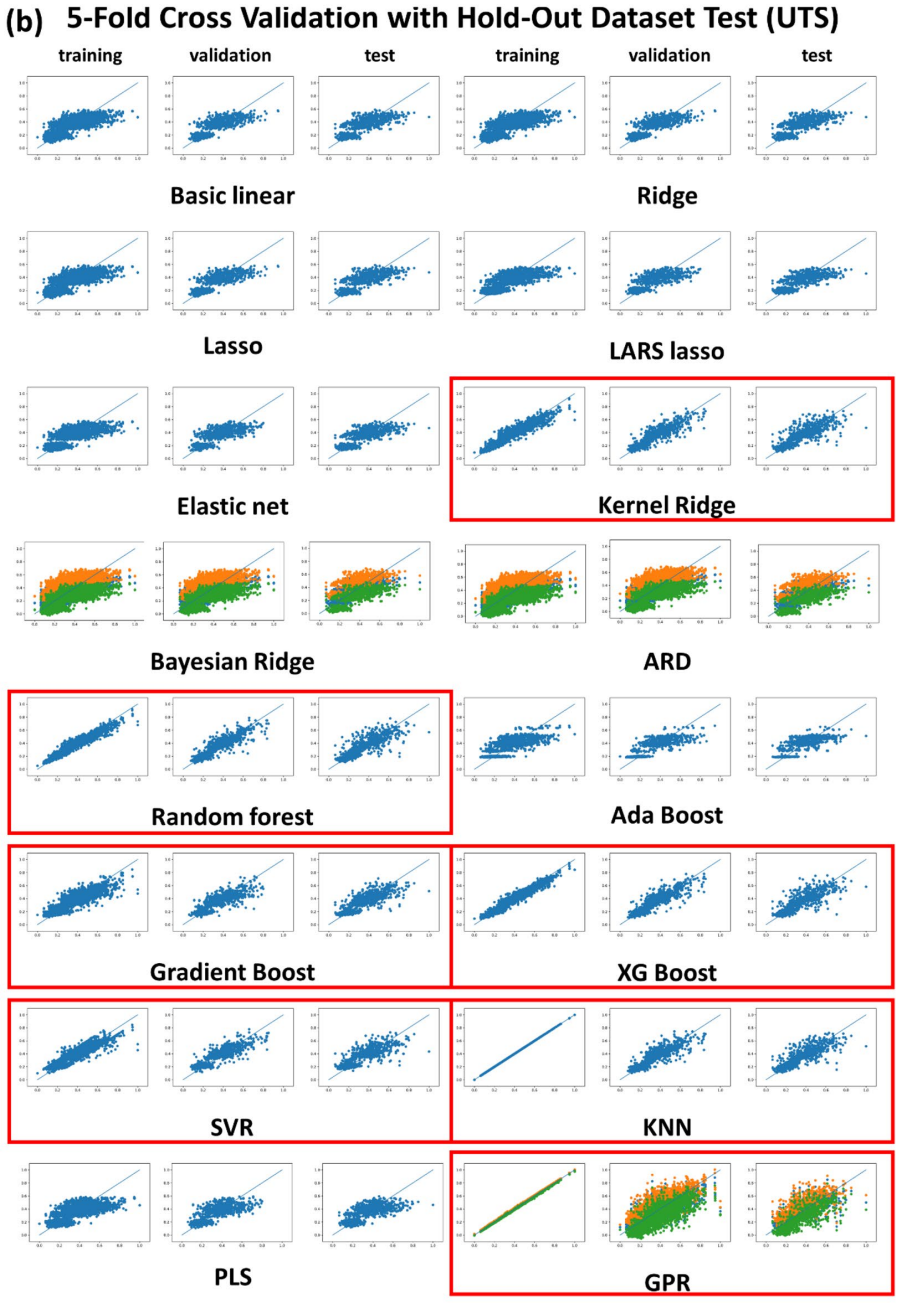
Plots of predicted vs. experimental (**a**) YS and (**b**) UTS for training, validation and hold-out test datasets for 5-fold cross validation.

Figure 2 shows the plots of the predicted vs. experimental YS and UTS, wherein the training, validation and test datasets for the fivefold cross validation led to a relatively good fit only for the seven nonlinear regression algorithms KRR, RF, Gradient boost, XG boost, SVR, KNN and GPR (marked as red boxes), but the basic and regularization-involved linear regression and PLS algorithms were far worse by comparison with the seven nonlinear regression algorithms. The same trend was detected in the sixfold cross validation results, which is available in the “Supplementary Information” (Fig. S1). ANN (or DNN) results were omitted from Table 1 and from Figs. 1 and 2, because the regression results were well described in the previous report^19^. The regression fitting quality for the DNN in the previous report was as good as those for the seven nonlinear regression algorithms. Consequently, the linear regression algorithms gave rise to the poorest regression results. The regularization-involved linear regression algorithms such as Ridge, LASSO, Elastic net, BRR, and ARD were never successful, and the absolute MSE and R^2^ levels were unacceptable and equivalent to the basic linear regression, as shown in Table 1 and in Fig. 2. Of the regularization-involved linear regression algorithms, only the KRR was acceptable. Although the main function of KRR is based on regularized linear regression, it could be regarded as a typical non-linear regression algorithm due to the use of kernels (we adopted a matern kernel through the hyper-parameter optimization procedures). The regularized linear regression algorithms work best in the case of linear problems with a small dataset (data paucity cases, normally). By contrast, our TMCP steel dataset had a non-linear nature with a dataset size that could be considered moderate, which is compatible with the size of the problem. This led to us to conclude that the TMCP steel dataset size was sufficiently large and no regularization was necessary. In addition, the PLS conferred an unacceptable regression quality, which could be rationalized by invoking the fact that the PLS algorithm is another type of linear regression, which would naturally give a poor regression result. The PLS algorithm is known to work best for a linear problem particularly when the number of data points is fewer than the number of descriptors^34^. That was not the case, however, for the TMCP steel dataset.

The ensemble algorithms returned promising regression results, thanks to their non-linear regression capability. It is interesting, however, that Ada boost had a poor regression result. The poor fitting quality for Ada boost was due to a choice of basic estimators that differed from that of the other ensemble algorithms. As already discussed above, decision trees with some depth levels were adopted as a basic estimator for the other three ensemble algorithms such as RF, Gradient boost, and XG boost. On the other hand, Ada boost employed a so-called stump that consists of a one-level decision tree, i.e., uses only a single attribute for splitting. In this regard, the model complexity for Ada boost has been significantly reduced so that it resembles a simple linear regression. Consequently, the higher complexities of the RF, Gradient boost, and XG boost algorithms were appropriate for the present non-linear problem.

As shown in Fig. 2, the BRR, ARD, and GPR regression based on the Bayesian approach produced conspicuous and distinctive regression results. Bayesian approach-involved algorithms give a range of confidence (error range) around the predicted mean rather than a deterministic prediction. The amber and green dots for the BRR, ARD, and GPR results designate the upper and lower confidence boundaries, as shown in Fig. 5. These Bayesian approaches are more favorable than the customary regression strategies due to the fact that uncertainty in the prediction can also be formulated. It is noted, however, that this sort of merit for the Bayesian approach did not take effect for the BRR and ARD algorithms because they were poorly fitted. It should be noted that BRR and ARD were still based on a linear regression. Only GPR algorithms capable of accommodating the non-linearity problem exhibited acceptable regression results, along with reasonable upper and lower confidence boundaries.

In addition to the fitting quality (bias issue) parameterized via MSE and R^2^, the over-fitting (variance) issue should be carefully taken into account when judging the regression fit. The ratio between the training and validation of R^2^ could be indicative of the level of over-fitting, which is referred to as the ‘over-fitting index’. Figure 1c shows the over-fitting index in a range of from 0.65 to 0.92, which is defined as Validation_R^2^/Training_R^2^. A higher over-fitting index indicates a better fit, i.e., 1 is reached in an ideal case. Figure 1c also shows the over-fitting index, defined as the Test_R^2^/Training_R^2^, which is similar to the Validation_R^2^/Training_R^2^. However, the over-fitting index would not matter for linear regression-based algorithms with a poor fitting quality, because the overfitting issue matters only under the premise that the regression results for a training dataset are acceptable—the overfitting index make sense only when the regression is not poorly fitted. Thus, the over-fitting index stood out only for the seven non-linear regression algorithms. Although all the linear regressions gave rise to quite an acceptable over-fitting index, it is futile to mention it since they were extremely biased (poorly fitted). Figure 2 shows such a seriously biased result for all the linear regressions. The seven non-linear regression algorithms raised no serious over-fitting issues, according to the evaluated over-fitting index values that are greater than 0.6.

**Figure 3:**
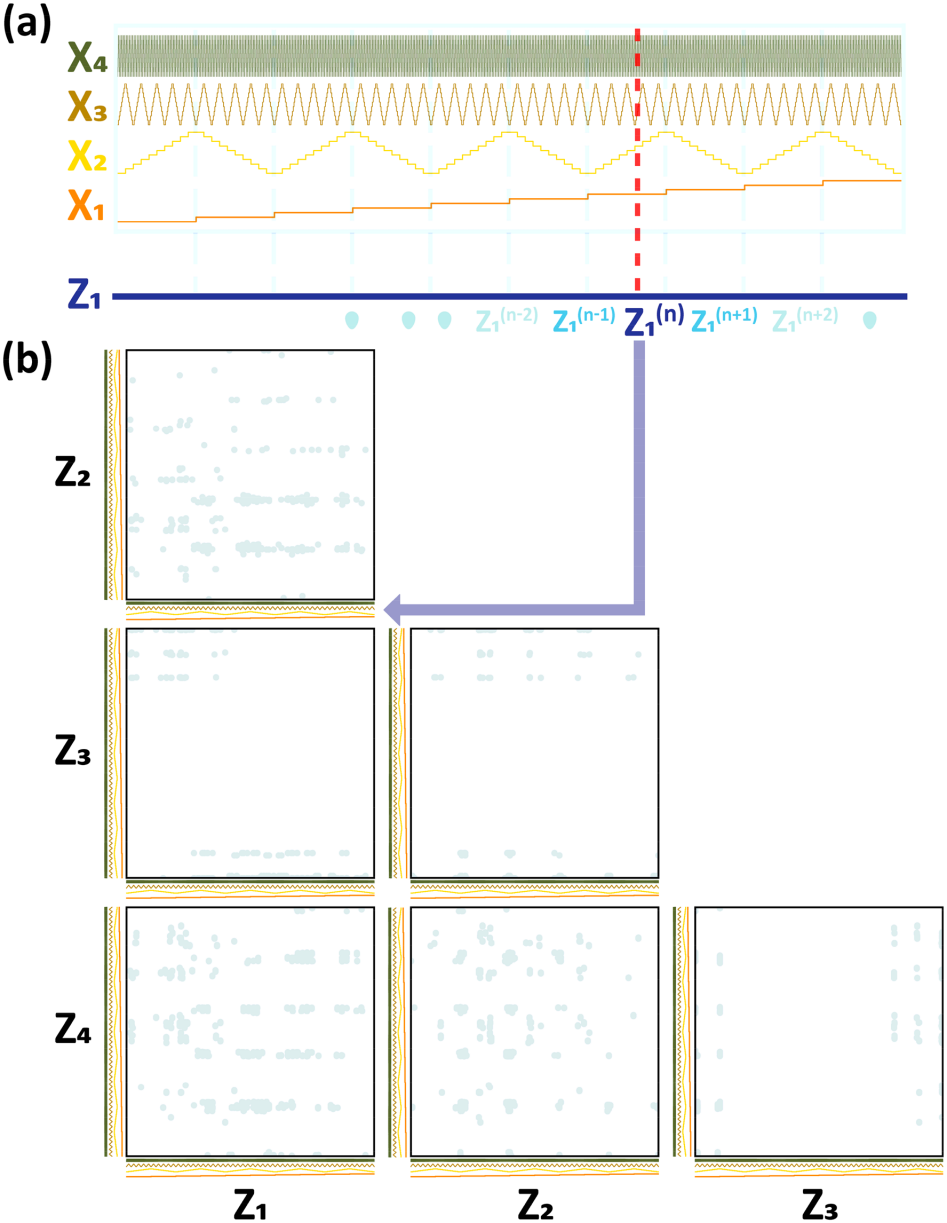

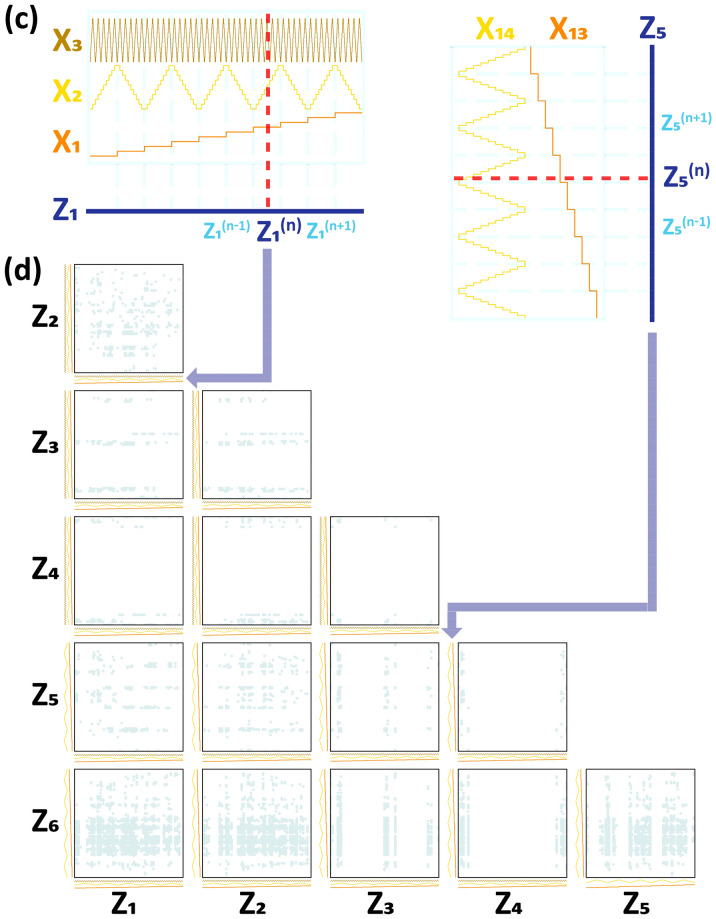
(**a**) HRS operation principle; four 10-level-descretized input features (x _1_–x_4_) with four different periodicities, 2L for x_1_, 1/5L for x_2_, 1/50L for x_3_, and 1/500L for x_4_ (L is the full width of the HSR plot). z_1_ is a new HRS-merged input feature representing x_1_–x_4_. (**b**) the TMCP steel dataset distribution plotted on the HRS representation space. Six pair-wise 2-D scatter plots consisting of z_1_–z_4_, visualize the data distribution in much simpler manner. (**c**) alternative x_i_-z_j_ feature mapping and (d) the corresponding15 pair-wise 2-D HRS scatter plots consisting of z_1_–z_6_.

We focused on real-world data of a dirty nature in the present investigation. The connotations of the term 'dirty' is a data distribution-related problem. The collected data are not identically and independently distributed (IID) random data, and the distribution for some input features (descriptors and attributes) is discrete and biased. ML works if the output loss (the squared residual between real and model-predicted outputs) can be approximated to a Gaussian distribution. The input-feature distribution does not necessarily have to be an IID-Gaussian distribution. However, such a highly biased non-IID data distribution would not be beneficial to ML regression. A conventional pair distribution representation of data distribution confirmed the highly biased, non-IID data nature in our previous report^19^, which also is reconfirmed by the HRS representation of the data distribution in Fig. 3b. The HRS plot is much easier to read than the typical pair-wise 2-D distribution plot.

### NSGA-II execution for optimization of KRR, RF, Gradient boost, XG boost, SVR, KNN, and GPR regression results

The NSGA-II was employed for an inverse design (prediction) using fully trained KRR, RF, Gradient boost, XG boost, SVR, KNN, and GPR regression models. The objective functions (output features: YS and UTS) were maximized by adjusting a decision variable (input feature, descriptor, or attribute: x_1_–x_16_) based on a principle of natural selection that involves both the principles for excellency preservation and diversity pursuit. To achieve multi-objective optimization, the NSGA was developed by employing a Pareto optimality theory, and was reinforced by a large-scale elitism strategy^39^. The elitist-reinforced NSGA, the so-called NSGA-II^38^, is a very robust multi-objective optimization algorithm for materials discovery^50–53^, but its performance is restricted to double-objective problems^40,41^. The NSGA-III was recently introduced to tackle multi-objective optimization issues^40,41^. In this context, we adopted the NSGA-II since we dealt with only two objective functions, YS and UTS, in the present investigation. A more detailed introduction for NSGA-II is aptly described in our previous reports^50–53^.

GAs have previously been used in association with ANNs^5,19,35–37,42–44^. In those cases, however, single-objective problems were the only concern. Neither NSGA-II nor NSGA-III has ever been used for an inverse prediction based on any type of ML regression model. The objective functions of our optimization problem were YS and UTS, which are the output features for all of the ML models, and the decision variables were the 16 input features. Both objective functions should be simultaneously maximized via NSGA-II iteration. We repeated the NSGA-II execution 200 times for each ML algorithm. Each NSGA-II execution produced 200 generations with a population size of 50 per each generation. Every NSGA-II execution provided a very narrow Pareto frontier in the last (200th) generation, so that we randomly collected one representative solution from the first Pareto frontier in the last generation for each NSGA-II execution. In other words, each NSGA-II execution yielded 200 generations and the last (200th) generation showed an inordinately concentrated objective function space so that the Pareto sorting of this final solution ended up with only a few concentrated Pareto groups. Consequently, we finally collected a solution from the first Pareto frontier of the 200th generation for each NSGA-II execution and eventually a total of 200 solutions for each ML algorithm.

**Figure 4:**
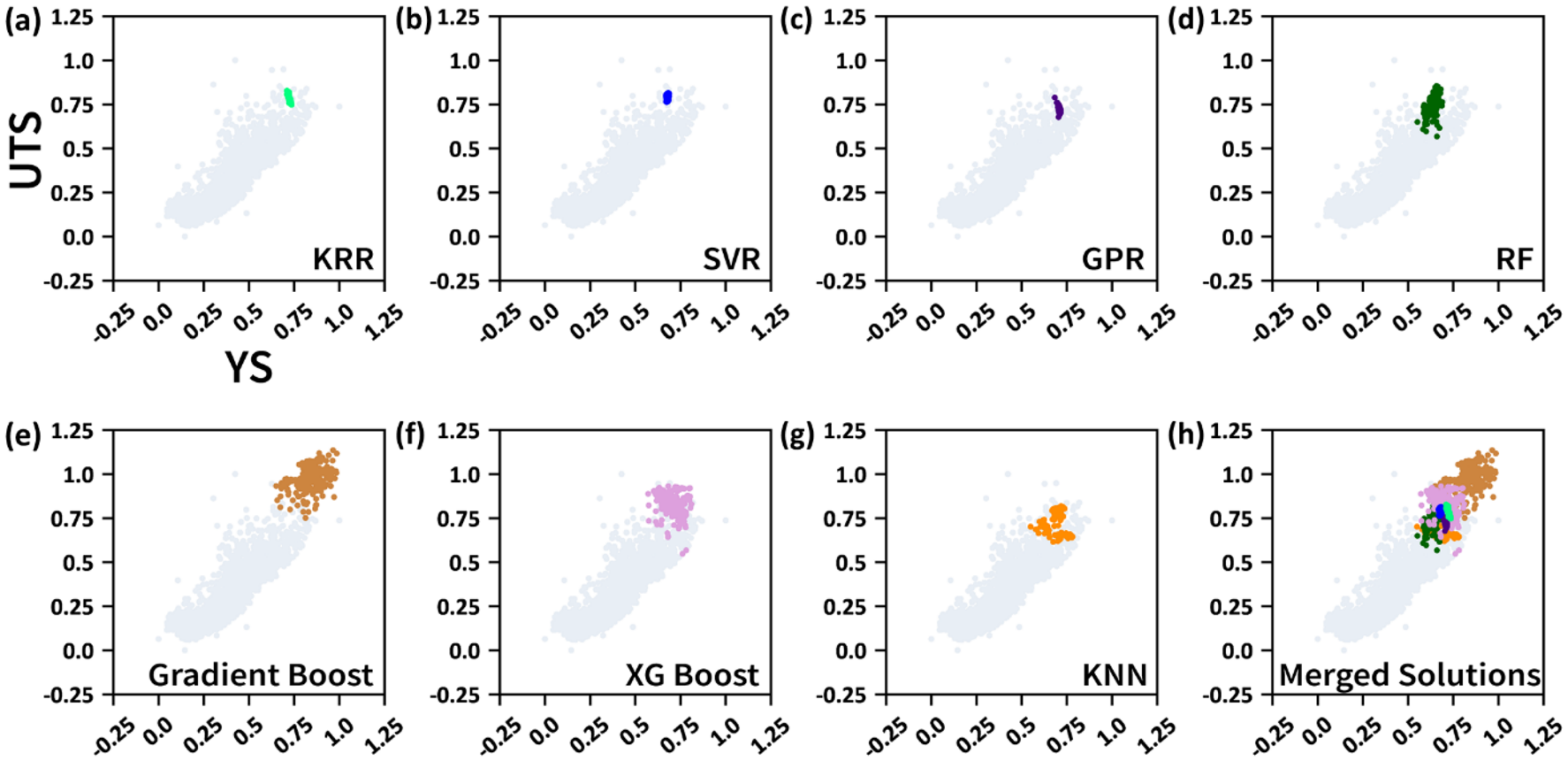
The inverse-predicted (NSGA-II-maximized) YS and UTS for (**a**) KRR, (**b**) SVR, (**c**) GPR, (**d**) RF, (**e**) Gradient Boost, (**f**) XG Boost, and (**g**) KNN regression models, obtained from the NSGA-II-driven inverse prediction for the seven non-linear ML algorithms along with the entire dataset, marked in different colors. (**h**) the merged solutions from all the seven ML algorithms. The entire dataset was also plotted as a background.

Those who are not completely familiar with the NSGA-II algorithm would misunderstand that NSGA-II only works best for what is known as a compromise (trade-off) problem. As can be clearly seen in Fig. 4, the YS-UTS relationship is not trade-off, but roughly a linear relationship. The NSGA-II worked out, however. Because the YS-UTS plot has a broad distribution (although the overall shape is roughly linear), the NSGA-II searched a Pareto frontier in such a broad YS-UTS distribution. More importantly, the objective functions that are not in the trade-off relationship are still applicable for NSGA-II algorithms. The point is that the NSGA-II algorithm was designed for trade-off problems, but it works well for non-trade-off cases as well.

Rather than pursuing diversity, the convergence seemed to take place preferably while 200 generations were produced for a single NSGA-II execution. To artificially secure a certain extent of diversity we executed the NSGA-II execution independently 200 times for each ML algorithm, and collected the final solution by nominating one solution from each NSGA-II execution. Figure 4 shows those solutions in the objective function space for each of the ML algorithms. Therefore, the final inverse prediction results involving 200 solutions for each of the ML algorithms (200 TMCP alloy candidates) are graphically represented in Fig. 4. The solutions from KRR, SVR, and GPR are concentrated in a small region, but those from KNN, RF, Gradient boost, and XG boost are distributed across a relatively large area. Except for the solution of the inverse prediction result for Gradient boost, all the others overlapped considerably with the experimental data in the high YS/UTS region. The Gradient Boost solution is distinctively located in a large area beyond the experimental data. According to the ML fundamentals, this sort of outstanding result for the Gradient boost would not be welcome but rather apt to be discarded since it could be an awkward outcome originating from severe overfitting. It is pointless to expect any type of ML algorithm to predict some excessively promising output by comparison with the training data. In terms of both diversity and excellency, the XG Boost solution would be the most promising of the seven ML algorithms. The KNN, KRR, SVR, RF, and GPR solutions seem a bit less promising since they exhibit both narrower distributions (low diversity) and lower values for YS/UTS (low excellency) by comparison with the XG boost solution. However, the lesson we must learn from the above-described inverse prediction result is that there is no point in finding an algorithm that outperforms the others. Instead of using a single superior model, it makes more sense to average the outcome of all the seven ML algorithms when predicting YS/UTS.

**Figure 5:**
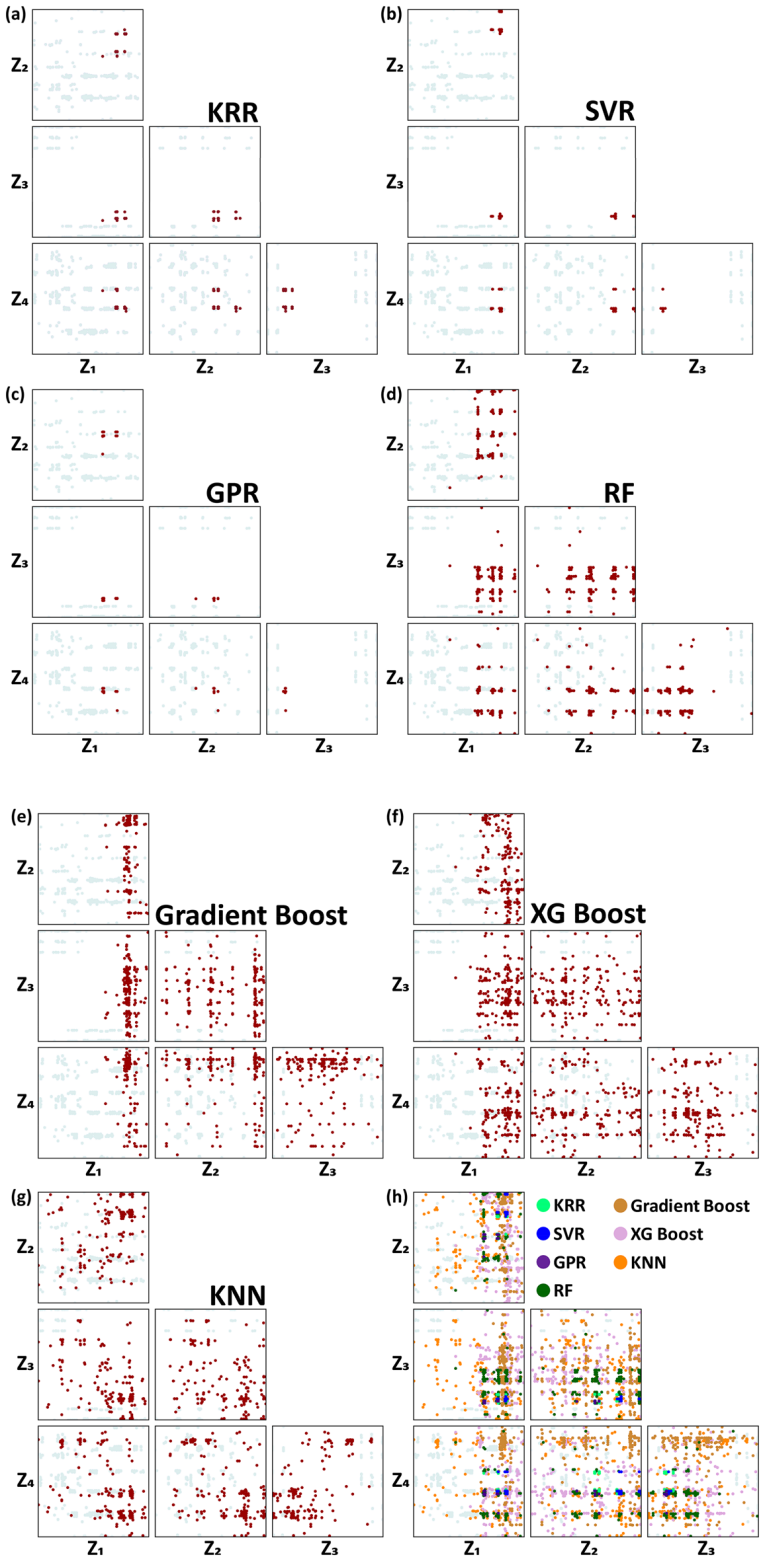
The HRS representation of inverse-predicted solutions for (**a**) KRR, (**b**) SVR, (**c**) GPR, (**d**) RF, (**e**) Gradient Boost, (**f**) XG Boost, and (**g**) KNN regression models, plotted on the HRS represented input feature space, consisting of six pair-wise 2-D scatter plots consisting of z_1_–z_4_, which are considered as an HRS representation unit. The entire dataset was also plotted as a background and the solutions from each ML algorithm are highlighted in dark red color. (**h**) The solutions from all the seven ML algorithms are merged in a single HRS representation unit. Each axis for the six pair-wise 2-D plots designate z_1_–z_4_, and the formulation principle for z_1_–z_4_ was presented in Fig. 3a.

**Figure 6:**
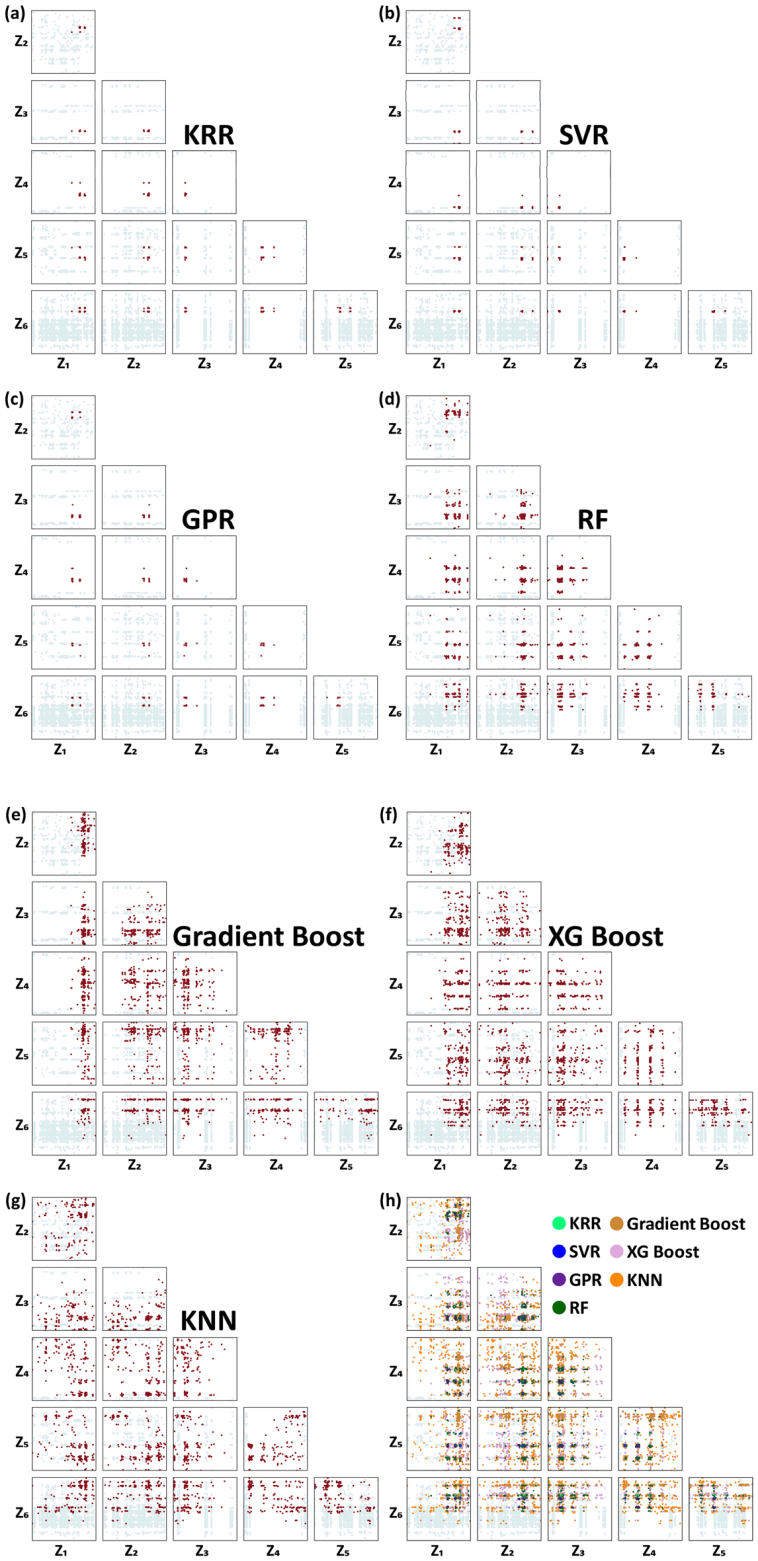
Alternative HRS representation; 15 2-D HRS plots involving six z_j_ features (four z_j_ features (z_1_–z_4_), each of which was mapped from three x_i_ features and two z_j_ features (z_5_ and z_6_) from two x_i_ features per each), considered as an HRS representation unit. Inverse-predicted solutions for (**a**) KRR, (**b**) SVR, (**c**) GPR, (**d**) RF, (**e**) Gradient Boost, (**f**) XG Boost, and (**g**) KNN regression models. The entire dataset was also plotted as a background and the solutions from each ML algorithm are highlighted in dark red color. (**h**) The solutions from all the seven ML algorithms are merged in a single HRS representation unit.

As Fig. 4 shows, all solutions are concentrated in a high YS/UTS area and mostly overlap one another. It should be noted that almost all multi-objective optimization studies deal with the diversity in the objective function (fitness) space. Diversity in the decision variable space has never been studied extensively, although there have been attempts to deal with this issue^54^. It is also important to examine the diversity issue in the decision variable (input feature) space and more importantly, the overlap between inverse-predicted solutions from different ML algorithms in the decision variable space should be systematically examined. For this sake, it is worthwhile to visualize the solution (the inverse prediction result) in the decision variable (input-feature) space. The solution points in the input-feature space were visualized using HRS, as shown in Fig. 5. Figure 6 shows the same data represented using a different HRS setting with better resolution, as discussed in the Computational details section. The YS/UTS value can also be plotted in terms of hue in the HRS space, which is available in the “Supplementary Information” (Fig. S2).

The solution distribution in such a high dimensional input-feature (decision variable) space differed from one ML algorithm to another. This means that the terrain of the predicted objective function (output feature) differed for each of the fully trained ML models. The KRR, SVR, and GPR returned a narrow solution distribution not only in the objective function space Fig. 4 but also in the decision variable space (Figs. 5 and 6), meaning that the objective function terrain for the fully trained KRR, SVR, and GPR might be similar to a uniquely convex topography, while the KNN, RF, Gradient boost, and XG boost resulted in a highly diverse and wider solution distribution, implying that the objective function terrain for the fully trained KNN, RF, Gradient boost, and XG boost should be bumpier in the 16-dimensional decision variable space. In scrutinizing the solutions, it appears that a certain portion of KRR and SVR solutions overlapped in the HRS space. Also, the GPR solution does not seem to heavily deviate from those for both KRR and SVR. The solution distribution for the KNN, RF, Gradient boost, and XG boost algorithms were so widely scattered that the solutions considerably overlapped one another and also overlapped the solutions from KRR, SVR, and GPR. All the solutions for every ML algorithm are co-plotted with different colors in Fig. 5h, showing the overlap in some areas (although it looks disordered).

It is evident that every ML algorithm gave rise to different inverse predictions despite a certain degree of overlap. To maximize the objective function (YS/UTS) values, NSGA-II herded alloy candidates in a high YS/UTS direction, which differed from one ML algorithm to another. A clear finding is that the NSGA-II execution worked well for all seven of the ML algorithms, but the finally optimized solutions were highly diverse depending on the selected ML algorithm, and there was no common global optimum, meaning that the solutions were not uniquely converged on a narrow area but scattered across a large input feature space. The exceptions were the KRR and SVR solutions that clearly overlapped within a narrow region. A more serious issue, however, is the fact that some of the solutions from all the algorithms were located out of the training data distribution. To confirm this, we plotted all the data for the 5473 TMCP alloys as background for all of the HRS plots in order accomplish systematic comparisons, as shown in Fig. 5. Many of the solutions were located either inside or nearby the training data area, but some were located far away from the training data distribution.

Those with a dearth of knowledge concerning ML might unconditionally welcome higher YS/UTS predictions without checking on the decision variable space. If the training dataset had a continuously dispersed data distribution, the prediction results would have been more reasonable. On the contrary, if a dataset exhibits a highly biased, skewed, discrete distribution similar to our TMCP steel dataset, then an in-depth examination of the solution space (= decision variable space = input feature space) should definitely be accounted for along with the conventional examination of objective function space. It is necessary to investigate an extremely high dimensional solution space. Although we introduced the HRS graphical representation in order to visualize the 16-D solution space, the HRS approach remains far from complete. We still need to examine the six 2-D HRS plots (or 15 2-D HRS plots) at the same time to completely understand the data status. It should, however, be noted that with the introduction of HRS, the 120 2-D plots for the conventional pair-wise data representation have been significantly reduced to just six (or 15) plots.

It is not surprising that the seven well-established ML algorithms ended up with quite different inverse-predicted solutions, albeit with a certain degree of overlap. The ML and metaheuristics never gave a definite common global optimal solution, but instead every ML algorithm gave rise to their own unique solution. On this ground, it would be extremely risky to select an ML algorithm only by relying on the goodness of fit, since we confirmed that the selected seven ML algorithms exhibited quite a satisfactory goodness of fit with very different solutions. Although all the ML algorithms exhibited similar inverse-predicted YS/UTS values, they are widely dispersed in the high dimensional solution space. The data-driven approach is not like a traditional rule-based analysis that leads to consistent, definite outcomes under all circumstances. Due to the heuristic, ad-hoc nature of data-driven approaches, each of the seven ML algorithms led to quite a scattered inverse-predicted result differing from one another. Therefore, the adoption of a particular ML algorithm selected on the basis of the goodness of fit for a certain problem would be risky. Instead, a group of ML algorithms similar to our so-called “integrated ML platform” should be adopted for a single problem with a single dataset. By doing so, various solutions could be attained. Thereafter, final solutions could be recommended by analyzing a variety of the solutions that result from various ML algorithms.

Although we provided all the inverse-predicted solutions from the seven ML algorithms and also graphically recommended some overlapped solutions, we did not extensively examine them in terms of the materials side in contrast to our previous report^19^ wherein the materials side of selected candidates were examined in detail. Several alloys that were inverse-predicted by ANN/NSGA-II algorithms in our previous report^19^ overlap with the solution in the present investigation. The previous solution was validated by field experts and also backed up by well-established theoretical calculation (e.g., thermos-Calc.)^55^, through which the Ae1 and Ae3 temperatures were calculated and the precipitation reactions of (Ti,Nb)C and VC were evaluated. The calculated thermodynamic conditions from the previous solution are quite reasonable in terms of conventional hot rolling and subsequent heat-treatment processes. Accordingly, the solution in the present investigation could be equally interpreted by refereeing to the previous result. An in-depth study of the performance and microstructures of the predicted materials will be dealt with separately in the near future. Of course, most of the alloy candidates suggested by the integrated ML platform (listed in Table S1) were within a reasonable range by referring to the common sense of field experts. The amount of C, Mn, Nb, and Si was well controlled by the NSGA-II optimization process and the increased amount of these key components in the solution seemed contributive to improving the strength and positive for the TMCP application. The massive solution data presented in Table S1 were succinctly rearranged as 1-D distribution plots for every input feature as shown in Fig. S3, wherein one can clearly detect a narrowed solution range in comparison to the entire dataset distribution.

The scope of the present investigation was limited to a methodology introduction in order to accomplish an efficient data-driven metal alloy design, and also to warn materials scientists regarding the ad-hoc, heuristic nature of ML approaches. A key lesson that we have learned from the present study is that we must not blind-trust the ML-based, inverse-predicted result unless the solution distribution in the input feature space is precisely examined by employing at least more than several ML algorithms simultaneously.

## Computational details

### Data acquisition and descriptor extraction

The dataset acquired from Hyundai Steel Co. included 5473 alloy entries with 14 alloy components, two processing variables, and the two materials properties of yield strength (YS) and ultimate tensile strength (UTS). X_1–14_ are the elemental compositions for C, Si, Mn, P, S, Cu, Sn, Ni, Cr, Mo, V, Nb, Ti, and Ca. X_15_ and X_16_ represent heating time and temperature, respectively. All the other processing conditions were fixed. Since the dataset was gleaned from real-world industry, better-organized features representing microstructural and physical attributes were not available, although a recent trend is to introduce intermediate descriptors to enhance predictability^2^. Consequently, the problem was simply set up with 16 input features and 2 output features. All the input/output features were min–max-normalized such that each feature ranged from 0 to 1, The distribution of each feature was well-described in the previous report^19^.

It is obvious that some of the input features could not approximate a continuous random variable, but these were in a highly biased and discrete state, and some of the continuous variables were not in a unimodal normal distribution, but were, instead, in the form of multi-modal distributions. The so-called ‘dirty nature' of the dataset was confirmed by both pair-wise data distribution plots and Pearson correlation analysis, which also revealed no severely correlated feature pairs in our dataset. However, we did not employ systematic ways of mapping data from various distributions to a normal distribution such as that seen in Box–Cox^56^, Yeo–Johnson^57^, and Quantile transformations^58^, since the conventional ML approach places no strict restrictions on the input data distribution. Note that the only restriction of the ML data is that the residual (difference between real and ML-predicted output feature) should be in a normal distribution, which refers to the L2 loss minimization (= the maximum likelihood estimation) that is a crucial prerequisite for successful ML. If this condition is met, the training dataset requires no other restrictions. Nonetheless, the dirty nature of the dataset would not seem favorable for the ML-based regression and the ensuing inverse prediction process.

The highly biased discrete data status was visually represented as 120 2-dimensional pair-distribution plots along with a Pearson coefficient matrix in the previous report^19^. Such intricate data visualization complicates the data status. Many high-dimensional data visualization techniques have been reported thus far. These include the pair-wise scatter plot that we used in the previous report^19^, a pair-wise correlation matrix and heatmap^59^, a tree diagram (dendrogram)^60^, Chernoff faces^61^, and many more^62^. In addition, more advanced strategies that are based on both statistical science and machine learning are also available for high-dimension complicated data, which is referred to as ‘big data' in many domains. These types of data reduction and visualization strategies include early-stage basic algorithms such as principal component analysis (PCA)^63^ and classical multidimensional scaling (MDS)^64^, along with more advanced recent algorithms such as stochastic neighbor embedding (SNE and t-SNE)^65,66^, and more recently developed algorithms such as uniform manifold approximation and projection (UMAP)^67^, ChemTreeMap^68^, tree map (TMAP)^69^, and the Potential of Heat-diffusion for Affinity-based Transition Embedding (PHATE)^70^.

Although many high-dimensional data visualization strategies have been reported^59–70^, none were adequate for our 16-dimensional data visualization. A typical PCA-based data dimension reduction and the ensuing 2-D projected data visualization causes too much information loss in the course of the data reduction process. We adopted an alternative multi-dimensional data visualization methodology referred to as the holographic research strategy (HRS), which was developed by Margitfalvi et al.^45–48^. The HRS can effectively visualize a high-dimensional dataset and minimize the loss of data information. Although the above-described high-dimensional data visualization strategies have been used for many problems in various domains, the HRS has not been widely utilized. It should be noted, however, that the HRS has been successfully used for the discovery (optimization) of multi-compositional inorganic catalysts^45–48^. Our understanding is that HRS deserves to be spotlighted since its potential for multi-dimensional data visualization is promising in cases where the dimensions of the data are not too high (< 20).

We combined four input features as a single feature in the manner described in Fig. 3a: x_1_–x_4_ as z_1_, x_5_–x_8_ as z_2_, x_9_–x_12_ as z_3_, and x_13_–x_16_ as z_4_. There are a large number of different combinations, any of which are acceptable. First, all the x_i_ features were discretized such that the min–max-normalized x_i_ feature (in the range 0–1) was classified into 10 levels. Thereafter, every x_i_ feature was rearranged as a periodical with the same amplitude (= 10 levels) but with a different periodicity. Thereafter, four x_i_ features with different periodicities were stacked one-on-another in turn as shown in Fig. 3a, and the vertical red dashed line bisecting all four x_i_ periodicals at different levels for each x_i_ feature ultimately constitute a certain single z_j_ value at the bottom. This means that the z_j_ value exactly represents those four x_i_ feature values that come across the vertical red line. In this way, the four dimensional input features (e.g. x_1_–x_4_) were mapped to one dimensional feature (z_1_) with no loss of information. Of course, we ignored a small amount of information loss caused by the x_i_ feature discretization process. Consequently, the 16-dimensional input features (x_i_, i = 1–16) were efficiently reduced to 4-dimensional input features (z_j_, j = 1–4), and thereafter any of the pairs out of the four z_j_ features could be drawn on a typical 2-dimensional (2-D) pair-wise plot, and eventually six 2-D plots could represent the original 16-dimensional data distribution.

Figure 3b shows six 2-D scatter plots (so-called 2-D HRS plots) elucidating the total data distribution that the four z_j_s (z_1_–z_4_) constitute. By comparison with a conventional pair-wise scatter plot using 16 x_i_ features that produce 120 2-D pair-wise plots, only six pair-wise scatter plots using four z_j_ features should be much more succinct and easier to understand. Finally, the TMCP steel dataset plotted on the HRS representation space shown in Fig. 3b also exhibits a very scattered, biased, and non-IID data status, as already proven in the conventional 120 pair-wise scatter plots in our previous report^48^. The YS and UTS were also plotted on the HRS representation space in terms of the hue, as shown in Fig. S5, which is available in the “Supplementary Information”. Figure S5 shows that no conversed area exhibiting high YS/UTS values exists on the HRS representation space, which implies that simple linear regression ML models would not suffice in this complicated 16-dimensional input feature space.

The x_i_ feature with the highest frequency (the smallest periodicity) would lose its resolution on the 2-D HRS diagram and make the HRS representation impractical. Note that the fourth x_i_ feature had a periodicity of 1/500L. Unless the 2-D HRS plot were zoomed in, the fourth x_i_ feature would have no effect. If a z_j_ feature is mapped from three (or two) x_i_ features, then adequate resolution can be ensured on the 2-D HRS plot. In such a case, we have six z_j_ features that result in fifteen 2-D HRS plots. Figure 3c elucidates the x_i_-to-z_j_ feature mapping process schematically: x_1_–x_3_ as z_1_, x_4_–x_6_ as z_2_, x_7_–x_9_ as z_3_, x_10_–x_12_ as z_4_, x_13_–x_14_ as z_5_, and x_15_–x_16_ as z_6_. The fifteen 2-D HRS plots (Fig. 3d) are much easier to read than the original 120 plots, although the fifteen plots still seem inordinate when compared to the six 2-D HRS plots with four z_j_ features (Fig. 3b). Figure 6 shows fifteen 2-D HRS plots involving six z_j_ features to visualize the inverse-predicted solutions for the seven ML algorithms. There would be no information loss caused by a high frequency x_i_ feature when a z_j_ feature was mapped from two or three x_i_ features. Those who are uncomfortable with the six 2-D HRS plots can refer to Fig. 6. However, the six 2-D HRS plots along with four z_j_ features, as shown in Fig. 3b, still have reasonable resolution in comprehending the data distribution. In addition, it is also possible to create a 3-D HRS plot as shown in Fig. S5. Despite the advantage that only a single 3-D HRS plot is sufficient to visualize the 16-dimensional input feature space, the 5-level discretization of x_i_ features was inevitable since each z_j_ feature is mapped from six or five x_i_ features. The sparser x_i_ feature discretization and the higher frequency would never lead to proper resolution in this 3-D HRS plot and a considerable information loss would be unavoidable.

### ML model selection

In this study we reviewed three categories of ML algorithms involving 16 versions. The first category includes the so-called regularized linear regression algorithms: Ridge regression, least absolute shrinkage and selection operator (Lasso) regression, least-angle regression (LARS), elastic net regression (ENR), kernel ridge regression (KRR), Bayesian ridge regression (BRR), and Bayesian automatic relevance determination (ARD). Basic linear regression was also incorporated in this first group as a baseline method. L2 (Ridge) and L1 (Lasso) regularizations are the most generally adopted form of regularization. L2 and L1 regularizations incorporate the L2 and L1 norms of weights as a penalty in the loss function. Other supplementary regularization algorithms such as LARS, KRR, BRR, and ARD are also employed. The LARS algorithm is a special version of the Lasso problem. In order to account for non-linearity, the KRR algorithm incorporates kernels such as polynomial, radial basis function (RBF), sigmoid, and matern. The BRR and ARD algorithms incorporate the Bayesian approach. The Bayesian approach is used for BRR and ARD regressions, which are used to make a final prediction that is distributive rather than deterministic. The prediction is not deterministic because it is made with a certain mean and variance originating from the fact that the fitted parameters are distributed rather than deterministic.

The second category includes ensemble algorithms. The ensemble algorithm model consists of a collection of individual small models (base estimators). The predictability of an individual model is limited since it is likely to give rise to over-fitting, but combining many such weak models in an ensemble leads to a much improved level of predictability. The most common individual weak model is a decision tree with different depths determined by the chosen algorithm. For example, Ada boost uses stumps that are trees with only a single-level depth and the other ensemble algorithms use trees with deeper depth levels. We employed several tree ensemble algorithms such as random forest (RF), adaptive (Ada) boost, Gradient boost, and extreme Gradient (XG) boost. The ensemble algorithm generally has two representative model implementations, i.e., bagging and boosting. The bagging is for use with RF and each base model is treated independently, while the boosting for use with Ada, Gradient, and XG boost algorithms sequentially place greater weight on the data of each model (on the feature), which could connect the wrong predictions and result in high rates of error.

**Figure 7:**
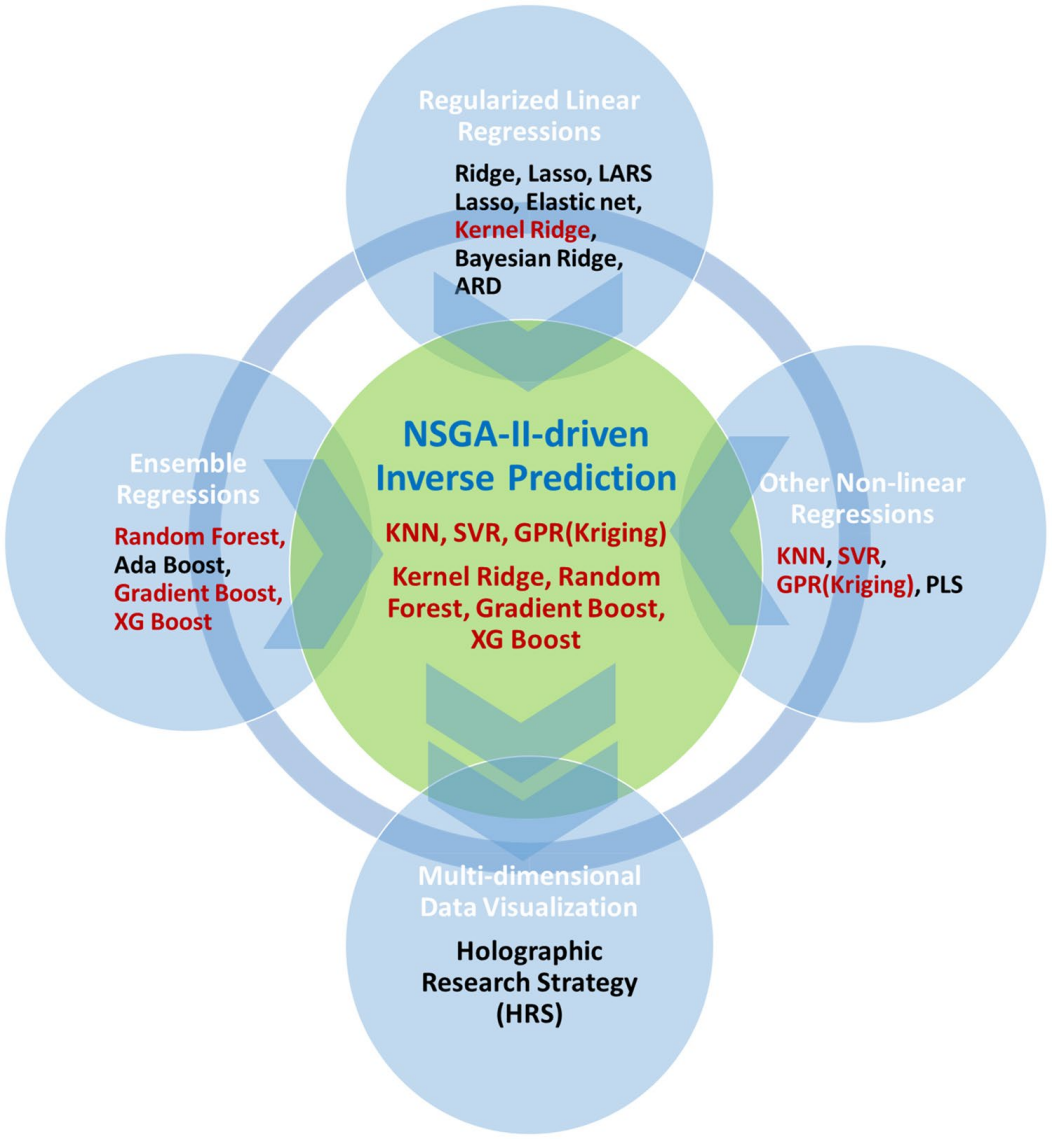
The overall graphical description for the integrated ML platform. Three groups of ML algorithms, the NSGA-II-driven inverse prediction, and the high-dimensional data visualization method are given.

We also incorporated some non-linear regression ML algorithms such as support vector machine regression (SVR), k-nearest neighbors (KNN), and Gaussian process regression (GPR). An additional linear regression algorithm, partial least square (PLS), was also introduced. Prior to the advent of deep learning, SVR outperformed conventional ANNs in sorting out many problems. The use of kernels such as polynomial, radial basis function (RBF), sigmoid, and matern kernels is essential for SVR and an RBF kernel was selected from the hyper-parameter optimization process for the present SVR. KNN, which is known as the simplest ML algorithm, is strongly dependent on an ad hoc determination of appropriate k values. Therefore, we obtained the best k value from the hyper-parameter selection procedures. GPR, so-called kriging, has recently attracted a great deal of attention as it often has been used as a surrogate function for Bayesian optimization^71^. As with BRR and ARD regression algorithms, the GPR prediction was also made with a certain mean and covariance. In particular, we noted that KNN and GPR are parameter-free (or model-free) ML algorithms that differ from most other ML algorithms that aim to search for optimal parameters constituting a ML model. PLS is a traditional linear regression method that is even applicable to problems with fewer data points than the number of input features, which is a situation far from ours. Our problem was ameliorated by considering the dataset size (5473 data points) relative to the number of input features (16). More interestingly, we omitted ANN here since it had already been employed for the same regression in our previous report^19^. Several ML algorithms were used for the ensuing inverse prediction based on the NSGA-II, although only the ANN(= DNN) was used for the inverse prediction in the previous report^19^. Figure 7 features a succinct schematic that describes the entire ML platform and the ensuing inverse prediction process as well as the high-dimensional data visualization. All the above-described regression algorithms are available in the Scikit-learn module^72^ with well-established default hyper-parameters, some of which were incorporated here. We performed a hyper-parameter optimization process, however, to determine some of the key hyper-parameters. The hyper-parameter screening process will be discussed in the following subsection.

### Training, validation, and test dataset splitting

Meticulous care should be taken when splitting the data into training, validation, and test datasets. Only a simple split into training and test datasets was not viable. We adopted two training schemes. First, we adopted a sixfold cross-validation^73–75^ scheme without preparing a holdout test dataset, and the results of validation were used for the hyper-parameter screening. Second, we set aside a holdout test dataset that included 913 alloys, and a 5-fold cross-validation was implemented for the remainder of the data, which included 4560 alloys. We then tested the fully trained model using the holdout test dataset. The optimal hyper-parameters obtained from the preceding 6-fold cross-validation process were employed for the ensuing 5-fold cross-validation and test processes.

Hyper-parameter optimization is one of most important ML issues that has been developed in recent years, and the most efficient way of tackling the issue is the use of Bayesian optimization^71^. Differing from typical deep-learning cases, however, the present problems of a moderate size did not require such an additional high-cost optimization strategy. Instead, we designed an enumerable hyper-parameter search space (mesh) for each algorithm. Each mesh involved a tractable number of hyper-parameter sets that amounted to approximately 100 for testing (the maximum number of hyper-parameter sets was at best 144 for a Gradient boost algorithm). We screened all the hyper-parameter sets by monitoring the validation MSE and R^2^ results evaluated from the 6-fold cross-validation, and eventually pinpointed an optimal hyper-parameter set for each algorithm. All the hyper-parameter sets with their validation MSE and R^2^ values are given in Table S2, wherein the finally selected hyper-parameter set for each algorithm is highlighted.

## Conclusions

An integrated ML platform involving 16 algorithms was developed to achieve YS/UTS predictions from compositional and processing descriptors. The 5473 TMCP steel alloys gleaned from the real-world industry were used to establish training, validation, and test datasets for use with an integrated ML model platform.

Nonlinear ML algorithms outperformed the basic linear regression algorithm as well as its regularized versions. Well-known non-linear regression algorithms such as KRR (with a matern kernel), RF, Gradient boost, XG boost, KNN, SVR, and GPR worked properly but all the others proved to be unacceptable. These seven fully trained non-linear ML algorithms were used to make NSGA-II-driven inverse predictions, and some desirable solutions were obtained.

In addition, the solutions were graphically visualized not only in the conventional low-dimensional objective function space but also in the 16-dimensional decision variable space using the HRS technique, so that the data status could be comprehended in a systematic manner. By visualizing the solution in the input feature space, a real-sense ML prediction was achieved. The ML algorithms never gave a common optimal solution, but every ML algorithm predicted a very diverse solution in the input feature space. We suggest that the adoption of either a single or a few ML algorithms for alloy design is very risky and it also is extremely risky to select an ML algorithm only by relying on the goodness of fit.

The amount of C, Mn, Nb, and Si was well controlled by the NSGA-II optimization process and the increased amount of these key alloying elements in the solution contributed to improving the strength and played a positive role for the TMCP application. The experimental validation should be the next step although most of the alloy candidates suggested in the present investigation were within a reasonable range by referring to the common sense of field experts.

## Supplementary Information

Supplementary Information.

## Data Availability

All data generated or analyzed during this study are included in this published article (and its “Supplementary Information” files) and the datasets used for the integrated ML platform during the current study are available from the corresponding author on reasonable request.

## Code availability

The codes used for this study are available from the GitHub link at https://github.com/socoolblue/integrated_ML_platform.
