# Supplementary material for: A machine-learning-based alloy design platform that enables both forward and inverse predictions for thermo-mechanically controlled processed (TMCP) steel alloys
Source: Sci Rep. 2021 May 26;11:11012. doi: 10.1038/s41598-021-90237-z (PMC8155048; doi:10.1038/s41598-021-90237-z)
Supplement: Supplementary file 1 — Supplementary Information. [file 41598_2021_90237_MOESM1_ESM.docx]

**Supplementary information**

**A machine-learning-based alloy design platform that enables both forward and inverse predictions for thermo-mechanically controlled processed (TMCP) steel alloys**

Jin-Woong Lee,^1,‡^ Chaewon Park,^1,‡^ Byung Do Lee,^1,‡^ Joonseo Park,^1^ Nam Hoon Goo,^2^ and Kee-Sun Sohn^1,*^

^1^Nanotechnology & Advanced Materials Engineering, Sejong University, 209 Neungdong-ro, Gwangjin-gu, Seoul, 143-747, South Korea

^2^Advanced Research Team, Hyundai Steel DangJin Works, DangJin, Chungnam, 31719, Republic of Korea

^‡^These authors contributed equally

*Corresponding authors: [kssohn@sejong.ac.kr](mailto:kssohn@sejong.ac.kr)

**List of Contents**

**Fig. S1.** **a** YS and **b** UTS plotted in terms of the hue on the HRS representation space for the entire TMCP steel dataset.

**Fig. S2.** Alternative HRS representations; 3-D HRS plots for inverse-predicted solutions from the seven non-linear ML algorithms such as KRR, SVR, GPR, RF, Gradient Boost, XG Boost, and KNN. The 3-D HRS plot for the entire dataset is also given.

**Fig. S3.** 1-D distribution of inverse-predicted solutions for every input feature; the entire dataset distribution is in dim grey color and the inverse-predicted solution distribution by KRR, SVR, GPR, RF, Gradient Boost, XG Boost, and KNN algorithms is in yellow color.

**Fig. S4.** Plots of predicted vs. experimental **a** YS and **b** UTS for training and validation datasets for 6-fold cross validation.

**Fig. S5. a** YS and **b** UTS plotted in terms of the hue on the HRS representation space for inverse-predicted solutions from the seven non-linear ML algorithms such as KRR, SVR, GPR, RF, Gradient Boost, XG Boost, and KNN.

**Table S1.** Details of the validation MSE and R^2^ values for all the hyper-parameter sets (6-fold cross-validation with no holdout dataset test). The finally selected set is highlighted in bold font.

**Table S2.** Details of the TMCP alloy candidates nominated by the inverse prediction for the seven non-linear ML algorithms such as KRR, RF, Gradient Boost, XG Boost, SVR, KNN, and GPR.

**Fig. S1**


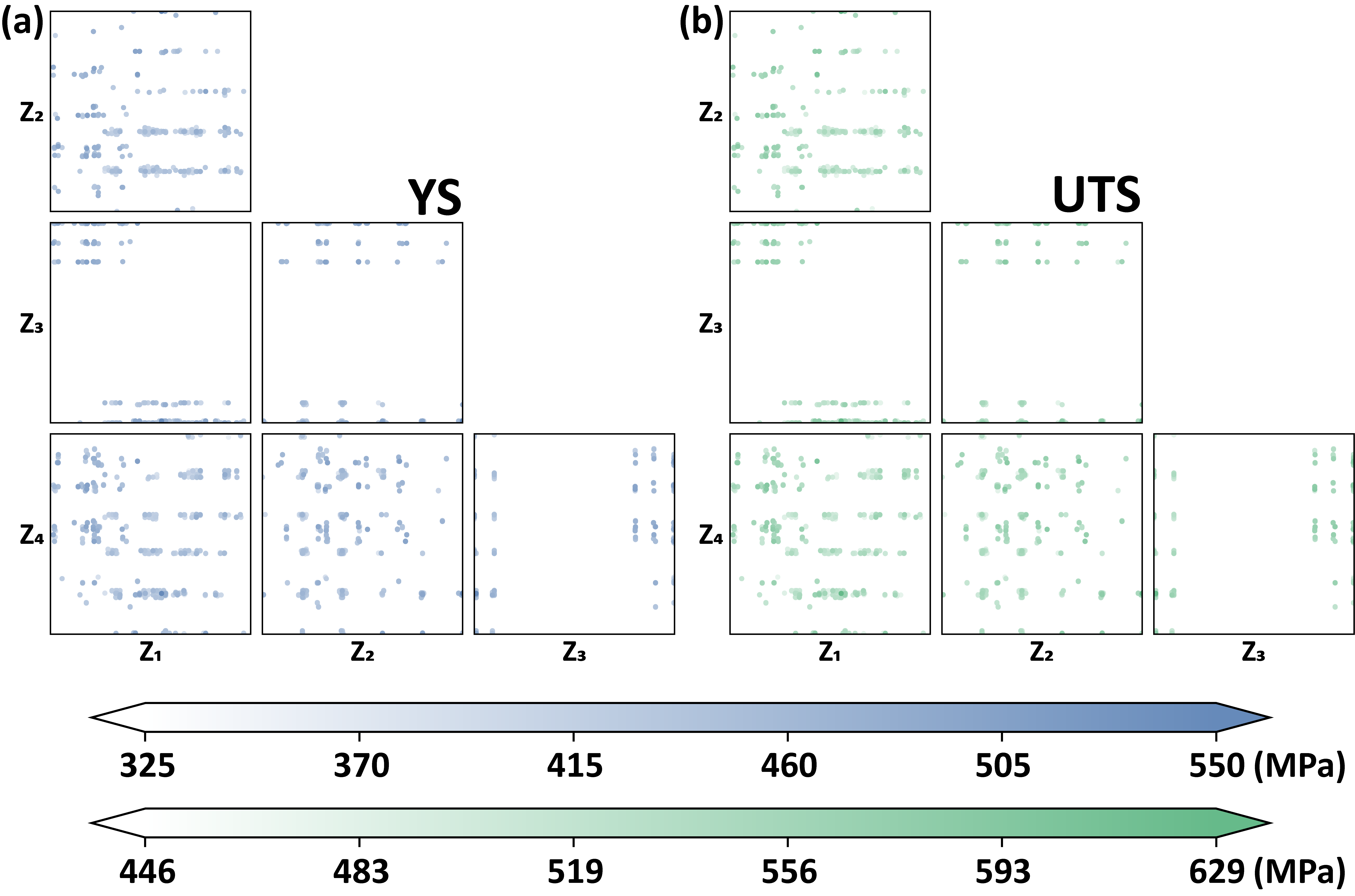


**Fig. S2**


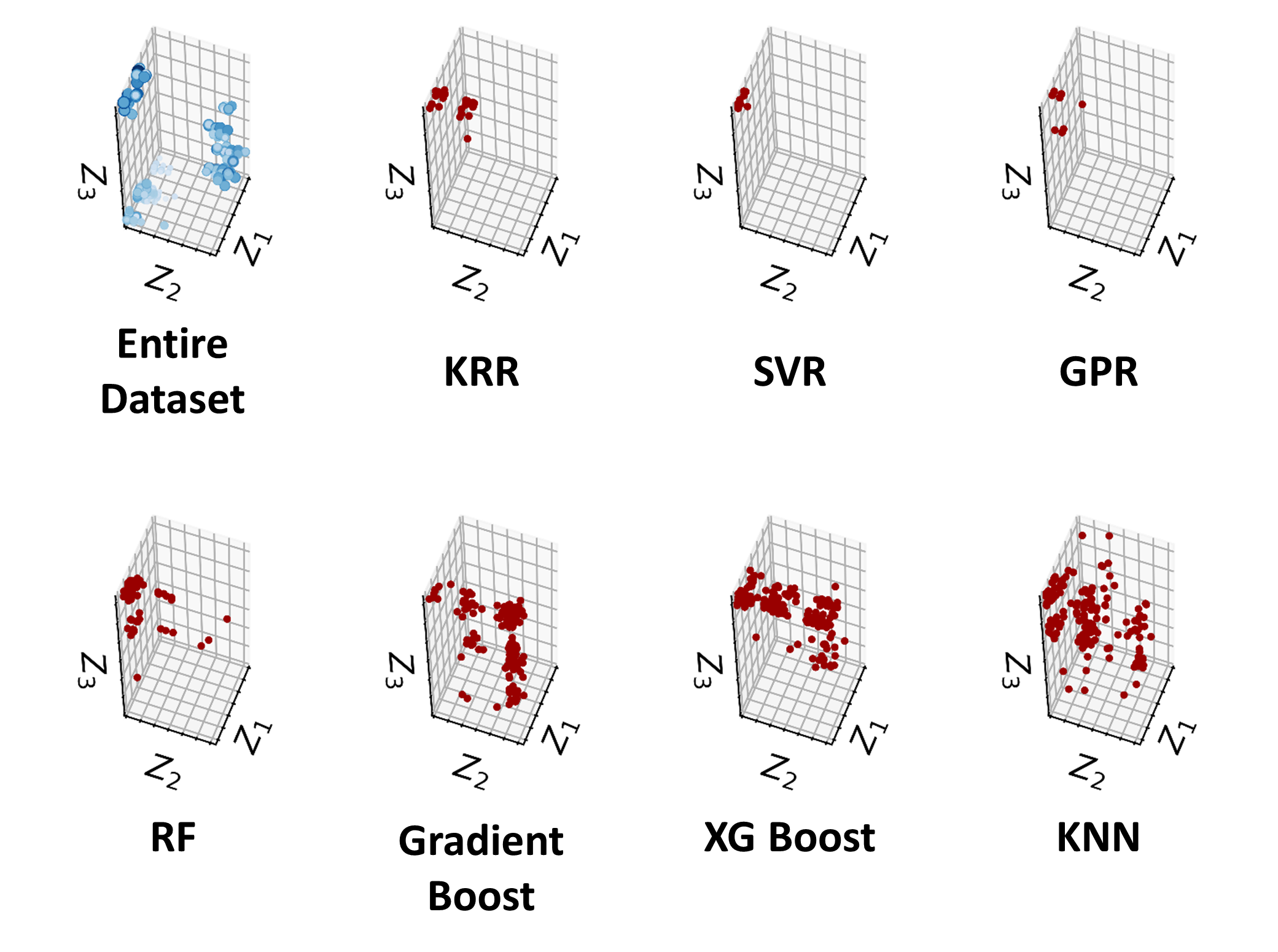


**Fig. S3**


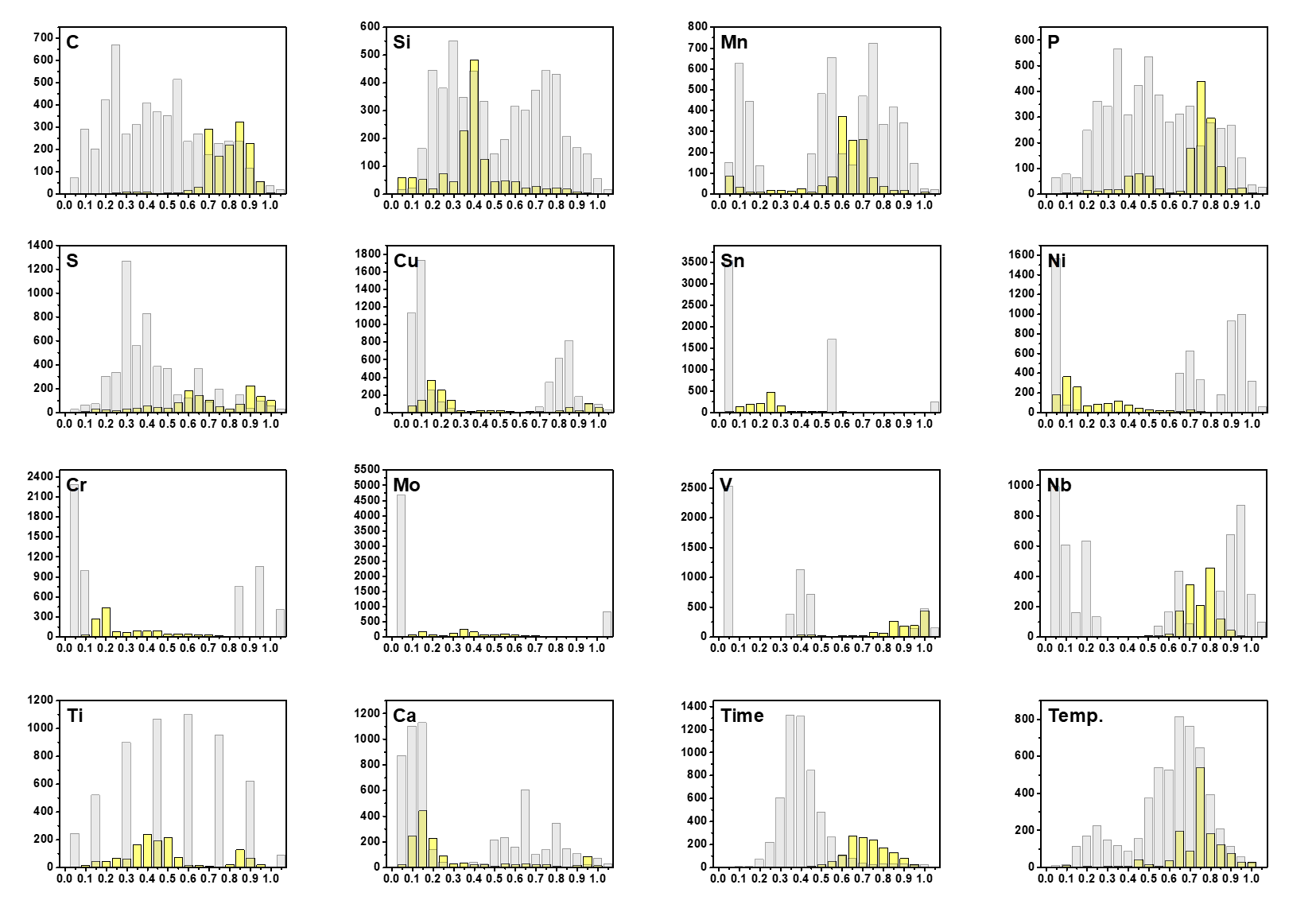


**Fig. S4**

**
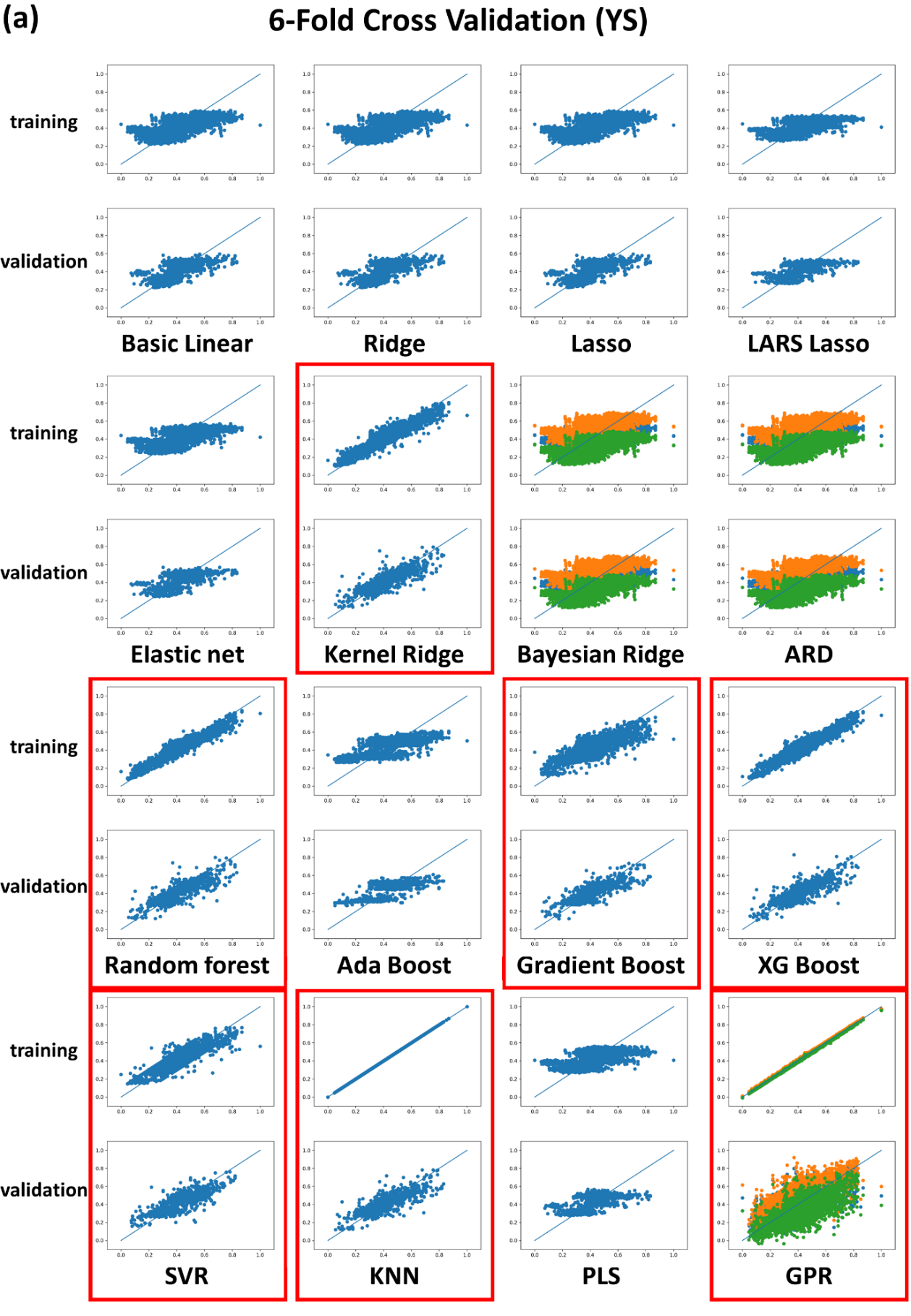
**

**Fig. S4**

**
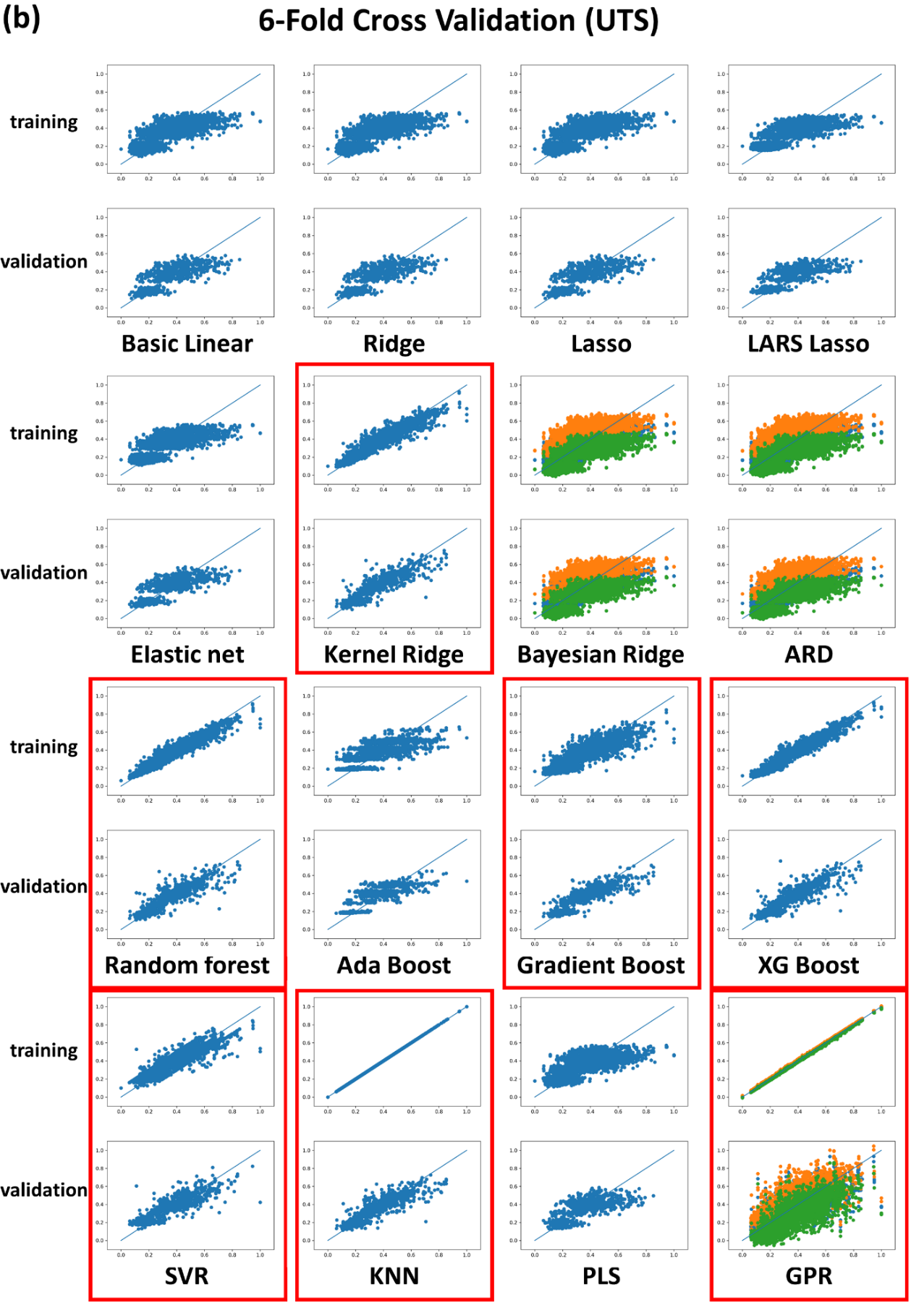
**

**Fig. S5**





**Fig. S5**





**Fig. S5**





**Fig. S5**





**Table S1**

| **KRR** | | | | | | | | | | | | | | | | | |
| --- | --- | --- | --- | --- | --- | --- | --- | --- | --- | --- | --- | --- | --- | --- | --- | --- | --- |
| C | Si | Mn | P | S | Cu | Sn | Ni | Cr | Mo | V | Nb | Ti | Ca | Time | Temp | YS | UTS |
| (wt%) | (wt%) | (wt%) | (wt%) | (wt%) | (wt%) | (wt%) | (wt%) | (wt%) | (wt%) | (wt%) | (wt%) | (wt%) | (wt%) | (s) | (℃) | (Mpa) | (Mpa) |
| 0.078 | 0.25 | 1.40 | 0.012 | 0.003 | 0.043 | 0.001 | 0.014 | 0.042 | 0.003 | 0.041 | 0.039 | 0.014 | 0.0007 | 399 | 1192 | 489 | 587 |
| 0.077 | 0.25 | 1.41 | 0.013 | 0.003 | 0.045 | 0.001 | 0.017 | 0.041 | 0.003 | 0.044 | 0.040 | 0.013 | 0.0006 | 403 | 1176 | 488 | 591 |
| 0.076 | 0.25 | 1.39 | 0.012 | 0.003 | 0.044 | 0.001 | 0.010 | 0.043 | 0.003 | 0.041 | 0.039 | 0.014 | 0.0006 | 397 | 1186 | 489 | 586 |
| 0.076 | 0.25 | 1.39 | 0.012 | 0.003 | 0.042 | 0.001 | 0.012 | 0.044 | 0.003 | 0.042 | 0.040 | 0.014 | 0.0006 | 402 | 1188 | 489 | 586 |
| 0.077 | 0.25 | 1.40 | 0.013 | 0.003 | 0.047 | 0.001 | 0.014 | 0.045 | 0.003 | 0.042 | 0.040 | 0.014 | 0.0007 | 404 | 1189 | 488 | 587 |
| 0.078 | 0.25 | 1.40 | 0.013 | 0.003 | 0.041 | 0.001 | 0.017 | 0.043 | 0.003 | 0.043 | 0.040 | 0.013 | 0.0006 | 404 | 1175 | 487 | 590 |
| 0.078 | 0.26 | 1.42 | 0.012 | 0.003 | 0.038 | 0.001 | 0.011 | 0.042 | 0.003 | 0.043 | 0.039 | 0.013 | 0.0006 | 397 | 1187 | 488 | 591 |
| 0.077 | 0.25 | 1.41 | 0.013 | 0.003 | 0.041 | 0.001 | 0.016 | 0.043 | 0.003 | 0.043 | 0.040 | 0.013 | 0.0006 | 414 | 1186 | 488 | 589 |
| 0.077 | 0.25 | 1.38 | 0.012 | 0.003 | 0.047 | 0.002 | 0.013 | 0.042 | 0.004 | 0.042 | 0.040 | 0.014 | 0.0007 | 404 | 1196 | 489 | 585 |
| 0.077 | 0.25 | 1.40 | 0.012 | 0.003 | 0.040 | 0.002 | 0.012 | 0.042 | 0.004 | 0.043 | 0.040 | 0.014 | 0.0006 | 402 | 1196 | 489 | 587 |
| 0.080 | 0.25 | 1.41 | 0.013 | 0.003 | 0.035 | 0.001 | 0.016 | 0.044 | 0.002 | 0.041 | 0.040 | 0.013 | 0.0005 | 411 | 1186 | 485 | 594 |
| 0.078 | 0.25 | 1.39 | 0.012 | 0.003 | 0.043 | 0.001 | 0.011 | 0.041 | 0.003 | 0.043 | 0.039 | 0.013 | 0.0006 | 399 | 1183 | 488 | 590 |
| 0.076 | 0.25 | 1.40 | 0.012 | 0.003 | 0.043 | 0.001 | 0.016 | 0.043 | 0.003 | 0.043 | 0.040 | 0.014 | 0.0007 | 408 | 1187 | 489 | 587 |
| 0.078 | 0.25 | 1.41 | 0.012 | 0.003 | 0.044 | 0.001 | 0.014 | 0.041 | 0.003 | 0.041 | 0.040 | 0.013 | 0.0006 | 409 | 1179 | 487 | 590 |
| 0.076 | 0.26 | 1.39 | 0.013 | 0.003 | 0.041 | 0.001 | 0.012 | 0.044 | 0.003 | 0.043 | 0.039 | 0.013 | 0.0006 | 401 | 1193 | 489 | 588 |
| 0.077 | 0.25 | 1.40 | 0.012 | 0.003 | 0.042 | 0.001 | 0.013 | 0.043 | 0.003 | 0.042 | 0.040 | 0.013 | 0.0007 | 404 | 1191 | 488 | 590 |
| 0.078 | 0.26 | 1.40 | 0.013 | 0.003 | 0.041 | 0.001 | 0.013 | 0.043 | 0.003 | 0.043 | 0.039 | 0.013 | 0.0007 | 404 | 1190 | 488 | 590 |
| 0.079 | 0.25 | 1.41 | 0.013 | 0.003 | 0.045 | 0.001 | 0.008 | 0.042 | 0.003 | 0.042 | 0.039 | 0.013 | 0.0007 | 408 | 1178 | 486 | 593 |
| 0.077 | 0.25 | 1.39 | 0.013 | 0.003 | 0.041 | 0.001 | 0.017 | 0.040 | 0.004 | 0.043 | 0.040 | 0.013 | 0.0006 | 401 | 1181 | 488 | 589 |
| 0.077 | 0.25 | 1.41 | 0.013 | 0.003 | 0.045 | 0.001 | 0.013 | 0.041 | 0.003 | 0.043 | 0.039 | 0.013 | 0.0006 | 403 | 1183 | 488 | 590 |
| 0.077 | 0.25 | 1.41 | 0.012 | 0.003 | 0.046 | 0.001 | 0.016 | 0.040 | 0.003 | 0.043 | 0.040 | 0.013 | 0.0006 | 417 | 1185 | 488 | 589 |
| 0.078 | 0.25 | 1.40 | 0.013 | 0.003 | 0.040 | 0.002 | 0.015 | 0.043 | 0.003 | 0.044 | 0.040 | 0.013 | 0.0007 | 395 | 1190 | 487 | 589 |
| 0.078 | 0.25 | 1.40 | 0.012 | 0.003 | 0.040 | 0.001 | 0.020 | 0.044 | 0.003 | 0.043 | 0.040 | 0.013 | 0.0006 | 412 | 1178 | 487 | 590 |
| 0.079 | 0.25 | 1.39 | 0.013 | 0.003 | 0.046 | 0.001 | 0.012 | 0.042 | 0.003 | 0.042 | 0.039 | 0.013 | 0.0007 | 403 | 1168 | 486 | 591 |
| 0.077 | 0.25 | 1.40 | 0.013 | 0.003 | 0.039 | 0.001 | 0.013 | 0.043 | 0.004 | 0.042 | 0.040 | 0.013 | 0.0007 | 408 | 1188 | 489 | 588 |
| 0.077 | 0.25 | 1.40 | 0.012 | 0.003 | 0.047 | 0.001 | 0.009 | 0.040 | 0.004 | 0.042 | 0.039 | 0.014 | 0.0007 | 398 | 1195 | 489 | 586 |
| 0.078 | 0.25 | 1.41 | 0.013 | 0.003 | 0.042 | 0.001 | 0.012 | 0.047 | 0.003 | 0.044 | 0.040 | 0.013 | 0.0006 | 402 | 1181 | 487 | 591 |
| 0.078 | 0.25 | 1.41 | 0.013 | 0.003 | 0.042 | 0.002 | 0.011 | 0.041 | 0.003 | 0.043 | 0.039 | 0.013 | 0.0006 | 405 | 1178 | 487 | 590 |
| 0.077 | 0.25 | 1.41 | 0.013 | 0.003 | 0.040 | 0.001 | 0.013 | 0.048 | 0.003 | 0.043 | 0.040 | 0.013 | 0.0007 | 407 | 1181 | 487 | 591 |
| 0.079 | 0.25 | 1.41 | 0.012 | 0.003 | 0.043 | 0.001 | 0.008 | 0.043 | 0.003 | 0.043 | 0.040 | 0.013 | 0.0007 | 402 | 1170 | 487 | 593 |
| 0.076 | 0.25 | 1.39 | 0.012 | 0.003 | 0.048 | 0.002 | 0.014 | 0.042 | 0.003 | 0.041 | 0.039 | 0.014 | 0.0006 | 405 | 1190 | 489 | 585 |
| 0.076 | 0.25 | 1.39 | 0.012 | 0.002 | 0.044 | 0.002 | 0.017 | 0.042 | 0.004 | 0.042 | 0.040 | 0.014 | 0.0006 | 402 | 1191 | 490 | 584 |
| 0.077 | 0.25 | 1.42 | 0.013 | 0.003 | 0.041 | 0.001 | 0.013 | 0.044 | 0.003 | 0.041 | 0.040 | 0.013 | 0.0007 | 411 | 1182 | 488 | 589 |
| 0.077 | 0.25 | 1.39 | 0.012 | 0.003 | 0.044 | 0.001 | 0.028 | 0.045 | 0.003 | 0.043 | 0.040 | 0.013 | 0.0006 | 409 | 1181 | 487 | 588 |
| 0.075 | 0.25 | 1.40 | 0.012 | 0.002 | 0.047 | 0.001 | 0.019 | 0.043 | 0.004 | 0.042 | 0.039 | 0.014 | 0.0007 | 402 | 1200 | 491 | 583 |
| 0.077 | 0.25 | 1.41 | 0.012 | 0.003 | 0.044 | 0.001 | 0.017 | 0.043 | 0.003 | 0.042 | 0.040 | 0.013 | 0.0006 | 400 | 1183 | 488 | 590 |
| 0.078 | 0.26 | 1.40 | 0.013 | 0.003 | 0.041 | 0.001 | 0.014 | 0.044 | 0.003 | 0.042 | 0.039 | 0.013 | 0.0006 | 399 | 1189 | 487 | 592 |
| 0.078 | 0.26 | 1.40 | 0.013 | 0.003 | 0.040 | 0.001 | 0.010 | 0.042 | 0.003 | 0.043 | 0.040 | 0.013 | 0.0006 | 400 | 1178 | 487 | 592 |
| 0.077 | 0.25 | 1.40 | 0.013 | 0.003 | 0.042 | 0.001 | 0.010 | 0.045 | 0.004 | 0.044 | 0.039 | 0.013 | 0.0006 | 411 | 1179 | 488 | 590 |
| 0.076 | 0.25 | 1.40 | 0.012 | 0.003 | 0.043 | 0.001 | 0.019 | 0.045 | 0.004 | 0.041 | 0.040 | 0.013 | 0.0007 | 412 | 1189 | 489 | 587 |
| 0.077 | 0.25 | 1.41 | 0.013 | 0.003 | 0.044 | 0.001 | 0.015 | 0.043 | 0.003 | 0.043 | 0.039 | 0.013 | 0.0006 | 408 | 1178 | 487 | 591 |
| 0.078 | 0.25 | 1.41 | 0.012 | 0.003 | 0.043 | 0.001 | 0.015 | 0.035 | 0.004 | 0.043 | 0.040 | 0.013 | 0.0006 | 408 | 1185 | 487 | 589 |
| 0.078 | 0.26 | 1.41 | 0.013 | 0.003 | 0.043 | 0.001 | 0.013 | 0.046 | 0.003 | 0.042 | 0.040 | 0.013 | 0.0007 | 404 | 1182 | 487 | 591 |
| 0.077 | 0.25 | 1.41 | 0.012 | 0.003 | 0.038 | 0.001 | 0.014 | 0.043 | 0.003 | 0.043 | 0.040 | 0.013 | 0.0006 | 415 | 1183 | 488 | 590 |
| 0.078 | 0.26 | 1.40 | 0.012 | 0.003 | 0.042 | 0.001 | 0.011 | 0.043 | 0.003 | 0.043 | 0.039 | 0.013 | 0.0007 | 402 | 1191 | 488 | 590 |
| 0.077 | 0.25 | 1.40 | 0.013 | 0.003 | 0.041 | 0.001 | 0.014 | 0.042 | 0.003 | 0.043 | 0.040 | 0.013 | 0.0006 | 407 | 1181 | 488 | 590 |
| 0.079 | 0.25 | 1.42 | 0.013 | 0.003 | 0.038 | 0.001 | 0.012 | 0.045 | 0.003 | 0.045 | 0.040 | 0.013 | 0.0006 | 410 | 1172 | 486 | 596 |
| 0.077 | 0.25 | 1.38 | 0.012 | 0.003 | 0.046 | 0.001 | 0.016 | 0.043 | 0.004 | 0.043 | 0.040 | 0.014 | 0.0007 | 398 | 1194 | 490 | 584 |
| 0.076 | 0.25 | 1.39 | 0.012 | 0.002 | 0.047 | 0.002 | 0.010 | 0.043 | 0.004 | 0.041 | 0.040 | 0.014 | 0.0006 | 397 | 1193 | 490 | 585 |
| 0.079 | 0.25 | 1.42 | 0.013 | 0.003 | 0.034 | 0.001 | 0.015 | 0.042 | 0.003 | 0.045 | 0.039 | 0.013 | 0.0006 | 402 | 1175 | 486 | 594 |
| 0.077 | 0.25 | 1.41 | 0.013 | 0.003 | 0.042 | 0.001 | 0.014 | 0.044 | 0.003 | 0.042 | 0.039 | 0.013 | 0.0007 | 402 | 1187 | 488 | 590 |
| 0.076 | 0.25 | 1.40 | 0.012 | 0.003 | 0.049 | 0.001 | 0.012 | 0.043 | 0.003 | 0.042 | 0.040 | 0.014 | 0.0006 | 383 | 1191 | 489 | 585 |
| 0.080 | 0.26 | 1.42 | 0.013 | 0.003 | 0.038 | 0.001 | 0.017 | 0.041 | 0.002 | 0.045 | 0.040 | 0.013 | 0.0006 | 413 | 1170 | 485 | 598 |
| 0.078 | 0.26 | 1.42 | 0.013 | 0.003 | 0.038 | 0.001 | 0.009 | 0.038 | 0.003 | 0.044 | 0.040 | 0.013 | 0.0006 | 400 | 1185 | 487 | 594 |
| 0.078 | 0.26 | 1.42 | 0.013 | 0.003 | 0.042 | 0.001 | 0.011 | 0.046 | 0.003 | 0.043 | 0.040 | 0.013 | 0.0007 | 401 | 1181 | 487 | 593 |
| 0.077 | 0.25 | 1.39 | 0.012 | 0.002 | 0.042 | 0.002 | 0.016 | 0.041 | 0.003 | 0.041 | 0.039 | 0.013 | 0.0006 | 403 | 1194 | 489 | 586 |
| 0.076 | 0.25 | 1.40 | 0.012 | 0.003 | 0.042 | 0.001 | 0.011 | 0.041 | 0.003 | 0.043 | 0.039 | 0.013 | 0.0006 | 404 | 1190 | 489 | 589 |
| 0.077 | 0.25 | 1.39 | 0.012 | 0.003 | 0.043 | 0.001 | 0.016 | 0.044 | 0.003 | 0.044 | 0.039 | 0.014 | 0.0008 | 403 | 1190 | 488 | 587 |
| 0.078 | 0.25 | 1.41 | 0.013 | 0.003 | 0.040 | 0.001 | 0.010 | 0.045 | 0.003 | 0.043 | 0.041 | 0.013 | 0.0006 | 408 | 1166 | 486 | 593 |
| 0.076 | 0.25 | 1.39 | 0.012 | 0.002 | 0.043 | 0.002 | 0.018 | 0.044 | 0.004 | 0.042 | 0.039 | 0.014 | 0.0007 | 399 | 1198 | 490 | 583 |
| 0.078 | 0.25 | 1.41 | 0.013 | 0.003 | 0.046 | 0.001 | 0.016 | 0.042 | 0.003 | 0.042 | 0.040 | 0.013 | 0.0006 | 403 | 1187 | 487 | 590 |
| 0.078 | 0.25 | 1.39 | 0.013 | 0.003 | 0.040 | 0.001 | 0.009 | 0.045 | 0.003 | 0.044 | 0.040 | 0.013 | 0.0006 | 401 | 1194 | 488 | 590 |
| 0.078 | 0.25 | 1.41 | 0.013 | 0.003 | 0.045 | 0.001 | 0.011 | 0.044 | 0.003 | 0.043 | 0.040 | 0.014 | 0.0006 | 405 | 1188 | 488 | 590 |
| 0.077 | 0.25 | 1.43 | 0.012 | 0.003 | 0.040 | 0.001 | 0.015 | 0.046 | 0.003 | 0.044 | 0.039 | 0.013 | 0.0006 | 403 | 1190 | 488 | 592 |
| 0.078 | 0.25 | 1.40 | 0.013 | 0.003 | 0.046 | 0.001 | 0.016 | 0.043 | 0.003 | 0.043 | 0.040 | 0.013 | 0.0006 | 403 | 1176 | 487 | 590 |
| 0.077 | 0.25 | 1.42 | 0.013 | 0.003 | 0.043 | 0.001 | 0.015 | 0.044 | 0.003 | 0.043 | 0.040 | 0.013 | 0.0006 | 407 | 1185 | 488 | 589 |
| 0.078 | 0.25 | 1.41 | 0.013 | 0.003 | 0.043 | 0.001 | 0.022 | 0.042 | 0.003 | 0.044 | 0.040 | 0.013 | 0.0007 | 407 | 1183 | 487 | 590 |
| 0.078 | 0.25 | 1.42 | 0.013 | 0.003 | 0.040 | 0.001 | 0.011 | 0.042 | 0.003 | 0.043 | 0.040 | 0.013 | 0.0006 | 409 | 1188 | 487 | 592 |
| 0.075 | 0.25 | 1.40 | 0.012 | 0.003 | 0.047 | 0.001 | 0.017 | 0.047 | 0.004 | 0.042 | 0.040 | 0.013 | 0.0007 | 402 | 1197 | 490 | 585 |
| 0.076 | 0.25 | 1.39 | 0.013 | 0.002 | 0.041 | 0.001 | 0.016 | 0.044 | 0.003 | 0.042 | 0.039 | 0.013 | 0.0007 | 405 | 1190 | 489 | 586 |
| 0.077 | 0.25 | 1.41 | 0.012 | 0.003 | 0.042 | 0.001 | 0.013 | 0.043 | 0.003 | 0.044 | 0.040 | 0.013 | 0.0007 | 404 | 1187 | 488 | 590 |
| 0.077 | 0.25 | 1.41 | 0.013 | 0.003 | 0.040 | 0.001 | 0.010 | 0.043 | 0.004 | 0.043 | 0.040 | 0.013 | 0.0006 | 405 | 1181 | 488 | 590 |
| 0.078 | 0.25 | 1.41 | 0.013 | 0.003 | 0.044 | 0.001 | 0.014 | 0.045 | 0.003 | 0.044 | 0.040 | 0.013 | 0.0006 | 397 | 1184 | 488 | 591 |
| 0.077 | 0.25 | 1.41 | 0.013 | 0.003 | 0.039 | 0.001 | 0.010 | 0.044 | 0.003 | 0.043 | 0.040 | 0.013 | 0.0006 | 407 | 1176 | 487 | 591 |
| 0.078 | 0.26 | 1.43 | 0.013 | 0.003 | 0.038 | 0.001 | 0.018 | 0.043 | 0.003 | 0.045 | 0.040 | 0.013 | 0.0006 | 407 | 1175 | 486 | 594 |
| 0.077 | 0.26 | 1.40 | 0.013 | 0.003 | 0.043 | 0.001 | 0.014 | 0.043 | 0.003 | 0.043 | 0.041 | 0.013 | 0.0006 | 398 | 1180 | 488 | 590 |
| 0.078 | 0.26 | 1.42 | 0.013 | 0.003 | 0.038 | 0.001 | 0.013 | 0.048 | 0.003 | 0.045 | 0.039 | 0.013 | 0.0006 | 407 | 1181 | 486 | 594 |
| 0.077 | 0.25 | 1.40 | 0.012 | 0.003 | 0.043 | 0.001 | 0.014 | 0.043 | 0.004 | 0.044 | 0.038 | 0.014 | 0.0007 | 408 | 1171 | 487 | 589 |
| 0.079 | 0.25 | 1.41 | 0.013 | 0.003 | 0.044 | 0.001 | 0.015 | 0.043 | 0.003 | 0.043 | 0.040 | 0.013 | 0.0007 | 414 | 1171 | 486 | 593 |
| 0.077 | 0.25 | 1.39 | 0.013 | 0.003 | 0.042 | 0.002 | 0.012 | 0.043 | 0.003 | 0.043 | 0.039 | 0.014 | 0.0007 | 401 | 1197 | 489 | 587 |
| 0.077 | 0.25 | 1.39 | 0.012 | 0.003 | 0.048 | 0.001 | 0.015 | 0.043 | 0.004 | 0.040 | 0.040 | 0.013 | 0.0007 | 408 | 1197 | 489 | 585 |
| 0.079 | 0.25 | 1.40 | 0.013 | 0.003 | 0.043 | 0.001 | 0.013 | 0.045 | 0.003 | 0.043 | 0.039 | 0.013 | 0.0006 | 399 | 1173 | 486 | 592 |
| 0.076 | 0.25 | 1.38 | 0.012 | 0.002 | 0.045 | 0.001 | 0.019 | 0.046 | 0.004 | 0.041 | 0.039 | 0.014 | 0.0007 | 401 | 1200 | 489 | 584 |
| 0.076 | 0.25 | 1.40 | 0.013 | 0.003 | 0.043 | 0.002 | 0.010 | 0.045 | 0.004 | 0.043 | 0.039 | 0.013 | 0.0007 | 403 | 1191 | 489 | 587 |
| 0.078 | 0.25 | 1.38 | 0.013 | 0.003 | 0.047 | 0.001 | 0.015 | 0.044 | 0.003 | 0.044 | 0.039 | 0.014 | 0.0006 | 399 | 1190 | 487 | 588 |
| 0.077 | 0.25 | 1.40 | 0.012 | 0.003 | 0.046 | 0.002 | 0.014 | 0.045 | 0.003 | 0.043 | 0.039 | 0.013 | 0.0007 | 404 | 1185 | 488 | 588 |
| 0.077 | 0.25 | 1.41 | 0.012 | 0.003 | 0.040 | 0.001 | 0.014 | 0.043 | 0.003 | 0.043 | 0.039 | 0.013 | 0.0007 | 400 | 1183 | 489 | 589 |
| 0.077 | 0.25 | 1.40 | 0.013 | 0.003 | 0.045 | 0.001 | 0.014 | 0.044 | 0.003 | 0.043 | 0.040 | 0.013 | 0.0005 | 405 | 1188 | 489 | 588 |
| 0.077 | 0.25 | 1.39 | 0.013 | 0.003 | 0.046 | 0.002 | 0.014 | 0.043 | 0.004 | 0.043 | 0.039 | 0.013 | 0.0007 | 394 | 1195 | 489 | 586 |
| 0.080 | 0.25 | 1.41 | 0.013 | 0.003 | 0.042 | 0.001 | 0.014 | 0.047 | 0.003 | 0.042 | 0.039 | 0.013 | 0.0004 | 404 | 1182 | 485 | 594 |
| 0.078 | 0.25 | 1.42 | 0.013 | 0.003 | 0.038 | 0.001 | 0.013 | 0.045 | 0.002 | 0.044 | 0.040 | 0.013 | 0.0006 | 396 | 1173 | 487 | 594 |
| 0.077 | 0.25 | 1.40 | 0.012 | 0.003 | 0.040 | 0.001 | 0.011 | 0.046 | 0.003 | 0.043 | 0.040 | 0.014 | 0.0007 | 407 | 1188 | 489 | 588 |
| 0.077 | 0.25 | 1.40 | 0.012 | 0.003 | 0.042 | 0.002 | 0.015 | 0.044 | 0.003 | 0.042 | 0.040 | 0.013 | 0.0007 | 402 | 1174 | 488 | 588 |
| 0.077 | 0.25 | 1.41 | 0.012 | 0.003 | 0.043 | 0.001 | 0.016 | 0.044 | 0.003 | 0.043 | 0.040 | 0.013 | 0.0006 | 402 | 1189 | 488 | 589 |
| 0.077 | 0.25 | 1.41 | 0.013 | 0.003 | 0.040 | 0.001 | 0.012 | 0.043 | 0.003 | 0.041 | 0.040 | 0.013 | 0.0006 | 402 | 1183 | 489 | 589 |
| 0.077 | 0.25 | 1.40 | 0.013 | 0.003 | 0.044 | 0.002 | 0.016 | 0.044 | 0.003 | 0.044 | 0.040 | 0.013 | 0.0006 | 405 | 1191 | 488 | 588 |
| 0.078 | 0.26 | 1.40 | 0.013 | 0.003 | 0.044 | 0.002 | 0.015 | 0.043 | 0.003 | 0.043 | 0.039 | 0.013 | 0.0006 | 399 | 1172 | 487 | 589 |
| 0.078 | 0.25 | 1.40 | 0.012 | 0.003 | 0.045 | 0.001 | 0.018 | 0.046 | 0.004 | 0.041 | 0.041 | 0.013 | 0.0007 | 416 | 1185 | 487 | 587 |
| 0.077 | 0.25 | 1.38 | 0.012 | 0.003 | 0.045 | 0.001 | 0.014 | 0.046 | 0.004 | 0.042 | 0.039 | 0.013 | 0.0006 | 403 | 1185 | 489 | 587 |
| 0.077 | 0.25 | 1.41 | 0.013 | 0.003 | 0.041 | 0.001 | 0.010 | 0.043 | 0.003 | 0.044 | 0.040 | 0.013 | 0.0006 | 407 | 1176 | 488 | 591 |
| 0.079 | 0.25 | 1.41 | 0.013 | 0.003 | 0.038 | 0.001 | 0.008 | 0.038 | 0.002 | 0.044 | 0.040 | 0.013 | 0.0006 | 395 | 1176 | 487 | 594 |
| 0.077 | 0.25 | 1.40 | 0.012 | 0.003 | 0.047 | 0.001 | 0.013 | 0.045 | 0.004 | 0.042 | 0.039 | 0.013 | 0.0007 | 403 | 1190 | 488 | 587 |
| 0.077 | 0.25 | 1.39 | 0.012 | 0.003 | 0.042 | 0.001 | 0.010 | 0.043 | 0.003 | 0.042 | 0.040 | 0.013 | 0.0006 | 400 | 1181 | 489 | 589 |
| 0.078 | 0.25 | 1.38 | 0.012 | 0.003 | 0.046 | 0.001 | 0.012 | 0.046 | 0.003 | 0.041 | 0.040 | 0.014 | 0.0006 | 411 | 1184 | 488 | 587 |
| 0.077 | 0.25 | 1.40 | 0.013 | 0.003 | 0.045 | 0.002 | 0.013 | 0.042 | 0.003 | 0.043 | 0.040 | 0.013 | 0.0007 | 408 | 1186 | 488 | 588 |
| 0.078 | 0.25 | 1.41 | 0.013 | 0.003 | 0.040 | 0.001 | 0.010 | 0.039 | 0.003 | 0.043 | 0.040 | 0.013 | 0.0006 | 404 | 1182 | 488 | 591 |
| 0.076 | 0.25 | 1.40 | 0.012 | 0.002 | 0.048 | 0.002 | 0.022 | 0.044 | 0.004 | 0.042 | 0.039 | 0.013 | 0.0006 | 401 | 1186 | 489 | 584 |
| 0.079 | 0.25 | 1.41 | 0.013 | 0.003 | 0.043 | 0.001 | 0.015 | 0.043 | 0.003 | 0.042 | 0.040 | 0.013 | 0.0006 | 399 | 1185 | 487 | 592 |
| 0.077 | 0.25 | 1.40 | 0.013 | 0.003 | 0.044 | 0.001 | 0.010 | 0.048 | 0.003 | 0.044 | 0.040 | 0.013 | 0.0006 | 417 | 1180 | 487 | 591 |
| 0.078 | 0.25 | 1.40 | 0.013 | 0.003 | 0.044 | 0.001 | 0.011 | 0.043 | 0.003 | 0.043 | 0.040 | 0.013 | 0.0006 | 401 | 1186 | 488 | 590 |
| 0.078 | 0.25 | 1.42 | 0.013 | 0.003 | 0.037 | 0.001 | 0.011 | 0.045 | 0.003 | 0.043 | 0.038 | 0.013 | 0.0006 | 405 | 1178 | 486 | 594 |
| 0.077 | 0.25 | 1.40 | 0.012 | 0.002 | 0.042 | 0.002 | 0.024 | 0.047 | 0.004 | 0.043 | 0.040 | 0.014 | 0.0007 | 403 | 1191 | 489 | 586 |
| 0.077 | 0.25 | 1.41 | 0.012 | 0.003 | 0.039 | 0.001 | 0.010 | 0.039 | 0.003 | 0.044 | 0.040 | 0.014 | 0.0007 | 395 | 1193 | 489 | 589 |
| 0.077 | 0.25 | 1.40 | 0.013 | 0.003 | 0.043 | 0.001 | 0.013 | 0.043 | 0.004 | 0.042 | 0.039 | 0.013 | 0.0006 | 405 | 1186 | 488 | 588 |
| 0.077 | 0.25 | 1.41 | 0.012 | 0.003 | 0.042 | 0.001 | 0.013 | 0.036 | 0.003 | 0.044 | 0.040 | 0.013 | 0.0007 | 404 | 1183 | 488 | 590 |
| 0.077 | 0.25 | 1.39 | 0.013 | 0.003 | 0.046 | 0.001 | 0.014 | 0.045 | 0.004 | 0.042 | 0.039 | 0.013 | 0.0007 | 406 | 1191 | 489 | 587 |
| 0.077 | 0.25 | 1.39 | 0.013 | 0.002 | 0.042 | 0.002 | 0.020 | 0.043 | 0.003 | 0.043 | 0.040 | 0.013 | 0.0006 | 403 | 1180 | 488 | 587 |
| 0.078 | 0.25 | 1.41 | 0.012 | 0.003 | 0.040 | 0.001 | 0.017 | 0.044 | 0.003 | 0.041 | 0.039 | 0.014 | 0.0006 | 402 | 1182 | 488 | 588 |
| 0.079 | 0.26 | 1.42 | 0.013 | 0.003 | 0.035 | 0.001 | 0.009 | 0.041 | 0.003 | 0.046 | 0.039 | 0.013 | 0.0006 | 411 | 1171 | 486 | 597 |
| 0.076 | 0.25 | 1.39 | 0.012 | 0.003 | 0.045 | 0.001 | 0.019 | 0.047 | 0.003 | 0.043 | 0.040 | 0.014 | 0.0007 | 401 | 1194 | 489 | 587 |
| 0.078 | 0.25 | 1.42 | 0.013 | 0.003 | 0.039 | 0.001 | 0.012 | 0.045 | 0.003 | 0.045 | 0.040 | 0.013 | 0.0006 | 401 | 1175 | 487 | 593 |
| 0.077 | 0.25 | 1.40 | 0.013 | 0.003 | 0.041 | 0.001 | 0.014 | 0.046 | 0.003 | 0.043 | 0.040 | 0.013 | 0.0007 | 404 | 1186 | 489 | 589 |
| 0.077 | 0.25 | 1.39 | 0.013 | 0.003 | 0.042 | 0.001 | 0.012 | 0.044 | 0.003 | 0.042 | 0.040 | 0.013 | 0.0006 | 401 | 1175 | 488 | 589 |
| 0.078 | 0.25 | 1.42 | 0.013 | 0.003 | 0.044 | 0.001 | 0.011 | 0.042 | 0.003 | 0.043 | 0.040 | 0.013 | 0.0006 | 403 | 1179 | 487 | 593 |
| 0.078 | 0.25 | 1.40 | 0.013 | 0.002 | 0.043 | 0.001 | 0.015 | 0.044 | 0.003 | 0.043 | 0.040 | 0.013 | 0.0006 | 402 | 1184 | 487 | 589 |
| 0.078 | 0.25 | 1.41 | 0.013 | 0.003 | 0.040 | 0.001 | 0.011 | 0.041 | 0.003 | 0.044 | 0.040 | 0.013 | 0.0006 | 398 | 1178 | 487 | 592 |
| 0.077 | 0.25 | 1.41 | 0.013 | 0.003 | 0.045 | 0.001 | 0.020 | 0.044 | 0.003 | 0.043 | 0.040 | 0.013 | 0.0005 | 402 | 1182 | 488 | 589 |
| 0.080 | 0.25 | 1.41 | 0.012 | 0.003 | 0.045 | 0.001 | 0.013 | 0.043 | 0.003 | 0.044 | 0.040 | 0.013 | 0.0005 | 414 | 1167 | 485 | 594 |
| 0.077 | 0.25 | 1.41 | 0.013 | 0.003 | 0.043 | 0.001 | 0.016 | 0.041 | 0.003 | 0.044 | 0.040 | 0.013 | 0.0006 | 399 | 1186 | 488 | 590 |
| 0.076 | 0.25 | 1.40 | 0.012 | 0.003 | 0.040 | 0.001 | 0.012 | 0.046 | 0.003 | 0.043 | 0.040 | 0.014 | 0.0007 | 408 | 1183 | 489 | 587 |
| 0.078 | 0.25 | 1.41 | 0.013 | 0.003 | 0.040 | 0.001 | 0.010 | 0.042 | 0.003 | 0.042 | 0.039 | 0.013 | 0.0005 | 404 | 1176 | 487 | 592 |
| 0.077 | 0.25 | 1.41 | 0.012 | 0.003 | 0.045 | 0.001 | 0.019 | 0.045 | 0.003 | 0.043 | 0.040 | 0.013 | 0.0006 | 398 | 1191 | 488 | 589 |
| 0.078 | 0.25 | 1.40 | 0.013 | 0.003 | 0.042 | 0.001 | 0.019 | 0.040 | 0.003 | 0.043 | 0.040 | 0.013 | 0.0006 | 401 | 1186 | 488 | 590 |
| 0.077 | 0.26 | 1.42 | 0.013 | 0.003 | 0.040 | 0.001 | 0.016 | 0.041 | 0.003 | 0.043 | 0.040 | 0.013 | 0.0005 | 400 | 1192 | 488 | 590 |
| 0.078 | 0.25 | 1.41 | 0.013 | 0.003 | 0.042 | 0.001 | 0.010 | 0.045 | 0.003 | 0.040 | 0.040 | 0.013 | 0.0006 | 408 | 1177 | 487 | 591 |
| 0.077 | 0.25 | 1.40 | 0.013 | 0.003 | 0.044 | 0.001 | 0.018 | 0.044 | 0.003 | 0.043 | 0.040 | 0.013 | 0.0007 | 405 | 1183 | 488 | 589 |
| 0.077 | 0.25 | 1.40 | 0.012 | 0.003 | 0.042 | 0.001 | 0.009 | 0.043 | 0.004 | 0.041 | 0.040 | 0.014 | 0.0007 | 407 | 1186 | 489 | 587 |
| 0.078 | 0.25 | 1.41 | 0.012 | 0.003 | 0.042 | 0.001 | 0.009 | 0.047 | 0.003 | 0.044 | 0.039 | 0.013 | 0.0007 | 408 | 1179 | 487 | 591 |
| 0.077 | 0.25 | 1.41 | 0.012 | 0.003 | 0.039 | 0.001 | 0.013 | 0.041 | 0.003 | 0.043 | 0.040 | 0.013 | 0.0006 | 410 | 1185 | 488 | 591 |
| 0.078 | 0.25 | 1.37 | 0.012 | 0.002 | 0.043 | 0.001 | 0.025 | 0.048 | 0.003 | 0.040 | 0.040 | 0.014 | 0.0007 | 404 | 1194 | 487 | 585 |
| 0.077 | 0.25 | 1.41 | 0.013 | 0.003 | 0.042 | 0.001 | 0.012 | 0.044 | 0.004 | 0.043 | 0.040 | 0.014 | 0.0006 | 402 | 1188 | 489 | 589 |
| 0.078 | 0.25 | 1.41 | 0.013 | 0.003 | 0.036 | 0.001 | 0.011 | 0.042 | 0.003 | 0.045 | 0.041 | 0.013 | 0.0006 | 410 | 1173 | 487 | 594 |
| 0.078 | 0.26 | 1.42 | 0.013 | 0.003 | 0.039 | 0.001 | 0.011 | 0.043 | 0.003 | 0.043 | 0.039 | 0.013 | 0.0006 | 403 | 1183 | 486 | 594 |
| 0.077 | 0.25 | 1.41 | 0.013 | 0.003 | 0.044 | 0.001 | 0.013 | 0.041 | 0.003 | 0.043 | 0.039 | 0.014 | 0.0007 | 404 | 1181 | 488 | 589 |
| 0.078 | 0.25 | 1.41 | 0.013 | 0.003 | 0.037 | 0.001 | 0.010 | 0.044 | 0.003 | 0.044 | 0.040 | 0.013 | 0.0005 | 406 | 1176 | 487 | 594 |
| 0.078 | 0.25 | 1.39 | 0.012 | 0.003 | 0.045 | 0.001 | 0.012 | 0.043 | 0.003 | 0.046 | 0.040 | 0.013 | 0.0006 | 425 | 1172 | 486 | 592 |
| 0.078 | 0.26 | 1.42 | 0.013 | 0.003 | 0.045 | 0.001 | 0.018 | 0.038 | 0.003 | 0.044 | 0.040 | 0.013 | 0.0006 | 400 | 1177 | 487 | 591 |
| 0.078 | 0.26 | 1.42 | 0.013 | 0.003 | 0.036 | 0.001 | 0.009 | 0.046 | 0.002 | 0.045 | 0.039 | 0.013 | 0.0006 | 409 | 1185 | 486 | 596 |
| 0.076 | 0.25 | 1.41 | 0.013 | 0.003 | 0.045 | 0.002 | 0.018 | 0.043 | 0.003 | 0.042 | 0.039 | 0.014 | 0.0007 | 409 | 1187 | 489 | 587 |
| 0.076 | 0.25 | 1.40 | 0.012 | 0.003 | 0.043 | 0.001 | 0.018 | 0.042 | 0.004 | 0.043 | 0.040 | 0.013 | 0.0007 | 409 | 1192 | 489 | 586 |
| 0.078 | 0.25 | 1.42 | 0.013 | 0.003 | 0.042 | 0.001 | 0.011 | 0.041 | 0.003 | 0.042 | 0.040 | 0.013 | 0.0006 | 399 | 1183 | 488 | 590 |
| 0.078 | 0.25 | 1.40 | 0.012 | 0.003 | 0.038 | 0.001 | 0.014 | 0.044 | 0.003 | 0.042 | 0.039 | 0.013 | 0.0006 | 405 | 1177 | 488 | 590 |
| 0.076 | 0.25 | 1.40 | 0.012 | 0.003 | 0.042 | 0.001 | 0.011 | 0.046 | 0.004 | 0.041 | 0.039 | 0.014 | 0.0007 | 406 | 1194 | 489 | 586 |
| 0.079 | 0.26 | 1.39 | 0.013 | 0.003 | 0.036 | 0.002 | 0.015 | 0.043 | 0.003 | 0.043 | 0.040 | 0.013 | 0.0006 | 405 | 1188 | 487 | 590 |
| 0.076 | 0.25 | 1.41 | 0.013 | 0.003 | 0.043 | 0.001 | 0.015 | 0.045 | 0.003 | 0.043 | 0.040 | 0.013 | 0.0006 | 398 | 1188 | 489 | 588 |
| 0.079 | 0.25 | 1.42 | 0.012 | 0.003 | 0.031 | 0.001 | 0.011 | 0.043 | 0.002 | 0.045 | 0.039 | 0.013 | 0.0006 | 392 | 1181 | 487 | 596 |
| 0.077 | 0.25 | 1.39 | 0.013 | 0.003 | 0.042 | 0.002 | 0.012 | 0.041 | 0.003 | 0.042 | 0.040 | 0.013 | 0.0006 | 401 | 1176 | 488 | 589 |
| 0.079 | 0.26 | 1.42 | 0.013 | 0.003 | 0.038 | 0.001 | 0.007 | 0.042 | 0.002 | 0.045 | 0.040 | 0.013 | 0.0005 | 400 | 1181 | 487 | 597 |
| 0.078 | 0.26 | 1.41 | 0.013 | 0.003 | 0.037 | 0.001 | 0.016 | 0.042 | 0.003 | 0.043 | 0.040 | 0.013 | 0.0006 | 410 | 1181 | 487 | 593 |
| 0.078 | 0.25 | 1.41 | 0.013 | 0.003 | 0.041 | 0.001 | 0.011 | 0.042 | 0.003 | 0.045 | 0.039 | 0.013 | 0.0006 | 404 | 1181 | 487 | 592 |
| 0.078 | 0.25 | 1.40 | 0.013 | 0.003 | 0.042 | 0.001 | 0.015 | 0.046 | 0.003 | 0.044 | 0.040 | 0.013 | 0.0006 | 399 | 1180 | 488 | 591 |
| 0.078 | 0.25 | 1.39 | 0.013 | 0.003 | 0.043 | 0.001 | 0.008 | 0.042 | 0.003 | 0.044 | 0.039 | 0.013 | 0.0007 | 410 | 1178 | 487 | 591 |
| 0.078 | 0.25 | 1.39 | 0.012 | 0.002 | 0.047 | 0.001 | 0.015 | 0.041 | 0.004 | 0.041 | 0.040 | 0.014 | 0.0006 | 404 | 1187 | 488 | 586 |
| 0.076 | 0.25 | 1.40 | 0.012 | 0.003 | 0.042 | 0.002 | 0.019 | 0.042 | 0.003 | 0.043 | 0.040 | 0.013 | 0.0007 | 398 | 1185 | 489 | 587 |
| 0.076 | 0.25 | 1.40 | 0.012 | 0.003 | 0.049 | 0.001 | 0.013 | 0.043 | 0.003 | 0.039 | 0.039 | 0.013 | 0.0007 | 404 | 1201 | 489 | 585 |
| 0.077 | 0.25 | 1.40 | 0.012 | 0.003 | 0.046 | 0.001 | 0.017 | 0.043 | 0.004 | 0.043 | 0.040 | 0.013 | 0.0006 | 415 | 1189 | 489 | 588 |
| 0.077 | 0.25 | 1.40 | 0.013 | 0.003 | 0.042 | 0.001 | 0.016 | 0.046 | 0.003 | 0.042 | 0.040 | 0.013 | 0.0006 | 412 | 1186 | 488 | 589 |
| 0.079 | 0.25 | 1.40 | 0.013 | 0.003 | 0.038 | 0.001 | 0.016 | 0.042 | 0.003 | 0.044 | 0.040 | 0.013 | 0.0007 | 405 | 1184 | 487 | 593 |
| 0.076 | 0.25 | 1.39 | 0.012 | 0.003 | 0.043 | 0.002 | 0.014 | 0.042 | 0.004 | 0.042 | 0.040 | 0.013 | 0.0006 | 398 | 1186 | 489 | 587 |
| 0.078 | 0.25 | 1.41 | 0.013 | 0.003 | 0.041 | 0.001 | 0.010 | 0.042 | 0.003 | 0.044 | 0.040 | 0.013 | 0.0006 | 396 | 1180 | 487 | 592 |
| 0.077 | 0.25 | 1.38 | 0.012 | 0.003 | 0.043 | 0.002 | 0.012 | 0.043 | 0.003 | 0.043 | 0.039 | 0.014 | 0.0006 | 397 | 1197 | 489 | 586 |
| 0.076 | 0.25 | 1.41 | 0.013 | 0.003 | 0.044 | 0.001 | 0.017 | 0.046 | 0.003 | 0.042 | 0.041 | 0.013 | 0.0006 | 407 | 1184 | 489 | 587 |
| 0.076 | 0.25 | 1.40 | 0.012 | 0.003 | 0.042 | 0.001 | 0.016 | 0.046 | 0.004 | 0.043 | 0.040 | 0.013 | 0.0006 | 404 | 1191 | 489 | 587 |
| 0.078 | 0.25 | 1.39 | 0.013 | 0.003 | 0.043 | 0.001 | 0.015 | 0.044 | 0.004 | 0.042 | 0.039 | 0.014 | 0.0007 | 400 | 1191 | 488 | 587 |
| 0.077 | 0.25 | 1.41 | 0.013 | 0.003 | 0.041 | 0.001 | 0.013 | 0.043 | 0.003 | 0.044 | 0.040 | 0.013 | 0.0006 | 400 | 1196 | 489 | 590 |
| 0.078 | 0.26 | 1.40 | 0.013 | 0.003 | 0.039 | 0.001 | 0.011 | 0.044 | 0.003 | 0.045 | 0.039 | 0.014 | 0.0007 | 397 | 1182 | 487 | 590 |
| 0.078 | 0.25 | 1.41 | 0.013 | 0.003 | 0.035 | 0.001 | 0.012 | 0.043 | 0.003 | 0.044 | 0.040 | 0.013 | 0.0006 | 409 | 1184 | 487 | 593 |
| 0.077 | 0.25 | 1.41 | 0.012 | 0.003 | 0.042 | 0.001 | 0.014 | 0.044 | 0.003 | 0.044 | 0.039 | 0.013 | 0.0006 | 410 | 1182 | 487 | 591 |
| 0.078 | 0.25 | 1.42 | 0.013 | 0.003 | 0.043 | 0.001 | 0.013 | 0.045 | 0.003 | 0.044 | 0.040 | 0.013 | 0.0007 | 409 | 1185 | 487 | 591 |
| 0.078 | 0.25 | 1.40 | 0.013 | 0.003 | 0.042 | 0.001 | 0.011 | 0.042 | 0.003 | 0.044 | 0.040 | 0.013 | 0.0006 | 400 | 1174 | 487 | 592 |
| 0.077 | 0.25 | 1.41 | 0.013 | 0.003 | 0.039 | 0.001 | 0.016 | 0.044 | 0.003 | 0.044 | 0.040 | 0.013 | 0.0007 | 413 | 1184 | 488 | 590 |
| 0.077 | 0.25 | 1.41 | 0.013 | 0.003 | 0.043 | 0.001 | 0.015 | 0.043 | 0.003 | 0.044 | 0.039 | 0.013 | 0.0006 | 410 | 1184 | 488 | 590 |
| 0.078 | 0.25 | 1.42 | 0.013 | 0.003 | 0.039 | 0.001 | 0.009 | 0.043 | 0.002 | 0.043 | 0.040 | 0.013 | 0.0006 | 396 | 1186 | 488 | 594 |
| 0.076 | 0.25 | 1.40 | 0.012 | 0.003 | 0.045 | 0.002 | 0.017 | 0.040 | 0.003 | 0.043 | 0.039 | 0.014 | 0.0007 | 401 | 1188 | 489 | 587 |
| 0.077 | 0.26 | 1.40 | 0.013 | 0.003 | 0.041 | 0.001 | 0.018 | 0.046 | 0.003 | 0.043 | 0.040 | 0.014 | 0.0006 | 401 | 1188 | 488 | 589 |
| 0.077 | 0.25 | 1.39 | 0.012 | 0.003 | 0.044 | 0.001 | 0.017 | 0.044 | 0.004 | 0.043 | 0.039 | 0.013 | 0.0006 | 413 | 1185 | 488 | 588 |
| 0.077 | 0.26 | 1.39 | 0.012 | 0.003 | 0.047 | 0.001 | 0.015 | 0.045 | 0.003 | 0.043 | 0.040 | 0.013 | 0.0007 | 400 | 1179 | 488 | 588 |
| 0.078 | 0.25 | 1.42 | 0.013 | 0.003 | 0.043 | 0.001 | 0.015 | 0.044 | 0.003 | 0.043 | 0.040 | 0.013 | 0.0006 | 409 | 1178 | 487 | 592 |
| 0.079 | 0.26 | 1.41 | 0.013 | 0.003 | 0.043 | 0.001 | 0.016 | 0.044 | 0.003 | 0.044 | 0.039 | 0.013 | 0.0005 | 425 | 1183 | 485 | 594 |
| 0.076 | 0.25 | 1.39 | 0.012 | 0.003 | 0.048 | 0.001 | 0.016 | 0.044 | 0.003 | 0.043 | 0.039 | 0.013 | 0.0006 | 399 | 1188 | 489 | 587 |
| 0.076 | 0.25 | 1.40 | 0.012 | 0.003 | 0.045 | 0.001 | 0.016 | 0.042 | 0.004 | 0.042 | 0.040 | 0.013 | 0.0007 | 399 | 1182 | 489 | 586 |
| 0.077 | 0.25 | 1.41 | 0.012 | 0.003 | 0.041 | 0.001 | 0.010 | 0.042 | 0.003 | 0.044 | 0.039 | 0.013 | 0.0006 | 404 | 1189 | 488 | 590 |
| 0.077 | 0.25 | 1.41 | 0.012 | 0.003 | 0.043 | 0.001 | 0.011 | 0.043 | 0.003 | 0.044 | 0.040 | 0.013 | 0.0006 | 401 | 1181 | 488 | 591 |
| 0.079 | 0.25 | 1.40 | 0.013 | 0.003 | 0.044 | 0.001 | 0.010 | 0.042 | 0.003 | 0.042 | 0.039 | 0.013 | 0.0006 | 401 | 1187 | 487 | 590 |
| 0.078 | 0.25 | 1.41 | 0.013 | 0.003 | 0.037 | 0.001 | 0.014 | 0.041 | 0.003 | 0.042 | 0.040 | 0.013 | 0.0006 | 408 | 1173 | 486 | 593 |
| 0.077 | 0.25 | 1.41 | 0.012 | 0.003 | 0.043 | 0.001 | 0.012 | 0.044 | 0.004 | 0.043 | 0.040 | 0.013 | 0.0007 | 406 | 1191 | 489 | 587 |
| 0.077 | 0.25 | 1.39 | 0.012 | 0.003 | 0.042 | 0.001 | 0.014 | 0.046 | 0.004 | 0.041 | 0.039 | 0.014 | 0.0007 | 398 | 1189 | 489 | 587 |
| 0.076 | 0.25 | 1.40 | 0.013 | 0.003 | 0.044 | 0.001 | 0.011 | 0.046 | 0.003 | 0.044 | 0.039 | 0.013 | 0.0007 | 404 | 1172 | 488 | 589 |
| 0.076 | 0.25 | 1.40 | 0.012 | 0.003 | 0.046 | 0.001 | 0.014 | 0.043 | 0.003 | 0.043 | 0.040 | 0.014 | 0.0006 | 402 | 1190 | 489 | 587 |
| 0.078 | 0.25 | 1.41 | 0.013 | 0.003 | 0.041 | 0.001 | 0.015 | 0.041 | 0.003 | 0.042 | 0.040 | 0.013 | 0.0006 | 413 | 1183 | 487 | 590 |
|  |  |  |  |  |  |  |  |  |  |  |  |  |  |  |  |  |  |
| **RF** | | | | | | | | | | | | | | | | | |
| C | Si | Mn | P | S | Cu | Sn | Ni | Cr | Mo | V | Nb | Ti | Ca | Time | Temp | YS | UTS |
| (wt%) | (wt%) | (wt%) | (wt%) | (wt%) | (wt%) | (wt%) | (wt%) | (wt%) | (wt%) | (wt%) | (wt%) | (wt%) | (wt%) | (s) | (℃) | (Mpa) | (Mpa) |
| 0.075 | 0.24 | 1.46 | 0.013 | 0.004 | 0.027 | 0.001 | 0.052 | 0.078 | 0.004 | 0.049 | 0.037 | 0.012 | 0.0003 | 342 | 1183 | 478 | 599 |
| 0.082 | 0.26 | 1.40 | 0.013 | 0.003 | 0.027 | 0.001 | 0.008 | 0.074 | 0.005 | 0.049 | 0.038 | 0.011 | 0.0003 | 412 | 1183 | 471 | 597 |
| 0.076 | 0.25 | 1.45 | 0.012 | 0.004 | 0.027 | 0.002 | 0.079 | 0.047 | 0.003 | 0.047 | 0.036 | 0.012 | 0.0003 | 422 | 1184 | 472 | 592 |
| 0.081 | 0.26 | 1.40 | 0.012 | 0.003 | 0.023 | 0.002 | 0.030 | 0.050 | 0.004 | 0.049 | 0.038 | 0.012 | 0.0003 | 399 | 1184 | 473 | 595 |
| 0.076 | 0.25 | 1.44 | 0.013 | 0.004 | 0.027 | 0.001 | 0.032 | 0.064 | 0.004 | 0.049 | 0.036 | 0.012 | 0.0008 | 411 | 1183 | 471 | 590 |
| 0.075 | 0.26 | 1.40 | 0.013 | 0.002 | 0.023 | 0.002 | 0.023 | 0.071 | 0.003 | 0.047 | 0.037 | 0.012 | 0.0003 | 416 | 1203 | 471 | 577 |
| 0.075 | 0.26 | 1.43 | 0.012 | 0.002 | 0.025 | 0.001 | 0.045 | 0.048 | 0.005 | 0.049 | 0.036 | 0.013 | 0.0003 | 417 | 1211 | 470 | 583 |
| 0.077 | 0.25 | 1.45 | 0.013 | 0.004 | 0.025 | 0.001 | 0.018 | 0.071 | 0.000 | 0.047 | 0.039 | 0.012 | 0.0003 | 405 | 1183 | 471 | 591 |
| 0.075 | 0.25 | 1.45 | 0.013 | 0.003 | 0.025 | 0.001 | 0.072 | 0.059 | 0.006 | 0.036 | 0.037 | 0.012 | 0.0003 | 374 | 1184 | 465 | 578 |
| 0.081 | 0.25 | 1.45 | 0.012 | 0.003 | 0.027 | 0.001 | 0.021 | 0.075 | 0.003 | 0.049 | 0.037 | 0.012 | 0.0003 | 427 | 1183 | 472 | 598 |
| 0.075 | 0.25 | 1.46 | 0.013 | 0.004 | 0.050 | 0.002 | 0.049 | 0.077 | 0.005 | 0.049 | 0.038 | 0.012 | 0.0003 | 368 | 1185 | 471 | 583 |
| 0.074 | 0.26 | 1.26 | 0.009 | 0.001 | 0.021 | 0.001 | 0.061 | 0.057 | 0.005 | 0.049 | 0.036 | 0.015 | 0.0002 | 423 | 1240 | 474 | 562 |
| 0.082 | 0.25 | 1.45 | 0.013 | 0.003 | 0.017 | 0.001 | 0.051 | 0.062 | 0.004 | 0.050 | 0.038 | 0.012 | 0.0003 | 413 | 1184 | 473 | 599 |
| 0.077 | 0.24 | 1.44 | 0.012 | 0.004 | 0.027 | 0.001 | 0.057 | 0.044 | 0.006 | 0.049 | 0.035 | 0.012 | 0.0003 | 364 | 1184 | 477 | 599 |
| 0.081 | 0.26 | 1.41 | 0.012 | 0.002 | 0.020 | 0.001 | 0.025 | 0.066 | 0.004 | 0.048 | 0.038 | 0.012 | 0.0003 | 377 | 1200 | 472 | 583 |
| 0.081 | 0.25 | 1.42 | 0.013 | 0.003 | 0.027 | 0.002 | 0.054 | 0.079 | 0.003 | 0.049 | 0.036 | 0.012 | 0.0003 | 414 | 1204 | 472 | 590 |
| 0.075 | 0.25 | 1.46 | 0.013 | 0.003 | 0.027 | 0.001 | 0.040 | 0.076 | 0.005 | 0.047 | 0.036 | 0.012 | 0.0003 | 432 | 1183 | 470 | 589 |
| 0.075 | 0.26 | 1.45 | 0.012 | 0.004 | 0.027 | 0.001 | 0.052 | 0.076 | 0.005 | 0.048 | 0.037 | 0.012 | 0.0003 | 418 | 1200 | 471 | 585 |
| 0.075 | 0.25 | 1.44 | 0.012 | 0.003 | 0.025 | 0.002 | 0.062 | 0.047 | 0.005 | 0.049 | 0.036 | 0.012 | 0.0003 | 431 | 1183 | 471 | 590 |
| 0.075 | 0.25 | 1.45 | 0.013 | 0.002 | 0.086 | 0.001 | 0.059 | 0.067 | 0.003 | 0.049 | 0.036 | 0.012 | 0.0003 | 434 | 1183 | 468 | 581 |
| 0.076 | 0.25 | 1.45 | 0.013 | 0.002 | 0.023 | 0.002 | 0.060 | 0.078 | 0.006 | 0.049 | 0.036 | 0.012 | 0.0003 | 410 | 1212 | 471 | 580 |
| 0.075 | 0.25 | 1.44 | 0.013 | 0.003 | 0.024 | 0.001 | 0.073 | 0.063 | 0.004 | 0.049 | 0.037 | 0.012 | 0.0003 | 413 | 1206 | 474 | 586 |
| 0.075 | 0.25 | 1.45 | 0.012 | 0.002 | 0.057 | 0.001 | 0.051 | 0.034 | 0.003 | 0.048 | 0.036 | 0.011 | 0.0008 | 408 | 1178 | 464 | 572 |
| 0.075 | 0.25 | 1.45 | 0.012 | 0.004 | 0.027 | 0.001 | 0.051 | 0.052 | 0.005 | 0.035 | 0.037 | 0.012 | 0.0003 | 338 | 1172 | 466 | 579 |
| 0.076 | 0.24 | 1.45 | 0.013 | 0.004 | 0.017 | 0.001 | 0.022 | 0.072 | 0.005 | 0.047 | 0.038 | 0.012 | 0.0003 | 450 | 1180 | 471 | 592 |
| 0.080 | 0.25 | 1.46 | 0.012 | 0.002 | 0.027 | 0.001 | 0.017 | 0.049 | 0.005 | 0.048 | 0.036 | 0.012 | 0.0003 | 413 | 1183 | 469 | 586 |
| 0.081 | 0.25 | 1.40 | 0.012 | 0.002 | 0.017 | 0.001 | 0.070 | 0.065 | 0.002 | 0.048 | 0.039 | 0.012 | 0.0003 | 412 | 1208 | 466 | 591 |
| 0.075 | 0.25 | 1.44 | 0.013 | 0.002 | 0.017 | 0.001 | 0.076 | 0.067 | 0.004 | 0.047 | 0.036 | 0.012 | 0.0003 | 416 | 1242 | 467 | 578 |
| 0.075 | 0.25 | 1.45 | 0.012 | 0.002 | 0.027 | 0.001 | 0.020 | 0.068 | 0.005 | 0.049 | 0.036 | 0.011 | 0.0003 | 436 | 1180 | 472 | 589 |
| 0.078 | 0.27 | 1.40 | 0.012 | 0.002 | 0.027 | 0.002 | 0.012 | 0.051 | 0.002 | 0.047 | 0.038 | 0.011 | 0.0005 | 402 | 1181 | 467 | 573 |
| 0.078 | 0.25 | 1.45 | 0.012 | 0.004 | 0.021 | 0.001 | 0.023 | 0.075 | 0.002 | 0.048 | 0.036 | 0.012 | 0.0003 | 444 | 1216 | 473 | 586 |
| 0.075 | 0.22 | 1.45 | 0.013 | 0.004 | 0.143 | 0.001 | 0.064 | 0.077 | 0.006 | 0.037 | 0.034 | 0.012 | 0.0004 | 452 | 1183 | 460 | 567 |
| 0.075 | 0.25 | 1.45 | 0.013 | 0.002 | 0.027 | 0.001 | 0.057 | 0.063 | 0.003 | 0.049 | 0.035 | 0.012 | 0.0005 | 408 | 1182 | 470 | 581 |
| 0.081 | 0.26 | 1.40 | 0.013 | 0.004 | 0.018 | 0.001 | 0.055 | 0.064 | 0.004 | 0.047 | 0.037 | 0.012 | 0.0003 | 397 | 1181 | 471 | 594 |
| 0.075 | 0.25 | 1.45 | 0.013 | 0.002 | 0.027 | 0.001 | 0.071 | 0.067 | 0.004 | 0.048 | 0.036 | 0.012 | 0.0003 | 426 | 1203 | 472 | 582 |
| 0.075 | 0.24 | 1.46 | 0.013 | 0.002 | 0.027 | 0.001 | 0.061 | 0.037 | 0.005 | 0.049 | 0.038 | 0.011 | 0.0003 | 431 | 1178 | 472 | 585 |
| 0.075 | 0.25 | 1.44 | 0.012 | 0.004 | 0.027 | 0.001 | 0.059 | 0.075 | 0.003 | 0.035 | 0.038 | 0.012 | 0.0003 | 353 | 1181 | 465 | 582 |
| 0.082 | 0.25 | 1.40 | 0.012 | 0.002 | 0.021 | 0.001 | 0.012 | 0.045 | 0.005 | 0.048 | 0.037 | 0.011 | 0.0003 | 441 | 945 | 465 | 590 |
| 0.080 | 0.25 | 1.44 | 0.013 | 0.003 | 0.027 | 0.001 | 0.079 | 0.069 | 0.004 | 0.048 | 0.038 | 0.012 | 0.0003 | 430 | 1160 | 465 | 581 |
| 0.075 | 0.25 | 1.45 | 0.013 | 0.002 | 0.080 | 0.001 | 0.049 | 0.065 | 0.005 | 0.049 | 0.037 | 0.016 | 0.0003 | 352 | 1216 | 469 | 572 |
| 0.075 | 0.25 | 1.44 | 0.013 | 0.004 | 0.025 | 0.001 | 0.052 | 0.042 | 0.004 | 0.048 | 0.036 | 0.012 | 0.0003 | 360 | 1183 | 473 | 589 |
| 0.075 | 0.25 | 1.46 | 0.012 | 0.004 | 0.027 | 0.001 | 0.023 | 0.046 | 0.004 | 0.049 | 0.036 | 0.012 | 0.0003 | 415 | 1178 | 474 | 597 |
| 0.081 | 0.25 | 1.40 | 0.012 | 0.002 | 0.017 | 0.001 | 0.012 | 0.029 | 0.005 | 0.048 | 0.042 | 0.011 | 0.0003 | 396 | 952 | 467 | 590 |
| 0.077 | 0.25 | 1.47 | 0.013 | 0.004 | 0.024 | 0.001 | 0.019 | 0.042 | 0.002 | 0.048 | 0.036 | 0.012 | 0.0003 | 413 | 1216 | 471 | 584 |
| 0.075 | 0.25 | 1.45 | 0.013 | 0.003 | 0.027 | 0.001 | 0.063 | 0.049 | 0.003 | 0.049 | 0.038 | 0.012 | 0.0003 | 409 | 1184 | 472 | 592 |
| 0.075 | 0.26 | 1.40 | 0.012 | 0.002 | 0.024 | 0.002 | 0.068 | 0.052 | 0.002 | 0.048 | 0.038 | 0.012 | 0.0002 | 426 | 1202 | 474 | 580 |
| 0.075 | 0.25 | 1.45 | 0.013 | 0.004 | 0.038 | 0.001 | 0.029 | 0.076 | 0.003 | 0.049 | 0.036 | 0.012 | 0.0003 | 414 | 1182 | 470 | 590 |
| 0.075 | 0.25 | 1.45 | 0.012 | 0.002 | 0.027 | 0.001 | 0.045 | 0.077 | 0.004 | 0.048 | 0.036 | 0.011 | 0.0003 | 418 | 1180 | 471 | 585 |
| 0.076 | 0.25 | 1.45 | 0.012 | 0.004 | 0.057 | 0.001 | 0.060 | 0.053 | 0.005 | 0.049 | 0.037 | 0.012 | 0.0003 | 357 | 1181 | 469 | 585 |
| 0.075 | 0.25 | 1.46 | 0.013 | 0.002 | 0.025 | 0.001 | 0.022 | 0.078 | 0.003 | 0.049 | 0.038 | 0.012 | 0.0008 | 427 | 1183 | 470 | 583 |
| 0.075 | 0.25 | 1.45 | 0.014 | 0.002 | 0.092 | 0.001 | 0.057 | 0.069 | 0.004 | 0.049 | 0.036 | 0.012 | 0.0003 | 426 | 1200 | 472 | 576 |
| 0.078 | 0.25 | 1.45 | 0.012 | 0.004 | 0.026 | 0.001 | 0.019 | 0.058 | 0.003 | 0.049 | 0.038 | 0.012 | 0.0003 | 415 | 1183 | 472 | 598 |
| 0.075 | 0.25 | 1.44 | 0.013 | 0.002 | 0.024 | 0.002 | 0.052 | 0.059 | 0.005 | 0.049 | 0.037 | 0.013 | 0.0003 | 411 | 1217 | 473 | 580 |
| 0.077 | 0.25 | 1.44 | 0.012 | 0.004 | 0.027 | 0.001 | 0.047 | 0.070 | 0.004 | 0.049 | 0.036 | 0.012 | 0.0003 | 410 | 1180 | 474 | 598 |
| 0.075 | 0.26 | 1.41 | 0.012 | 0.002 | 0.023 | 0.001 | 0.059 | 0.078 | 0.004 | 0.049 | 0.037 | 0.015 | 0.0003 | 413 | 1219 | 470 | 581 |
| 0.077 | 0.25 | 1.45 | 0.013 | 0.004 | 0.058 | 0.001 | 0.069 | 0.073 | 0.003 | 0.036 | 0.036 | 0.012 | 0.0008 | 415 | 1181 | 459 | 570 |
| 0.084 | 0.26 | 1.41 | 0.012 | 0.004 | 0.022 | 0.002 | 0.040 | 0.061 | 0.004 | 0.047 | 0.038 | 0.012 | 0.0003 | 401 | 1184 | 469 | 591 |
| 0.075 | 0.25 | 1.45 | 0.014 | 0.002 | 0.027 | 0.001 | 0.016 | 0.046 | 0.003 | 0.049 | 0.035 | 0.012 | 0.0003 | 415 | 1183 | 471 | 587 |
| 0.078 | 0.25 | 1.45 | 0.012 | 0.003 | 0.020 | 0.001 | 0.056 | 0.056 | 0.005 | 0.049 | 0.037 | 0.012 | 0.0003 | 429 | 1212 | 471 | 586 |
| 0.078 | 0.25 | 1.45 | 0.012 | 0.004 | 0.015 | 0.001 | 0.020 | 0.071 | 0.005 | 0.048 | 0.036 | 0.012 | 0.0003 | 421 | 1175 | 473 | 588 |
| 0.075 | 0.24 | 1.45 | 0.013 | 0.004 | 0.027 | 0.001 | 0.027 | 0.057 | 0.002 | 0.042 | 0.035 | 0.012 | 0.0003 | 411 | 1183 | 464 | 579 |
| 0.077 | 0.25 | 1.45 | 0.013 | 0.003 | 0.027 | 0.001 | 0.046 | 0.061 | 0.006 | 0.037 | 0.035 | 0.013 | 0.0003 | 373 | 1180 | 462 | 574 |
| 0.076 | 0.25 | 1.45 | 0.013 | 0.004 | 0.019 | 0.001 | 0.048 | 0.065 | 0.002 | 0.049 | 0.038 | 0.012 | 0.0003 | 447 | 1182 | 472 | 596 |
| 0.082 | 0.25 | 1.43 | 0.013 | 0.003 | 0.016 | 0.001 | 0.032 | 0.076 | 0.003 | 0.035 | 0.037 | 0.011 | 0.0003 | 396 | 969 | 458 | 579 |
| 0.075 | 0.25 | 1.45 | 0.013 | 0.003 | 0.086 | 0.001 | 0.048 | 0.055 | 0.005 | 0.048 | 0.036 | 0.012 | 0.0008 | 451 | 1181 | 465 | 574 |
| 0.076 | 0.24 | 1.43 | 0.012 | 0.004 | 0.022 | 0.001 | 0.021 | 0.068 | 0.003 | 0.049 | 0.038 | 0.012 | 0.0003 | 363 | 1183 | 476 | 592 |
| 0.075 | 0.26 | 1.45 | 0.013 | 0.003 | 0.027 | 0.001 | 0.022 | 0.064 | 0.004 | 0.049 | 0.036 | 0.013 | 0.0007 | 405 | 1243 | 466 | 574 |
| 0.075 | 0.25 | 1.44 | 0.012 | 0.003 | 0.027 | 0.001 | 0.058 | 0.054 | 0.003 | 0.048 | 0.038 | 0.012 | 0.0002 | 399 | 1211 | 473 | 584 |
| 0.075 | 0.25 | 1.45 | 0.013 | 0.002 | 0.027 | 0.001 | 0.021 | 0.053 | 0.005 | 0.048 | 0.037 | 0.012 | 0.0003 | 412 | 1184 | 472 | 585 |
| 0.077 | 0.25 | 1.45 | 0.012 | 0.004 | 0.027 | 0.001 | 0.067 | 0.074 | 0.003 | 0.035 | 0.035 | 0.012 | 0.0005 | 415 | 1183 | 464 | 578 |
| 0.078 | 0.26 | 1.40 | 0.012 | 0.002 | 0.027 | 0.001 | 0.006 | 0.078 | 0.005 | 0.049 | 0.038 | 0.012 | 0.0003 | 428 | 1181 | 470 | 588 |
| 0.082 | 0.26 | 1.40 | 0.013 | 0.002 | 0.020 | 0.002 | 0.022 | 0.060 | 0.003 | 0.048 | 0.037 | 0.012 | 0.0003 | 389 | 1186 | 472 | 583 |
| 0.075 | 0.25 | 1.45 | 0.012 | 0.004 | 0.027 | 0.001 | 0.039 | 0.058 | 0.005 | 0.038 | 0.038 | 0.012 | 0.0003 | 409 | 1182 | 464 | 583 |
| 0.075 | 0.25 | 1.45 | 0.013 | 0.003 | 0.024 | 0.002 | 0.078 | 0.067 | 0.002 | 0.049 | 0.037 | 0.012 | 0.0003 | 323 | 1202 | 479 | 582 |
| 0.075 | 0.25 | 1.45 | 0.012 | 0.002 | 0.024 | 0.001 | 0.021 | 0.076 | 0.003 | 0.049 | 0.036 | 0.013 | 0.0003 | 437 | 1239 | 472 | 582 |
| 0.082 | 0.25 | 1.40 | 0.013 | 0.003 | 0.017 | 0.001 | 0.071 | 0.068 | 0.002 | 0.036 | 0.037 | 0.011 | 0.0003 | 419 | 962 | 457 | 583 |
| 0.077 | 0.25 | 1.45 | 0.012 | 0.004 | 0.027 | 0.001 | 0.070 | 0.074 | 0.004 | 0.036 | 0.036 | 0.012 | 0.0003 | 398 | 1181 | 463 | 581 |
| 0.081 | 0.25 | 1.44 | 0.012 | 0.004 | 0.027 | 0.001 | 0.047 | 0.055 | 0.002 | 0.049 | 0.037 | 0.012 | 0.0003 | 409 | 1180 | 473 | 600 |
| 0.081 | 0.25 | 1.40 | 0.012 | 0.004 | 0.015 | 0.002 | 0.072 | 0.048 | 0.005 | 0.049 | 0.042 | 0.012 | 0.0003 | 414 | 1184 | 473 | 602 |
| 0.075 | 0.25 | 1.45 | 0.013 | 0.004 | 0.027 | 0.001 | 0.054 | 0.063 | 0.002 | 0.040 | 0.035 | 0.012 | 0.0003 | 416 | 1182 | 465 | 582 |
| 0.076 | 0.25 | 1.44 | 0.013 | 0.002 | 0.027 | 0.001 | 0.021 | 0.078 | 0.002 | 0.048 | 0.038 | 0.012 | 0.0003 | 408 | 1184 | 471 | 584 |
| 0.063 | 0.24 | 1.17 | 0.008 | 0.001 | 0.143 | 0.002 | 0.092 | 0.080 | 0.006 | 0.021 | 0.043 | 0.016 | 0.0017 | 420 | 1218 | 473 | 550 |
| 0.081 | 0.25 | 1.40 | 0.013 | 0.003 | 0.017 | 0.002 | 0.025 | 0.074 | 0.002 | 0.019 | 0.038 | 0.011 | 0.0013 | 435 | 1021 | 449 | 565 |
| 0.075 | 0.25 | 1.44 | 0.013 | 0.004 | 0.027 | 0.001 | 0.036 | 0.052 | 0.002 | 0.037 | 0.038 | 0.012 | 0.0003 | 431 | 1182 | 464 | 582 |
| 0.081 | 0.25 | 1.46 | 0.013 | 0.002 | 0.023 | 0.001 | 0.068 | 0.047 | 0.005 | 0.049 | 0.037 | 0.011 | 0.0008 | 450 | 1184 | 468 | 580 |
| 0.076 | 0.25 | 1.45 | 0.013 | 0.002 | 0.057 | 0.002 | 0.041 | 0.076 | 0.005 | 0.048 | 0.038 | 0.012 | 0.0003 | 414 | 1210 | 470 | 571 |
| 0.081 | 0.26 | 1.41 | 0.013 | 0.002 | 0.017 | 0.001 | 0.031 | 0.072 | 0.001 | 0.049 | 0.036 | 0.013 | 0.0003 | 415 | 1179 | 469 | 596 |
| 0.075 | 0.25 | 1.45 | 0.012 | 0.002 | 0.028 | 0.001 | 0.038 | 0.043 | 0.003 | 0.039 | 0.036 | 0.012 | 0.0008 | 411 | 1199 | 458 | 562 |
| 0.076 | 0.25 | 1.45 | 0.012 | 0.003 | 0.027 | 0.001 | 0.076 | 0.059 | 0.005 | 0.048 | 0.036 | 0.012 | 0.0003 | 337 | 1181 | 476 | 588 |
| 0.081 | 0.25 | 1.45 | 0.013 | 0.004 | 0.019 | 0.002 | 0.020 | 0.052 | 0.003 | 0.036 | 0.036 | 0.012 | 0.0003 | 338 | 1184 | 466 | 579 |
| 0.083 | 0.25 | 1.45 | 0.012 | 0.004 | 0.017 | 0.001 | 0.063 | 0.070 | 0.004 | 0.049 | 0.037 | 0.012 | 0.0003 | 411 | 1214 | 472 | 594 |
| 0.075 | 0.25 | 1.45 | 0.013 | 0.004 | 0.027 | 0.001 | 0.022 | 0.080 | 0.004 | 0.037 | 0.036 | 0.012 | 0.0003 | 397 | 1184 | 463 | 578 |
| 0.075 | 0.28 | 1.39 | 0.012 | 0.004 | 0.018 | 0.002 | 0.055 | 0.065 | 0.004 | 0.047 | 0.036 | 0.012 | 0.0003 | 418 | 1182 | 465 | 577 |
| 0.077 | 0.25 | 1.44 | 0.013 | 0.004 | 0.078 | 0.001 | 0.054 | 0.049 | 0.003 | 0.049 | 0.038 | 0.012 | 0.0005 | 408 | 1180 | 467 | 583 |
| 0.081 | 0.26 | 1.40 | 0.013 | 0.002 | 0.027 | 0.001 | 0.015 | 0.074 | 0.004 | 0.048 | 0.038 | 0.011 | 0.0003 | 390 | 1181 | 471 | 584 |
| 0.075 | 0.25 | 1.44 | 0.013 | 0.004 | 0.027 | 0.001 | 0.068 | 0.069 | 0.003 | 0.049 | 0.035 | 0.012 | 0.0005 | 416 | 1178 | 473 | 594 |
| 0.076 | 0.25 | 1.45 | 0.013 | 0.004 | 0.017 | 0.001 | 0.069 | 0.075 | 0.004 | 0.035 | 0.037 | 0.012 | 0.0003 | 411 | 1183 | 463 | 581 |
| 0.075 | 0.25 | 1.44 | 0.012 | 0.004 | 0.027 | 0.001 | 0.021 | 0.070 | 0.002 | 0.048 | 0.036 | 0.012 | 0.0003 | 408 | 1202 | 475 | 587 |
| 0.075 | 0.25 | 1.45 | 0.013 | 0.004 | 0.027 | 0.001 | 0.054 | 0.043 | 0.005 | 0.036 | 0.036 | 0.012 | 0.0003 | 415 | 1184 | 465 | 581 |
| 0.076 | 0.25 | 1.45 | 0.013 | 0.004 | 0.027 | 0.001 | 0.040 | 0.077 | 0.005 | 0.038 | 0.035 | 0.012 | 0.0003 | 338 | 1181 | 466 | 582 |
| 0.076 | 0.25 | 1.45 | 0.013 | 0.003 | 0.027 | 0.001 | 0.024 | 0.075 | 0.005 | 0.049 | 0.036 | 0.012 | 0.0005 | 370 | 1182 | 470 | 586 |
| 0.084 | 0.25 | 1.44 | 0.013 | 0.004 | 0.019 | 0.001 | 0.049 | 0.074 | 0.003 | 0.041 | 0.036 | 0.012 | 0.0003 | 413 | 1182 | 461 | 581 |
| 0.075 | 0.25 | 1.45 | 0.012 | 0.003 | 0.081 | 0.001 | 0.050 | 0.069 | 0.002 | 0.049 | 0.036 | 0.012 | 0.0003 | 415 | 1204 | 471 | 580 |
| 0.081 | 0.25 | 1.40 | 0.012 | 0.004 | 0.021 | 0.002 | 0.049 | 0.037 | 0.003 | 0.048 | 0.038 | 0.011 | 0.0002 | 447 | 1205 | 470 | 592 |
| 0.076 | 0.25 | 1.44 | 0.013 | 0.002 | 0.026 | 0.001 | 0.059 | 0.074 | 0.002 | 0.048 | 0.035 | 0.012 | 0.0003 | 409 | 1181 | 467 | 581 |
| 0.075 | 0.25 | 1.45 | 0.013 | 0.002 | 0.027 | 0.002 | 0.039 | 0.052 | 0.004 | 0.048 | 0.036 | 0.013 | 0.0003 | 397 | 1202 | 471 | 578 |
| 0.076 | 0.23 | 1.45 | 0.013 | 0.004 | 0.027 | 0.001 | 0.069 | 0.074 | 0.005 | 0.047 | 0.035 | 0.012 | 0.0003 | 385 | 1181 | 469 | 589 |
| 0.078 | 0.24 | 1.45 | 0.013 | 0.004 | 0.042 | 0.001 | 0.066 | 0.060 | 0.003 | 0.049 | 0.036 | 0.012 | 0.0003 | 409 | 1183 | 471 | 588 |
| 0.081 | 0.26 | 1.41 | 0.013 | 0.003 | 0.016 | 0.001 | 0.024 | 0.053 | 0.003 | 0.049 | 0.037 | 0.011 | 0.0003 | 443 | 1202 | 471 | 601 |
| 0.075 | 0.25 | 1.45 | 0.013 | 0.003 | 0.026 | 0.001 | 0.057 | 0.079 | 0.005 | 0.036 | 0.037 | 0.012 | 0.0003 | 397 | 1181 | 462 | 577 |
| 0.076 | 0.25 | 1.46 | 0.013 | 0.002 | 0.027 | 0.001 | 0.025 | 0.060 | 0.003 | 0.049 | 0.036 | 0.012 | 0.0006 | 411 | 1184 | 469 | 584 |
| 0.082 | 0.26 | 1.41 | 0.013 | 0.003 | 0.017 | 0.001 | 0.063 | 0.052 | 0.004 | 0.047 | 0.038 | 0.012 | 0.0003 | 412 | 1197 | 467 | 592 |
| 0.081 | 0.26 | 1.41 | 0.012 | 0.003 | 0.017 | 0.002 | 0.041 | 0.060 | 0.005 | 0.049 | 0.036 | 0.013 | 0.0005 | 424 | 1178 | 464 | 587 |
| 0.075 | 0.25 | 1.44 | 0.012 | 0.004 | 0.028 | 0.001 | 0.023 | 0.071 | 0.004 | 0.049 | 0.036 | 0.012 | 0.0003 | 418 | 1183 | 475 | 599 |
| 0.077 | 0.26 | 1.46 | 0.013 | 0.003 | 0.016 | 0.001 | 0.018 | 0.064 | 0.005 | 0.035 | 0.039 | 0.011 | 0.0005 | 418 | 1183 | 459 | 569 |
| 0.076 | 0.25 | 1.46 | 0.012 | 0.002 | 0.023 | 0.001 | 0.068 | 0.057 | 0.002 | 0.049 | 0.036 | 0.012 | 0.0003 | 412 | 1183 | 470 | 589 |
| 0.075 | 0.26 | 1.39 | 0.012 | 0.002 | 0.027 | 0.002 | 0.044 | 0.025 | 0.005 | 0.047 | 0.038 | 0.013 | 0.0002 | 406 | 1186 | 467 | 573 |
| 0.075 | 0.28 | 1.45 | 0.013 | 0.002 | 0.016 | 0.001 | 0.044 | 0.138 | 0.001 | 0.049 | 0.036 | 0.011 | 0.0005 | 404 | 1181 | 466 | 573 |
| 0.081 | 0.26 | 1.41 | 0.012 | 0.004 | 0.023 | 0.002 | 0.008 | 0.074 | 0.005 | 0.049 | 0.036 | 0.012 | 0.0002 | 446 | 1204 | 476 | 597 |
| 0.076 | 0.25 | 1.46 | 0.013 | 0.002 | 0.027 | 0.001 | 0.070 | 0.065 | 0.004 | 0.049 | 0.038 | 0.012 | 0.0003 | 376 | 1183 | 471 | 586 |
| 0.082 | 0.25 | 1.43 | 0.012 | 0.002 | 0.013 | 0.001 | 0.007 | 0.068 | 0.004 | 0.049 | 0.036 | 0.012 | 0.0002 | 417 | 967 | 465 | 589 |
| 0.075 | 0.25 | 1.45 | 0.013 | 0.004 | 0.027 | 0.001 | 0.032 | 0.068 | 0.003 | 0.037 | 0.035 | 0.012 | 0.0003 | 424 | 1178 | 465 | 581 |
| 0.076 | 0.25 | 1.45 | 0.013 | 0.002 | 0.024 | 0.001 | 0.030 | 0.061 | 0.005 | 0.049 | 0.037 | 0.012 | 0.0003 | 430 | 1208 | 474 | 581 |
| 0.081 | 0.26 | 1.44 | 0.012 | 0.004 | 0.015 | 0.001 | 0.057 | 0.048 | 0.003 | 0.035 | 0.036 | 0.011 | 0.0003 | 405 | 1179 | 457 | 577 |
| 0.075 | 0.25 | 1.44 | 0.012 | 0.002 | 0.098 | 0.001 | 0.063 | 0.065 | 0.002 | 0.048 | 0.038 | 0.012 | 0.0003 | 417 | 1200 | 470 | 574 |
| 0.075 | 0.25 | 1.45 | 0.012 | 0.004 | 0.022 | 0.001 | 0.025 | 0.061 | 0.001 | 0.043 | 0.036 | 0.012 | 0.0005 | 422 | 1180 | 461 | 575 |
| 0.075 | 0.25 | 1.44 | 0.013 | 0.003 | 0.027 | 0.001 | 0.024 | 0.066 | 0.002 | 0.048 | 0.036 | 0.012 | 0.0005 | 424 | 1180 | 468 | 584 |
| 0.076 | 0.25 | 1.45 | 0.013 | 0.004 | 0.027 | 0.001 | 0.047 | 0.078 | 0.002 | 0.037 | 0.035 | 0.012 | 0.0003 | 361 | 1181 | 464 | 580 |
| 0.081 | 0.26 | 1.41 | 0.012 | 0.002 | 0.022 | 0.001 | 0.024 | 0.067 | 0.004 | 0.049 | 0.036 | 0.012 | 0.0003 | 395 | 1198 | 470 | 589 |
| 0.078 | 0.28 | 1.40 | 0.012 | 0.002 | 0.023 | 0.002 | 0.067 | 0.066 | 0.004 | 0.035 | 0.036 | 0.014 | 0.0003 | 423 | 1214 | 456 | 558 |
| 0.075 | 0.25 | 1.47 | 0.013 | 0.003 | 0.023 | 0.002 | 0.059 | 0.068 | 0.005 | 0.049 | 0.036 | 0.014 | 0.0003 | 431 | 1238 | 470 | 577 |
| 0.075 | 0.25 | 1.45 | 0.012 | 0.002 | 0.025 | 0.001 | 0.024 | 0.061 | 0.005 | 0.048 | 0.037 | 0.012 | 0.0003 | 381 | 1203 | 472 | 579 |
| 0.075 | 0.25 | 1.46 | 0.012 | 0.003 | 0.027 | 0.001 | 0.056 | 0.065 | 0.005 | 0.049 | 0.036 | 0.012 | 0.0003 | 388 | 1184 | 474 | 592 |
| 0.076 | 0.25 | 1.46 | 0.012 | 0.002 | 0.024 | 0.001 | 0.053 | 0.066 | 0.004 | 0.049 | 0.038 | 0.012 | 0.0003 | 415 | 1183 | 471 | 589 |
| 0.075 | 0.26 | 1.39 | 0.012 | 0.002 | 0.024 | 0.002 | 0.079 | 0.063 | 0.003 | 0.048 | 0.036 | 0.014 | 0.0003 | 412 | 1226 | 470 | 574 |
| 0.074 | 0.25 | 1.45 | 0.012 | 0.004 | 0.027 | 0.001 | 0.055 | 0.052 | 0.004 | 0.038 | 0.035 | 0.012 | 0.0003 | 324 | 1181 | 466 | 582 |
| 0.081 | 0.25 | 1.45 | 0.013 | 0.004 | 0.027 | 0.001 | 0.045 | 0.045 | 0.004 | 0.037 | 0.037 | 0.012 | 0.0003 | 406 | 1182 | 462 | 582 |
| 0.082 | 0.25 | 1.41 | 0.012 | 0.003 | 0.017 | 0.001 | 0.034 | 0.064 | 0.001 | 0.049 | 0.038 | 0.010 | 0.0003 | 393 | 951 | 470 | 594 |
| 0.076 | 0.25 | 1.44 | 0.013 | 0.002 | 0.027 | 0.001 | 0.013 | 0.057 | 0.002 | 0.048 | 0.037 | 0.012 | 0.0003 | 410 | 1181 | 469 | 584 |
| 0.074 | 0.26 | 1.28 | 0.010 | 0.002 | 0.021 | 0.001 | 0.016 | 0.065 | 0.004 | 0.047 | 0.037 | 0.012 | 0.0002 | 427 | 1186 | 473 | 565 |
| 0.081 | 0.25 | 1.45 | 0.013 | 0.004 | 0.017 | 0.001 | 0.033 | 0.063 | 0.003 | 0.049 | 0.036 | 0.012 | 0.0003 | 433 | 1181 | 474 | 602 |
| 0.078 | 0.25 | 1.45 | 0.012 | 0.002 | 0.059 | 0.001 | 0.024 | 0.064 | 0.003 | 0.049 | 0.037 | 0.011 | 0.0009 | 416 | 1184 | 467 | 575 |
| 0.075 | 0.25 | 1.45 | 0.013 | 0.004 | 0.020 | 0.001 | 0.019 | 0.063 | 0.004 | 0.048 | 0.036 | 0.012 | 0.0003 | 411 | 1184 | 473 | 592 |
| 0.082 | 0.24 | 1.41 | 0.013 | 0.004 | 0.017 | 0.001 | 0.072 | 0.079 | 0.004 | 0.048 | 0.034 | 0.012 | 0.0003 | 411 | 954 | 466 | 584 |
| 0.076 | 0.25 | 1.45 | 0.013 | 0.003 | 0.027 | 0.001 | 0.040 | 0.073 | 0.003 | 0.036 | 0.035 | 0.012 | 0.0003 | 408 | 1181 | 462 | 577 |
| 0.081 | 0.26 | 1.37 | 0.013 | 0.002 | 0.030 | 0.002 | 0.076 | 0.054 | 0.002 | 0.047 | 0.038 | 0.013 | 0.0005 | 399 | 1224 | 458 | 564 |
| 0.075 | 0.25 | 1.44 | 0.012 | 0.004 | 0.027 | 0.001 | 0.044 | 0.061 | 0.004 | 0.035 | 0.036 | 0.012 | 0.0003 | 410 | 1183 | 465 | 583 |
| 0.076 | 0.25 | 1.45 | 0.013 | 0.004 | 0.027 | 0.001 | 0.067 | 0.077 | 0.005 | 0.035 | 0.036 | 0.012 | 0.0003 | 436 | 1183 | 464 | 582 |
| 0.076 | 0.25 | 1.45 | 0.013 | 0.003 | 0.024 | 0.001 | 0.018 | 0.032 | 0.003 | 0.049 | 0.036 | 0.013 | 0.0003 | 339 | 1213 | 480 | 586 |
| 0.076 | 0.24 | 1.46 | 0.013 | 0.003 | 0.027 | 0.001 | 0.066 | 0.066 | 0.007 | 0.049 | 0.038 | 0.013 | 0.0003 | 366 | 1183 | 471 | 586 |
| 0.081 | 0.26 | 1.40 | 0.013 | 0.002 | 0.023 | 0.001 | 0.056 | 0.032 | 0.005 | 0.049 | 0.037 | 0.013 | 0.0003 | 405 | 1222 | 469 | 590 |
| 0.075 | 0.25 | 1.47 | 0.013 | 0.003 | 0.020 | 0.001 | 0.021 | 0.068 | 0.003 | 0.049 | 0.036 | 0.012 | 0.0003 | 435 | 1216 | 472 | 582 |
| 0.075 | 0.25 | 1.45 | 0.013 | 0.004 | 0.027 | 0.002 | 0.020 | 0.062 | 0.004 | 0.049 | 0.036 | 0.012 | 0.0003 | 411 | 1183 | 475 | 597 |
| 0.077 | 0.25 | 1.45 | 0.013 | 0.002 | 0.024 | 0.001 | 0.071 | 0.062 | 0.004 | 0.049 | 0.036 | 0.011 | 0.0005 | 426 | 1186 | 471 | 582 |
| 0.076 | 0.25 | 1.46 | 0.012 | 0.002 | 0.027 | 0.001 | 0.015 | 0.071 | 0.004 | 0.048 | 0.036 | 0.012 | 0.0005 | 450 | 1182 | 469 | 582 |
| 0.078 | 0.27 | 1.41 | 0.012 | 0.002 | 0.023 | 0.002 | 0.073 | 0.040 | 0.004 | 0.049 | 0.037 | 0.011 | 0.0005 | 450 | 1180 | 467 | 576 |
| 0.075 | 0.25 | 1.45 | 0.013 | 0.003 | 0.025 | 0.001 | 0.050 | 0.058 | 0.004 | 0.049 | 0.037 | 0.012 | 0.0003 | 414 | 1183 | 471 | 591 |
| 0.081 | 0.22 | 1.45 | 0.012 | 0.002 | 0.163 | 0.001 | 0.012 | 0.115 | 0.003 | 0.031 | 0.034 | 0.016 | 0.0013 | 391 | 1228 | 460 | 555 |
| 0.075 | 0.26 | 1.41 | 0.012 | 0.004 | 0.022 | 0.002 | 0.040 | 0.061 | 0.004 | 0.047 | 0.038 | 0.012 | 0.0003 | 412 | 1184 | 469 | 587 |
| 0.075 | 0.25 | 1.45 | 0.012 | 0.004 | 0.018 | 0.001 | 0.066 | 0.073 | 0.002 | 0.035 | 0.039 | 0.012 | 0.0003 | 415 | 1184 | 462 | 580 |
| 0.076 | 0.25 | 1.46 | 0.013 | 0.002 | 0.027 | 0.001 | 0.038 | 0.061 | 0.002 | 0.035 | 0.037 | 0.012 | 0.0005 | 411 | 1183 | 461 | 569 |
| 0.081 | 0.26 | 1.43 | 0.012 | 0.002 | 0.024 | 0.002 | 0.066 | 0.056 | 0.003 | 0.049 | 0.036 | 0.014 | 0.0003 | 407 | 1205 | 468 | 587 |
| 0.078 | 0.29 | 1.40 | 0.012 | 0.002 | 0.022 | 0.002 | 0.024 | 0.074 | 0.004 | 0.048 | 0.038 | 0.011 | 0.0005 | 438 | 1185 | 465 | 575 |
| 0.074 | 0.25 | 1.46 | 0.013 | 0.004 | 0.027 | 0.001 | 0.013 | 0.035 | 0.004 | 0.048 | 0.036 | 0.012 | 0.0003 | 320 | 1183 | 478 | 595 |
| 0.075 | 0.25 | 1.47 | 0.012 | 0.004 | 0.027 | 0.001 | 0.060 | 0.046 | 0.004 | 0.049 | 0.036 | 0.013 | 0.0003 | 397 | 1184 | 474 | 592 |
| 0.075 | 0.25 | 1.45 | 0.012 | 0.004 | 0.026 | 0.001 | 0.052 | 0.066 | 0.001 | 0.040 | 0.040 | 0.012 | 0.0003 | 409 | 1181 | 463 | 581 |
| 0.074 | 0.29 | 1.35 | 0.013 | 0.002 | 0.027 | 0.002 | 0.050 | 0.059 | 0.005 | 0.049 | 0.036 | 0.014 | 0.0003 | 430 | 1244 | 468 | 566 |
| 0.075 | 0.25 | 1.45 | 0.012 | 0.004 | 0.027 | 0.001 | 0.057 | 0.055 | 0.004 | 0.035 | 0.035 | 0.012 | 0.0003 | 408 | 1183 | 466 | 582 |
| 0.076 | 0.24 | 1.44 | 0.013 | 0.004 | 0.027 | 0.001 | 0.055 | 0.065 | 0.004 | 0.049 | 0.035 | 0.012 | 0.0003 | 414 | 1183 | 477 | 599 |
| 0.076 | 0.25 | 1.45 | 0.012 | 0.002 | 0.019 | 0.001 | 0.042 | 0.057 | 0.005 | 0.049 | 0.037 | 0.012 | 0.0005 | 417 | 1166 | 470 | 580 |
| 0.072 | 0.23 | 1.31 | 0.010 | 0.002 | 0.043 | 0.001 | 0.038 | 0.049 | 0.004 | 0.048 | 0.037 | 0.012 | 0.0002 | 407 | 1221 | 476 | 559 |
| 0.076 | 0.25 | 1.45 | 0.013 | 0.003 | 0.023 | 0.001 | 0.019 | 0.038 | 0.003 | 0.049 | 0.036 | 0.011 | 0.0003 | 410 | 1181 | 471 | 594 |
| 0.076 | 0.25 | 1.45 | 0.012 | 0.004 | 0.090 | 0.001 | 0.068 | 0.058 | 0.002 | 0.036 | 0.036 | 0.012 | 0.0003 | 397 | 1182 | 460 | 574 |
| 0.074 | 0.28 | 1.35 | 0.012 | 0.001 | 0.027 | 0.002 | 0.056 | 0.024 | 0.004 | 0.049 | 0.036 | 0.011 | 0.0005 | 415 | 1184 | 473 | 572 |
| 0.076 | 0.24 | 1.44 | 0.012 | 0.003 | 0.027 | 0.002 | 0.021 | 0.061 | 0.004 | 0.049 | 0.035 | 0.013 | 0.0003 | 404 | 1182 | 468 | 586 |
| 0.075 | 0.25 | 1.45 | 0.013 | 0.003 | 0.030 | 0.001 | 0.043 | 0.072 | 0.004 | 0.049 | 0.036 | 0.012 | 0.0003 | 429 | 1183 | 468 | 583 |
| 0.083 | 0.25 | 1.43 | 0.014 | 0.003 | 0.013 | 0.001 | 0.041 | 0.047 | 0.004 | 0.038 | 0.036 | 0.011 | 0.0002 | 414 | 956 | 457 | 581 |
| 0.075 | 0.25 | 1.46 | 0.013 | 0.003 | 0.027 | 0.001 | 0.024 | 0.067 | 0.002 | 0.047 | 0.040 | 0.012 | 0.0003 | 409 | 1181 | 469 | 588 |
| 0.076 | 0.25 | 1.47 | 0.013 | 0.002 | 0.027 | 0.001 | 0.023 | 0.038 | 0.004 | 0.049 | 0.036 | 0.012 | 0.0005 | 409 | 1182 | 469 | 583 |
| 0.075 | 0.25 | 1.43 | 0.012 | 0.004 | 0.016 | 0.001 | 0.049 | 0.070 | 0.004 | 0.038 | 0.037 | 0.012 | 0.0003 | 446 | 1054 | 457 | 574 |
| 0.081 | 0.25 | 1.41 | 0.013 | 0.004 | 0.024 | 0.002 | 0.080 | 0.049 | 0.005 | 0.048 | 0.040 | 0.012 | 0.0003 | 396 | 1181 | 470 | 590 |
| 0.081 | 0.26 | 1.42 | 0.013 | 0.002 | 0.021 | 0.002 | 0.013 | 0.110 | 0.005 | 0.049 | 0.040 | 0.013 | 0.0005 | 435 | 1198 | 465 | 578 |
| 0.075 | 0.25 | 1.44 | 0.012 | 0.004 | 0.027 | 0.001 | 0.039 | 0.061 | 0.002 | 0.037 | 0.036 | 0.012 | 0.0003 | 414 | 1183 | 465 | 583 |
| 0.081 | 0.25 | 1.45 | 0.015 | 0.003 | 0.017 | 0.001 | 0.059 | 0.066 | 0.003 | 0.048 | 0.036 | 0.012 | 0.0003 | 449 | 1205 | 470 | 587 |
| 0.075 | 0.24 | 1.45 | 0.013 | 0.004 | 0.027 | 0.001 | 0.024 | 0.066 | 0.004 | 0.049 | 0.037 | 0.012 | 0.0003 | 369 | 1181 | 476 | 598 |
| 0.075 | 0.26 | 1.45 | 0.013 | 0.004 | 0.027 | 0.001 | 0.064 | 0.078 | 0.003 | 0.041 | 0.037 | 0.012 | 0.0003 | 419 | 1181 | 462 | 579 |
| 0.076 | 0.25 | 1.44 | 0.013 | 0.002 | 0.027 | 0.001 | 0.024 | 0.067 | 0.004 | 0.049 | 0.036 | 0.013 | 0.0003 | 413 | 1217 | 473 | 579 |
| 0.081 | 0.25 | 1.45 | 0.012 | 0.003 | 0.017 | 0.001 | 0.043 | 0.043 | 0.003 | 0.049 | 0.038 | 0.012 | 0.0003 | 412 | 1164 | 475 | 594 |
| 0.076 | 0.25 | 1.44 | 0.013 | 0.002 | 0.028 | 0.001 | 0.067 | 0.074 | 0.004 | 0.047 | 0.036 | 0.012 | 0.0005 | 404 | 1204 | 467 | 574 |
| 0.075 | 0.24 | 1.46 | 0.012 | 0.002 | 0.024 | 0.001 | 0.044 | 0.060 | 0.006 | 0.048 | 0.036 | 0.011 | 0.0003 | 349 | 1184 | 469 | 580 |
| 0.075 | 0.25 | 1.45 | 0.012 | 0.003 | 0.048 | 0.002 | 0.058 | 0.060 | 0.004 | 0.038 | 0.036 | 0.012 | 0.0003 | 408 | 1180 | 459 | 571 |
| 0.075 | 0.24 | 1.46 | 0.013 | 0.004 | 0.025 | 0.001 | 0.023 | 0.063 | 0.004 | 0.038 | 0.035 | 0.012 | 0.0003 | 339 | 1178 | 464 | 580 |
| 0.077 | 0.25 | 1.45 | 0.013 | 0.003 | 0.024 | 0.002 | 0.051 | 0.075 | 0.004 | 0.049 | 0.035 | 0.011 | 0.0004 | 418 | 1181 | 462 | 577 |
| 0.075 | 0.25 | 1.45 | 0.012 | 0.002 | 0.020 | 0.001 | 0.039 | 0.062 | 0.003 | 0.042 | 0.036 | 0.012 | 0.0005 | 436 | 1183 | 461 | 570 |
| 0.076 | 0.25 | 1.45 | 0.013 | 0.003 | 0.027 | 0.001 | 0.013 | 0.070 | 0.006 | 0.048 | 0.036 | 0.012 | 0.0003 | 396 | 1181 | 470 | 583 |
| 0.076 | 0.26 | 1.28 | 0.009 | 0.004 | 0.027 | 0.001 | 0.068 | 0.053 | 0.003 | 0.048 | 0.036 | 0.012 | 0.0003 | 407 | 1215 | 468 | 565 |
| 0.081 | 0.25 | 1.44 | 0.013 | 0.004 | 0.027 | 0.001 | 0.051 | 0.078 | 0.004 | 0.038 | 0.035 | 0.012 | 0.0003 | 415 | 1183 | 463 | 581 |
| 0.076 | 0.25 | 1.45 | 0.012 | 0.003 | 0.026 | 0.001 | 0.056 | 0.046 | 0.003 | 0.049 | 0.036 | 0.012 | 0.0003 | 417 | 1183 | 470 | 592 |
| 0.075 | 0.25 | 1.45 | 0.012 | 0.002 | 0.085 | 0.001 | 0.054 | 0.052 | 0.002 | 0.036 | 0.036 | 0.012 | 0.0003 | 413 | 1200 | 461 | 563 |
| 0.077 | 0.24 | 1.45 | 0.013 | 0.002 | 0.027 | 0.001 | 0.024 | 0.063 | 0.005 | 0.049 | 0.035 | 0.012 | 0.0003 | 407 | 1181 | 472 | 588 |
|  |  |  |  |  |  |  |  |  |  |  |  |  |  |  |  |  |  |
| **Gradient Boost** | | | | | | | | | | | | | | | | | |
| C | Si | Mn | P | S | Cu | Sn | Ni | Cr | Mo | V | Nb | Ti | Ca | Time | Temp | YS | UTS |
| (wt%) | (wt%) | (wt%) | (wt%) | (wt%) | (wt%) | (wt%) | (wt%) | (wt%) | (wt%) | (wt%) | (wt%) | (wt%) | (wt%) | (s) | (℃) | (Mpa) | (Mpa) |
| 0.081 | 0.25 | 1.43 | 0.013 | 0.004 | 0.029 | 0.001 | 0.111 | 0.089 | 0.006 | 0.049 | 0.036 | 0.014 | 0.0016 | 342 | 993 | 477 | 606 |
| 0.078 | 0.23 | 1.16 | 0.010 | 0.003 | 0.163 | 0.001 | 0.052 | 0.106 | 0.002 | 0.037 | 0.037 | 0.016 | 0.0002 | 452 | 1262 | 509 | 626 |
| 0.079 | 0.24 | 1.26 | 0.013 | 0.002 | 0.049 | 0.001 | 0.051 | 0.082 | 0.009 | 0.049 | 0.039 | 0.016 | 0.0027 | 427 | 1266 | 522 | 620 |
| 0.078 | 0.22 | 1.58 | 0.013 | 0.004 | 0.161 | 0.001 | 0.070 | 0.068 | 0.001 | 0.018 | 0.036 | 0.016 | 0.0019 | 438 | 1214 | 526 | 635 |
| 0.079 | 0.24 | 1.32 | 0.010 | 0.001 | 0.162 | 0.001 | 0.055 | 0.106 | 0.006 | 0.036 | 0.040 | 0.016 | 0.0027 | 452 | 1232 | 506 | 616 |
| 0.079 | 0.22 | 1.16 | 0.010 | 0.003 | 0.169 | 0.001 | 0.078 | 0.070 | 0.006 | 0.036 | 0.041 | 0.016 | 0.0002 | 434 | 1271 | 507 | 635 |
| 0.079 | 0.24 | 1.16 | 0.013 | 0.002 | 0.162 | 0.001 | 0.046 | 0.082 | 0.007 | 0.049 | 0.039 | 0.016 | 0.0003 | 434 | 1238 | 522 | 631 |
| 0.072 | 0.22 | 1.58 | 0.015 | 0.003 | 0.163 | 0.002 | 0.042 | 0.054 | 0.002 | 0.049 | 0.039 | 0.017 | 0.0026 | 452 | 1221 | 523 | 617 |
| 0.079 | 0.24 | 1.45 | 0.009 | 0.004 | 0.171 | 0.002 | 0.138 | 0.115 | 0.006 | 0.020 | 0.037 | 0.016 | 0.0027 | 426 | 1166 | 523 | 628 |
| 0.079 | 0.22 | 1.16 | 0.010 | 0.004 | 0.165 | 0.001 | 0.033 | 0.076 | 0.005 | 0.040 | 0.038 | 0.016 | 0.0019 | 423 | 1221 | 522 | 644 |
| 0.079 | 0.22 | 1.46 | 0.009 | 0.004 | 0.165 | 0.001 | 0.091 | 0.073 | 0.005 | 0.026 | 0.037 | 0.016 | 0.0019 | 446 | 1217 | 504 | 640 |
| 0.078 | 0.22 | 1.21 | 0.010 | 0.003 | 0.164 | 0.001 | 0.045 | 0.135 | 0.006 | 0.018 | 0.039 | 0.016 | 0.0026 | 446 | 1259 | 499 | 615 |
| 0.079 | 0.23 | 1.43 | 0.009 | 0.003 | 0.175 | 0.001 | 0.144 | 0.118 | 0.001 | 0.020 | 0.038 | 0.016 | 0.0027 | 439 | 1217 | 505 | 624 |
| 0.078 | 0.22 | 1.14 | 0.009 | 0.002 | 0.162 | 0.001 | 0.075 | 0.034 | 0.006 | 0.021 | 0.040 | 0.016 | 0.0026 | 431 | 1261 | 520 | 626 |
| 0.084 | 0.30 | 1.30 | 0.013 | 0.001 | 0.021 | 0.001 | 0.071 | 0.089 | 0.006 | 0.050 | 0.036 | 0.011 | 0.0010 | 458 | 1237 | 505 | 601 |
| 0.081 | 0.22 | 1.41 | 0.010 | 0.004 | 0.165 | 0.001 | 0.062 | 0.078 | 0.006 | 0.036 | 0.036 | 0.016 | 0.0017 | 413 | 1221 | 500 | 638 |
| 0.079 | 0.24 | 1.15 | 0.012 | 0.003 | 0.160 | 0.001 | 0.052 | 0.066 | 0.005 | 0.049 | 0.040 | 0.016 | 0.0002 | 414 | 1275 | 509 | 642 |
| 0.072 | 0.24 | 1.58 | 0.013 | 0.001 | 0.163 | 0.001 | 0.068 | 0.051 | 0.006 | 0.019 | 0.036 | 0.016 | 0.0027 | 440 | 1216 | 520 | 593 |
| 0.079 | 0.30 | 1.59 | 0.013 | 0.004 | 0.126 | 0.001 | 0.020 | 0.045 | 0.006 | 0.017 | 0.042 | 0.011 | 0.0027 | 365 | 1161 | 543 | 605 |
| 0.079 | 0.22 | 1.15 | 0.009 | 0.002 | 0.161 | 0.002 | 0.077 | 0.070 | 0.007 | 0.020 | 0.036 | 0.016 | 0.0027 | 427 | 1261 | 525 | 621 |
| 0.079 | 0.22 | 1.15 | 0.013 | 0.004 | 0.098 | 0.001 | 0.065 | 0.078 | 0.004 | 0.039 | 0.037 | 0.016 | 0.0010 | 454 | 1260 | 538 | 623 |
| 0.079 | 0.31 | 1.58 | 0.012 | 0.004 | 0.050 | 0.001 | 0.043 | 0.095 | 0.005 | 0.020 | 0.042 | 0.012 | 0.0011 | 354 | 989 | 515 | 614 |
| 0.079 | 0.22 | 1.45 | 0.009 | 0.003 | 0.165 | 0.001 | 0.073 | 0.069 | 0.007 | 0.036 | 0.039 | 0.016 | 0.0027 | 451 | 1216 | 504 | 621 |
| 0.079 | 0.30 | 1.45 | 0.010 | 0.004 | 0.072 | 0.001 | 0.056 | 0.059 | 0.005 | 0.036 | 0.039 | 0.013 | 0.0028 | 396 | 1153 | 507 | 610 |
| 0.079 | 0.22 | 1.46 | 0.010 | 0.003 | 0.173 | 0.001 | 0.019 | 0.033 | 0.008 | 0.019 | 0.040 | 0.016 | 0.0025 | 425 | 1219 | 490 | 624 |
| 0.082 | 0.25 | 1.15 | 0.009 | 0.004 | 0.168 | 0.002 | 0.077 | 0.071 | 0.005 | 0.047 | 0.041 | 0.011 | 0.0027 | 457 | 1233 | 540 | 629 |
| 0.078 | 0.22 | 1.15 | 0.013 | 0.003 | 0.071 | 0.001 | 0.077 | 0.034 | 0.005 | 0.049 | 0.039 | 0.016 | 0.0026 | 437 | 1271 | 532 | 635 |
| 0.078 | 0.22 | 1.58 | 0.014 | 0.004 | 0.153 | 0.002 | 0.076 | 0.103 | 0.003 | 0.049 | 0.039 | 0.016 | 0.0005 | 345 | 1210 | 541 | 644 |
| 0.079 | 0.30 | 1.54 | 0.013 | 0.004 | 0.060 | 0.001 | 0.094 | 0.068 | 0.006 | 0.049 | 0.036 | 0.013 | 0.0008 | 445 | 1178 | 519 | 633 |
| 0.079 | 0.24 | 1.16 | 0.013 | 0.001 | 0.017 | 0.001 | 0.061 | 0.043 | 0.005 | 0.049 | 0.037 | 0.016 | 0.0027 | 454 | 1253 | 546 | 631 |
| 0.079 | 0.22 | 1.16 | 0.014 | 0.002 | 0.161 | 0.002 | 0.064 | 0.124 | 0.003 | 0.044 | 0.040 | 0.016 | 0.0019 | 427 | 1238 | 497 | 621 |
| 0.079 | 0.30 | 1.14 | 0.015 | 0.002 | 0.154 | 0.002 | 0.064 | 0.095 | 0.004 | 0.037 | 0.042 | 0.011 | 0.0027 | 459 | 1272 | 502 | 619 |
| 0.079 | 0.22 | 1.17 | 0.013 | 0.002 | 0.162 | 0.002 | 0.058 | 0.108 | 0.006 | 0.049 | 0.036 | 0.016 | 0.0003 | 461 | 1236 | 536 | 626 |
| 0.082 | 0.29 | 1.51 | 0.012 | 0.001 | 0.163 | 0.001 | 0.075 | 0.106 | 0.004 | 0.022 | 0.040 | 0.011 | 0.0020 | 425 | 1234 | 476 | 595 |
| 0.079 | 0.22 | 1.14 | 0.013 | 0.004 | 0.152 | 0.001 | 0.077 | 0.042 | 0.006 | 0.049 | 0.039 | 0.016 | 0.0005 | 432 | 1221 | 546 | 651 |
| 0.079 | 0.22 | 1.47 | 0.009 | 0.002 | 0.163 | 0.001 | 0.072 | 0.074 | 0.006 | 0.038 | 0.042 | 0.016 | 0.0019 | 430 | 1218 | 483 | 616 |
| 0.079 | 0.25 | 1.32 | 0.010 | 0.004 | 0.172 | 0.001 | 0.053 | 0.112 | 0.006 | 0.048 | 0.039 | 0.012 | 0.0028 | 423 | 1135 | 514 | 622 |
| 0.079 | 0.22 | 1.16 | 0.009 | 0.003 | 0.164 | 0.001 | 0.017 | 0.098 | 0.005 | 0.048 | 0.041 | 0.016 | 0.0027 | 452 | 1215 | 535 | 631 |
| 0.078 | 0.22 | 1.16 | 0.013 | 0.004 | 0.161 | 0.001 | 0.068 | 0.079 | 0.004 | 0.024 | 0.037 | 0.016 | 0.0027 | 428 | 1237 | 533 | 644 |
| 0.078 | 0.22 | 1.53 | 0.010 | 0.003 | 0.164 | 0.001 | 0.038 | 0.074 | 0.002 | 0.020 | 0.037 | 0.016 | 0.0016 | 392 | 1215 | 487 | 608 |
| 0.079 | 0.21 | 1.43 | 0.009 | 0.004 | 0.163 | 0.001 | 0.072 | 0.044 | 0.006 | 0.018 | 0.040 | 0.016 | 0.0016 | 425 | 1215 | 517 | 638 |
| 0.079 | 0.22 | 1.15 | 0.010 | 0.003 | 0.164 | 0.001 | 0.078 | 0.103 | 0.005 | 0.030 | 0.042 | 0.017 | 0.0028 | 454 | 1271 | 516 | 638 |
| 0.079 | 0.22 | 1.43 | 0.012 | 0.004 | 0.172 | 0.001 | 0.060 | 0.110 | 0.003 | 0.017 | 0.041 | 0.016 | 0.0025 | 430 | 1211 | 491 | 622 |
| 0.079 | 0.22 | 1.16 | 0.009 | 0.002 | 0.167 | 0.001 | 0.092 | 0.073 | 0.006 | 0.026 | 0.037 | 0.016 | 0.0017 | 258 | 1208 | 508 | 584 |
| 0.078 | 0.23 | 1.58 | 0.013 | 0.003 | 0.167 | 0.002 | 0.044 | 0.092 | 0.003 | 0.049 | 0.038 | 0.016 | 0.0025 | 426 | 1216 | 499 | 623 |
| 0.079 | 0.24 | 1.27 | 0.012 | 0.002 | 0.156 | 0.001 | 0.060 | 0.106 | 0.002 | 0.049 | 0.041 | 0.016 | 0.0012 | 433 | 1250 | 503 | 615 |
| 0.079 | 0.22 | 1.16 | 0.009 | 0.002 | 0.165 | 0.001 | 0.139 | 0.064 | 0.002 | 0.048 | 0.038 | 0.016 | 0.0027 | 454 | 1240 | 538 | 626 |
| 0.079 | 0.22 | 1.41 | 0.013 | 0.003 | 0.162 | 0.001 | 0.107 | 0.043 | 0.005 | 0.019 | 0.039 | 0.016 | 0.0025 | 432 | 1260 | 483 | 620 |
| 0.079 | 0.23 | 1.16 | 0.009 | 0.002 | 0.168 | 0.001 | 0.123 | 0.107 | 0.006 | 0.020 | 0.040 | 0.016 | 0.0027 | 433 | 1235 | 523 | 622 |
| 0.079 | 0.22 | 1.17 | 0.011 | 0.004 | 0.162 | 0.001 | 0.047 | 0.032 | 0.005 | 0.021 | 0.036 | 0.016 | 0.0027 | 432 | 1213 | 537 | 649 |
| 0.081 | 0.30 | 1.44 | 0.012 | 0.003 | 0.162 | 0.001 | 0.067 | 0.109 | 0.006 | 0.049 | 0.037 | 0.016 | 0.0020 | 451 | 1217 | 478 | 620 |
| 0.081 | 0.24 | 1.48 | 0.013 | 0.003 | 0.164 | 0.001 | 0.051 | 0.084 | 0.002 | 0.049 | 0.041 | 0.012 | 0.0026 | 430 | 1216 | 482 | 624 |
| 0.079 | 0.30 | 1.43 | 0.009 | 0.004 | 0.057 | 0.002 | 0.015 | 0.088 | 0.001 | 0.025 | 0.038 | 0.016 | 0.0016 | 396 | 1157 | 500 | 606 |
| 0.079 | 0.22 | 1.45 | 0.012 | 0.003 | 0.170 | 0.001 | 0.067 | 0.091 | 0.003 | 0.017 | 0.041 | 0.016 | 0.0017 | 437 | 1216 | 486 | 620 |
| 0.079 | 0.22 | 1.17 | 0.010 | 0.003 | 0.163 | 0.001 | 0.065 | 0.108 | 0.005 | 0.048 | 0.042 | 0.016 | 0.0027 | 424 | 1276 | 526 | 642 |
| 0.079 | 0.21 | 1.43 | 0.009 | 0.002 | 0.162 | 0.001 | 0.114 | 0.090 | 0.005 | 0.036 | 0.036 | 0.016 | 0.0017 | 438 | 1222 | 485 | 613 |
| 0.082 | 0.30 | 1.50 | 0.013 | 0.003 | 0.049 | 0.001 | 0.041 | 0.086 | 0.008 | 0.049 | 0.038 | 0.013 | 0.0027 | 433 | 1216 | 478 | 614 |
| 0.080 | 0.25 | 1.28 | 0.013 | 0.001 | 0.143 | 0.002 | 0.065 | 0.036 | 0.007 | 0.049 | 0.034 | 0.013 | 0.0028 | 460 | 1243 | 498 | 606 |
| 0.078 | 0.23 | 1.54 | 0.012 | 0.004 | 0.103 | 0.002 | 0.055 | 0.088 | 0.005 | 0.021 | 0.037 | 0.016 | 0.0026 | 429 | 1212 | 524 | 622 |
| 0.079 | 0.22 | 1.15 | 0.012 | 0.002 | 0.164 | 0.001 | 0.048 | 0.039 | 0.006 | 0.049 | 0.037 | 0.016 | 0.0013 | 428 | 1221 | 529 | 629 |
| 0.078 | 0.22 | 1.16 | 0.013 | 0.002 | 0.162 | 0.001 | 0.152 | 0.049 | 0.005 | 0.049 | 0.038 | 0.016 | 0.0002 | 444 | 1236 | 528 | 633 |
| 0.079 | 0.22 | 1.15 | 0.010 | 0.002 | 0.162 | 0.001 | 0.059 | 0.089 | 0.006 | 0.021 | 0.036 | 0.016 | 0.0027 | 429 | 1271 | 517 | 637 |
| 0.079 | 0.24 | 1.53 | 0.010 | 0.001 | 0.138 | 0.002 | 0.101 | 0.096 | 0.007 | 0.048 | 0.042 | 0.016 | 0.0027 | 424 | 1252 | 506 | 599 |
| 0.079 | 0.30 | 1.43 | 0.010 | 0.004 | 0.162 | 0.001 | 0.058 | 0.047 | 0.002 | 0.028 | 0.039 | 0.016 | 0.0017 | 425 | 1215 | 473 | 602 |
| 0.079 | 0.22 | 1.14 | 0.009 | 0.003 | 0.167 | 0.001 | 0.062 | 0.077 | 0.005 | 0.040 | 0.042 | 0.016 | 0.0002 | 454 | 1273 | 513 | 635 |
| 0.079 | 0.23 | 1.16 | 0.012 | 0.004 | 0.171 | 0.001 | 0.058 | 0.118 | 0.002 | 0.020 | 0.036 | 0.016 | 0.0027 | 430 | 1273 | 542 | 655 |
| 0.081 | 0.29 | 1.29 | 0.010 | 0.001 | 0.172 | 0.001 | 0.076 | 0.062 | 0.003 | 0.048 | 0.040 | 0.011 | 0.0029 | 457 | 1244 | 509 | 610 |
| 0.079 | 0.24 | 1.16 | 0.013 | 0.002 | 0.081 | 0.002 | 0.067 | 0.035 | 0.004 | 0.048 | 0.039 | 0.012 | 0.0027 | 452 | 1233 | 520 | 614 |
| 0.079 | 0.24 | 1.16 | 0.010 | 0.002 | 0.162 | 0.002 | 0.065 | 0.050 | 0.006 | 0.037 | 0.039 | 0.016 | 0.0027 | 438 | 1271 | 510 | 640 |
| 0.079 | 0.24 | 1.23 | 0.013 | 0.002 | 0.164 | 0.002 | 0.068 | 0.077 | 0.006 | 0.047 | 0.038 | 0.016 | 0.0002 | 451 | 1222 | 502 | 615 |
| 0.082 | 0.24 | 1.45 | 0.009 | 0.004 | 0.101 | 0.001 | 0.010 | 0.029 | 0.006 | 0.036 | 0.041 | 0.017 | 0.0027 | 457 | 1213 | 513 | 629 |
| 0.079 | 0.22 | 1.45 | 0.012 | 0.004 | 0.162 | 0.001 | 0.039 | 0.042 | 0.000 | 0.020 | 0.039 | 0.016 | 0.0020 | 457 | 1215 | 515 | 640 |
| 0.082 | 0.23 | 1.47 | 0.009 | 0.001 | 0.161 | 0.001 | 0.051 | 0.098 | 0.005 | 0.037 | 0.037 | 0.017 | 0.0012 | 425 | 1216 | 471 | 617 |
| 0.079 | 0.21 | 1.15 | 0.009 | 0.002 | 0.169 | 0.001 | 0.052 | 0.026 | 0.005 | 0.019 | 0.038 | 0.016 | 0.0027 | 428 | 1272 | 526 | 631 |
| 0.079 | 0.22 | 1.43 | 0.009 | 0.003 | 0.162 | 0.001 | 0.069 | 0.082 | 0.005 | 0.020 | 0.036 | 0.016 | 0.0027 | 423 | 1222 | 497 | 620 |
| 0.078 | 0.22 | 1.17 | 0.012 | 0.002 | 0.026 | 0.001 | 0.055 | 0.086 | 0.006 | 0.048 | 0.040 | 0.016 | 0.0027 | 436 | 1275 | 529 | 622 |
| 0.079 | 0.22 | 1.16 | 0.010 | 0.002 | 0.166 | 0.001 | 0.068 | 0.086 | 0.005 | 0.048 | 0.037 | 0.016 | 0.0026 | 438 | 1230 | 528 | 633 |
| 0.077 | 0.23 | 1.16 | 0.009 | 0.004 | 0.163 | 0.002 | 0.076 | 0.079 | 0.006 | 0.021 | 0.037 | 0.016 | 0.0027 | 428 | 1233 | 546 | 633 |
| 0.079 | 0.23 | 1.15 | 0.012 | 0.004 | 0.114 | 0.001 | 0.072 | 0.115 | 0.003 | 0.020 | 0.036 | 0.016 | 0.0027 | 426 | 1239 | 534 | 633 |
| 0.079 | 0.23 | 1.47 | 0.010 | 0.004 | 0.084 | 0.001 | 0.072 | 0.108 | 0.007 | 0.037 | 0.037 | 0.016 | 0.0027 | 437 | 1215 | 515 | 628 |
| 0.078 | 0.24 | 1.58 | 0.013 | 0.004 | 0.125 | 0.002 | 0.094 | 0.096 | 0.005 | 0.049 | 0.038 | 0.014 | 0.0003 | 354 | 1027 | 525 | 629 |
| 0.079 | 0.22 | 1.45 | 0.010 | 0.004 | 0.162 | 0.001 | 0.073 | 0.089 | 0.004 | 0.042 | 0.041 | 0.016 | 0.0017 | 425 | 1214 | 508 | 640 |
| 0.078 | 0.22 | 1.17 | 0.010 | 0.002 | 0.162 | 0.002 | 0.076 | 0.073 | 0.007 | 0.020 | 0.040 | 0.016 | 0.0028 | 452 | 1253 | 523 | 618 |
| 0.079 | 0.23 | 1.16 | 0.009 | 0.003 | 0.095 | 0.001 | 0.060 | 0.074 | 0.005 | 0.048 | 0.042 | 0.016 | 0.0013 | 426 | 1259 | 521 | 604 |
| 0.079 | 0.22 | 1.15 | 0.013 | 0.003 | 0.161 | 0.001 | 0.072 | 0.055 | 0.005 | 0.048 | 0.038 | 0.016 | 0.0010 | 404 | 1271 | 517 | 631 |
| 0.079 | 0.21 | 1.15 | 0.013 | 0.002 | 0.051 | 0.002 | 0.020 | 0.049 | 0.006 | 0.049 | 0.042 | 0.016 | 0.0027 | 452 | 1239 | 544 | 625 |
| 0.082 | 0.24 | 1.54 | 0.013 | 0.004 | 0.168 | 0.002 | 0.109 | 0.071 | 0.002 | 0.044 | 0.040 | 0.011 | 0.0027 | 452 | 1221 | 527 | 615 |
| 0.079 | 0.22 | 1.52 | 0.009 | 0.004 | 0.098 | 0.001 | 0.074 | 0.062 | 0.005 | 0.035 | 0.042 | 0.016 | 0.0013 | 454 | 1214 | 522 | 621 |
| 0.078 | 0.24 | 1.16 | 0.012 | 0.002 | 0.037 | 0.002 | 0.033 | 0.083 | 0.005 | 0.049 | 0.042 | 0.011 | 0.0027 | 436 | 1233 | 524 | 623 |
| 0.083 | 0.32 | 1.48 | 0.012 | 0.001 | 0.174 | 0.001 | 0.064 | 0.055 | 0.005 | 0.036 | 0.037 | 0.016 | 0.0026 | 352 | 1220 | 498 | 597 |
| 0.082 | 0.22 | 1.50 | 0.009 | 0.001 | 0.172 | 0.001 | 0.078 | 0.138 | 0.006 | 0.037 | 0.040 | 0.016 | 0.0027 | 452 | 1221 | 497 | 618 |
| 0.079 | 0.29 | 1.45 | 0.012 | 0.004 | 0.086 | 0.001 | 0.026 | 0.103 | 0.005 | 0.049 | 0.036 | 0.013 | 0.0016 | 459 | 1165 | 518 | 635 |
| 0.079 | 0.22 | 1.16 | 0.010 | 0.003 | 0.168 | 0.002 | 0.073 | 0.060 | 0.005 | 0.036 | 0.038 | 0.016 | 0.0028 | 455 | 1234 | 525 | 626 |
| 0.079 | 0.30 | 1.15 | 0.012 | 0.003 | 0.163 | 0.002 | 0.075 | 0.081 | 0.007 | 0.049 | 0.038 | 0.014 | 0.0019 | 455 | 1232 | 509 | 622 |
| 0.079 | 0.22 | 1.30 | 0.009 | 0.002 | 0.139 | 0.001 | 0.070 | 0.057 | 0.005 | 0.048 | 0.036 | 0.016 | 0.0027 | 433 | 1233 | 520 | 596 |
| 0.079 | 0.25 | 1.15 | 0.012 | 0.002 | 0.164 | 0.002 | 0.053 | 0.089 | 0.006 | 0.049 | 0.042 | 0.016 | 0.0017 | 454 | 1217 | 520 | 620 |
| 0.079 | 0.24 | 1.15 | 0.014 | 0.002 | 0.142 | 0.002 | 0.070 | 0.064 | 0.007 | 0.049 | 0.040 | 0.016 | 0.0028 | 442 | 1233 | 531 | 633 |
| 0.078 | 0.22 | 1.17 | 0.010 | 0.002 | 0.162 | 0.002 | 0.065 | 0.095 | 0.006 | 0.036 | 0.042 | 0.016 | 0.0027 | 435 | 1271 | 511 | 633 |
| 0.079 | 0.24 | 1.54 | 0.014 | 0.002 | 0.169 | 0.002 | 0.071 | 0.092 | 0.006 | 0.038 | 0.042 | 0.016 | 0.0019 | 432 | 1221 | 487 | 611 |
| 0.081 | 0.24 | 1.53 | 0.008 | 0.001 | 0.172 | 0.001 | 0.022 | 0.046 | 0.005 | 0.020 | 0.040 | 0.016 | 0.0026 | 414 | 1215 | 496 | 610 |
| 0.079 | 0.22 | 1.15 | 0.012 | 0.002 | 0.158 | 0.001 | 0.058 | 0.075 | 0.006 | 0.048 | 0.038 | 0.016 | 0.0009 | 350 | 1271 | 521 | 616 |
| 0.078 | 0.24 | 1.15 | 0.009 | 0.002 | 0.064 | 0.002 | 0.071 | 0.100 | 0.005 | 0.048 | 0.038 | 0.016 | 0.0028 | 416 | 1271 | 520 | 620 |
| 0.079 | 0.24 | 1.24 | 0.009 | 0.002 | 0.164 | 0.001 | 0.061 | 0.084 | 0.005 | 0.020 | 0.036 | 0.016 | 0.0027 | 431 | 1233 | 502 | 614 |
| 0.079 | 0.22 | 1.45 | 0.012 | 0.004 | 0.163 | 0.002 | 0.080 | 0.086 | 0.001 | 0.032 | 0.037 | 0.016 | 0.0016 | 397 | 1169 | 504 | 629 |
| 0.079 | 0.22 | 1.51 | 0.010 | 0.004 | 0.163 | 0.001 | 0.066 | 0.053 | 0.005 | 0.021 | 0.039 | 0.016 | 0.0021 | 436 | 1192 | 504 | 638 |
| 0.079 | 0.22 | 1.16 | 0.009 | 0.002 | 0.172 | 0.001 | 0.075 | 0.071 | 0.001 | 0.041 | 0.042 | 0.016 | 0.0027 | 428 | 1238 | 519 | 629 |
| 0.079 | 0.23 | 1.16 | 0.013 | 0.002 | 0.164 | 0.001 | 0.070 | 0.091 | 0.003 | 0.048 | 0.037 | 0.016 | 0.0005 | 451 | 1258 | 520 | 621 |
| 0.082 | 0.22 | 1.48 | 0.009 | 0.001 | 0.163 | 0.001 | 0.070 | 0.147 | 0.002 | 0.018 | 0.039 | 0.016 | 0.0010 | 291 | 1214 | 485 | 612 |
| 0.079 | 0.22 | 1.26 | 0.009 | 0.002 | 0.162 | 0.001 | 0.073 | 0.060 | 0.007 | 0.036 | 0.036 | 0.016 | 0.0027 | 458 | 1244 | 503 | 614 |
| 0.079 | 0.22 | 1.15 | 0.010 | 0.002 | 0.163 | 0.001 | 0.078 | 0.072 | 0.007 | 0.030 | 0.039 | 0.016 | 0.0007 | 329 | 1215 | 506 | 598 |
| 0.079 | 0.23 | 1.44 | 0.009 | 0.004 | 0.163 | 0.001 | 0.060 | 0.084 | 0.007 | 0.035 | 0.039 | 0.016 | 0.0008 | 425 | 1209 | 512 | 642 |
| 0.078 | 0.22 | 1.16 | 0.013 | 0.003 | 0.168 | 0.001 | 0.062 | 0.098 | 0.007 | 0.049 | 0.040 | 0.016 | 0.0002 | 454 | 1271 | 525 | 651 |
| 0.079 | 0.22 | 1.16 | 0.013 | 0.002 | 0.154 | 0.003 | 0.076 | 0.047 | 0.008 | 0.049 | 0.042 | 0.014 | 0.0018 | 456 | 1267 | 520 | 612 |
| 0.078 | 0.22 | 1.16 | 0.009 | 0.002 | 0.161 | 0.001 | 0.070 | 0.058 | 0.005 | 0.041 | 0.036 | 0.016 | 0.0027 | 424 | 1274 | 516 | 633 |
| 0.079 | 0.24 | 1.16 | 0.010 | 0.002 | 0.168 | 0.002 | 0.079 | 0.065 | 0.007 | 0.037 | 0.036 | 0.016 | 0.0028 | 459 | 1232 | 519 | 631 |
| 0.078 | 0.22 | 1.16 | 0.009 | 0.004 | 0.167 | 0.001 | 0.062 | 0.063 | 0.005 | 0.035 | 0.040 | 0.016 | 0.0011 | 432 | 1271 | 528 | 646 |
| 0.079 | 0.24 | 1.47 | 0.012 | 0.004 | 0.155 | 0.001 | 0.076 | 0.051 | 0.004 | 0.022 | 0.039 | 0.016 | 0.0009 | 350 | 1029 | 495 | 624 |
| 0.081 | 0.29 | 1.17 | 0.009 | 0.001 | 0.162 | 0.002 | 0.070 | 0.071 | 0.006 | 0.037 | 0.040 | 0.016 | 0.0027 | 424 | 1241 | 501 | 614 |
| 0.079 | 0.30 | 1.53 | 0.013 | 0.004 | 0.056 | 0.001 | 0.067 | 0.039 | 0.002 | 0.049 | 0.036 | 0.015 | 0.0009 | 342 | 983 | 518 | 633 |
| 0.081 | 0.22 | 1.44 | 0.012 | 0.004 | 0.162 | 0.001 | 0.056 | 0.062 | 0.004 | 0.035 | 0.038 | 0.011 | 0.0027 | 456 | 1212 | 522 | 625 |
| 0.079 | 0.31 | 1.25 | 0.012 | 0.002 | 0.112 | 0.001 | 0.003 | 0.105 | 0.006 | 0.049 | 0.039 | 0.013 | 0.0028 | 447 | 1217 | 510 | 600 |
| 0.078 | 0.22 | 1.15 | 0.013 | 0.002 | 0.150 | 0.002 | 0.076 | 0.079 | 0.006 | 0.049 | 0.039 | 0.016 | 0.0015 | 435 | 1262 | 527 | 623 |
| 0.078 | 0.29 | 1.15 | 0.013 | 0.002 | 0.169 | 0.002 | 0.077 | 0.125 | 0.006 | 0.049 | 0.036 | 0.012 | 0.0027 | 454 | 1262 | 529 | 631 |
| 0.079 | 0.24 | 1.26 | 0.012 | 0.002 | 0.036 | 0.001 | 0.068 | 0.082 | 0.006 | 0.048 | 0.039 | 0.016 | 0.0026 | 437 | 1231 | 505 | 590 |
| 0.078 | 0.22 | 1.15 | 0.010 | 0.002 | 0.164 | 0.001 | 0.073 | 0.091 | 0.003 | 0.019 | 0.042 | 0.016 | 0.0005 | 424 | 1273 | 505 | 623 |
| 0.079 | 0.29 | 1.58 | 0.013 | 0.004 | 0.144 | 0.002 | 0.092 | 0.064 | 0.007 | 0.049 | 0.038 | 0.016 | 0.0020 | 343 | 1259 | 539 | 617 |
| 0.079 | 0.22 | 1.15 | 0.010 | 0.003 | 0.165 | 0.002 | 0.041 | 0.106 | 0.007 | 0.038 | 0.042 | 0.016 | 0.0002 | 455 | 1263 | 515 | 629 |
| 0.079 | 0.24 | 1.17 | 0.010 | 0.002 | 0.161 | 0.002 | 0.077 | 0.062 | 0.005 | 0.040 | 0.036 | 0.016 | 0.0027 | 455 | 1233 | 520 | 629 |
| 0.078 | 0.22 | 1.15 | 0.013 | 0.002 | 0.163 | 0.001 | 0.043 | 0.062 | 0.005 | 0.049 | 0.038 | 0.016 | 0.0027 | 458 | 1221 | 540 | 635 |
| 0.082 | 0.25 | 1.29 | 0.013 | 0.002 | 0.074 | 0.001 | 0.040 | 0.087 | 0.006 | 0.049 | 0.039 | 0.013 | 0.0002 | 432 | 1259 | 492 | 602 |
| 0.073 | 0.22 | 1.58 | 0.013 | 0.003 | 0.166 | 0.001 | 0.049 | 0.075 | 0.004 | 0.019 | 0.038 | 0.013 | 0.0028 | 414 | 1216 | 511 | 590 |
| 0.079 | 0.29 | 1.55 | 0.012 | 0.004 | 0.112 | 0.001 | 0.018 | 0.029 | 0.005 | 0.028 | 0.038 | 0.012 | 0.0016 | 423 | 1156 | 506 | 608 |
| 0.081 | 0.24 | 1.29 | 0.009 | 0.002 | 0.091 | 0.001 | 0.066 | 0.052 | 0.002 | 0.048 | 0.037 | 0.012 | 0.0009 | 429 | 1245 | 489 | 592 |
| 0.079 | 0.23 | 1.45 | 0.012 | 0.004 | 0.049 | 0.001 | 0.034 | 0.098 | 0.005 | 0.049 | 0.040 | 0.016 | 0.0019 | 432 | 1209 | 514 | 642 |
| 0.078 | 0.21 | 1.15 | 0.009 | 0.003 | 0.161 | 0.001 | 0.058 | 0.048 | 0.005 | 0.035 | 0.038 | 0.016 | 0.0027 | 433 | 1216 | 510 | 626 |
| 0.081 | 0.23 | 1.54 | 0.009 | 0.001 | 0.173 | 0.002 | 0.067 | 0.075 | 0.008 | 0.048 | 0.039 | 0.016 | 0.0020 | 426 | 1217 | 484 | 624 |
| 0.078 | 0.31 | 1.57 | 0.012 | 0.004 | 0.169 | 0.001 | 0.057 | 0.082 | 0.001 | 0.019 | 0.038 | 0.016 | 0.0019 | 353 | 1170 | 534 | 618 |
| 0.078 | 0.22 | 1.17 | 0.008 | 0.002 | 0.168 | 0.001 | 0.023 | 0.050 | 0.002 | 0.027 | 0.041 | 0.016 | 0.0027 | 455 | 1211 | 525 | 619 |
| 0.082 | 0.24 | 1.29 | 0.012 | 0.001 | 0.048 | 0.001 | 0.075 | 0.100 | 0.008 | 0.049 | 0.036 | 0.016 | 0.0016 | 461 | 1245 | 515 | 612 |
| 0.082 | 0.24 | 1.42 | 0.010 | 0.004 | 0.142 | 0.001 | 0.032 | 0.104 | 0.006 | 0.021 | 0.040 | 0.016 | 0.0027 | 424 | 1220 | 510 | 631 |
| 0.078 | 0.24 | 1.15 | 0.011 | 0.002 | 0.173 | 0.001 | 0.019 | 0.085 | 0.009 | 0.036 | 0.037 | 0.016 | 0.0028 | 431 | 1216 | 506 | 628 |
| 0.076 | 0.21 | 1.15 | 0.013 | 0.003 | 0.045 | 0.001 | 0.065 | 0.071 | 0.005 | 0.048 | 0.042 | 0.016 | 0.0027 | 425 | 1242 | 529 | 606 |
| 0.078 | 0.22 | 1.14 | 0.012 | 0.004 | 0.159 | 0.001 | 0.145 | 0.124 | 0.002 | 0.047 | 0.040 | 0.016 | 0.0013 | 417 | 1261 | 543 | 633 |
| 0.078 | 0.22 | 1.15 | 0.012 | 0.004 | 0.157 | 0.002 | 0.066 | 0.061 | 0.003 | 0.020 | 0.038 | 0.012 | 0.0002 | 459 | 1233 | 531 | 616 |
| 0.082 | 0.24 | 1.15 | 0.009 | 0.001 | 0.164 | 0.002 | 0.042 | 0.109 | 0.005 | 0.033 | 0.038 | 0.012 | 0.0029 | 430 | 1271 | 506 | 622 |
| 0.079 | 0.22 | 1.44 | 0.013 | 0.004 | 0.153 | 0.002 | 0.121 | 0.145 | 0.004 | 0.041 | 0.047 | 0.016 | 0.0019 | 427 | 1169 | 508 | 629 |
| 0.079 | 0.30 | 1.57 | 0.012 | 0.004 | 0.168 | 0.001 | 0.072 | 0.058 | 0.004 | 0.025 | 0.039 | 0.012 | 0.0016 | 422 | 1142 | 516 | 614 |
| 0.079 | 0.24 | 1.47 | 0.013 | 0.004 | 0.162 | 0.002 | 0.075 | 0.026 | 0.005 | 0.026 | 0.039 | 0.016 | 0.0027 | 432 | 1220 | 518 | 637 |
| 0.079 | 0.22 | 1.54 | 0.010 | 0.004 | 0.164 | 0.001 | 0.068 | 0.088 | 0.005 | 0.020 | 0.042 | 0.016 | 0.0007 | 426 | 1215 | 520 | 638 |
| 0.079 | 0.24 | 1.14 | 0.014 | 0.002 | 0.081 | 0.002 | 0.074 | 0.117 | 0.005 | 0.037 | 0.042 | 0.016 | 0.0028 | 453 | 1231 | 506 | 617 |
| 0.079 | 0.22 | 1.17 | 0.010 | 0.003 | 0.168 | 0.001 | 0.064 | 0.073 | 0.005 | 0.021 | 0.036 | 0.016 | 0.0018 | 424 | 1218 | 504 | 621 |
| 0.079 | 0.22 | 1.15 | 0.010 | 0.002 | 0.164 | 0.001 | 0.029 | 0.079 | 0.006 | 0.020 | 0.039 | 0.016 | 0.0028 | 444 | 1269 | 518 | 629 |
| 0.079 | 0.30 | 1.53 | 0.012 | 0.004 | 0.069 | 0.001 | 0.079 | 0.094 | 0.005 | 0.019 | 0.038 | 0.011 | 0.0027 | 428 | 1179 | 527 | 611 |
| 0.079 | 0.21 | 1.15 | 0.009 | 0.003 | 0.163 | 0.001 | 0.064 | 0.064 | 0.005 | 0.019 | 0.042 | 0.016 | 0.0027 | 428 | 1263 | 519 | 629 |
| 0.079 | 0.24 | 1.16 | 0.014 | 0.001 | 0.111 | 0.002 | 0.057 | 0.061 | 0.003 | 0.048 | 0.039 | 0.016 | 0.0027 | 292 | 1208 | 517 | 608 |
| 0.079 | 0.24 | 1.53 | 0.010 | 0.004 | 0.065 | 0.001 | 0.071 | 0.074 | 0.007 | 0.035 | 0.042 | 0.016 | 0.0027 | 407 | 1210 | 522 | 627 |
| 0.078 | 0.24 | 1.45 | 0.013 | 0.004 | 0.071 | 0.002 | 0.067 | 0.067 | 0.005 | 0.049 | 0.040 | 0.012 | 0.0006 | 383 | 1026 | 500 | 622 |
| 0.079 | 0.22 | 1.45 | 0.010 | 0.004 | 0.163 | 0.001 | 0.064 | 0.055 | 0.005 | 0.047 | 0.037 | 0.016 | 0.0008 | 457 | 1210 | 522 | 644 |
| 0.079 | 0.22 | 1.16 | 0.009 | 0.003 | 0.162 | 0.001 | 0.022 | 0.054 | 0.007 | 0.048 | 0.042 | 0.016 | 0.0027 | 436 | 1244 | 522 | 635 |
| 0.079 | 0.22 | 1.15 | 0.009 | 0.002 | 0.161 | 0.002 | 0.055 | 0.076 | 0.005 | 0.039 | 0.038 | 0.016 | 0.0011 | 432 | 1238 | 511 | 615 |
| 0.079 | 0.22 | 1.44 | 0.013 | 0.003 | 0.165 | 0.001 | 0.075 | 0.099 | 0.005 | 0.047 | 0.038 | 0.016 | 0.0017 | 426 | 1247 | 478 | 618 |
| 0.079 | 0.24 | 1.40 | 0.009 | 0.004 | 0.165 | 0.001 | 0.093 | 0.043 | 0.003 | 0.026 | 0.042 | 0.011 | 0.0017 | 442 | 1246 | 500 | 622 |
| 0.079 | 0.22 | 1.15 | 0.013 | 0.003 | 0.143 | 0.002 | 0.046 | 0.072 | 0.005 | 0.049 | 0.040 | 0.016 | 0.0006 | 423 | 1272 | 520 | 629 |
| 0.079 | 0.21 | 1.16 | 0.010 | 0.002 | 0.168 | 0.001 | 0.066 | 0.087 | 0.007 | 0.020 | 0.036 | 0.016 | 0.0019 | 429 | 1235 | 506 | 619 |
| 0.079 | 0.29 | 1.15 | 0.010 | 0.001 | 0.166 | 0.002 | 0.060 | 0.093 | 0.005 | 0.048 | 0.041 | 0.016 | 0.0026 | 434 | 1232 | 525 | 620 |
| 0.078 | 0.22 | 1.16 | 0.014 | 0.002 | 0.018 | 0.001 | 0.073 | 0.120 | 0.005 | 0.050 | 0.036 | 0.016 | 0.0002 | 455 | 1261 | 534 | 628 |
| 0.079 | 0.22 | 1.14 | 0.013 | 0.004 | 0.044 | 0.001 | 0.055 | 0.043 | 0.002 | 0.020 | 0.042 | 0.016 | 0.0027 | 458 | 1245 | 539 | 623 |
| 0.079 | 0.23 | 1.15 | 0.010 | 0.002 | 0.154 | 0.002 | 0.062 | 0.093 | 0.007 | 0.020 | 0.038 | 0.016 | 0.0027 | 453 | 1262 | 518 | 624 |
| 0.079 | 0.31 | 1.54 | 0.013 | 0.004 | 0.156 | 0.002 | 0.013 | 0.103 | 0.003 | 0.037 | 0.039 | 0.011 | 0.0027 | 381 | 984 | 499 | 616 |
| 0.078 | 0.22 | 1.15 | 0.013 | 0.002 | 0.163 | 0.002 | 0.043 | 0.037 | 0.005 | 0.024 | 0.040 | 0.016 | 0.0002 | 426 | 1270 | 501 | 624 |
| 0.078 | 0.21 | 1.16 | 0.010 | 0.004 | 0.166 | 0.002 | 0.051 | 0.101 | 0.002 | 0.020 | 0.037 | 0.016 | 0.0017 | 444 | 1273 | 528 | 646 |
| 0.081 | 0.25 | 1.27 | 0.013 | 0.001 | 0.162 | 0.001 | 0.073 | 0.049 | 0.006 | 0.049 | 0.036 | 0.012 | 0.0028 | 453 | 1232 | 513 | 619 |
| 0.079 | 0.22 | 1.16 | 0.010 | 0.002 | 0.168 | 0.001 | 0.066 | 0.083 | 0.005 | 0.036 | 0.037 | 0.016 | 0.0027 | 424 | 1240 | 517 | 628 |
| 0.079 | 0.22 | 1.15 | 0.014 | 0.002 | 0.164 | 0.002 | 0.067 | 0.088 | 0.007 | 0.050 | 0.042 | 0.016 | 0.0017 | 454 | 1236 | 525 | 635 |
| 0.079 | 0.22 | 1.15 | 0.012 | 0.003 | 0.091 | 0.001 | 0.077 | 0.058 | 0.003 | 0.048 | 0.038 | 0.016 | 0.0002 | 427 | 1261 | 519 | 624 |
| 0.078 | 0.22 | 1.29 | 0.010 | 0.002 | 0.139 | 0.001 | 0.067 | 0.095 | 0.007 | 0.048 | 0.037 | 0.016 | 0.0027 | 450 | 1215 | 521 | 597 |
| 0.073 | 0.22 | 1.15 | 0.010 | 0.003 | 0.170 | 0.001 | 0.019 | 0.056 | 0.005 | 0.020 | 0.038 | 0.016 | 0.0028 | 455 | 1241 | 523 | 612 |
| 0.079 | 0.22 | 1.43 | 0.009 | 0.003 | 0.172 | 0.001 | 0.032 | 0.073 | 0.005 | 0.020 | 0.038 | 0.016 | 0.0027 | 442 | 1216 | 500 | 624 |
| 0.082 | 0.29 | 1.54 | 0.012 | 0.001 | 0.174 | 0.001 | 0.139 | 0.069 | 0.004 | 0.019 | 0.045 | 0.011 | 0.0027 | 461 | 1237 | 509 | 598 |
| 0.079 | 0.22 | 1.16 | 0.010 | 0.002 | 0.162 | 0.001 | 0.077 | 0.105 | 0.006 | 0.037 | 0.041 | 0.016 | 0.0026 | 461 | 1244 | 522 | 628 |
| 0.078 | 0.22 | 1.15 | 0.013 | 0.003 | 0.171 | 0.001 | 0.047 | 0.095 | 0.003 | 0.049 | 0.042 | 0.016 | 0.0027 | 452 | 1233 | 535 | 637 |
| 0.079 | 0.22 | 1.45 | 0.013 | 0.003 | 0.022 | 0.001 | 0.066 | 0.088 | 0.008 | 0.049 | 0.041 | 0.016 | 0.0007 | 452 | 1219 | 502 | 614 |
| 0.078 | 0.22 | 1.54 | 0.010 | 0.004 | 0.163 | 0.001 | 0.075 | 0.060 | 0.006 | 0.048 | 0.036 | 0.016 | 0.0017 | 431 | 1222 | 513 | 644 |
| 0.079 | 0.23 | 1.53 | 0.013 | 0.004 | 0.169 | 0.001 | 0.043 | 0.104 | 0.003 | 0.018 | 0.036 | 0.016 | 0.0016 | 403 | 1191 | 506 | 637 |
| 0.082 | 0.23 | 1.31 | 0.012 | 0.002 | 0.082 | 0.001 | 0.047 | 0.139 | 0.005 | 0.049 | 0.041 | 0.011 | 0.0002 | 456 | 1231 | 509 | 615 |
| 0.079 | 0.22 | 1.17 | 0.010 | 0.002 | 0.162 | 0.001 | 0.028 | 0.127 | 0.008 | 0.048 | 0.035 | 0.016 | 0.0028 | 443 | 1234 | 532 | 629 |
| 0.079 | 0.22 | 1.17 | 0.010 | 0.003 | 0.172 | 0.001 | 0.079 | 0.093 | 0.005 | 0.019 | 0.036 | 0.016 | 0.0027 | 452 | 1233 | 524 | 633 |
| 0.079 | 0.24 | 1.16 | 0.012 | 0.004 | 0.168 | 0.003 | 0.075 | 0.066 | 0.001 | 0.035 | 0.041 | 0.016 | 0.0020 | 451 | 1274 | 523 | 646 |
| 0.079 | 0.22 | 1.42 | 0.012 | 0.004 | 0.066 | 0.001 | 0.052 | 0.066 | 0.003 | 0.048 | 0.037 | 0.016 | 0.0027 | 427 | 1210 | 524 | 635 |
| 0.079 | 0.23 | 1.52 | 0.010 | 0.004 | 0.168 | 0.002 | 0.102 | 0.076 | 0.004 | 0.021 | 0.037 | 0.016 | 0.0014 | 417 | 1230 | 510 | 627 |
| 0.079 | 0.24 | 1.16 | 0.012 | 0.002 | 0.168 | 0.002 | 0.046 | 0.056 | 0.007 | 0.050 | 0.036 | 0.016 | 0.0021 | 418 | 1251 | 519 | 635 |
| 0.078 | 0.22 | 1.42 | 0.009 | 0.004 | 0.164 | 0.001 | 0.099 | 0.079 | 0.007 | 0.038 | 0.036 | 0.017 | 0.0021 | 457 | 1215 | 513 | 639 |
| 0.079 | 0.24 | 1.16 | 0.010 | 0.001 | 0.161 | 0.002 | 0.058 | 0.064 | 0.002 | 0.025 | 0.036 | 0.016 | 0.0027 | 452 | 1238 | 514 | 626 |
| 0.079 | 0.24 | 1.44 | 0.009 | 0.004 | 0.161 | 0.002 | 0.074 | 0.040 | 0.006 | 0.035 | 0.036 | 0.016 | 0.0007 | 414 | 1212 | 505 | 635 |
| 0.079 | 0.22 | 1.40 | 0.010 | 0.003 | 0.173 | 0.001 | 0.041 | 0.037 | 0.005 | 0.019 | 0.040 | 0.016 | 0.0020 | 415 | 1238 | 488 | 622 |
| 0.079 | 0.22 | 1.44 | 0.010 | 0.004 | 0.162 | 0.001 | 0.073 | 0.085 | 0.005 | 0.036 | 0.042 | 0.016 | 0.0021 | 457 | 1212 | 517 | 642 |
| 0.079 | 0.21 | 1.16 | 0.013 | 0.003 | 0.066 | 0.001 | 0.018 | 0.062 | 0.005 | 0.049 | 0.036 | 0.016 | 0.0002 | 455 | 1241 | 534 | 625 |
| 0.079 | 0.30 | 1.46 | 0.012 | 0.004 | 0.173 | 0.002 | 0.066 | 0.081 | 0.003 | 0.041 | 0.036 | 0.016 | 0.0016 | 427 | 1196 | 509 | 624 |
| 0.079 | 0.22 | 1.16 | 0.010 | 0.002 | 0.162 | 0.001 | 0.074 | 0.053 | 0.008 | 0.047 | 0.037 | 0.016 | 0.0003 | 453 | 1245 | 528 | 620 |
| 0.079 | 0.24 | 1.52 | 0.010 | 0.004 | 0.097 | 0.001 | 0.054 | 0.044 | 0.005 | 0.037 | 0.036 | 0.011 | 0.0028 | 457 | 1213 | 524 | 618 |
|  |  |  |  |  |  |  |  |  |  |  |  |  |  |  |  |  |  |
| **XG Boost** | | | | | | | | | | | | | | | | | |
| C | Si | Mn | P | S | Cu | Sn | Ni | Cr | Mo | V | Nb | Ti | Ca | Time | Temp | YS | UTS |
| (wt%) | (wt%) | (wt%) | (wt%) | (wt%) | (wt%) | (wt%) | (wt%) | (wt%) | (wt%) | (wt%) | (wt%) | (wt%) | (wt%) | (s) | (℃) | (Mpa) | (Mpa) |
| 0.082 | 0.28 | 1.35 | 0.009 | 0.002 | 0.145 | 0.002 | 0.012 | 0.056 | 0.005 | 0.049 | 0.036 | 0.013 | 0.0012 | 412 | 1082 | 474 | 598 |
| 0.074 | 0.29 | 1.39 | 0.007 | 0.003 | 0.163 | 0.002 | 0.041 | 0.103 | 0.005 | 0.037 | 0.040 | 0.012 | 0.0006 | 416 | 1187 | 477 | 568 |
| 0.081 | 0.24 | 1.16 | 0.007 | 0.002 | 0.148 | 0.001 | 0.013 | 0.065 | 0.005 | 0.026 | 0.036 | 0.016 | 0.0012 | 417 | 1211 | 493 | 587 |
| 0.075 | 0.30 | 1.44 | 0.012 | 0.004 | 0.022 | 0.001 | 0.138 | 0.109 | 0.003 | 0.049 | 0.037 | 0.013 | 0.0011 | 345 | 1185 | 487 | 616 |
| 0.083 | 0.27 | 1.31 | 0.009 | 0.002 | 0.146 | 0.001 | 0.008 | 0.092 | 0.005 | 0.048 | 0.043 | 0.011 | 0.0023 | 416 | 1083 | 470 | 598 |
| 0.078 | 0.25 | 1.49 | 0.013 | 0.003 | 0.136 | 0.002 | 0.010 | 0.077 | 0.006 | 0.036 | 0.039 | 0.013 | 0.0003 | 450 | 1016 | 465 | 577 |
| 0.082 | 0.24 | 1.39 | 0.009 | 0.002 | 0.169 | 0.001 | 0.061 | 0.051 | 0.005 | 0.047 | 0.042 | 0.013 | 0.0014 | 454 | 1185 | 486 | 601 |
| 0.082 | 0.30 | 1.44 | 0.009 | 0.003 | 0.080 | 0.002 | 0.124 | 0.036 | 0.004 | 0.048 | 0.042 | 0.013 | 0.0027 | 389 | 1166 | 474 | 596 |
| 0.081 | 0.24 | 1.39 | 0.009 | 0.003 | 0.011 | 0.001 | 0.061 | 0.111 | 0.002 | 0.048 | 0.039 | 0.012 | 0.0005 | 424 | 1183 | 481 | 600 |
| 0.072 | 0.22 | 1.47 | 0.009 | 0.002 | 0.144 | 0.001 | 0.092 | 0.075 | 0.004 | 0.044 | 0.038 | 0.016 | 0.0018 | 421 | 1192 | 498 | 580 |
| 0.075 | 0.30 | 1.17 | 0.006 | 0.001 | 0.143 | 0.002 | 0.088 | 0.029 | 0.005 | 0.036 | 0.040 | 0.016 | 0.0027 | 417 | 1197 | 493 | 581 |
| 0.074 | 0.29 | 1.37 | 0.008 | 0.004 | 0.168 | 0.002 | 0.031 | 0.058 | 0.003 | 0.049 | 0.036 | 0.012 | 0.0005 | 422 | 1186 | 507 | 598 |
| 0.082 | 0.28 | 1.32 | 0.009 | 0.002 | 0.016 | 0.002 | 0.109 | 0.026 | 0.003 | 0.048 | 0.041 | 0.014 | 0.0003 | 417 | 1082 | 476 | 599 |
| 0.081 | 0.27 | 1.37 | 0.009 | 0.002 | 0.147 | 0.001 | 0.081 | 0.059 | 0.002 | 0.048 | 0.042 | 0.012 | 0.0003 | 439 | 1097 | 474 | 597 |
| 0.081 | 0.29 | 1.41 | 0.010 | 0.003 | 0.149 | 0.002 | 0.082 | 0.105 | 0.003 | 0.048 | 0.040 | 0.012 | 0.0004 | 395 | 1186 | 489 | 598 |
| 0.074 | 0.27 | 1.46 | 0.008 | 0.002 | 0.166 | 0.003 | 0.045 | 0.092 | 0.004 | 0.048 | 0.043 | 0.012 | 0.0004 | 419 | 1186 | 491 | 602 |
| 0.082 | 0.27 | 1.35 | 0.010 | 0.002 | 0.126 | 0.001 | 0.108 | 0.041 | 0.004 | 0.048 | 0.037 | 0.014 | 0.0003 | 410 | 1036 | 470 | 599 |
| 0.081 | 0.25 | 1.39 | 0.009 | 0.003 | 0.108 | 0.001 | 0.057 | 0.075 | 0.006 | 0.048 | 0.040 | 0.011 | 0.0025 | 420 | 1189 | 480 | 596 |
| 0.082 | 0.27 | 1.35 | 0.009 | 0.002 | 0.145 | 0.002 | 0.014 | 0.085 | 0.001 | 0.048 | 0.040 | 0.012 | 0.0003 | 413 | 1082 | 478 | 602 |
| 0.081 | 0.28 | 1.44 | 0.012 | 0.001 | 0.018 | 0.001 | 0.122 | 0.059 | 0.006 | 0.047 | 0.039 | 0.012 | 0.0011 | 399 | 1076 | 465 | 613 |
| 0.083 | 0.28 | 1.35 | 0.010 | 0.001 | 0.047 | 0.002 | 0.131 | 0.068 | 0.004 | 0.050 | 0.042 | 0.013 | 0.0003 | 422 | 1096 | 476 | 598 |
| 0.082 | 0.26 | 1.35 | 0.015 | 0.001 | 0.017 | 0.002 | 0.110 | 0.079 | 0.005 | 0.048 | 0.040 | 0.012 | 0.0013 | 399 | 1082 | 454 | 609 |
| 0.075 | 0.27 | 1.46 | 0.009 | 0.002 | 0.167 | 0.001 | 0.083 | 0.072 | 0.005 | 0.048 | 0.038 | 0.012 | 0.0004 | 415 | 1185 | 492 | 600 |
| 0.074 | 0.27 | 1.36 | 0.009 | 0.003 | 0.167 | 0.002 | 0.048 | 0.100 | 0.003 | 0.048 | 0.038 | 0.012 | 0.0006 | 437 | 1195 | 482 | 593 |
| 0.081 | 0.31 | 1.43 | 0.012 | 0.004 | 0.022 | 0.001 | 0.112 | 0.053 | 0.006 | 0.049 | 0.037 | 0.013 | 0.0004 | 339 | 1185 | 493 | 613 |
| 0.082 | 0.27 | 1.35 | 0.009 | 0.002 | 0.012 | 0.001 | 0.107 | 0.068 | 0.005 | 0.047 | 0.042 | 0.014 | 0.0002 | 444 | 1036 | 471 | 599 |
| 0.082 | 0.28 | 1.35 | 0.008 | 0.001 | 0.146 | 0.001 | 0.061 | 0.055 | 0.005 | 0.047 | 0.036 | 0.014 | 0.0013 | 417 | 1076 | 473 | 598 |
| 0.082 | 0.28 | 1.40 | 0.009 | 0.002 | 0.160 | 0.002 | 0.084 | 0.060 | 0.006 | 0.048 | 0.037 | 0.011 | 0.0003 | 423 | 1080 | 476 | 600 |
| 0.079 | 0.27 | 1.40 | 0.013 | 0.002 | 0.162 | 0.003 | 0.049 | 0.088 | 0.005 | 0.049 | 0.036 | 0.012 | 0.0011 | 450 | 1202 | 486 | 589 |
| 0.081 | 0.30 | 1.48 | 0.012 | 0.003 | 0.097 | 0.001 | 0.107 | 0.073 | 0.005 | 0.049 | 0.041 | 0.015 | 0.0005 | 343 | 1154 | 475 | 608 |
| 0.074 | 0.29 | 1.31 | 0.009 | 0.003 | 0.168 | 0.001 | 0.106 | 0.086 | 0.005 | 0.048 | 0.036 | 0.013 | 0.0004 | 411 | 1192 | 484 | 592 |
| 0.074 | 0.28 | 1.30 | 0.009 | 0.004 | 0.081 | 0.001 | 0.012 | 0.100 | 0.003 | 0.048 | 0.038 | 0.014 | 0.0010 | 437 | 1195 | 480 | 594 |
| 0.075 | 0.22 | 1.36 | 0.008 | 0.002 | 0.147 | 0.001 | 0.029 | 0.069 | 0.008 | 0.035 | 0.037 | 0.016 | 0.0010 | 424 | 1186 | 492 | 583 |
| 0.075 | 0.28 | 1.47 | 0.008 | 0.004 | 0.058 | 0.002 | 0.080 | 0.087 | 0.004 | 0.048 | 0.039 | 0.013 | 0.0007 | 349 | 1185 | 478 | 602 |
| 0.074 | 0.22 | 1.37 | 0.009 | 0.002 | 0.145 | 0.001 | 0.096 | 0.078 | 0.005 | 0.049 | 0.037 | 0.016 | 0.0019 | 426 | 1191 | 498 | 591 |
| 0.082 | 0.29 | 1.18 | 0.009 | 0.002 | 0.018 | 0.001 | 0.115 | 0.089 | 0.005 | 0.048 | 0.035 | 0.011 | 0.0004 | 451 | 1085 | 478 | 594 |
| 0.082 | 0.27 | 1.31 | 0.013 | 0.003 | 0.109 | 0.002 | 0.083 | 0.040 | 0.005 | 0.048 | 0.034 | 0.014 | 0.0003 | 414 | 1079 | 470 | 594 |
| 0.075 | 0.26 | 1.45 | 0.009 | 0.004 | 0.073 | 0.001 | 0.140 | 0.138 | 0.004 | 0.049 | 0.043 | 0.013 | 0.0011 | 399 | 1185 | 503 | 607 |
| 0.081 | 0.25 | 1.35 | 0.012 | 0.001 | 0.016 | 0.001 | 0.093 | 0.073 | 0.005 | 0.049 | 0.038 | 0.013 | 0.0003 | 452 | 1030 | 471 | 613 |
| 0.082 | 0.28 | 1.40 | 0.009 | 0.002 | 0.145 | 0.002 | 0.084 | 0.060 | 0.006 | 0.048 | 0.037 | 0.011 | 0.0003 | 423 | 1080 | 474 | 600 |
| 0.080 | 0.27 | 1.43 | 0.014 | 0.001 | 0.017 | 0.001 | 0.014 | 0.102 | 0.004 | 0.050 | 0.040 | 0.012 | 0.0005 | 322 | 1181 | 489 | 604 |
| 0.081 | 0.27 | 1.30 | 0.009 | 0.001 | 0.040 | 0.002 | 0.012 | 0.093 | 0.002 | 0.048 | 0.036 | 0.012 | 0.0003 | 398 | 1040 | 487 | 590 |
| 0.081 | 0.27 | 1.32 | 0.009 | 0.001 | 0.135 | 0.001 | 0.097 | 0.085 | 0.006 | 0.043 | 0.042 | 0.013 | 0.0026 | 415 | 1097 | 478 | 564 |
| 0.072 | 0.23 | 1.18 | 0.007 | 0.002 | 0.088 | 0.001 | 0.075 | 0.053 | 0.005 | 0.038 | 0.039 | 0.016 | 0.0027 | 435 | 1191 | 496 | 547 |
| 0.082 | 0.23 | 1.16 | 0.008 | 0.002 | 0.020 | 0.002 | 0.017 | 0.144 | 0.003 | 0.048 | 0.038 | 0.016 | 0.0013 | 306 | 1165 | 500 | 590 |
| 0.082 | 0.27 | 1.47 | 0.015 | 0.001 | 0.058 | 0.001 | 0.112 | 0.088 | 0.006 | 0.048 | 0.040 | 0.016 | 0.0010 | 338 | 1160 | 472 | 612 |
| 0.081 | 0.28 | 1.39 | 0.012 | 0.003 | 0.017 | 0.002 | 0.047 | 0.038 | 0.004 | 0.048 | 0.039 | 0.013 | 0.0004 | 438 | 1087 | 462 | 609 |
| 0.081 | 0.29 | 1.51 | 0.009 | 0.004 | 0.059 | 0.001 | 0.097 | 0.084 | 0.005 | 0.048 | 0.039 | 0.015 | 0.0003 | 405 | 1159 | 485 | 606 |
| 0.074 | 0.22 | 1.39 | 0.009 | 0.002 | 0.144 | 0.001 | 0.038 | 0.093 | 0.002 | 0.021 | 0.036 | 0.016 | 0.0021 | 426 | 1196 | 490 | 581 |
| 0.082 | 0.27 | 1.19 | 0.009 | 0.001 | 0.029 | 0.002 | 0.094 | 0.071 | 0.003 | 0.050 | 0.036 | 0.014 | 0.0009 | 446 | 1084 | 477 | 592 |
| 0.083 | 0.28 | 1.45 | 0.012 | 0.001 | 0.017 | 0.001 | 0.109 | 0.041 | 0.005 | 0.048 | 0.036 | 0.013 | 0.0009 | 419 | 1108 | 461 | 611 |
| 0.081 | 0.27 | 1.42 | 0.013 | 0.002 | 0.075 | 0.002 | 0.143 | 0.101 | 0.006 | 0.048 | 0.039 | 0.011 | 0.0003 | 413 | 1082 | 470 | 605 |
| 0.080 | 0.22 | 1.37 | 0.009 | 0.002 | 0.041 | 0.001 | 0.068 | 0.066 | 0.005 | 0.048 | 0.042 | 0.013 | 0.0010 | 437 | 1191 | 487 | 597 |
| 0.074 | 0.22 | 1.39 | 0.009 | 0.003 | 0.085 | 0.001 | 0.063 | 0.054 | 0.005 | 0.047 | 0.041 | 0.015 | 0.0007 | 438 | 1192 | 487 | 594 |
| 0.070 | 0.22 | 1.36 | 0.009 | 0.002 | 0.156 | 0.001 | 0.091 | 0.087 | 0.006 | 0.035 | 0.033 | 0.016 | 0.0018 | 431 | 1223 | 491 | 573 |
| 0.082 | 0.28 | 1.35 | 0.010 | 0.002 | 0.143 | 0.002 | 0.017 | 0.092 | 0.004 | 0.048 | 0.036 | 0.013 | 0.0003 | 417 | 1084 | 479 | 604 |
| 0.082 | 0.30 | 1.46 | 0.012 | 0.001 | 0.017 | 0.001 | 0.084 | 0.049 | 0.006 | 0.049 | 0.036 | 0.016 | 0.0008 | 339 | 1160 | 489 | 616 |
| 0.075 | 0.24 | 1.45 | 0.009 | 0.003 | 0.024 | 0.002 | 0.033 | 0.102 | 0.004 | 0.049 | 0.038 | 0.012 | 0.0028 | 453 | 1186 | 491 | 596 |
| 0.075 | 0.24 | 1.45 | 0.009 | 0.003 | 0.046 | 0.002 | 0.033 | 0.102 | 0.004 | 0.049 | 0.038 | 0.012 | 0.0028 | 453 | 1186 | 491 | 596 |
| 0.074 | 0.27 | 1.51 | 0.009 | 0.002 | 0.031 | 0.001 | 0.097 | 0.115 | 0.004 | 0.048 | 0.039 | 0.010 | 0.0012 | 453 | 1185 | 491 | 594 |
| 0.084 | 0.28 | 1.48 | 0.009 | 0.003 | 0.139 | 0.001 | 0.085 | 0.029 | 0.001 | 0.048 | 0.038 | 0.014 | 0.0003 | 390 | 1153 | 480 | 605 |
| 0.082 | 0.27 | 1.44 | 0.012 | 0.001 | 0.072 | 0.002 | 0.142 | 0.070 | 0.003 | 0.049 | 0.037 | 0.013 | 0.0013 | 453 | 1080 | 463 | 609 |
| 0.083 | 0.27 | 1.38 | 0.009 | 0.002 | 0.144 | 0.001 | 0.017 | 0.090 | 0.005 | 0.048 | 0.039 | 0.013 | 0.0003 | 411 | 1035 | 472 | 600 |
| 0.081 | 0.24 | 1.35 | 0.012 | 0.001 | 0.019 | 0.001 | 0.080 | 0.126 | 0.004 | 0.048 | 0.038 | 0.013 | 0.0003 | 396 | 956 | 477 | 599 |
| 0.079 | 0.27 | 1.50 | 0.012 | 0.004 | 0.154 | 0.002 | 0.084 | 0.053 | 0.003 | 0.049 | 0.039 | 0.014 | 0.0013 | 326 | 1180 | 493 | 613 |
| 0.082 | 0.27 | 1.49 | 0.012 | 0.001 | 0.016 | 0.001 | 0.111 | 0.100 | 0.006 | 0.048 | 0.040 | 0.013 | 0.0005 | 396 | 1082 | 472 | 612 |
| 0.074 | 0.27 | 1.47 | 0.009 | 0.002 | 0.149 | 0.003 | 0.086 | 0.028 | 0.003 | 0.048 | 0.039 | 0.012 | 0.0004 | 399 | 1186 | 493 | 597 |
| 0.082 | 0.30 | 1.40 | 0.008 | 0.003 | 0.145 | 0.002 | 0.161 | 0.068 | 0.003 | 0.049 | 0.038 | 0.012 | 0.0003 | 420 | 1086 | 464 | 602 |
| 0.077 | 0.27 | 1.45 | 0.014 | 0.004 | 0.161 | 0.002 | 0.082 | 0.076 | 0.006 | 0.048 | 0.037 | 0.012 | 0.0010 | 324 | 1184 | 495 | 615 |
| 0.081 | 0.22 | 1.38 | 0.010 | 0.003 | 0.046 | 0.001 | 0.049 | 0.092 | 0.007 | 0.049 | 0.037 | 0.010 | 0.0005 | 422 | 1186 | 487 | 605 |
| 0.083 | 0.27 | 1.45 | 0.009 | 0.002 | 0.017 | 0.001 | 0.138 | 0.060 | 0.005 | 0.047 | 0.040 | 0.016 | 0.0023 | 410 | 1153 | 471 | 600 |
| 0.081 | 0.27 | 1.51 | 0.013 | 0.001 | 0.090 | 0.001 | 0.092 | 0.110 | 0.002 | 0.048 | 0.042 | 0.013 | 0.0015 | 397 | 1071 | 465 | 604 |
| 0.081 | 0.28 | 1.39 | 0.012 | 0.001 | 0.016 | 0.002 | 0.082 | 0.101 | 0.004 | 0.049 | 0.037 | 0.013 | 0.0005 | 454 | 1083 | 467 | 615 |
| 0.081 | 0.24 | 1.46 | 0.013 | 0.001 | 0.014 | 0.001 | 0.062 | 0.111 | 0.006 | 0.048 | 0.042 | 0.010 | 0.0023 | 424 | 1185 | 484 | 612 |
| 0.081 | 0.27 | 1.35 | 0.012 | 0.001 | 0.143 | 0.002 | 0.019 | 0.092 | 0.003 | 0.049 | 0.037 | 0.013 | 0.0008 | 458 | 1082 | 464 | 607 |
| 0.081 | 0.26 | 1.46 | 0.012 | 0.002 | 0.136 | 0.001 | 0.012 | 0.071 | 0.005 | 0.048 | 0.040 | 0.013 | 0.0005 | 395 | 1080 | 472 | 607 |
| 0.062 | 0.23 | 1.17 | 0.005 | 0.002 | 0.144 | 0.001 | 0.079 | 0.085 | 0.003 | 0.035 | 0.034 | 0.016 | 0.0016 | 293 | 1196 | 495 | 579 |
| 0.074 | 0.24 | 1.45 | 0.009 | 0.004 | 0.099 | 0.002 | 0.032 | 0.053 | 0.004 | 0.049 | 0.038 | 0.010 | 0.0004 | 453 | 1186 | 499 | 598 |
| 0.076 | 0.31 | 1.45 | 0.008 | 0.004 | 0.018 | 0.001 | 0.089 | 0.141 | 0.003 | 0.047 | 0.036 | 0.013 | 0.0009 | 342 | 1161 | 497 | 602 |
| 0.082 | 0.27 | 1.32 | 0.009 | 0.002 | 0.148 | 0.002 | 0.010 | 0.072 | 0.003 | 0.048 | 0.036 | 0.012 | 0.0003 | 422 | 1082 | 482 | 599 |
| 0.081 | 0.28 | 1.42 | 0.012 | 0.001 | 0.088 | 0.002 | 0.086 | 0.075 | 0.002 | 0.048 | 0.039 | 0.013 | 0.0003 | 415 | 1082 | 477 | 602 |
| 0.081 | 0.27 | 1.35 | 0.010 | 0.002 | 0.089 | 0.001 | 0.021 | 0.055 | 0.004 | 0.048 | 0.040 | 0.013 | 0.0003 | 418 | 1096 | 477 | 601 |
| 0.082 | 0.26 | 1.29 | 0.009 | 0.001 | 0.092 | 0.002 | 0.082 | 0.073 | 0.005 | 0.048 | 0.038 | 0.014 | 0.0012 | 418 | 1094 | 473 | 594 |
| 0.081 | 0.28 | 1.32 | 0.009 | 0.002 | 0.144 | 0.001 | 0.019 | 0.080 | 0.002 | 0.048 | 0.041 | 0.013 | 0.0003 | 417 | 1034 | 482 | 596 |
| 0.082 | 0.28 | 1.33 | 0.013 | 0.001 | 0.016 | 0.002 | 0.117 | 0.072 | 0.004 | 0.048 | 0.035 | 0.013 | 0.0003 | 399 | 1084 | 474 | 605 |
| 0.075 | 0.30 | 1.46 | 0.013 | 0.004 | 0.081 | 0.002 | 0.082 | 0.094 | 0.004 | 0.049 | 0.038 | 0.013 | 0.0012 | 342 | 1185 | 487 | 614 |
| 0.083 | 0.26 | 1.32 | 0.009 | 0.002 | 0.146 | 0.002 | 0.014 | 0.078 | 0.003 | 0.048 | 0.038 | 0.013 | 0.0003 | 421 | 1087 | 481 | 599 |
| 0.078 | 0.21 | 1.37 | 0.009 | 0.002 | 0.088 | 0.003 | 0.044 | 0.067 | 0.006 | 0.032 | 0.041 | 0.016 | 0.0021 | 424 | 1185 | 501 | 550 |
| 0.080 | 0.29 | 1.46 | 0.013 | 0.001 | 0.168 | 0.002 | 0.042 | 0.055 | 0.004 | 0.048 | 0.037 | 0.011 | 0.0013 | 411 | 1190 | 482 | 600 |
| 0.076 | 0.25 | 1.46 | 0.012 | 0.003 | 0.077 | 0.002 | 0.100 | 0.099 | 0.001 | 0.048 | 0.042 | 0.014 | 0.0010 | 341 | 1182 | 488 | 601 |
| 0.082 | 0.28 | 1.35 | 0.009 | 0.002 | 0.045 | 0.002 | 0.128 | 0.074 | 0.004 | 0.048 | 0.036 | 0.013 | 0.0003 | 460 | 1041 | 474 | 601 |
| 0.074 | 0.31 | 1.54 | 0.013 | 0.004 | 0.026 | 0.002 | 0.141 | 0.025 | 0.002 | 0.049 | 0.036 | 0.011 | 0.0026 | 462 | 1186 | 489 | 611 |
| 0.082 | 0.25 | 1.45 | 0.009 | 0.002 | 0.095 | 0.002 | 0.111 | 0.065 | 0.001 | 0.048 | 0.036 | 0.012 | 0.0013 | 461 | 1186 | 490 | 599 |
| 0.081 | 0.27 | 1.47 | 0.009 | 0.003 | 0.148 | 0.001 | 0.093 | 0.138 | 0.006 | 0.050 | 0.041 | 0.011 | 0.0004 | 424 | 1197 | 486 | 602 |
| 0.082 | 0.28 | 1.39 | 0.010 | 0.001 | 0.020 | 0.001 | 0.109 | 0.091 | 0.007 | 0.048 | 0.037 | 0.011 | 0.0013 | 417 | 1083 | 471 | 600 |
| 0.076 | 0.30 | 1.46 | 0.012 | 0.004 | 0.024 | 0.001 | 0.102 | 0.058 | 0.004 | 0.049 | 0.037 | 0.012 | 0.0009 | 328 | 1185 | 505 | 615 |
| 0.082 | 0.25 | 1.35 | 0.012 | 0.001 | 0.020 | 0.002 | 0.129 | 0.077 | 0.005 | 0.049 | 0.037 | 0.012 | 0.0005 | 453 | 1086 | 470 | 609 |
| 0.082 | 0.27 | 1.46 | 0.012 | 0.001 | 0.017 | 0.001 | 0.023 | 0.066 | 0.006 | 0.048 | 0.036 | 0.013 | 0.0003 | 399 | 1082 | 471 | 611 |
| 0.081 | 0.27 | 1.40 | 0.012 | 0.002 | 0.016 | 0.002 | 0.097 | 0.067 | 0.002 | 0.048 | 0.037 | 0.014 | 0.0003 | 437 | 1082 | 465 | 608 |
| 0.082 | 0.28 | 1.35 | 0.006 | 0.001 | 0.077 | 0.002 | 0.087 | 0.064 | 0.003 | 0.047 | 0.035 | 0.013 | 0.0002 | 419 | 960 | 470 | 593 |
| 0.084 | 0.28 | 1.40 | 0.010 | 0.001 | 0.077 | 0.001 | 0.046 | 0.066 | 0.004 | 0.048 | 0.036 | 0.014 | 0.0007 | 437 | 1095 | 475 | 592 |
| 0.081 | 0.25 | 1.48 | 0.012 | 0.003 | 0.059 | 0.002 | 0.037 | 0.064 | 0.005 | 0.048 | 0.036 | 0.012 | 0.0004 | 461 | 1184 | 480 | 611 |
| 0.075 | 0.27 | 1.45 | 0.009 | 0.004 | 0.038 | 0.001 | 0.050 | 0.051 | 0.003 | 0.050 | 0.038 | 0.011 | 0.0010 | 453 | 1185 | 500 | 605 |
| 0.081 | 0.29 | 1.35 | 0.012 | 0.002 | 0.017 | 0.001 | 0.047 | 0.049 | 0.003 | 0.049 | 0.037 | 0.012 | 0.0003 | 446 | 1080 | 466 | 614 |
| 0.082 | 0.26 | 1.45 | 0.008 | 0.003 | 0.168 | 0.001 | 0.038 | 0.056 | 0.005 | 0.049 | 0.038 | 0.012 | 0.0004 | 424 | 1186 | 483 | 606 |
| 0.071 | 0.24 | 1.32 | 0.009 | 0.002 | 0.145 | 0.003 | 0.071 | 0.099 | 0.006 | 0.024 | 0.036 | 0.016 | 0.0027 | 455 | 1226 | 492 | 575 |
| 0.081 | 0.28 | 1.16 | 0.009 | 0.002 | 0.144 | 0.001 | 0.021 | 0.100 | 0.006 | 0.033 | 0.037 | 0.012 | 0.0005 | 421 | 1082 | 478 | 564 |
| 0.081 | 0.25 | 1.45 | 0.013 | 0.001 | 0.020 | 0.002 | 0.143 | 0.060 | 0.007 | 0.048 | 0.036 | 0.013 | 0.0003 | 394 | 1083 | 473 | 609 |
| 0.082 | 0.27 | 1.35 | 0.009 | 0.002 | 0.164 | 0.002 | 0.094 | 0.084 | 0.003 | 0.048 | 0.037 | 0.013 | 0.0003 | 418 | 1081 | 479 | 603 |
| 0.074 | 0.27 | 1.47 | 0.009 | 0.002 | 0.168 | 0.001 | 0.064 | 0.066 | 0.003 | 0.048 | 0.043 | 0.010 | 0.0004 | 403 | 1186 | 488 | 601 |
| 0.080 | 0.27 | 1.44 | 0.012 | 0.003 | 0.161 | 0.001 | 0.080 | 0.067 | 0.008 | 0.047 | 0.037 | 0.014 | 0.0005 | 389 | 1195 | 480 | 597 |
| 0.078 | 0.30 | 1.48 | 0.013 | 0.003 | 0.023 | 0.001 | 0.104 | 0.063 | 0.003 | 0.049 | 0.041 | 0.013 | 0.0007 | 327 | 1178 | 502 | 604 |
| 0.082 | 0.30 | 1.17 | 0.009 | 0.002 | 0.150 | 0.001 | 0.026 | 0.050 | 0.002 | 0.048 | 0.037 | 0.012 | 0.0004 | 416 | 1195 | 496 | 599 |
| 0.082 | 0.29 | 1.35 | 0.009 | 0.002 | 0.038 | 0.002 | 0.013 | 0.081 | 0.005 | 0.048 | 0.035 | 0.013 | 0.0004 | 399 | 1072 | 477 | 593 |
| 0.075 | 0.22 | 1.32 | 0.009 | 0.002 | 0.144 | 0.003 | 0.081 | 0.065 | 0.006 | 0.028 | 0.039 | 0.016 | 0.0016 | 400 | 1185 | 500 | 576 |
| 0.082 | 0.22 | 1.36 | 0.009 | 0.002 | 0.152 | 0.001 | 0.147 | 0.066 | 0.006 | 0.023 | 0.041 | 0.016 | 0.0019 | 450 | 1232 | 477 | 594 |
| 0.075 | 0.30 | 1.46 | 0.012 | 0.004 | 0.171 | 0.001 | 0.141 | 0.068 | 0.002 | 0.049 | 0.039 | 0.014 | 0.0011 | 336 | 1182 | 498 | 614 |
| 0.081 | 0.25 | 1.45 | 0.012 | 0.003 | 0.060 | 0.001 | 0.084 | 0.065 | 0.007 | 0.048 | 0.039 | 0.014 | 0.0022 | 397 | 1183 | 481 | 607 |
| 0.081 | 0.28 | 1.45 | 0.010 | 0.003 | 0.018 | 0.001 | 0.081 | 0.038 | 0.005 | 0.047 | 0.038 | 0.015 | 0.0003 | 350 | 1146 | 466 | 606 |
| 0.071 | 0.22 | 1.51 | 0.010 | 0.002 | 0.152 | 0.001 | 0.143 | 0.050 | 0.003 | 0.034 | 0.033 | 0.017 | 0.0004 | 339 | 1192 | 470 | 581 |
| 0.081 | 0.30 | 1.47 | 0.013 | 0.003 | 0.023 | 0.002 | 0.119 | 0.084 | 0.008 | 0.048 | 0.039 | 0.014 | 0.0026 | 395 | 1153 | 470 | 609 |
| 0.075 | 0.29 | 1.45 | 0.009 | 0.004 | 0.149 | 0.001 | 0.069 | 0.041 | 0.005 | 0.048 | 0.039 | 0.012 | 0.0011 | 399 | 1185 | 496 | 599 |
| 0.081 | 0.30 | 1.40 | 0.009 | 0.004 | 0.020 | 0.001 | 0.124 | 0.088 | 0.005 | 0.049 | 0.040 | 0.012 | 0.0004 | 446 | 1084 | 475 | 608 |
| 0.082 | 0.27 | 1.45 | 0.009 | 0.002 | 0.061 | 0.001 | 0.029 | 0.069 | 0.006 | 0.048 | 0.041 | 0.012 | 0.0004 | 454 | 1185 | 487 | 600 |
| 0.081 | 0.28 | 1.40 | 0.010 | 0.002 | 0.083 | 0.001 | 0.100 | 0.074 | 0.004 | 0.048 | 0.040 | 0.013 | 0.0003 | 420 | 1099 | 471 | 602 |
| 0.081 | 0.28 | 1.47 | 0.009 | 0.003 | 0.042 | 0.001 | 0.041 | 0.098 | 0.008 | 0.050 | 0.043 | 0.012 | 0.0004 | 457 | 1182 | 480 | 607 |
| 0.081 | 0.25 | 1.35 | 0.012 | 0.002 | 0.035 | 0.001 | 0.090 | 0.100 | 0.006 | 0.050 | 0.037 | 0.014 | 0.0003 | 449 | 956 | 467 | 603 |
| 0.081 | 0.22 | 1.33 | 0.009 | 0.002 | 0.147 | 0.001 | 0.016 | 0.096 | 0.006 | 0.028 | 0.036 | 0.016 | 0.0018 | 417 | 1225 | 491 | 586 |
| 0.074 | 0.22 | 1.39 | 0.009 | 0.002 | 0.146 | 0.001 | 0.016 | 0.100 | 0.005 | 0.038 | 0.037 | 0.016 | 0.0018 | 454 | 1225 | 491 | 582 |
| 0.082 | 0.26 | 1.48 | 0.009 | 0.002 | 0.163 | 0.003 | 0.068 | 0.116 | 0.005 | 0.048 | 0.039 | 0.010 | 0.0004 | 436 | 1186 | 489 | 606 |
| 0.081 | 0.29 | 1.46 | 0.012 | 0.003 | 0.062 | 0.001 | 0.091 | 0.077 | 0.006 | 0.048 | 0.041 | 0.012 | 0.0013 | 392 | 1183 | 477 | 610 |
| 0.082 | 0.27 | 1.45 | 0.012 | 0.003 | 0.154 | 0.003 | 0.084 | 0.114 | 0.003 | 0.049 | 0.038 | 0.012 | 0.0004 | 453 | 1182 | 478 | 617 |
| 0.070 | 0.30 | 1.44 | 0.012 | 0.004 | 0.016 | 0.001 | 0.009 | 0.105 | 0.007 | 0.048 | 0.033 | 0.012 | 0.0021 | 427 | 1123 | 471 | 600 |
| 0.074 | 0.25 | 1.31 | 0.009 | 0.002 | 0.173 | 0.001 | 0.059 | 0.098 | 0.005 | 0.048 | 0.038 | 0.011 | 0.0024 | 419 | 1188 | 490 | 585 |
| 0.081 | 0.27 | 1.45 | 0.012 | 0.001 | 0.084 | 0.001 | 0.102 | 0.064 | 0.006 | 0.048 | 0.043 | 0.013 | 0.0013 | 412 | 1080 | 463 | 608 |
| 0.081 | 0.28 | 1.35 | 0.010 | 0.002 | 0.028 | 0.002 | 0.117 | 0.058 | 0.003 | 0.048 | 0.037 | 0.013 | 0.0005 | 451 | 1085 | 476 | 601 |
| 0.082 | 0.24 | 1.41 | 0.009 | 0.003 | 0.171 | 0.001 | 0.096 | 0.086 | 0.005 | 0.049 | 0.037 | 0.013 | 0.0011 | 451 | 1186 | 483 | 604 |
| 0.081 | 0.27 | 1.39 | 0.009 | 0.004 | 0.043 | 0.001 | 0.128 | 0.053 | 0.005 | 0.048 | 0.036 | 0.012 | 0.0015 | 411 | 1186 | 493 | 601 |
| 0.081 | 0.21 | 1.17 | 0.008 | 0.002 | 0.054 | 0.001 | 0.058 | 0.047 | 0.002 | 0.048 | 0.041 | 0.016 | 0.0012 | 396 | 1195 | 506 | 594 |
| 0.081 | 0.25 | 1.45 | 0.012 | 0.001 | 0.076 | 0.001 | 0.090 | 0.141 | 0.005 | 0.049 | 0.040 | 0.010 | 0.0016 | 402 | 1186 | 483 | 606 |
| 0.075 | 0.27 | 1.47 | 0.009 | 0.001 | 0.168 | 0.003 | 0.086 | 0.073 | 0.003 | 0.049 | 0.038 | 0.012 | 0.0015 | 399 | 1185 | 489 | 598 |
| 0.081 | 0.27 | 1.50 | 0.010 | 0.001 | 0.069 | 0.001 | 0.081 | 0.062 | 0.006 | 0.048 | 0.039 | 0.014 | 0.0003 | 395 | 1154 | 477 | 605 |
| 0.081 | 0.29 | 1.35 | 0.012 | 0.001 | 0.016 | 0.002 | 0.093 | 0.076 | 0.002 | 0.048 | 0.036 | 0.013 | 0.0005 | 444 | 1076 | 463 | 609 |
| 0.078 | 0.26 | 1.49 | 0.012 | 0.003 | 0.086 | 0.002 | 0.124 | 0.068 | 0.005 | 0.048 | 0.036 | 0.014 | 0.0005 | 401 | 1080 | 463 | 591 |
| 0.082 | 0.23 | 1.17 | 0.006 | 0.002 | 0.146 | 0.003 | 0.101 | 0.114 | 0.005 | 0.022 | 0.039 | 0.017 | 0.0016 | 398 | 1224 | 490 | 590 |
| 0.082 | 0.27 | 1.35 | 0.009 | 0.001 | 0.069 | 0.001 | 0.103 | 0.059 | 0.001 | 0.049 | 0.037 | 0.012 | 0.0003 | 453 | 980 | 474 | 598 |
| 0.084 | 0.25 | 1.36 | 0.014 | 0.001 | 0.097 | 0.001 | 0.121 | 0.069 | 0.006 | 0.048 | 0.036 | 0.013 | 0.0008 | 356 | 1032 | 454 | 597 |
| 0.074 | 0.27 | 1.47 | 0.008 | 0.002 | 0.168 | 0.001 | 0.136 | 0.095 | 0.004 | 0.048 | 0.037 | 0.012 | 0.0004 | 414 | 1186 | 491 | 600 |
| 0.081 | 0.24 | 1.40 | 0.009 | 0.002 | 0.143 | 0.002 | 0.015 | 0.089 | 0.006 | 0.048 | 0.038 | 0.012 | 0.0005 | 431 | 1186 | 493 | 597 |
| 0.075 | 0.25 | 1.45 | 0.009 | 0.003 | 0.013 | 0.001 | 0.098 | 0.076 | 0.003 | 0.048 | 0.037 | 0.012 | 0.0022 | 418 | 1183 | 487 | 590 |
| 0.081 | 0.22 | 1.47 | 0.009 | 0.003 | 0.105 | 0.001 | 0.014 | 0.068 | 0.004 | 0.049 | 0.043 | 0.014 | 0.0014 | 428 | 1196 | 496 | 597 |
| 0.081 | 0.27 | 1.32 | 0.009 | 0.002 | 0.143 | 0.001 | 0.014 | 0.072 | 0.004 | 0.048 | 0.035 | 0.013 | 0.0002 | 421 | 1030 | 483 | 597 |
| 0.075 | 0.26 | 1.47 | 0.010 | 0.004 | 0.034 | 0.002 | 0.017 | 0.071 | 0.002 | 0.049 | 0.043 | 0.012 | 0.0012 | 342 | 1189 | 482 | 597 |
| 0.074 | 0.27 | 1.45 | 0.009 | 0.004 | 0.149 | 0.001 | 0.079 | 0.071 | 0.004 | 0.049 | 0.044 | 0.013 | 0.0004 | 451 | 1180 | 502 | 607 |
| 0.081 | 0.29 | 1.35 | 0.009 | 0.001 | 0.108 | 0.002 | 0.090 | 0.071 | 0.004 | 0.049 | 0.040 | 0.013 | 0.0014 | 425 | 1082 | 473 | 598 |
| 0.075 | 0.24 | 1.45 | 0.009 | 0.004 | 0.070 | 0.001 | 0.009 | 0.065 | 0.005 | 0.049 | 0.039 | 0.012 | 0.0013 | 457 | 1186 | 506 | 606 |
| 0.082 | 0.24 | 1.36 | 0.009 | 0.002 | 0.152 | 0.002 | 0.069 | 0.098 | 0.002 | 0.030 | 0.043 | 0.016 | 0.0016 | 460 | 1225 | 476 | 588 |
| 0.077 | 0.25 | 1.46 | 0.012 | 0.004 | 0.035 | 0.002 | 0.081 | 0.074 | 0.002 | 0.047 | 0.038 | 0.013 | 0.0011 | 397 | 1183 | 485 | 598 |
| 0.075 | 0.29 | 1.45 | 0.009 | 0.003 | 0.101 | 0.002 | 0.077 | 0.073 | 0.001 | 0.048 | 0.044 | 0.011 | 0.0004 | 426 | 1185 | 484 | 596 |
| 0.075 | 0.27 | 1.47 | 0.009 | 0.004 | 0.148 | 0.001 | 0.088 | 0.053 | 0.004 | 0.048 | 0.041 | 0.012 | 0.0004 | 400 | 1186 | 505 | 602 |
| 0.074 | 0.28 | 1.29 | 0.006 | 0.001 | 0.106 | 0.002 | 0.078 | 0.095 | 0.004 | 0.048 | 0.044 | 0.011 | 0.0004 | 416 | 1213 | 483 | 585 |
| 0.081 | 0.22 | 1.37 | 0.009 | 0.002 | 0.015 | 0.003 | 0.063 | 0.079 | 0.007 | 0.049 | 0.039 | 0.016 | 0.0019 | 428 | 1187 | 493 | 597 |
| 0.078 | 0.21 | 1.25 | 0.008 | 0.002 | 0.165 | 0.001 | 0.116 | 0.060 | 0.005 | 0.033 | 0.039 | 0.016 | 0.0018 | 418 | 1185 | 500 | 574 |
| 0.075 | 0.22 | 1.37 | 0.009 | 0.002 | 0.143 | 0.001 | 0.047 | 0.083 | 0.004 | 0.035 | 0.039 | 0.016 | 0.0018 | 424 | 1187 | 483 | 587 |
| 0.075 | 0.24 | 1.50 | 0.009 | 0.003 | 0.076 | 0.003 | 0.027 | 0.088 | 0.002 | 0.048 | 0.039 | 0.012 | 0.0024 | 433 | 1186 | 495 | 593 |
| 0.082 | 0.29 | 1.47 | 0.013 | 0.001 | 0.044 | 0.001 | 0.099 | 0.047 | 0.008 | 0.049 | 0.040 | 0.015 | 0.0005 | 328 | 1160 | 492 | 599 |
| 0.075 | 0.27 | 1.48 | 0.009 | 0.002 | 0.164 | 0.001 | 0.056 | 0.083 | 0.003 | 0.048 | 0.036 | 0.011 | 0.0028 | 416 | 1185 | 487 | 598 |
| 0.075 | 0.27 | 1.38 | 0.009 | 0.003 | 0.146 | 0.002 | 0.071 | 0.140 | 0.003 | 0.022 | 0.037 | 0.014 | 0.0004 | 424 | 1212 | 470 | 577 |
| 0.081 | 0.28 | 1.35 | 0.010 | 0.002 | 0.146 | 0.001 | 0.019 | 0.091 | 0.004 | 0.048 | 0.036 | 0.012 | 0.0003 | 416 | 1097 | 478 | 603 |
| 0.084 | 0.24 | 1.41 | 0.009 | 0.003 | 0.060 | 0.002 | 0.092 | 0.088 | 0.004 | 0.048 | 0.037 | 0.014 | 0.0021 | 398 | 1191 | 484 | 590 |
| 0.081 | 0.22 | 1.23 | 0.009 | 0.002 | 0.043 | 0.001 | 0.072 | 0.064 | 0.001 | 0.049 | 0.036 | 0.011 | 0.0003 | 425 | 1215 | 474 | 595 |
| 0.081 | 0.27 | 1.48 | 0.012 | 0.001 | 0.017 | 0.001 | 0.084 | 0.055 | 0.006 | 0.048 | 0.042 | 0.013 | 0.0015 | 421 | 1110 | 460 | 612 |
| 0.075 | 0.26 | 1.45 | 0.009 | 0.004 | 0.093 | 0.002 | 0.093 | 0.088 | 0.003 | 0.048 | 0.038 | 0.012 | 0.0004 | 400 | 1186 | 498 | 603 |
| 0.082 | 0.22 | 1.32 | 0.009 | 0.002 | 0.146 | 0.003 | 0.059 | 0.090 | 0.003 | 0.030 | 0.037 | 0.016 | 0.0018 | 414 | 1224 | 488 | 586 |
| 0.075 | 0.28 | 1.46 | 0.013 | 0.004 | 0.023 | 0.001 | 0.012 | 0.097 | 0.005 | 0.049 | 0.038 | 0.012 | 0.0007 | 337 | 1180 | 504 | 614 |
| 0.082 | 0.29 | 1.35 | 0.010 | 0.001 | 0.168 | 0.002 | 0.057 | 0.094 | 0.003 | 0.048 | 0.038 | 0.012 | 0.0009 | 400 | 1084 | 484 | 596 |
| 0.081 | 0.24 | 1.40 | 0.009 | 0.002 | 0.017 | 0.001 | 0.100 | 0.092 | 0.005 | 0.048 | 0.037 | 0.011 | 0.0018 | 453 | 1186 | 496 | 599 |
| 0.073 | 0.22 | 1.39 | 0.009 | 0.002 | 0.145 | 0.003 | 0.088 | 0.095 | 0.003 | 0.035 | 0.037 | 0.016 | 0.0019 | 425 | 1191 | 493 | 586 |
| 0.082 | 0.27 | 1.35 | 0.009 | 0.001 | 0.172 | 0.002 | 0.087 | 0.071 | 0.004 | 0.048 | 0.037 | 0.013 | 0.0010 | 412 | 1084 | 478 | 600 |
| 0.081 | 0.27 | 1.45 | 0.009 | 0.003 | 0.073 | 0.001 | 0.022 | 0.064 | 0.007 | 0.049 | 0.036 | 0.011 | 0.0004 | 434 | 1192 | 482 | 597 |
| 0.081 | 0.30 | 1.43 | 0.013 | 0.003 | 0.013 | 0.003 | 0.103 | 0.074 | 0.005 | 0.037 | 0.040 | 0.015 | 0.0011 | 393 | 1209 | 463 | 580 |
| 0.082 | 0.25 | 1.46 | 0.012 | 0.004 | 0.016 | 0.001 | 0.044 | 0.082 | 0.005 | 0.048 | 0.039 | 0.012 | 0.0005 | 452 | 1160 | 482 | 610 |
| 0.075 | 0.24 | 1.16 | 0.006 | 0.002 | 0.145 | 0.002 | 0.082 | 0.085 | 0.005 | 0.029 | 0.044 | 0.016 | 0.0015 | 400 | 1224 | 495 | 581 |
| 0.074 | 0.29 | 1.48 | 0.005 | 0.002 | 0.154 | 0.003 | 0.058 | 0.082 | 0.004 | 0.048 | 0.044 | 0.012 | 0.0004 | 422 | 1186 | 489 | 596 |
| 0.081 | 0.30 | 1.46 | 0.009 | 0.002 | 0.149 | 0.001 | 0.069 | 0.087 | 0.006 | 0.050 | 0.039 | 0.012 | 0.0004 | 434 | 1183 | 487 | 605 |
| 0.082 | 0.29 | 1.43 | 0.013 | 0.003 | 0.083 | 0.003 | 0.076 | 0.118 | 0.006 | 0.038 | 0.037 | 0.014 | 0.0004 | 442 | 1212 | 454 | 581 |
| 0.074 | 0.27 | 1.43 | 0.010 | 0.004 | 0.096 | 0.001 | 0.086 | 0.102 | 0.003 | 0.048 | 0.037 | 0.015 | 0.0007 | 342 | 1159 | 469 | 605 |
| 0.076 | 0.27 | 1.40 | 0.007 | 0.001 | 0.163 | 0.001 | 0.074 | 0.106 | 0.002 | 0.048 | 0.037 | 0.012 | 0.0004 | 437 | 1186 | 494 | 589 |
| 0.078 | 0.27 | 1.15 | 0.009 | 0.002 | 0.144 | 0.002 | 0.012 | 0.046 | 0.003 | 0.050 | 0.043 | 0.011 | 0.0012 | 418 | 1220 | 485 | 576 |
| 0.081 | 0.21 | 1.39 | 0.009 | 0.004 | 0.118 | 0.001 | 0.026 | 0.041 | 0.006 | 0.049 | 0.040 | 0.013 | 0.0005 | 426 | 1191 | 497 | 605 |
| 0.073 | 0.22 | 1.17 | 0.007 | 0.001 | 0.146 | 0.003 | 0.117 | 0.045 | 0.006 | 0.049 | 0.038 | 0.016 | 0.0005 | 461 | 1193 | 501 | 587 |
| 0.082 | 0.24 | 1.48 | 0.009 | 0.003 | 0.050 | 0.003 | 0.087 | 0.070 | 0.003 | 0.049 | 0.037 | 0.012 | 0.0021 | 454 | 1187 | 495 | 597 |
| 0.075 | 0.29 | 1.39 | 0.009 | 0.004 | 0.053 | 0.001 | 0.097 | 0.065 | 0.005 | 0.047 | 0.039 | 0.014 | 0.0004 | 411 | 1186 | 498 | 600 |
| 0.083 | 0.28 | 1.44 | 0.013 | 0.001 | 0.021 | 0.001 | 0.084 | 0.110 | 0.005 | 0.048 | 0.039 | 0.012 | 0.0003 | 422 | 1084 | 469 | 608 |
| 0.075 | 0.29 | 1.48 | 0.010 | 0.003 | 0.140 | 0.001 | 0.131 | 0.060 | 0.005 | 0.048 | 0.038 | 0.013 | 0.0003 | 437 | 1152 | 478 | 595 |
| 0.081 | 0.29 | 1.47 | 0.012 | 0.003 | 0.058 | 0.001 | 0.131 | 0.037 | 0.005 | 0.049 | 0.037 | 0.012 | 0.0004 | 333 | 1182 | 486 | 603 |
| 0.081 | 0.24 | 1.46 | 0.012 | 0.003 | 0.049 | 0.001 | 0.129 | 0.049 | 0.007 | 0.048 | 0.038 | 0.012 | 0.0005 | 447 | 1183 | 478 | 611 |
| 0.081 | 0.28 | 1.27 | 0.012 | 0.001 | 0.029 | 0.002 | 0.081 | 0.075 | 0.004 | 0.049 | 0.036 | 0.013 | 0.0003 | 420 | 1082 | 474 | 600 |
| 0.083 | 0.27 | 1.45 | 0.009 | 0.003 | 0.012 | 0.002 | 0.045 | 0.060 | 0.003 | 0.048 | 0.039 | 0.014 | 0.0026 | 418 | 1153 | 477 | 605 |
| 0.081 | 0.22 | 1.15 | 0.009 | 0.002 | 0.154 | 0.002 | 0.066 | 0.057 | 0.005 | 0.034 | 0.038 | 0.016 | 0.0023 | 415 | 1166 | 499 | 585 |
|  |  |  |  |  |  |  |  |  |  |  |  |  |  |  |  |  |  |
| **SVR** | | | | | | | | | | | | | | | | | |
| C | Si | Mn | P | S | Cu | Sn | Ni | Cr | Mo | V | Nb | Ti | Ca | Time | Temp | YS | UTS |
| (wt%) | (wt%) | (wt%) | (wt%) | (wt%) | (wt%) | (wt%) | (wt%) | (wt%) | (wt%) | (wt%) | (wt%) | (wt%) | (wt%) | (s) | (℃) | (Mpa) | (Mpa) |
| 0.080 | 0.25 | 1.43 | 0.013 | 0.003 | 0.026 | 0.001 | 0.007 | 0.041 | 0.002 | 0.047 | 0.040 | 0.013 | 0.0004 | 390 | 1169 | 477 | 594 |
| 0.079 | 0.25 | 1.43 | 0.013 | 0.004 | 0.031 | 0.001 | 0.014 | 0.040 | 0.001 | 0.048 | 0.040 | 0.014 | 0.0005 | 384 | 1202 | 478 | 590 |
| 0.080 | 0.25 | 1.42 | 0.013 | 0.003 | 0.028 | 0.001 | 0.006 | 0.041 | 0.001 | 0.048 | 0.040 | 0.013 | 0.0005 | 389 | 1203 | 479 | 592 |
| 0.080 | 0.25 | 1.42 | 0.013 | 0.003 | 0.029 | 0.001 | 0.009 | 0.041 | 0.002 | 0.046 | 0.040 | 0.013 | 0.0005 | 389 | 1210 | 478 | 589 |
| 0.079 | 0.24 | 1.43 | 0.013 | 0.003 | 0.029 | 0.001 | 0.009 | 0.043 | 0.002 | 0.047 | 0.039 | 0.013 | 0.0004 | 382 | 1206 | 478 | 589 |
| 0.080 | 0.25 | 1.44 | 0.013 | 0.003 | 0.029 | 0.001 | 0.007 | 0.039 | 0.001 | 0.047 | 0.040 | 0.013 | 0.0005 | 398 | 1190 | 477 | 592 |
| 0.081 | 0.25 | 1.43 | 0.013 | 0.003 | 0.026 | 0.001 | 0.019 | 0.039 | 0.001 | 0.047 | 0.040 | 0.013 | 0.0004 | 388 | 1187 | 478 | 593 |
| 0.081 | 0.25 | 1.42 | 0.013 | 0.003 | 0.027 | 0.001 | 0.013 | 0.041 | 0.002 | 0.047 | 0.039 | 0.013 | 0.0004 | 392 | 1189 | 477 | 593 |
| 0.080 | 0.26 | 1.42 | 0.013 | 0.003 | 0.031 | 0.001 | 0.017 | 0.040 | 0.001 | 0.045 | 0.040 | 0.013 | 0.0005 | 390 | 1196 | 476 | 590 |
| 0.080 | 0.25 | 1.41 | 0.013 | 0.003 | 0.030 | 0.001 | 0.005 | 0.041 | 0.001 | 0.047 | 0.041 | 0.013 | 0.0004 | 385 | 1188 | 478 | 591 |
| 0.081 | 0.25 | 1.42 | 0.013 | 0.003 | 0.032 | 0.001 | 0.012 | 0.042 | 0.001 | 0.047 | 0.040 | 0.013 | 0.0003 | 394 | 1191 | 476 | 591 |
| 0.079 | 0.24 | 1.42 | 0.013 | 0.003 | 0.028 | 0.001 | 0.013 | 0.041 | 0.001 | 0.048 | 0.041 | 0.013 | 0.0004 | 379 | 1215 | 478 | 587 |
| 0.080 | 0.24 | 1.40 | 0.013 | 0.003 | 0.034 | 0.001 | 0.008 | 0.041 | 0.002 | 0.048 | 0.040 | 0.013 | 0.0005 | 386 | 1203 | 477 | 589 |
| 0.081 | 0.25 | 1.43 | 0.013 | 0.004 | 0.029 | 0.001 | 0.011 | 0.042 | 0.001 | 0.048 | 0.040 | 0.013 | 0.0005 | 385 | 1188 | 479 | 593 |
| 0.080 | 0.25 | 1.42 | 0.013 | 0.003 | 0.026 | 0.001 | 0.007 | 0.041 | 0.002 | 0.048 | 0.040 | 0.013 | 0.0005 | 391 | 1177 | 478 | 593 |
| 0.080 | 0.25 | 1.42 | 0.013 | 0.003 | 0.028 | 0.001 | 0.006 | 0.042 | 0.001 | 0.047 | 0.039 | 0.013 | 0.0004 | 386 | 1202 | 478 | 592 |
| 0.080 | 0.25 | 1.43 | 0.013 | 0.003 | 0.025 | 0.001 | 0.011 | 0.040 | 0.001 | 0.046 | 0.040 | 0.013 | 0.0005 | 400 | 1187 | 477 | 593 |
| 0.079 | 0.25 | 1.44 | 0.013 | 0.004 | 0.033 | 0.001 | 0.008 | 0.039 | 0.002 | 0.048 | 0.040 | 0.013 | 0.0004 | 386 | 1207 | 479 | 590 |
| 0.081 | 0.25 | 1.44 | 0.013 | 0.003 | 0.026 | 0.001 | 0.012 | 0.040 | 0.001 | 0.049 | 0.040 | 0.013 | 0.0004 | 388 | 1177 | 477 | 593 |
| 0.080 | 0.25 | 1.44 | 0.013 | 0.003 | 0.030 | 0.001 | 0.005 | 0.041 | 0.001 | 0.049 | 0.039 | 0.013 | 0.0004 | 381 | 1192 | 479 | 593 |
| 0.080 | 0.25 | 1.43 | 0.013 | 0.003 | 0.028 | 0.001 | 0.005 | 0.042 | 0.001 | 0.047 | 0.039 | 0.013 | 0.0004 | 391 | 1197 | 478 | 593 |
| 0.080 | 0.25 | 1.43 | 0.013 | 0.004 | 0.023 | 0.001 | 0.009 | 0.041 | 0.001 | 0.047 | 0.040 | 0.013 | 0.0004 | 388 | 1181 | 479 | 595 |
| 0.079 | 0.26 | 1.43 | 0.013 | 0.003 | 0.024 | 0.001 | 0.004 | 0.037 | 0.001 | 0.045 | 0.038 | 0.013 | 0.0005 | 386 | 1190 | 477 | 591 |
| 0.081 | 0.25 | 1.43 | 0.013 | 0.004 | 0.027 | 0.001 | 0.016 | 0.043 | 0.002 | 0.048 | 0.039 | 0.013 | 0.0005 | 398 | 1183 | 477 | 594 |
| 0.079 | 0.25 | 1.43 | 0.013 | 0.004 | 0.027 | 0.001 | 0.009 | 0.040 | 0.001 | 0.047 | 0.039 | 0.013 | 0.0004 | 387 | 1192 | 479 | 594 |
| 0.080 | 0.25 | 1.44 | 0.013 | 0.003 | 0.024 | 0.001 | 0.006 | 0.042 | 0.001 | 0.046 | 0.041 | 0.013 | 0.0004 | 384 | 1192 | 478 | 593 |
| 0.080 | 0.25 | 1.43 | 0.013 | 0.003 | 0.032 | 0.001 | 0.009 | 0.040 | 0.001 | 0.047 | 0.039 | 0.013 | 0.0005 | 384 | 1182 | 478 | 594 |
| 0.079 | 0.25 | 1.42 | 0.013 | 0.003 | 0.029 | 0.001 | 0.017 | 0.040 | 0.001 | 0.047 | 0.040 | 0.013 | 0.0004 | 396 | 1192 | 477 | 592 |
| 0.080 | 0.25 | 1.42 | 0.013 | 0.003 | 0.027 | 0.001 | 0.008 | 0.038 | 0.001 | 0.047 | 0.041 | 0.013 | 0.0005 | 378 | 1192 | 478 | 589 |
| 0.080 | 0.25 | 1.44 | 0.013 | 0.003 | 0.028 | 0.001 | 0.008 | 0.044 | 0.001 | 0.048 | 0.040 | 0.013 | 0.0003 | 389 | 1171 | 477 | 594 |
| 0.081 | 0.25 | 1.43 | 0.013 | 0.003 | 0.026 | 0.001 | 0.010 | 0.043 | 0.001 | 0.048 | 0.039 | 0.013 | 0.0005 | 384 | 1196 | 478 | 592 |
| 0.079 | 0.25 | 1.44 | 0.013 | 0.003 | 0.032 | 0.001 | 0.009 | 0.042 | 0.001 | 0.047 | 0.040 | 0.014 | 0.0004 | 382 | 1193 | 477 | 589 |
| 0.081 | 0.26 | 1.42 | 0.013 | 0.003 | 0.029 | 0.001 | 0.011 | 0.040 | 0.001 | 0.048 | 0.040 | 0.013 | 0.0006 | 390 | 1187 | 477 | 592 |
| 0.080 | 0.25 | 1.43 | 0.013 | 0.003 | 0.027 | 0.001 | 0.002 | 0.041 | 0.001 | 0.047 | 0.040 | 0.013 | 0.0004 | 386 | 1186 | 478 | 593 |
| 0.081 | 0.25 | 1.43 | 0.013 | 0.003 | 0.027 | 0.001 | 0.003 | 0.044 | 0.001 | 0.049 | 0.039 | 0.013 | 0.0004 | 394 | 1181 | 478 | 595 |
| 0.079 | 0.26 | 1.43 | 0.013 | 0.003 | 0.028 | 0.001 | 0.004 | 0.045 | 0.001 | 0.046 | 0.040 | 0.013 | 0.0004 | 383 | 1195 | 478 | 591 |
| 0.080 | 0.25 | 1.42 | 0.013 | 0.003 | 0.030 | 0.001 | 0.005 | 0.042 | 0.001 | 0.047 | 0.040 | 0.013 | 0.0004 | 386 | 1185 | 478 | 592 |
| 0.080 | 0.25 | 1.43 | 0.014 | 0.003 | 0.027 | 0.001 | 0.005 | 0.035 | 0.001 | 0.048 | 0.040 | 0.013 | 0.0004 | 389 | 1187 | 477 | 592 |
| 0.080 | 0.25 | 1.42 | 0.013 | 0.003 | 0.029 | 0.001 | 0.012 | 0.041 | 0.001 | 0.046 | 0.040 | 0.013 | 0.0005 | 382 | 1186 | 478 | 592 |
| 0.080 | 0.25 | 1.42 | 0.013 | 0.003 | 0.028 | 0.001 | 0.008 | 0.040 | 0.001 | 0.049 | 0.040 | 0.013 | 0.0005 | 386 | 1187 | 477 | 592 |
| 0.080 | 0.26 | 1.44 | 0.013 | 0.003 | 0.029 | 0.001 | 0.007 | 0.041 | 0.001 | 0.048 | 0.039 | 0.013 | 0.0004 | 391 | 1192 | 478 | 593 |
| 0.078 | 0.25 | 1.41 | 0.013 | 0.003 | 0.029 | 0.001 | 0.017 | 0.039 | 0.001 | 0.048 | 0.040 | 0.014 | 0.0006 | 387 | 1215 | 478 | 586 |
| 0.080 | 0.25 | 1.42 | 0.013 | 0.003 | 0.028 | 0.001 | 0.011 | 0.040 | 0.001 | 0.046 | 0.040 | 0.013 | 0.0004 | 382 | 1195 | 478 | 591 |
| 0.079 | 0.25 | 1.43 | 0.013 | 0.003 | 0.029 | 0.001 | 0.026 | 0.040 | 0.001 | 0.047 | 0.040 | 0.014 | 0.0005 | 383 | 1214 | 476 | 587 |
| 0.080 | 0.26 | 1.43 | 0.013 | 0.003 | 0.030 | 0.001 | 0.007 | 0.040 | 0.001 | 0.045 | 0.039 | 0.014 | 0.0004 | 387 | 1194 | 477 | 590 |
| 0.079 | 0.25 | 1.43 | 0.013 | 0.003 | 0.027 | 0.001 | 0.004 | 0.043 | 0.001 | 0.049 | 0.040 | 0.013 | 0.0005 | 382 | 1209 | 479 | 591 |
| 0.080 | 0.25 | 1.42 | 0.013 | 0.003 | 0.031 | 0.001 | 0.008 | 0.043 | 0.001 | 0.048 | 0.039 | 0.013 | 0.0004 | 381 | 1197 | 479 | 591 |
| 0.080 | 0.25 | 1.43 | 0.013 | 0.003 | 0.027 | 0.001 | 0.006 | 0.036 | 0.001 | 0.047 | 0.039 | 0.013 | 0.0004 | 385 | 1197 | 479 | 592 |
| 0.080 | 0.25 | 1.42 | 0.013 | 0.003 | 0.026 | 0.001 | 0.020 | 0.036 | 0.001 | 0.046 | 0.039 | 0.013 | 0.0005 | 383 | 1192 | 477 | 591 |
| 0.080 | 0.25 | 1.43 | 0.013 | 0.003 | 0.027 | 0.001 | 0.008 | 0.039 | 0.001 | 0.045 | 0.040 | 0.013 | 0.0004 | 379 | 1199 | 478 | 591 |
| 0.080 | 0.25 | 1.43 | 0.013 | 0.003 | 0.027 | 0.001 | 0.018 | 0.036 | 0.001 | 0.047 | 0.040 | 0.013 | 0.0005 | 379 | 1195 | 478 | 591 |
| 0.080 | 0.25 | 1.43 | 0.013 | 0.003 | 0.026 | 0.001 | 0.009 | 0.042 | 0.001 | 0.048 | 0.040 | 0.013 | 0.0005 | 386 | 1197 | 478 | 592 |
| 0.080 | 0.25 | 1.43 | 0.013 | 0.003 | 0.023 | 0.001 | 0.013 | 0.044 | 0.001 | 0.048 | 0.039 | 0.013 | 0.0005 | 391 | 1191 | 478 | 594 |
| 0.079 | 0.25 | 1.42 | 0.013 | 0.003 | 0.036 | 0.001 | 0.007 | 0.042 | 0.002 | 0.044 | 0.040 | 0.014 | 0.0005 | 380 | 1199 | 477 | 587 |
| 0.080 | 0.25 | 1.43 | 0.013 | 0.003 | 0.032 | 0.001 | 0.005 | 0.044 | 0.001 | 0.048 | 0.039 | 0.013 | 0.0004 | 393 | 1174 | 476 | 592 |
| 0.080 | 0.26 | 1.43 | 0.013 | 0.003 | 0.029 | 0.001 | 0.008 | 0.038 | 0.001 | 0.047 | 0.041 | 0.013 | 0.0004 | 384 | 1198 | 479 | 592 |
| 0.080 | 0.26 | 1.43 | 0.013 | 0.003 | 0.029 | 0.001 | 0.010 | 0.038 | 0.001 | 0.047 | 0.040 | 0.013 | 0.0004 | 395 | 1191 | 478 | 594 |
| 0.080 | 0.26 | 1.41 | 0.013 | 0.003 | 0.028 | 0.001 | 0.010 | 0.045 | 0.001 | 0.045 | 0.040 | 0.013 | 0.0006 | 395 | 1194 | 476 | 589 |
| 0.080 | 0.25 | 1.42 | 0.013 | 0.004 | 0.028 | 0.001 | 0.023 | 0.041 | 0.001 | 0.045 | 0.041 | 0.013 | 0.0005 | 376 | 1210 | 478 | 587 |
| 0.080 | 0.25 | 1.43 | 0.013 | 0.003 | 0.025 | 0.001 | 0.011 | 0.041 | 0.001 | 0.047 | 0.039 | 0.013 | 0.0004 | 395 | 1187 | 477 | 592 |
| 0.080 | 0.25 | 1.43 | 0.013 | 0.003 | 0.027 | 0.001 | 0.003 | 0.040 | 0.001 | 0.046 | 0.040 | 0.013 | 0.0005 | 378 | 1195 | 479 | 592 |
| 0.080 | 0.25 | 1.43 | 0.013 | 0.003 | 0.028 | 0.001 | 0.012 | 0.040 | 0.001 | 0.045 | 0.040 | 0.013 | 0.0003 | 386 | 1200 | 478 | 591 |
| 0.079 | 0.24 | 1.42 | 0.013 | 0.004 | 0.027 | 0.001 | 0.016 | 0.041 | 0.002 | 0.047 | 0.040 | 0.013 | 0.0004 | 396 | 1185 | 476 | 590 |
| 0.079 | 0.25 | 1.42 | 0.013 | 0.003 | 0.029 | 0.001 | 0.009 | 0.042 | 0.001 | 0.047 | 0.040 | 0.013 | 0.0005 | 383 | 1212 | 479 | 589 |
| 0.080 | 0.25 | 1.43 | 0.013 | 0.003 | 0.031 | 0.001 | 0.014 | 0.041 | 0.001 | 0.046 | 0.040 | 0.013 | 0.0005 | 386 | 1201 | 478 | 589 |
| 0.078 | 0.25 | 1.43 | 0.013 | 0.003 | 0.031 | 0.001 | 0.010 | 0.040 | 0.002 | 0.047 | 0.040 | 0.013 | 0.0004 | 389 | 1215 | 478 | 588 |
| 0.080 | 0.25 | 1.41 | 0.013 | 0.003 | 0.029 | 0.001 | 0.008 | 0.043 | 0.001 | 0.048 | 0.040 | 0.013 | 0.0005 | 381 | 1202 | 478 | 590 |
| 0.079 | 0.25 | 1.45 | 0.013 | 0.003 | 0.026 | 0.001 | 0.011 | 0.041 | 0.001 | 0.047 | 0.040 | 0.013 | 0.0004 | 389 | 1201 | 478 | 592 |
| 0.078 | 0.25 | 1.45 | 0.013 | 0.003 | 0.029 | 0.001 | 0.016 | 0.038 | 0.001 | 0.046 | 0.039 | 0.014 | 0.0005 | 391 | 1184 | 476 | 590 |
| 0.080 | 0.26 | 1.42 | 0.013 | 0.003 | 0.027 | 0.001 | 0.009 | 0.040 | 0.001 | 0.048 | 0.040 | 0.013 | 0.0004 | 383 | 1201 | 479 | 591 |
| 0.080 | 0.25 | 1.43 | 0.013 | 0.003 | 0.021 | 0.001 | 0.009 | 0.037 | 0.001 | 0.048 | 0.039 | 0.013 | 0.0004 | 380 | 1185 | 479 | 595 |
| 0.079 | 0.24 | 1.42 | 0.013 | 0.003 | 0.032 | 0.001 | 0.008 | 0.041 | 0.001 | 0.048 | 0.040 | 0.013 | 0.0005 | 386 | 1201 | 478 | 590 |
| 0.081 | 0.25 | 1.41 | 0.013 | 0.003 | 0.023 | 0.001 | 0.010 | 0.042 | 0.002 | 0.045 | 0.040 | 0.013 | 0.0003 | 382 | 1196 | 478 | 590 |
| 0.080 | 0.25 | 1.43 | 0.013 | 0.003 | 0.021 | 0.001 | 0.007 | 0.043 | 0.001 | 0.044 | 0.040 | 0.013 | 0.0005 | 394 | 1205 | 477 | 590 |
| 0.081 | 0.25 | 1.42 | 0.013 | 0.004 | 0.027 | 0.001 | 0.005 | 0.042 | 0.001 | 0.047 | 0.040 | 0.013 | 0.0004 | 386 | 1178 | 478 | 594 |
| 0.081 | 0.25 | 1.42 | 0.013 | 0.003 | 0.025 | 0.001 | 0.008 | 0.041 | 0.001 | 0.044 | 0.041 | 0.013 | 0.0005 | 391 | 1188 | 476 | 590 |
| 0.080 | 0.25 | 1.43 | 0.013 | 0.003 | 0.028 | 0.001 | 0.010 | 0.035 | 0.002 | 0.048 | 0.039 | 0.013 | 0.0004 | 384 | 1182 | 478 | 592 |
| 0.079 | 0.25 | 1.43 | 0.013 | 0.003 | 0.029 | 0.001 | 0.014 | 0.040 | 0.002 | 0.047 | 0.040 | 0.013 | 0.0004 | 374 | 1198 | 478 | 589 |
| 0.080 | 0.25 | 1.41 | 0.013 | 0.003 | 0.026 | 0.001 | 0.009 | 0.043 | 0.001 | 0.045 | 0.040 | 0.013 | 0.0004 | 385 | 1198 | 477 | 590 |
| 0.079 | 0.26 | 1.44 | 0.013 | 0.004 | 0.030 | 0.001 | 0.004 | 0.038 | 0.001 | 0.048 | 0.039 | 0.013 | 0.0004 | 390 | 1195 | 480 | 594 |
| 0.080 | 0.25 | 1.42 | 0.013 | 0.003 | 0.028 | 0.001 | 0.009 | 0.037 | 0.001 | 0.049 | 0.039 | 0.013 | 0.0004 | 387 | 1187 | 478 | 594 |
| 0.078 | 0.26 | 1.42 | 0.013 | 0.003 | 0.032 | 0.001 | 0.009 | 0.044 | 0.002 | 0.046 | 0.040 | 0.013 | 0.0005 | 388 | 1217 | 477 | 587 |
| 0.079 | 0.25 | 1.43 | 0.013 | 0.003 | 0.028 | 0.001 | 0.015 | 0.041 | 0.001 | 0.046 | 0.040 | 0.014 | 0.0005 | 390 | 1204 | 478 | 590 |
| 0.079 | 0.26 | 1.41 | 0.013 | 0.003 | 0.026 | 0.001 | 0.015 | 0.039 | 0.002 | 0.048 | 0.039 | 0.013 | 0.0006 | 384 | 1205 | 477 | 588 |
| 0.080 | 0.26 | 1.43 | 0.013 | 0.003 | 0.029 | 0.001 | 0.006 | 0.041 | 0.002 | 0.046 | 0.040 | 0.013 | 0.0005 | 386 | 1201 | 478 | 590 |
| 0.081 | 0.25 | 1.42 | 0.013 | 0.003 | 0.027 | 0.001 | 0.010 | 0.044 | 0.001 | 0.047 | 0.040 | 0.013 | 0.0004 | 386 | 1188 | 478 | 592 |
| 0.079 | 0.26 | 1.42 | 0.013 | 0.003 | 0.030 | 0.001 | 0.011 | 0.038 | 0.001 | 0.047 | 0.040 | 0.013 | 0.0005 | 384 | 1192 | 477 | 590 |
| 0.081 | 0.25 | 1.44 | 0.013 | 0.003 | 0.028 | 0.001 | 0.004 | 0.044 | 0.001 | 0.044 | 0.040 | 0.013 | 0.0004 | 391 | 1188 | 477 | 592 |
| 0.080 | 0.25 | 1.43 | 0.013 | 0.003 | 0.027 | 0.001 | 0.007 | 0.042 | 0.001 | 0.047 | 0.039 | 0.014 | 0.0005 | 390 | 1215 | 478 | 590 |
| 0.080 | 0.25 | 1.42 | 0.013 | 0.003 | 0.036 | 0.001 | 0.003 | 0.042 | 0.002 | 0.048 | 0.041 | 0.014 | 0.0004 | 383 | 1206 | 478 | 588 |
| 0.080 | 0.25 | 1.43 | 0.013 | 0.003 | 0.028 | 0.001 | 0.009 | 0.040 | 0.001 | 0.047 | 0.040 | 0.013 | 0.0004 | 402 | 1177 | 477 | 595 |
| 0.080 | 0.24 | 1.44 | 0.013 | 0.003 | 0.033 | 0.001 | 0.006 | 0.041 | 0.001 | 0.046 | 0.040 | 0.013 | 0.0005 | 385 | 1192 | 478 | 592 |
| 0.079 | 0.25 | 1.41 | 0.013 | 0.003 | 0.028 | 0.001 | 0.013 | 0.042 | 0.001 | 0.047 | 0.040 | 0.013 | 0.0005 | 371 | 1207 | 479 | 588 |
| 0.080 | 0.25 | 1.45 | 0.013 | 0.004 | 0.028 | 0.001 | 0.016 | 0.042 | 0.002 | 0.047 | 0.040 | 0.013 | 0.0005 | 387 | 1188 | 477 | 592 |
| 0.079 | 0.26 | 1.43 | 0.013 | 0.003 | 0.028 | 0.001 | 0.011 | 0.040 | 0.002 | 0.047 | 0.040 | 0.013 | 0.0004 | 390 | 1188 | 477 | 591 |
| 0.079 | 0.26 | 1.44 | 0.013 | 0.003 | 0.028 | 0.001 | 0.007 | 0.040 | 0.001 | 0.046 | 0.039 | 0.013 | 0.0004 | 389 | 1193 | 477 | 591 |
| 0.079 | 0.25 | 1.44 | 0.013 | 0.003 | 0.037 | 0.001 | 0.015 | 0.038 | 0.002 | 0.047 | 0.040 | 0.014 | 0.0004 | 386 | 1198 | 477 | 589 |
| 0.080 | 0.25 | 1.43 | 0.013 | 0.003 | 0.024 | 0.001 | 0.005 | 0.037 | 0.001 | 0.046 | 0.040 | 0.013 | 0.0005 | 382 | 1193 | 479 | 592 |
| 0.080 | 0.25 | 1.43 | 0.013 | 0.003 | 0.029 | 0.001 | 0.006 | 0.040 | 0.002 | 0.048 | 0.040 | 0.013 | 0.0005 | 379 | 1196 | 479 | 591 |
| 0.079 | 0.25 | 1.41 | 0.013 | 0.003 | 0.029 | 0.001 | 0.009 | 0.038 | 0.001 | 0.046 | 0.039 | 0.013 | 0.0006 | 379 | 1206 | 478 | 589 |
| 0.080 | 0.25 | 1.43 | 0.013 | 0.003 | 0.033 | 0.001 | 0.010 | 0.043 | 0.001 | 0.046 | 0.040 | 0.013 | 0.0004 | 380 | 1195 | 477 | 589 |
| 0.080 | 0.26 | 1.43 | 0.013 | 0.004 | 0.026 | 0.001 | 0.010 | 0.042 | 0.001 | 0.047 | 0.039 | 0.013 | 0.0005 | 396 | 1189 | 478 | 594 |
| 0.080 | 0.25 | 1.42 | 0.013 | 0.003 | 0.029 | 0.001 | 0.005 | 0.043 | 0.001 | 0.047 | 0.040 | 0.013 | 0.0005 | 385 | 1200 | 478 | 591 |
| 0.080 | 0.25 | 1.43 | 0.013 | 0.003 | 0.024 | 0.001 | 0.010 | 0.039 | 0.001 | 0.047 | 0.040 | 0.014 | 0.0004 | 389 | 1193 | 477 | 590 |
| 0.081 | 0.25 | 1.43 | 0.013 | 0.003 | 0.024 | 0.001 | 0.003 | 0.043 | 0.001 | 0.049 | 0.040 | 0.013 | 0.0005 | 381 | 1210 | 480 | 592 |
| 0.080 | 0.25 | 1.42 | 0.013 | 0.003 | 0.029 | 0.001 | 0.011 | 0.043 | 0.001 | 0.048 | 0.041 | 0.013 | 0.0004 | 387 | 1198 | 478 | 590 |
| 0.080 | 0.25 | 1.42 | 0.013 | 0.003 | 0.028 | 0.001 | 0.014 | 0.037 | 0.001 | 0.047 | 0.040 | 0.014 | 0.0005 | 385 | 1192 | 478 | 590 |
| 0.080 | 0.25 | 1.41 | 0.013 | 0.003 | 0.029 | 0.001 | 0.006 | 0.039 | 0.001 | 0.047 | 0.039 | 0.013 | 0.0004 | 375 | 1189 | 478 | 591 |
| 0.080 | 0.26 | 1.44 | 0.013 | 0.003 | 0.031 | 0.001 | 0.006 | 0.040 | 0.001 | 0.045 | 0.040 | 0.013 | 0.0004 | 391 | 1188 | 477 | 593 |
| 0.079 | 0.25 | 1.43 | 0.013 | 0.003 | 0.034 | 0.001 | 0.012 | 0.045 | 0.001 | 0.047 | 0.040 | 0.013 | 0.0004 | 383 | 1209 | 478 | 589 |
| 0.080 | 0.26 | 1.42 | 0.014 | 0.003 | 0.029 | 0.001 | 0.005 | 0.041 | 0.002 | 0.045 | 0.041 | 0.013 | 0.0005 | 379 | 1212 | 477 | 588 |
| 0.080 | 0.25 | 1.44 | 0.013 | 0.003 | 0.031 | 0.001 | 0.006 | 0.040 | 0.001 | 0.048 | 0.040 | 0.013 | 0.0004 | 397 | 1190 | 478 | 593 |
| 0.080 | 0.25 | 1.42 | 0.013 | 0.003 | 0.022 | 0.001 | 0.009 | 0.040 | 0.001 | 0.047 | 0.040 | 0.013 | 0.0004 | 386 | 1183 | 478 | 594 |
| 0.079 | 0.25 | 1.42 | 0.013 | 0.003 | 0.030 | 0.001 | 0.011 | 0.042 | 0.001 | 0.048 | 0.040 | 0.013 | 0.0004 | 392 | 1201 | 478 | 590 |
| 0.079 | 0.25 | 1.42 | 0.013 | 0.003 | 0.029 | 0.001 | 0.010 | 0.040 | 0.001 | 0.048 | 0.040 | 0.013 | 0.0005 | 389 | 1202 | 477 | 590 |
| 0.079 | 0.25 | 1.41 | 0.013 | 0.003 | 0.030 | 0.001 | 0.006 | 0.042 | 0.001 | 0.046 | 0.040 | 0.014 | 0.0006 | 391 | 1200 | 477 | 589 |
| 0.079 | 0.25 | 1.42 | 0.013 | 0.003 | 0.027 | 0.001 | 0.016 | 0.039 | 0.001 | 0.048 | 0.040 | 0.013 | 0.0004 | 381 | 1204 | 478 | 590 |
| 0.078 | 0.26 | 1.43 | 0.014 | 0.003 | 0.031 | 0.001 | 0.011 | 0.035 | 0.001 | 0.048 | 0.040 | 0.013 | 0.0004 | 386 | 1204 | 478 | 589 |
| 0.081 | 0.25 | 1.41 | 0.013 | 0.003 | 0.026 | 0.001 | 0.008 | 0.044 | 0.001 | 0.047 | 0.040 | 0.013 | 0.0004 | 386 | 1192 | 478 | 592 |
| 0.080 | 0.25 | 1.44 | 0.014 | 0.003 | 0.029 | 0.001 | 0.007 | 0.043 | 0.001 | 0.045 | 0.041 | 0.013 | 0.0004 | 389 | 1210 | 477 | 590 |
| 0.081 | 0.25 | 1.42 | 0.013 | 0.003 | 0.026 | 0.001 | 0.008 | 0.040 | 0.001 | 0.045 | 0.039 | 0.013 | 0.0004 | 383 | 1200 | 478 | 591 |
| 0.079 | 0.25 | 1.42 | 0.013 | 0.003 | 0.027 | 0.001 | 0.009 | 0.045 | 0.002 | 0.047 | 0.041 | 0.013 | 0.0004 | 377 | 1202 | 478 | 590 |
| 0.080 | 0.25 | 1.44 | 0.013 | 0.003 | 0.031 | 0.001 | 0.011 | 0.040 | 0.001 | 0.048 | 0.040 | 0.013 | 0.0004 | 378 | 1192 | 477 | 592 |
| 0.080 | 0.25 | 1.42 | 0.013 | 0.004 | 0.029 | 0.001 | 0.009 | 0.043 | 0.001 | 0.047 | 0.040 | 0.013 | 0.0005 | 385 | 1201 | 479 | 592 |
| 0.079 | 0.25 | 1.42 | 0.013 | 0.003 | 0.030 | 0.001 | 0.014 | 0.039 | 0.001 | 0.048 | 0.039 | 0.013 | 0.0004 | 382 | 1212 | 478 | 589 |
| 0.079 | 0.25 | 1.43 | 0.013 | 0.003 | 0.029 | 0.001 | 0.013 | 0.040 | 0.001 | 0.048 | 0.039 | 0.013 | 0.0004 | 385 | 1201 | 478 | 591 |
| 0.080 | 0.25 | 1.43 | 0.013 | 0.003 | 0.030 | 0.001 | 0.007 | 0.040 | 0.001 | 0.047 | 0.040 | 0.013 | 0.0005 | 386 | 1201 | 478 | 592 |
| 0.079 | 0.25 | 1.42 | 0.013 | 0.003 | 0.031 | 0.001 | 0.007 | 0.043 | 0.001 | 0.048 | 0.040 | 0.013 | 0.0005 | 382 | 1197 | 478 | 591 |
| 0.081 | 0.25 | 1.43 | 0.013 | 0.003 | 0.025 | 0.001 | 0.012 | 0.041 | 0.001 | 0.047 | 0.040 | 0.013 | 0.0004 | 385 | 1190 | 478 | 592 |
| 0.080 | 0.25 | 1.43 | 0.013 | 0.003 | 0.023 | 0.001 | 0.005 | 0.043 | 0.001 | 0.045 | 0.040 | 0.013 | 0.0004 | 390 | 1179 | 477 | 594 |
| 0.079 | 0.25 | 1.42 | 0.013 | 0.003 | 0.029 | 0.001 | 0.010 | 0.039 | 0.002 | 0.046 | 0.041 | 0.013 | 0.0005 | 388 | 1211 | 477 | 587 |
| 0.081 | 0.25 | 1.42 | 0.013 | 0.003 | 0.026 | 0.001 | 0.010 | 0.044 | 0.002 | 0.046 | 0.040 | 0.013 | 0.0004 | 396 | 1183 | 476 | 592 |
| 0.080 | 0.25 | 1.43 | 0.013 | 0.003 | 0.029 | 0.001 | 0.008 | 0.040 | 0.001 | 0.048 | 0.039 | 0.013 | 0.0004 | 381 | 1190 | 479 | 592 |
| 0.080 | 0.25 | 1.44 | 0.013 | 0.003 | 0.031 | 0.001 | 0.027 | 0.041 | 0.001 | 0.048 | 0.039 | 0.013 | 0.0005 | 393 | 1183 | 475 | 592 |
| 0.080 | 0.25 | 1.42 | 0.013 | 0.003 | 0.024 | 0.001 | 0.006 | 0.040 | 0.001 | 0.046 | 0.040 | 0.013 | 0.0004 | 386 | 1192 | 478 | 593 |
| 0.080 | 0.25 | 1.43 | 0.013 | 0.003 | 0.031 | 0.001 | 0.010 | 0.041 | 0.001 | 0.046 | 0.040 | 0.013 | 0.0005 | 382 | 1185 | 478 | 592 |
| 0.079 | 0.25 | 1.42 | 0.013 | 0.003 | 0.029 | 0.001 | 0.014 | 0.041 | 0.001 | 0.045 | 0.041 | 0.013 | 0.0004 | 386 | 1200 | 477 | 589 |
| 0.080 | 0.26 | 1.43 | 0.013 | 0.003 | 0.025 | 0.001 | 0.016 | 0.040 | 0.001 | 0.045 | 0.040 | 0.013 | 0.0004 | 382 | 1204 | 478 | 590 |
| 0.081 | 0.25 | 1.44 | 0.013 | 0.003 | 0.034 | 0.001 | 0.020 | 0.040 | 0.001 | 0.047 | 0.041 | 0.013 | 0.0005 | 398 | 1180 | 476 | 593 |
| 0.080 | 0.25 | 1.43 | 0.013 | 0.004 | 0.028 | 0.001 | 0.017 | 0.042 | 0.002 | 0.047 | 0.039 | 0.013 | 0.0006 | 389 | 1194 | 477 | 591 |
| 0.081 | 0.25 | 1.42 | 0.013 | 0.003 | 0.029 | 0.001 | 0.003 | 0.046 | 0.002 | 0.047 | 0.040 | 0.013 | 0.0004 | 386 | 1194 | 477 | 590 |
| 0.080 | 0.25 | 1.43 | 0.013 | 0.003 | 0.030 | 0.001 | 0.019 | 0.041 | 0.001 | 0.049 | 0.040 | 0.013 | 0.0005 | 381 | 1186 | 478 | 592 |
| 0.080 | 0.25 | 1.43 | 0.013 | 0.003 | 0.029 | 0.001 | 0.005 | 0.038 | 0.001 | 0.048 | 0.040 | 0.013 | 0.0004 | 387 | 1199 | 479 | 593 |
| 0.080 | 0.25 | 1.42 | 0.013 | 0.003 | 0.029 | 0.001 | 0.006 | 0.041 | 0.001 | 0.046 | 0.039 | 0.013 | 0.0004 | 384 | 1195 | 478 | 592 |
| 0.079 | 0.25 | 1.43 | 0.013 | 0.003 | 0.028 | 0.001 | 0.015 | 0.043 | 0.001 | 0.046 | 0.039 | 0.013 | 0.0004 | 379 | 1190 | 478 | 591 |
| 0.080 | 0.25 | 1.43 | 0.013 | 0.003 | 0.028 | 0.001 | 0.016 | 0.045 | 0.001 | 0.046 | 0.040 | 0.013 | 0.0006 | 398 | 1198 | 477 | 590 |
| 0.079 | 0.25 | 1.42 | 0.013 | 0.003 | 0.035 | 0.001 | 0.007 | 0.042 | 0.002 | 0.048 | 0.039 | 0.013 | 0.0004 | 387 | 1209 | 478 | 590 |
| 0.080 | 0.25 | 1.42 | 0.013 | 0.003 | 0.032 | 0.001 | 0.020 | 0.044 | 0.002 | 0.046 | 0.041 | 0.014 | 0.0006 | 385 | 1215 | 476 | 586 |
| 0.079 | 0.25 | 1.43 | 0.013 | 0.003 | 0.033 | 0.001 | 0.007 | 0.042 | 0.001 | 0.046 | 0.040 | 0.013 | 0.0005 | 395 | 1191 | 477 | 591 |
| 0.080 | 0.25 | 1.43 | 0.013 | 0.003 | 0.024 | 0.001 | 0.005 | 0.041 | 0.001 | 0.047 | 0.040 | 0.013 | 0.0005 | 387 | 1188 | 479 | 593 |
| 0.081 | 0.25 | 1.43 | 0.013 | 0.003 | 0.025 | 0.001 | 0.006 | 0.043 | 0.001 | 0.047 | 0.041 | 0.013 | 0.0004 | 383 | 1201 | 478 | 590 |
| 0.079 | 0.25 | 1.43 | 0.013 | 0.003 | 0.030 | 0.001 | 0.010 | 0.041 | 0.001 | 0.047 | 0.040 | 0.014 | 0.0005 | 379 | 1216 | 478 | 587 |
| 0.078 | 0.25 | 1.42 | 0.013 | 0.003 | 0.031 | 0.001 | 0.027 | 0.042 | 0.002 | 0.047 | 0.042 | 0.013 | 0.0005 | 386 | 1196 | 476 | 586 |
| 0.080 | 0.25 | 1.44 | 0.013 | 0.003 | 0.027 | 0.001 | 0.015 | 0.044 | 0.001 | 0.048 | 0.040 | 0.013 | 0.0004 | 388 | 1186 | 478 | 593 |
| 0.079 | 0.25 | 1.42 | 0.013 | 0.003 | 0.029 | 0.001 | 0.013 | 0.040 | 0.001 | 0.047 | 0.039 | 0.013 | 0.0004 | 383 | 1193 | 479 | 592 |
| 0.080 | 0.25 | 1.43 | 0.013 | 0.003 | 0.026 | 0.001 | 0.015 | 0.040 | 0.001 | 0.048 | 0.040 | 0.013 | 0.0005 | 380 | 1205 | 478 | 591 |
| 0.080 | 0.25 | 1.43 | 0.013 | 0.003 | 0.031 | 0.001 | 0.004 | 0.043 | 0.001 | 0.047 | 0.039 | 0.013 | 0.0005 | 383 | 1190 | 479 | 593 |
| 0.080 | 0.25 | 1.40 | 0.013 | 0.004 | 0.029 | 0.001 | 0.012 | 0.041 | 0.001 | 0.046 | 0.040 | 0.013 | 0.0005 | 384 | 1180 | 478 | 591 |
| 0.079 | 0.25 | 1.43 | 0.013 | 0.004 | 0.027 | 0.001 | 0.007 | 0.042 | 0.001 | 0.046 | 0.039 | 0.013 | 0.0005 | 385 | 1197 | 478 | 590 |
| 0.080 | 0.24 | 1.41 | 0.013 | 0.003 | 0.031 | 0.001 | 0.012 | 0.045 | 0.002 | 0.046 | 0.041 | 0.014 | 0.0005 | 380 | 1201 | 477 | 586 |
| 0.081 | 0.25 | 1.43 | 0.013 | 0.003 | 0.025 | 0.001 | 0.004 | 0.038 | 0.001 | 0.049 | 0.040 | 0.013 | 0.0004 | 386 | 1192 | 480 | 595 |
| 0.080 | 0.25 | 1.44 | 0.013 | 0.004 | 0.030 | 0.001 | 0.014 | 0.041 | 0.001 | 0.046 | 0.039 | 0.013 | 0.0004 | 395 | 1193 | 478 | 593 |
| 0.080 | 0.25 | 1.42 | 0.013 | 0.003 | 0.028 | 0.001 | 0.006 | 0.044 | 0.001 | 0.047 | 0.040 | 0.013 | 0.0005 | 388 | 1203 | 478 | 590 |
| 0.080 | 0.25 | 1.43 | 0.013 | 0.003 | 0.031 | 0.001 | 0.008 | 0.041 | 0.001 | 0.046 | 0.040 | 0.013 | 0.0004 | 384 | 1206 | 479 | 591 |
| 0.078 | 0.26 | 1.41 | 0.013 | 0.003 | 0.027 | 0.001 | 0.006 | 0.038 | 0.001 | 0.048 | 0.039 | 0.013 | 0.0004 | 391 | 1172 | 477 | 593 |
| 0.080 | 0.25 | 1.42 | 0.013 | 0.003 | 0.026 | 0.001 | 0.006 | 0.040 | 0.002 | 0.045 | 0.040 | 0.013 | 0.0005 | 387 | 1195 | 477 | 590 |
| 0.080 | 0.25 | 1.44 | 0.013 | 0.003 | 0.024 | 0.001 | 0.011 | 0.041 | 0.001 | 0.047 | 0.040 | 0.013 | 0.0005 | 380 | 1191 | 478 | 592 |
| 0.081 | 0.25 | 1.43 | 0.013 | 0.003 | 0.027 | 0.001 | 0.013 | 0.042 | 0.001 | 0.046 | 0.039 | 0.013 | 0.0005 | 391 | 1188 | 477 | 593 |
| 0.080 | 0.25 | 1.42 | 0.013 | 0.003 | 0.028 | 0.001 | 0.007 | 0.044 | 0.001 | 0.048 | 0.040 | 0.013 | 0.0005 | 387 | 1194 | 478 | 591 |
| 0.081 | 0.25 | 1.42 | 0.013 | 0.003 | 0.024 | 0.001 | 0.005 | 0.044 | 0.001 | 0.048 | 0.041 | 0.013 | 0.0005 | 385 | 1190 | 478 | 592 |
| 0.079 | 0.25 | 1.43 | 0.013 | 0.003 | 0.030 | 0.001 | 0.005 | 0.041 | 0.001 | 0.047 | 0.040 | 0.014 | 0.0005 | 380 | 1194 | 478 | 590 |
| 0.081 | 0.26 | 1.42 | 0.013 | 0.003 | 0.028 | 0.001 | 0.006 | 0.042 | 0.002 | 0.047 | 0.039 | 0.013 | 0.0004 | 381 | 1188 | 477 | 592 |
| 0.080 | 0.25 | 1.42 | 0.013 | 0.003 | 0.027 | 0.001 | 0.014 | 0.040 | 0.001 | 0.048 | 0.039 | 0.013 | 0.0004 | 393 | 1182 | 478 | 593 |
| 0.080 | 0.25 | 1.42 | 0.013 | 0.003 | 0.029 | 0.001 | 0.004 | 0.039 | 0.001 | 0.049 | 0.038 | 0.013 | 0.0003 | 380 | 1194 | 479 | 592 |
| 0.080 | 0.25 | 1.43 | 0.013 | 0.003 | 0.025 | 0.001 | 0.013 | 0.042 | 0.001 | 0.048 | 0.040 | 0.013 | 0.0004 | 393 | 1183 | 478 | 594 |
| 0.080 | 0.25 | 1.44 | 0.013 | 0.003 | 0.030 | 0.001 | 0.012 | 0.038 | 0.001 | 0.048 | 0.039 | 0.013 | 0.0004 | 387 | 1203 | 478 | 592 |
| 0.079 | 0.25 | 1.42 | 0.013 | 0.003 | 0.030 | 0.001 | 0.006 | 0.038 | 0.002 | 0.047 | 0.040 | 0.013 | 0.0005 | 386 | 1201 | 478 | 588 |
| 0.079 | 0.25 | 1.41 | 0.013 | 0.003 | 0.029 | 0.001 | 0.019 | 0.043 | 0.002 | 0.047 | 0.040 | 0.014 | 0.0004 | 384 | 1211 | 477 | 586 |
| 0.080 | 0.25 | 1.43 | 0.013 | 0.003 | 0.029 | 0.001 | 0.016 | 0.041 | 0.001 | 0.046 | 0.041 | 0.013 | 0.0005 | 398 | 1214 | 477 | 589 |
| 0.080 | 0.25 | 1.43 | 0.013 | 0.003 | 0.028 | 0.001 | 0.007 | 0.043 | 0.001 | 0.048 | 0.040 | 0.013 | 0.0004 | 390 | 1184 | 477 | 594 |
| 0.080 | 0.25 | 1.44 | 0.013 | 0.004 | 0.027 | 0.001 | 0.005 | 0.039 | 0.001 | 0.046 | 0.040 | 0.013 | 0.0004 | 404 | 1191 | 478 | 594 |
| 0.080 | 0.25 | 1.43 | 0.013 | 0.003 | 0.028 | 0.001 | 0.004 | 0.040 | 0.001 | 0.048 | 0.039 | 0.013 | 0.0005 | 388 | 1191 | 478 | 592 |
| 0.080 | 0.26 | 1.42 | 0.013 | 0.003 | 0.032 | 0.001 | 0.006 | 0.040 | 0.002 | 0.047 | 0.039 | 0.013 | 0.0004 | 383 | 1180 | 477 | 592 |
| 0.080 | 0.25 | 1.43 | 0.013 | 0.003 | 0.030 | 0.001 | 0.011 | 0.048 | 0.002 | 0.048 | 0.040 | 0.013 | 0.0004 | 381 | 1188 | 478 | 591 |
| 0.081 | 0.25 | 1.42 | 0.013 | 0.003 | 0.027 | 0.001 | 0.006 | 0.047 | 0.001 | 0.046 | 0.040 | 0.013 | 0.0005 | 384 | 1185 | 477 | 591 |
| 0.081 | 0.25 | 1.44 | 0.013 | 0.003 | 0.026 | 0.001 | 0.015 | 0.041 | 0.001 | 0.049 | 0.040 | 0.013 | 0.0005 | 389 | 1196 | 478 | 594 |
| 0.080 | 0.25 | 1.42 | 0.013 | 0.003 | 0.031 | 0.001 | 0.008 | 0.041 | 0.001 | 0.046 | 0.040 | 0.013 | 0.0004 | 389 | 1214 | 478 | 590 |
| 0.080 | 0.26 | 1.43 | 0.013 | 0.003 | 0.028 | 0.001 | 0.019 | 0.038 | 0.001 | 0.046 | 0.040 | 0.013 | 0.0004 | 392 | 1201 | 477 | 592 |
| 0.080 | 0.25 | 1.42 | 0.013 | 0.003 | 0.029 | 0.001 | 0.005 | 0.044 | 0.001 | 0.048 | 0.039 | 0.013 | 0.0004 | 393 | 1193 | 477 | 592 |
| 0.080 | 0.25 | 1.43 | 0.013 | 0.004 | 0.027 | 0.001 | 0.011 | 0.036 | 0.001 | 0.047 | 0.040 | 0.013 | 0.0005 | 389 | 1176 | 478 | 593 |
| 0.081 | 0.25 | 1.43 | 0.013 | 0.003 | 0.029 | 0.001 | 0.010 | 0.040 | 0.001 | 0.044 | 0.040 | 0.013 | 0.0004 | 394 | 1201 | 477 | 591 |
| 0.080 | 0.25 | 1.43 | 0.013 | 0.003 | 0.027 | 0.001 | 0.009 | 0.043 | 0.001 | 0.044 | 0.040 | 0.014 | 0.0004 | 382 | 1201 | 477 | 588 |
| 0.080 | 0.25 | 1.44 | 0.013 | 0.003 | 0.027 | 0.001 | 0.018 | 0.042 | 0.001 | 0.044 | 0.040 | 0.013 | 0.0004 | 385 | 1194 | 477 | 590 |
| 0.080 | 0.25 | 1.42 | 0.013 | 0.003 | 0.029 | 0.001 | 0.010 | 0.043 | 0.001 | 0.049 | 0.040 | 0.013 | 0.0005 | 384 | 1200 | 478 | 592 |
| 0.080 | 0.25 | 1.42 | 0.013 | 0.003 | 0.026 | 0.001 | 0.008 | 0.043 | 0.002 | 0.047 | 0.040 | 0.013 | 0.0005 | 386 | 1204 | 478 | 591 |
| 0.080 | 0.24 | 1.43 | 0.013 | 0.003 | 0.026 | 0.001 | 0.003 | 0.040 | 0.001 | 0.048 | 0.041 | 0.013 | 0.0004 | 376 | 1197 | 479 | 590 |
| 0.080 | 0.25 | 1.44 | 0.013 | 0.003 | 0.028 | 0.001 | 0.011 | 0.040 | 0.001 | 0.048 | 0.039 | 0.013 | 0.0004 | 376 | 1206 | 479 | 590 |
| 0.079 | 0.25 | 1.43 | 0.013 | 0.003 | 0.026 | 0.001 | 0.019 | 0.043 | 0.001 | 0.048 | 0.039 | 0.013 | 0.0006 | 379 | 1194 | 478 | 592 |
| 0.079 | 0.25 | 1.44 | 0.013 | 0.004 | 0.025 | 0.001 | 0.015 | 0.042 | 0.001 | 0.046 | 0.039 | 0.013 | 0.0005 | 394 | 1186 | 478 | 593 |
| 0.080 | 0.25 | 1.43 | 0.013 | 0.003 | 0.029 | 0.001 | 0.008 | 0.045 | 0.002 | 0.047 | 0.040 | 0.013 | 0.0005 | 379 | 1203 | 478 | 590 |
|  |  |  |  |  |  |  |  |  |  |  |  |  |  |  |  |  |  |
| **KNN** | | | | | | | | | | | | | | | | | |
| C | Si | Mn | P | S | Cu | Sn | Ni | Cr | Mo | V | Nb | Ti | Ca | Time | Temp | YS | UTS |
| (wt%) | (wt%) | (wt%) | (wt%) | (wt%) | (wt%) | (wt%) | (wt%) | (wt%) | (wt%) | (wt%) | (wt%) | (wt%) | (wt%) | (s) | (℃) | (Mpa) | (Mpa) |
| 0.060 | 0.23 | 1.45 | 0.014 | 0.003 | 0.090 | 0.001 | 0.062 | 0.025 | 0.004 | 0.035 | 0.042 | 0.012 | 0.0021 | 346 | 1052 | 476 | 582 |
| 0.082 | 0.26 | 1.49 | 0.012 | 0.003 | 0.042 | 0.001 | 0.025 | 0.037 | 0.001 | 0.048 | 0.037 | 0.013 | 0.0004 | 471 | 1128 | 483 | 591 |
| 0.077 | 0.26 | 1.42 | 0.010 | 0.004 | 0.036 | 0.001 | 0.010 | 0.036 | 0.000 | 0.048 | 0.037 | 0.013 | 0.0004 | 472 | 1181 | 486 | 596 |
| 0.070 | 0.26 | 1.20 | 0.008 | 0.001 | 0.122 | 0.002 | 0.143 | 0.080 | 0.008 | 0.024 | 0.032 | 0.016 | 0.0019 | 403 | 1213 | 484 | 561 |
| 0.072 | 0.24 | 1.44 | 0.013 | 0.003 | 0.086 | 0.001 | 0.050 | 0.082 | 0.006 | 0.038 | 0.017 | 0.014 | 0.0026 | 329 | 1141 | 478 | 586 |
| 0.082 | 0.26 | 1.44 | 0.008 | 0.004 | 0.036 | 0.001 | 0.018 | 0.045 | 0.001 | 0.048 | 0.032 | 0.011 | 0.0006 | 478 | 1174 | 490 | 594 |
| 0.079 | 0.22 | 1.50 | 0.009 | 0.003 | 0.045 | 0.002 | 0.011 | 0.125 | 0.005 | 0.003 | 0.041 | 0.011 | 0.0025 | 383 | 1157 | 484 | 589 |
| 0.069 | 0.22 | 1.31 | 0.006 | 0.002 | 0.076 | 0.002 | 0.070 | 0.105 | 0.008 | 0.042 | 0.029 | 0.016 | 0.0020 | 471 | 1238 | 493 | 568 |
| 0.079 | 0.24 | 1.42 | 0.012 | 0.003 | 0.060 | 0.001 | 0.095 | 0.078 | 0.007 | 0.033 | 0.024 | 0.015 | 0.0005 | 488 | 1157 | 477 | 590 |
| 0.080 | 0.29 | 1.46 | 0.013 | 0.003 | 0.057 | 0.001 | 0.037 | 0.043 | 0.007 | 0.031 | 0.034 | 0.012 | 0.0009 | 472 | 1135 | 477 | 590 |
| 0.063 | 0.23 | 1.23 | 0.007 | 0.002 | 0.145 | 0.001 | 0.117 | 0.065 | 0.009 | 0.031 | 0.022 | 0.017 | 0.0018 | 358 | 1183 | 497 | 566 |
| 0.061 | 0.23 | 1.23 | 0.007 | 0.002 | 0.123 | 0.001 | 0.156 | 0.112 | 0.008 | 0.027 | 0.043 | 0.016 | 0.0021 | 353 | 1185 | 498 | 564 |
| 0.071 | 0.27 | 1.26 | 0.015 | 0.003 | 0.047 | 0.002 | 0.065 | 0.054 | 0.009 | 0.038 | 0.027 | 0.012 | 0.0012 | 381 | 1148 | 464 | 581 |
| 0.079 | 0.22 | 1.22 | 0.007 | 0.002 | 0.082 | 0.001 | 0.076 | 0.061 | 0.007 | 0.035 | 0.024 | 0.016 | 0.0023 | 279 | 1241 | 500 | 563 |
| 0.073 | 0.28 | 1.45 | 0.007 | 0.003 | 0.035 | 0.001 | 0.023 | 0.051 | 0.001 | 0.042 | 0.040 | 0.011 | 0.0011 | 460 | 1180 | 491 | 593 |
| 0.077 | 0.24 | 1.46 | 0.012 | 0.003 | 0.065 | 0.001 | 0.054 | 0.042 | 0.000 | 0.044 | 0.036 | 0.011 | 0.0004 | 468 | 1060 | 481 | 591 |
| 0.078 | 0.24 | 1.41 | 0.012 | 0.004 | 0.031 | 0.001 | 0.018 | 0.031 | 0.000 | 0.048 | 0.037 | 0.011 | 0.0003 | 467 | 1122 | 483 | 592 |
| 0.080 | 0.27 | 1.46 | 0.013 | 0.003 | 0.049 | 0.001 | 0.023 | 0.034 | 0.002 | 0.041 | 0.033 | 0.011 | 0.0007 | 441 | 1188 | 484 | 590 |
| 0.054 | 0.22 | 1.19 | 0.008 | 0.002 | 0.125 | 0.001 | 0.156 | 0.115 | 0.009 | 0.020 | 0.043 | 0.016 | 0.0021 | 262 | 1184 | 500 | 563 |
| 0.069 | 0.23 | 1.18 | 0.007 | 0.003 | 0.109 | 0.002 | 0.138 | 0.087 | 0.009 | 0.020 | 0.035 | 0.016 | 0.0019 | 354 | 1222 | 497 | 567 |
| 0.076 | 0.27 | 1.44 | 0.013 | 0.003 | 0.027 | 0.001 | 0.015 | 0.031 | 0.000 | 0.046 | 0.036 | 0.012 | 0.0003 | 472 | 1213 | 477 | 593 |
| 0.081 | 0.27 | 1.44 | 0.009 | 0.004 | 0.016 | 0.001 | 0.012 | 0.035 | 0.001 | 0.035 | 0.037 | 0.013 | 0.0004 | 453 | 1183 | 491 | 594 |
| 0.078 | 0.27 | 1.44 | 0.013 | 0.003 | 0.034 | 0.001 | 0.028 | 0.039 | 0.002 | 0.046 | 0.038 | 0.011 | 0.0006 | 459 | 1176 | 484 | 591 |
| 0.083 | 0.25 | 1.38 | 0.015 | 0.004 | 0.022 | 0.001 | 0.126 | 0.043 | 0.005 | 0.020 | 0.043 | 0.012 | 0.0019 | 382 | 1156 | 484 | 593 |
| 0.080 | 0.25 | 1.48 | 0.013 | 0.003 | 0.041 | 0.001 | 0.044 | 0.038 | 0.001 | 0.041 | 0.031 | 0.011 | 0.0005 | 434 | 1236 | 481 | 591 |
| 0.060 | 0.23 | 1.30 | 0.006 | 0.002 | 0.141 | 0.001 | 0.124 | 0.109 | 0.009 | 0.010 | 0.042 | 0.016 | 0.0014 | 283 | 1187 | 500 | 563 |
| 0.073 | 0.23 | 1.22 | 0.005 | 0.002 | 0.160 | 0.002 | 0.024 | 0.093 | 0.009 | 0.035 | 0.022 | 0.016 | 0.0021 | 348 | 1183 | 492 | 566 |
| 0.071 | 0.27 | 1.47 | 0.013 | 0.004 | 0.044 | 0.002 | 0.057 | 0.118 | 0.007 | 0.027 | 0.031 | 0.013 | 0.0006 | 341 | 1003 | 458 | 568 |
| 0.077 | 0.26 | 1.47 | 0.013 | 0.003 | 0.033 | 0.001 | 0.016 | 0.038 | 0.001 | 0.042 | 0.038 | 0.012 | 0.0003 | 473 | 1208 | 477 | 593 |
| 0.082 | 0.26 | 1.38 | 0.011 | 0.003 | 0.049 | 0.001 | 0.023 | 0.046 | 0.008 | 0.032 | 0.040 | 0.011 | 0.0007 | 473 | 1133 | 479 | 585 |
| 0.061 | 0.23 | 1.24 | 0.008 | 0.001 | 0.113 | 0.001 | 0.054 | 0.070 | 0.008 | 0.019 | 0.026 | 0.016 | 0.0010 | 315 | 1179 | 492 | 565 |
| 0.082 | 0.25 | 1.45 | 0.013 | 0.003 | 0.042 | 0.001 | 0.018 | 0.031 | 0.000 | 0.038 | 0.038 | 0.012 | 0.0003 | 467 | 1112 | 483 | 591 |
| 0.067 | 0.23 | 1.24 | 0.008 | 0.002 | 0.111 | 0.001 | 0.145 | 0.114 | 0.009 | 0.022 | 0.039 | 0.016 | 0.0019 | 356 | 1187 | 490 | 567 |
| 0.075 | 0.22 | 1.51 | 0.013 | 0.003 | 0.074 | 0.002 | 0.079 | 0.050 | 0.006 | 0.011 | 0.031 | 0.011 | 0.0023 | 318 | 1145 | 480 | 586 |
| 0.055 | 0.22 | 1.25 | 0.012 | 0.001 | 0.061 | 0.002 | 0.042 | 0.061 | 0.004 | 0.042 | 0.040 | 0.011 | 0.0025 | 443 | 1185 | 487 | 568 |
| 0.082 | 0.27 | 1.42 | 0.009 | 0.004 | 0.048 | 0.001 | 0.021 | 0.040 | 0.001 | 0.045 | 0.036 | 0.013 | 0.0006 | 454 | 1131 | 490 | 594 |
| 0.060 | 0.22 | 1.28 | 0.009 | 0.001 | 0.120 | 0.001 | 0.141 | 0.124 | 0.007 | 0.023 | 0.042 | 0.016 | 0.0021 | 331 | 1184 | 493 | 564 |
| 0.075 | 0.27 | 1.35 | 0.014 | 0.002 | 0.061 | 0.002 | 0.058 | 0.076 | 0.007 | 0.030 | 0.035 | 0.015 | 0.0004 | 447 | 1182 | 469 | 575 |
| 0.078 | 0.26 | 1.47 | 0.011 | 0.003 | 0.037 | 0.001 | 0.024 | 0.040 | 0.000 | 0.048 | 0.037 | 0.011 | 0.0006 | 470 | 1112 | 483 | 591 |
| 0.079 | 0.25 | 1.45 | 0.012 | 0.003 | 0.044 | 0.001 | 0.012 | 0.035 | 0.001 | 0.043 | 0.036 | 0.011 | 0.0004 | 463 | 1255 | 481 | 592 |
| 0.079 | 0.26 | 1.46 | 0.012 | 0.003 | 0.012 | 0.001 | 0.012 | 0.032 | 0.000 | 0.044 | 0.038 | 0.012 | 0.0003 | 468 | 1104 | 483 | 592 |
| 0.063 | 0.27 | 1.26 | 0.008 | 0.001 | 0.118 | 0.002 | 0.121 | 0.120 | 0.007 | 0.024 | 0.037 | 0.015 | 0.0018 | 414 | 1268 | 486 | 562 |
| 0.077 | 0.24 | 1.43 | 0.012 | 0.003 | 0.040 | 0.001 | 0.057 | 0.094 | 0.004 | 0.031 | 0.038 | 0.011 | 0.0015 | 459 | 1099 | 484 | 590 |
| 0.081 | 0.25 | 1.46 | 0.012 | 0.003 | 0.048 | 0.001 | 0.021 | 0.055 | 0.002 | 0.045 | 0.037 | 0.012 | 0.0005 | 452 | 1128 | 484 | 591 |
| 0.079 | 0.26 | 1.30 | 0.015 | 0.001 | 0.044 | 0.002 | 0.083 | 0.037 | 0.001 | 0.045 | 0.038 | 0.013 | 0.0008 | 381 | 1185 | 465 | 575 |
| 0.072 | 0.24 | 1.20 | 0.008 | 0.002 | 0.142 | 0.002 | 0.017 | 0.050 | 0.006 | 0.030 | 0.029 | 0.013 | 0.0006 | 457 | 1146 | 482 | 560 |
| 0.072 | 0.29 | 1.27 | 0.015 | 0.002 | 0.075 | 0.002 | 0.031 | 0.047 | 0.004 | 0.026 | 0.038 | 0.013 | 0.0011 | 368 | 1185 | 466 | 573 |
| 0.081 | 0.25 | 1.47 | 0.013 | 0.003 | 0.046 | 0.001 | 0.033 | 0.032 | 0.001 | 0.040 | 0.037 | 0.012 | 0.0007 | 474 | 1199 | 486 | 592 |
| 0.078 | 0.25 | 1.49 | 0.013 | 0.003 | 0.041 | 0.001 | 0.021 | 0.034 | 0.000 | 0.042 | 0.038 | 0.011 | 0.0006 | 439 | 1176 | 484 | 590 |
| 0.080 | 0.23 | 1.50 | 0.011 | 0.003 | 0.032 | 0.001 | 0.023 | 0.035 | 0.001 | 0.042 | 0.038 | 0.011 | 0.0004 | 459 | 1200 | 484 | 591 |
| 0.068 | 0.23 | 1.27 | 0.006 | 0.002 | 0.142 | 0.001 | 0.084 | 0.086 | 0.009 | 0.016 | 0.039 | 0.016 | 0.0019 | 368 | 1189 | 490 | 567 |
| 0.082 | 0.27 | 1.32 | 0.013 | 0.002 | 0.047 | 0.001 | 0.043 | 0.052 | 0.003 | 0.037 | 0.036 | 0.011 | 0.0013 | 462 | 988 | 465 | 579 |
| 0.078 | 0.26 | 1.43 | 0.013 | 0.003 | 0.062 | 0.001 | 0.021 | 0.033 | 0.000 | 0.043 | 0.037 | 0.013 | 0.0003 | 469 | 1217 | 481 | 593 |
| 0.061 | 0.23 | 1.24 | 0.006 | 0.002 | 0.134 | 0.001 | 0.146 | 0.117 | 0.009 | 0.023 | 0.043 | 0.016 | 0.0020 | 353 | 1180 | 498 | 565 |
| 0.071 | 0.29 | 1.17 | 0.006 | 0.001 | 0.153 | 0.002 | 0.015 | 0.048 | 0.008 | 0.038 | 0.030 | 0.016 | 0.0015 | 382 | 1241 | 483 | 563 |
| 0.072 | 0.23 | 1.19 | 0.007 | 0.002 | 0.131 | 0.002 | 0.144 | 0.110 | 0.009 | 0.024 | 0.041 | 0.016 | 0.0016 | 364 | 1184 | 498 | 564 |
| 0.067 | 0.23 | 1.22 | 0.009 | 0.002 | 0.088 | 0.001 | 0.150 | 0.092 | 0.008 | 0.024 | 0.034 | 0.015 | 0.0022 | 327 | 1192 | 500 | 564 |
| 0.060 | 0.31 | 1.17 | 0.010 | 0.002 | 0.161 | 0.001 | 0.050 | 0.061 | 0.004 | 0.038 | 0.035 | 0.011 | 0.0004 | 412 | 1198 | 486 | 567 |
| 0.076 | 0.24 | 1.34 | 0.014 | 0.001 | 0.021 | 0.002 | 0.017 | 0.035 | 0.001 | 0.041 | 0.037 | 0.013 | 0.0004 | 361 | 1168 | 465 | 576 |
| 0.059 | 0.22 | 1.43 | 0.013 | 0.003 | 0.094 | 0.001 | 0.019 | 0.081 | 0.005 | 0.038 | 0.019 | 0.016 | 0.0002 | 374 | 1146 | 480 | 586 |
| 0.082 | 0.26 | 1.44 | 0.013 | 0.003 | 0.020 | 0.001 | 0.008 | 0.030 | 0.004 | 0.035 | 0.039 | 0.013 | 0.0013 | 473 | 1178 | 484 | 590 |
| 0.081 | 0.26 | 1.52 | 0.013 | 0.003 | 0.098 | 0.001 | 0.056 | 0.053 | 0.007 | 0.033 | 0.035 | 0.014 | 0.0025 | 324 | 1144 | 487 | 588 |
| 0.081 | 0.25 | 1.49 | 0.012 | 0.003 | 0.045 | 0.001 | 0.023 | 0.047 | 0.001 | 0.047 | 0.036 | 0.013 | 0.0006 | 449 | 1179 | 484 | 591 |
| 0.082 | 0.24 | 1.48 | 0.013 | 0.004 | 0.041 | 0.001 | 0.005 | 0.061 | 0.001 | 0.039 | 0.033 | 0.011 | 0.0006 | 465 | 984 | 482 | 594 |
| 0.063 | 0.23 | 1.19 | 0.007 | 0.002 | 0.110 | 0.001 | 0.103 | 0.104 | 0.008 | 0.028 | 0.040 | 0.016 | 0.0016 | 355 | 1182 | 497 | 566 |
| 0.081 | 0.24 | 1.47 | 0.007 | 0.003 | 0.025 | 0.001 | 0.049 | 0.097 | 0.007 | 0.028 | 0.043 | 0.016 | 0.0015 | 398 | 1137 | 482 | 591 |
| 0.082 | 0.25 | 1.47 | 0.014 | 0.003 | 0.094 | 0.002 | 0.065 | 0.035 | 0.003 | 0.045 | 0.035 | 0.011 | 0.0009 | 433 | 1220 | 487 | 592 |
| 0.079 | 0.27 | 1.49 | 0.010 | 0.004 | 0.084 | 0.001 | 0.010 | 0.023 | 0.002 | 0.043 | 0.024 | 0.011 | 0.0006 | 462 | 1132 | 488 | 594 |
| 0.060 | 0.23 | 1.23 | 0.007 | 0.002 | 0.116 | 0.001 | 0.145 | 0.118 | 0.009 | 0.015 | 0.035 | 0.016 | 0.0017 | 367 | 1218 | 493 | 565 |
| 0.077 | 0.27 | 1.45 | 0.009 | 0.003 | 0.036 | 0.002 | 0.029 | 0.047 | 0.000 | 0.034 | 0.037 | 0.013 | 0.0007 | 335 | 1166 | 489 | 585 |
| 0.079 | 0.26 | 1.49 | 0.013 | 0.003 | 0.014 | 0.001 | 0.017 | 0.034 | 0.001 | 0.045 | 0.036 | 0.012 | 0.0005 | 449 | 1187 | 484 | 591 |
| 0.062 | 0.25 | 1.22 | 0.008 | 0.002 | 0.119 | 0.001 | 0.148 | 0.127 | 0.009 | 0.017 | 0.041 | 0.016 | 0.0022 | 315 | 1180 | 492 | 565 |
| 0.067 | 0.28 | 1.26 | 0.009 | 0.002 | 0.032 | 0.002 | 0.043 | 0.034 | 0.006 | 0.041 | 0.031 | 0.013 | 0.0008 | 453 | 1178 | 479 | 566 |
| 0.061 | 0.23 | 1.18 | 0.009 | 0.002 | 0.140 | 0.001 | 0.143 | 0.116 | 0.009 | 0.019 | 0.044 | 0.016 | 0.0020 | 353 | 1182 | 498 | 564 |
| 0.075 | 0.26 | 1.32 | 0.014 | 0.003 | 0.024 | 0.002 | 0.016 | 0.036 | 0.001 | 0.041 | 0.037 | 0.012 | 0.0003 | 335 | 1092 | 455 | 572 |
| 0.083 | 0.26 | 1.42 | 0.014 | 0.003 | 0.028 | 0.001 | 0.015 | 0.034 | 0.001 | 0.038 | 0.039 | 0.013 | 0.0005 | 454 | 1150 | 475 | 591 |
| 0.056 | 0.22 | 1.44 | 0.013 | 0.003 | 0.077 | 0.001 | 0.025 | 0.087 | 0.005 | 0.002 | 0.038 | 0.012 | 0.0007 | 360 | 1159 | 480 | 586 |
| 0.069 | 0.25 | 1.28 | 0.010 | 0.002 | 0.054 | 0.002 | 0.182 | 0.078 | 0.005 | 0.038 | 0.040 | 0.012 | 0.0009 | 447 | 1197 | 495 | 563 |
| 0.079 | 0.25 | 1.47 | 0.013 | 0.003 | 0.028 | 0.001 | 0.008 | 0.040 | 0.001 | 0.047 | 0.040 | 0.011 | 0.0004 | 453 | 1174 | 481 | 591 |
| 0.077 | 0.27 | 1.43 | 0.007 | 0.004 | 0.068 | 0.001 | 0.037 | 0.050 | 0.003 | 0.044 | 0.036 | 0.012 | 0.0012 | 462 | 1181 | 487 | 596 |
| 0.072 | 0.31 | 1.41 | 0.013 | 0.003 | 0.166 | 0.002 | 0.058 | 0.065 | 0.007 | 0.029 | 0.027 | 0.011 | 0.0010 | 344 | 1073 | 462 | 575 |
| 0.070 | 0.27 | 1.14 | 0.010 | 0.002 | 0.050 | 0.001 | 0.195 | 0.075 | 0.005 | 0.032 | 0.035 | 0.011 | 0.0007 | 421 | 1188 | 487 | 568 |
| 0.082 | 0.27 | 1.51 | 0.013 | 0.003 | 0.025 | 0.001 | 0.014 | 0.042 | 0.000 | 0.045 | 0.028 | 0.011 | 0.0003 | 449 | 1177 | 484 | 591 |
| 0.073 | 0.23 | 1.49 | 0.014 | 0.003 | 0.018 | 0.001 | 0.024 | 0.048 | 0.001 | 0.035 | 0.037 | 0.013 | 0.0003 | 491 | 1230 | 481 | 589 |
| 0.080 | 0.25 | 1.43 | 0.013 | 0.003 | 0.057 | 0.001 | 0.042 | 0.037 | 0.001 | 0.043 | 0.037 | 0.012 | 0.0006 | 445 | 1248 | 481 | 592 |
| 0.079 | 0.27 | 1.49 | 0.009 | 0.003 | 0.036 | 0.001 | 0.021 | 0.033 | 0.002 | 0.040 | 0.028 | 0.012 | 0.0005 | 472 | 1178 | 487 | 596 |
| 0.079 | 0.25 | 1.49 | 0.011 | 0.003 | 0.034 | 0.001 | 0.025 | 0.031 | 0.000 | 0.047 | 0.038 | 0.011 | 0.0004 | 462 | 1181 | 484 | 592 |
| 0.082 | 0.25 | 1.48 | 0.012 | 0.004 | 0.056 | 0.001 | 0.024 | 0.071 | 0.002 | 0.038 | 0.033 | 0.011 | 0.0012 | 474 | 1121 | 484 | 590 |
| 0.078 | 0.27 | 1.48 | 0.013 | 0.003 | 0.051 | 0.001 | 0.048 | 0.057 | 0.002 | 0.032 | 0.040 | 0.011 | 0.0007 | 474 | 1186 | 481 | 591 |
| 0.078 | 0.26 | 1.48 | 0.013 | 0.003 | 0.051 | 0.001 | 0.048 | 0.057 | 0.003 | 0.032 | 0.040 | 0.011 | 0.0008 | 474 | 1186 | 481 | 591 |
| 0.079 | 0.26 | 1.45 | 0.013 | 0.003 | 0.047 | 0.001 | 0.017 | 0.031 | 0.001 | 0.046 | 0.037 | 0.012 | 0.0003 | 472 | 1216 | 481 | 593 |
| 0.077 | 0.32 | 1.59 | 0.005 | 0.003 | 0.118 | 0.001 | 0.088 | 0.061 | 0.006 | 0.034 | 0.038 | 0.011 | 0.0027 | 385 | 1161 | 484 | 589 |
| 0.059 | 0.22 | 1.18 | 0.007 | 0.001 | 0.077 | 0.001 | 0.136 | 0.080 | 0.008 | 0.014 | 0.036 | 0.016 | 0.0015 | 361 | 1181 | 498 | 564 |
| 0.075 | 0.27 | 1.36 | 0.015 | 0.002 | 0.054 | 0.002 | 0.066 | 0.055 | 0.004 | 0.042 | 0.037 | 0.013 | 0.0011 | 443 | 1176 | 465 | 577 |
| 0.063 | 0.23 | 1.14 | 0.006 | 0.002 | 0.077 | 0.001 | 0.109 | 0.120 | 0.008 | 0.023 | 0.040 | 0.016 | 0.0010 | 330 | 1237 | 501 | 564 |
| 0.069 | 0.24 | 1.25 | 0.007 | 0.002 | 0.130 | 0.001 | 0.100 | 0.107 | 0.008 | 0.017 | 0.038 | 0.016 | 0.0019 | 278 | 1179 | 500 | 563 |
| 0.073 | 0.24 | 1.32 | 0.007 | 0.002 | 0.036 | 0.002 | 0.034 | 0.034 | 0.005 | 0.043 | 0.036 | 0.013 | 0.0005 | 427 | 1132 | 477 | 559 |
| 0.063 | 0.23 | 1.48 | 0.013 | 0.002 | 0.051 | 0.001 | 0.010 | 0.045 | 0.001 | 0.043 | 0.037 | 0.012 | 0.0008 | 346 | 1207 | 486 | 581 |
| 0.084 | 0.28 | 1.15 | 0.010 | 0.002 | 0.073 | 0.001 | 0.077 | 0.060 | 0.005 | 0.032 | 0.037 | 0.011 | 0.0009 | 436 | 1188 | 483 | 576 |
| 0.073 | 0.22 | 1.58 | 0.013 | 0.003 | 0.152 | 0.002 | 0.069 | 0.088 | 0.009 | 0.017 | 0.030 | 0.010 | 0.0002 | 460 | 1127 | 471 | 571 |
| 0.080 | 0.23 | 1.25 | 0.007 | 0.002 | 0.038 | 0.002 | 0.126 | 0.110 | 0.009 | 0.039 | 0.028 | 0.015 | 0.0020 | 485 | 1200 | 485 | 566 |
| 0.078 | 0.26 | 1.47 | 0.012 | 0.003 | 0.032 | 0.001 | 0.017 | 0.032 | 0.001 | 0.048 | 0.037 | 0.011 | 0.0003 | 456 | 1177 | 484 | 592 |
| 0.064 | 0.25 | 1.48 | 0.014 | 0.003 | 0.070 | 0.001 | 0.036 | 0.042 | 0.008 | 0.033 | 0.042 | 0.015 | 0.0007 | 309 | 1138 | 466 | 568 |
| 0.081 | 0.26 | 1.41 | 0.010 | 0.003 | 0.070 | 0.001 | 0.030 | 0.049 | 0.008 | 0.041 | 0.025 | 0.011 | 0.0012 | 444 | 1122 | 478 | 582 |
| 0.063 | 0.23 | 1.18 | 0.008 | 0.002 | 0.126 | 0.001 | 0.146 | 0.127 | 0.009 | 0.022 | 0.041 | 0.016 | 0.0017 | 355 | 1197 | 491 | 567 |
| 0.078 | 0.25 | 1.47 | 0.011 | 0.003 | 0.037 | 0.001 | 0.026 | 0.042 | 0.001 | 0.047 | 0.038 | 0.011 | 0.0004 | 461 | 1174 | 484 | 591 |
| 0.077 | 0.27 | 1.44 | 0.008 | 0.004 | 0.055 | 0.001 | 0.020 | 0.049 | 0.001 | 0.044 | 0.033 | 0.011 | 0.0008 | 438 | 1135 | 488 | 596 |
| 0.079 | 0.27 | 1.46 | 0.013 | 0.003 | 0.046 | 0.002 | 0.036 | 0.034 | 0.001 | 0.044 | 0.034 | 0.012 | 0.0003 | 337 | 1172 | 481 | 585 |
| 0.081 | 0.25 | 1.42 | 0.014 | 0.003 | 0.033 | 0.001 | 0.024 | 0.034 | 0.001 | 0.048 | 0.036 | 0.012 | 0.0007 | 447 | 1162 | 484 | 591 |
| 0.065 | 0.22 | 1.22 | 0.007 | 0.002 | 0.118 | 0.001 | 0.143 | 0.126 | 0.009 | 0.028 | 0.039 | 0.016 | 0.0025 | 366 | 1187 | 490 | 567 |
| 0.060 | 0.22 | 1.23 | 0.006 | 0.002 | 0.108 | 0.001 | 0.165 | 0.097 | 0.007 | 0.017 | 0.028 | 0.016 | 0.0004 | 352 | 1242 | 499 | 566 |
| 0.078 | 0.23 | 1.43 | 0.014 | 0.003 | 0.016 | 0.001 | 0.060 | 0.067 | 0.001 | 0.037 | 0.036 | 0.011 | 0.0009 | 465 | 1184 | 484 | 590 |
| 0.077 | 0.26 | 1.48 | 0.012 | 0.003 | 0.041 | 0.001 | 0.020 | 0.033 | 0.001 | 0.045 | 0.037 | 0.011 | 0.0003 | 459 | 1094 | 483 | 591 |
| 0.071 | 0.23 | 1.21 | 0.007 | 0.002 | 0.147 | 0.001 | 0.146 | 0.097 | 0.008 | 0.028 | 0.038 | 0.016 | 0.0019 | 373 | 1201 | 492 | 567 |
| 0.082 | 0.28 | 1.47 | 0.013 | 0.003 | 0.039 | 0.002 | 0.018 | 0.037 | 0.000 | 0.047 | 0.036 | 0.011 | 0.0005 | 445 | 1230 | 480 | 593 |
| 0.077 | 0.25 | 1.44 | 0.013 | 0.003 | 0.014 | 0.001 | 0.043 | 0.035 | 0.002 | 0.038 | 0.037 | 0.011 | 0.0006 | 443 | 1180 | 484 | 590 |
| 0.060 | 0.23 | 1.46 | 0.013 | 0.002 | 0.045 | 0.001 | 0.065 | 0.063 | 0.004 | 0.045 | 0.037 | 0.013 | 0.0018 | 403 | 1153 | 478 | 575 |
| 0.064 | 0.24 | 1.15 | 0.007 | 0.002 | 0.109 | 0.001 | 0.143 | 0.128 | 0.009 | 0.024 | 0.041 | 0.016 | 0.0019 | 357 | 1191 | 490 | 567 |
| 0.079 | 0.24 | 1.45 | 0.013 | 0.003 | 0.015 | 0.001 | 0.026 | 0.037 | 0.001 | 0.043 | 0.036 | 0.011 | 0.0004 | 402 | 1184 | 485 | 590 |
| 0.072 | 0.28 | 1.27 | 0.010 | 0.001 | 0.029 | 0.002 | 0.014 | 0.032 | 0.007 | 0.045 | 0.033 | 0.012 | 0.0007 | 444 | 1166 | 471 | 567 |
| 0.076 | 0.27 | 1.51 | 0.008 | 0.003 | 0.017 | 0.001 | 0.030 | 0.045 | 0.004 | 0.043 | 0.038 | 0.012 | 0.0008 | 474 | 1165 | 484 | 593 |
| 0.072 | 0.23 | 1.28 | 0.014 | 0.003 | 0.023 | 0.002 | 0.032 | 0.067 | 0.007 | 0.017 | 0.036 | 0.012 | 0.0014 | 332 | 1163 | 469 | 575 |
| 0.074 | 0.24 | 1.45 | 0.013 | 0.003 | 0.106 | 0.001 | 0.044 | 0.090 | 0.006 | 0.034 | 0.040 | 0.012 | 0.0010 | 338 | 1148 | 480 | 586 |
| 0.080 | 0.27 | 1.50 | 0.007 | 0.004 | 0.026 | 0.001 | 0.027 | 0.064 | 0.002 | 0.046 | 0.033 | 0.013 | 0.0007 | 468 | 1182 | 487 | 596 |
| 0.079 | 0.25 | 1.45 | 0.012 | 0.003 | 0.032 | 0.001 | 0.010 | 0.034 | 0.001 | 0.049 | 0.037 | 0.011 | 0.0006 | 455 | 1062 | 476 | 592 |
| 0.059 | 0.23 | 1.21 | 0.008 | 0.002 | 0.134 | 0.001 | 0.146 | 0.122 | 0.009 | 0.014 | 0.040 | 0.016 | 0.0021 | 359 | 1243 | 499 | 566 |
| 0.081 | 0.24 | 1.42 | 0.012 | 0.003 | 0.043 | 0.001 | 0.028 | 0.038 | 0.001 | 0.044 | 0.035 | 0.011 | 0.0003 | 470 | 1178 | 484 | 591 |
| 0.070 | 0.24 | 1.22 | 0.012 | 0.002 | 0.082 | 0.002 | 0.034 | 0.058 | 0.003 | 0.019 | 0.036 | 0.012 | 0.0011 | 400 | 1181 | 482 | 567 |
| 0.075 | 0.27 | 1.48 | 0.009 | 0.003 | 0.045 | 0.001 | 0.017 | 0.051 | 0.002 | 0.046 | 0.031 | 0.011 | 0.0006 | 471 | 1181 | 487 | 596 |
| 0.077 | 0.25 | 1.43 | 0.013 | 0.004 | 0.048 | 0.002 | 0.036 | 0.021 | 0.003 | 0.041 | 0.040 | 0.011 | 0.0008 | 434 | 1177 | 484 | 590 |
| 0.065 | 0.23 | 1.23 | 0.008 | 0.001 | 0.136 | 0.001 | 0.148 | 0.124 | 0.009 | 0.023 | 0.040 | 0.016 | 0.0023 | 311 | 1188 | 492 | 565 |
| 0.058 | 0.31 | 1.51 | 0.012 | 0.004 | 0.045 | 0.001 | 0.061 | 0.110 | 0.006 | 0.045 | 0.022 | 0.013 | 0.0016 | 345 | 1157 | 484 | 591 |
| 0.075 | 0.26 | 1.48 | 0.013 | 0.003 | 0.016 | 0.001 | 0.024 | 0.034 | 0.003 | 0.038 | 0.037 | 0.011 | 0.0011 | 473 | 1125 | 484 | 590 |
| 0.075 | 0.27 | 1.35 | 0.012 | 0.002 | 0.027 | 0.002 | 0.017 | 0.034 | 0.009 | 0.044 | 0.037 | 0.011 | 0.0009 | 477 | 1176 | 472 | 564 |
| 0.081 | 0.26 | 1.49 | 0.012 | 0.003 | 0.046 | 0.001 | 0.034 | 0.032 | 0.003 | 0.042 | 0.037 | 0.013 | 0.0005 | 434 | 1180 | 484 | 590 |
| 0.077 | 0.27 | 1.42 | 0.013 | 0.002 | 0.084 | 0.001 | 0.045 | 0.038 | 0.001 | 0.028 | 0.037 | 0.012 | 0.0012 | 452 | 1191 | 478 | 590 |
| 0.074 | 0.24 | 1.44 | 0.014 | 0.004 | 0.144 | 0.002 | 0.082 | 0.036 | 0.006 | 0.028 | 0.024 | 0.015 | 0.0014 | 354 | 1143 | 480 | 586 |
| 0.071 | 0.25 | 1.47 | 0.012 | 0.004 | 0.095 | 0.001 | 0.060 | 0.100 | 0.007 | 0.038 | 0.023 | 0.016 | 0.0010 | 358 | 1145 | 478 | 586 |
| 0.061 | 0.23 | 1.20 | 0.008 | 0.002 | 0.145 | 0.001 | 0.141 | 0.121 | 0.008 | 0.019 | 0.044 | 0.016 | 0.0022 | 354 | 1181 | 497 | 566 |
| 0.074 | 0.26 | 1.43 | 0.014 | 0.003 | 0.041 | 0.002 | 0.029 | 0.032 | 0.008 | 0.035 | 0.033 | 0.012 | 0.0008 | 337 | 1157 | 463 | 578 |
| 0.079 | 0.25 | 1.44 | 0.012 | 0.003 | 0.052 | 0.001 | 0.010 | 0.033 | 0.001 | 0.041 | 0.036 | 0.011 | 0.0008 | 452 | 1180 | 484 | 591 |
| 0.080 | 0.26 | 1.45 | 0.014 | 0.004 | 0.048 | 0.001 | 0.018 | 0.042 | 0.000 | 0.044 | 0.037 | 0.012 | 0.0006 | 478 | 958 | 479 | 594 |
| 0.080 | 0.27 | 1.51 | 0.009 | 0.004 | 0.043 | 0.001 | 0.032 | 0.042 | 0.001 | 0.042 | 0.034 | 0.012 | 0.0005 | 470 | 1193 | 487 | 596 |
| 0.081 | 0.25 | 1.49 | 0.012 | 0.003 | 0.050 | 0.001 | 0.038 | 0.136 | 0.008 | 0.023 | 0.028 | 0.013 | 0.0016 | 391 | 1140 | 480 | 587 |
| 0.077 | 0.24 | 1.42 | 0.013 | 0.003 | 0.035 | 0.001 | 0.015 | 0.032 | 0.000 | 0.040 | 0.030 | 0.011 | 0.0005 | 474 | 1070 | 481 | 592 |
| 0.066 | 0.23 | 1.20 | 0.006 | 0.002 | 0.137 | 0.001 | 0.112 | 0.124 | 0.008 | 0.022 | 0.038 | 0.016 | 0.0021 | 355 | 1181 | 497 | 566 |
| 0.077 | 0.26 | 1.44 | 0.013 | 0.003 | 0.029 | 0.001 | 0.023 | 0.041 | 0.001 | 0.045 | 0.037 | 0.012 | 0.0004 | 471 | 1211 | 477 | 593 |
| 0.071 | 0.28 | 1.39 | 0.014 | 0.002 | 0.024 | 0.002 | 0.044 | 0.065 | 0.001 | 0.043 | 0.035 | 0.012 | 0.0010 | 382 | 1192 | 468 | 573 |
| 0.081 | 0.27 | 1.39 | 0.010 | 0.004 | 0.047 | 0.001 | 0.013 | 0.046 | 0.000 | 0.046 | 0.038 | 0.012 | 0.0006 | 468 | 1218 | 490 | 594 |
| 0.082 | 0.24 | 1.23 | 0.013 | 0.003 | 0.018 | 0.003 | 0.009 | 0.095 | 0.009 | 0.007 | 0.040 | 0.012 | 0.0025 | 473 | 1124 | 465 | 582 |
| 0.064 | 0.23 | 1.25 | 0.008 | 0.002 | 0.137 | 0.001 | 0.161 | 0.102 | 0.008 | 0.014 | 0.039 | 0.016 | 0.0021 | 354 | 1184 | 498 | 564 |
| 0.082 | 0.27 | 1.38 | 0.013 | 0.003 | 0.088 | 0.002 | 0.034 | 0.068 | 0.003 | 0.040 | 0.037 | 0.012 | 0.0014 | 350 | 957 | 449 | 575 |
| 0.074 | 0.22 | 1.47 | 0.014 | 0.003 | 0.052 | 0.001 | 0.052 | 0.040 | 0.002 | 0.033 | 0.034 | 0.013 | 0.0012 | 330 | 1232 | 485 | 583 |
| 0.079 | 0.25 | 1.31 | 0.014 | 0.002 | 0.047 | 0.002 | 0.030 | 0.056 | 0.002 | 0.043 | 0.040 | 0.012 | 0.0009 | 318 | 1175 | 468 | 573 |
| 0.076 | 0.29 | 1.37 | 0.015 | 0.002 | 0.038 | 0.002 | 0.038 | 0.042 | 0.002 | 0.044 | 0.037 | 0.011 | 0.0007 | 339 | 1183 | 466 | 574 |
| 0.079 | 0.25 | 1.43 | 0.012 | 0.003 | 0.042 | 0.001 | 0.010 | 0.031 | 0.007 | 0.044 | 0.030 | 0.012 | 0.0006 | 341 | 1158 | 487 | 584 |
| 0.059 | 0.24 | 1.26 | 0.007 | 0.002 | 0.150 | 0.001 | 0.137 | 0.112 | 0.008 | 0.025 | 0.039 | 0.016 | 0.0020 | 354 | 1180 | 497 | 566 |
| 0.074 | 0.25 | 1.40 | 0.012 | 0.003 | 0.029 | 0.001 | 0.015 | 0.039 | 0.001 | 0.038 | 0.036 | 0.011 | 0.0004 | 466 | 1177 | 484 | 591 |
| 0.066 | 0.22 | 1.25 | 0.008 | 0.002 | 0.139 | 0.001 | 0.077 | 0.105 | 0.009 | 0.012 | 0.034 | 0.016 | 0.0022 | 322 | 1255 | 499 | 565 |
| 0.078 | 0.27 | 1.49 | 0.009 | 0.004 | 0.021 | 0.001 | 0.019 | 0.043 | 0.001 | 0.044 | 0.036 | 0.013 | 0.0007 | 466 | 1186 | 487 | 596 |
| 0.054 | 0.31 | 1.34 | 0.015 | 0.004 | 0.141 | 0.002 | 0.069 | 0.043 | 0.008 | 0.029 | 0.035 | 0.012 | 0.0009 | 355 | 1161 | 463 | 578 |
| 0.079 | 0.27 | 1.52 | 0.012 | 0.004 | 0.015 | 0.001 | 0.022 | 0.042 | 0.001 | 0.046 | 0.029 | 0.011 | 0.0004 | 432 | 1228 | 484 | 590 |
| 0.079 | 0.26 | 1.45 | 0.014 | 0.003 | 0.048 | 0.001 | 0.019 | 0.037 | 0.001 | 0.047 | 0.035 | 0.012 | 0.0004 | 471 | 1217 | 477 | 593 |
| 0.077 | 0.28 | 1.36 | 0.014 | 0.002 | 0.069 | 0.002 | 0.069 | 0.056 | 0.003 | 0.039 | 0.037 | 0.013 | 0.0012 | 337 | 1183 | 468 | 573 |
| 0.079 | 0.23 | 1.45 | 0.008 | 0.003 | 0.047 | 0.001 | 0.014 | 0.036 | 0.001 | 0.043 | 0.037 | 0.010 | 0.0005 | 458 | 1175 | 486 | 591 |
| 0.080 | 0.27 | 1.49 | 0.009 | 0.004 | 0.051 | 0.001 | 0.022 | 0.041 | 0.002 | 0.043 | 0.036 | 0.012 | 0.0014 | 474 | 1123 | 484 | 595 |
| 0.080 | 0.24 | 1.47 | 0.014 | 0.003 | 0.016 | 0.002 | 0.032 | 0.038 | 0.004 | 0.045 | 0.039 | 0.013 | 0.0004 | 469 | 1178 | 477 | 593 |
| 0.077 | 0.28 | 1.49 | 0.012 | 0.003 | 0.055 | 0.001 | 0.015 | 0.036 | 0.002 | 0.042 | 0.038 | 0.011 | 0.0008 | 445 | 1178 | 484 | 590 |
| 0.081 | 0.25 | 1.52 | 0.015 | 0.003 | 0.018 | 0.001 | 0.007 | 0.045 | 0.001 | 0.039 | 0.033 | 0.012 | 0.0006 | 472 | 1213 | 486 | 592 |
| 0.077 | 0.28 | 1.44 | 0.007 | 0.003 | 0.073 | 0.001 | 0.054 | 0.043 | 0.001 | 0.036 | 0.038 | 0.012 | 0.0002 | 472 | 1172 | 487 | 596 |
| 0.081 | 0.25 | 1.47 | 0.012 | 0.003 | 0.041 | 0.001 | 0.021 | 0.031 | 0.001 | 0.047 | 0.039 | 0.012 | 0.0006 | 463 | 1125 | 483 | 591 |
| 0.082 | 0.26 | 1.46 | 0.012 | 0.003 | 0.048 | 0.001 | 0.013 | 0.043 | 0.000 | 0.045 | 0.037 | 0.013 | 0.0004 | 452 | 1177 | 484 | 591 |
| 0.061 | 0.23 | 1.20 | 0.008 | 0.002 | 0.144 | 0.001 | 0.130 | 0.097 | 0.007 | 0.019 | 0.038 | 0.016 | 0.0020 | 355 | 1185 | 498 | 564 |
| 0.068 | 0.28 | 1.49 | 0.015 | 0.003 | 0.031 | 0.003 | 0.064 | 0.025 | 0.009 | 0.041 | 0.039 | 0.014 | 0.0010 | 347 | 1143 | 460 | 580 |
| 0.082 | 0.27 | 1.48 | 0.009 | 0.003 | 0.018 | 0.001 | 0.014 | 0.035 | 0.000 | 0.046 | 0.031 | 0.013 | 0.0004 | 476 | 1151 | 488 | 594 |
| 0.076 | 0.27 | 1.48 | 0.012 | 0.002 | 0.076 | 0.002 | 0.054 | 0.055 | 0.003 | 0.046 | 0.034 | 0.012 | 0.0011 | 313 | 1164 | 478 | 584 |
| 0.076 | 0.25 | 1.51 | 0.013 | 0.004 | 0.035 | 0.001 | 0.063 | 0.066 | 0.007 | 0.027 | 0.037 | 0.012 | 0.0011 | 339 | 1160 | 480 | 586 |
| 0.073 | 0.25 | 1.31 | 0.015 | 0.003 | 0.129 | 0.002 | 0.079 | 0.041 | 0.007 | 0.008 | 0.040 | 0.011 | 0.0004 | 370 | 1160 | 463 | 578 |
| 0.060 | 0.28 | 1.44 | 0.014 | 0.004 | 0.089 | 0.002 | 0.038 | 0.051 | 0.006 | 0.040 | 0.024 | 0.013 | 0.0014 | 321 | 1140 | 464 | 581 |
| 0.077 | 0.28 | 1.47 | 0.013 | 0.002 | 0.041 | 0.001 | 0.007 | 0.042 | 0.001 | 0.045 | 0.038 | 0.011 | 0.0006 | 453 | 1181 | 480 | 592 |
| 0.058 | 0.28 | 1.33 | 0.015 | 0.003 | 0.042 | 0.002 | 0.081 | 0.077 | 0.009 | 0.042 | 0.042 | 0.013 | 0.0009 | 355 | 1163 | 463 | 578 |
| 0.074 | 0.22 | 1.49 | 0.012 | 0.003 | 0.071 | 0.001 | 0.078 | 0.077 | 0.007 | 0.037 | 0.037 | 0.016 | 0.0017 | 344 | 1145 | 478 | 586 |
| 0.080 | 0.26 | 1.46 | 0.010 | 0.003 | 0.043 | 0.001 | 0.018 | 0.041 | 0.001 | 0.045 | 0.038 | 0.012 | 0.0004 | 454 | 1175 | 484 | 591 |
| 0.072 | 0.29 | 1.40 | 0.014 | 0.002 | 0.044 | 0.002 | 0.014 | 0.036 | 0.003 | 0.027 | 0.031 | 0.014 | 0.0010 | 374 | 1179 | 468 | 576 |
| 0.078 | 0.25 | 1.48 | 0.012 | 0.003 | 0.064 | 0.001 | 0.020 | 0.044 | 0.002 | 0.039 | 0.034 | 0.011 | 0.0004 | 455 | 1178 | 484 | 590 |
| 0.063 | 0.24 | 1.24 | 0.007 | 0.002 | 0.133 | 0.001 | 0.145 | 0.126 | 0.009 | 0.021 | 0.042 | 0.016 | 0.0023 | 355 | 1189 | 490 | 567 |
| 0.080 | 0.23 | 1.47 | 0.013 | 0.003 | 0.054 | 0.002 | 0.161 | 0.067 | 0.006 | 0.032 | 0.042 | 0.012 | 0.0010 | 481 | 1150 | 477 | 590 |
| 0.078 | 0.26 | 1.47 | 0.013 | 0.003 | 0.015 | 0.001 | 0.025 | 0.035 | 0.001 | 0.047 | 0.033 | 0.011 | 0.0003 | 449 | 1174 | 484 | 591 |
| 0.062 | 0.23 | 1.23 | 0.007 | 0.002 | 0.147 | 0.001 | 0.149 | 0.127 | 0.009 | 0.019 | 0.037 | 0.016 | 0.0021 | 314 | 1240 | 499 | 566 |
| 0.083 | 0.26 | 1.47 | 0.013 | 0.004 | 0.015 | 0.001 | 0.009 | 0.038 | 0.001 | 0.042 | 0.040 | 0.012 | 0.0003 | 464 | 982 | 483 | 592 |
| 0.079 | 0.27 | 1.51 | 0.012 | 0.003 | 0.056 | 0.002 | 0.034 | 0.070 | 0.002 | 0.039 | 0.036 | 0.013 | 0.0005 | 472 | 1230 | 481 | 593 |
| 0.073 | 0.25 | 1.32 | 0.014 | 0.002 | 0.038 | 0.002 | 0.022 | 0.040 | 0.001 | 0.045 | 0.032 | 0.012 | 0.0008 | 310 | 1086 | 456 | 572 |
| 0.053 | 0.31 | 1.37 | 0.014 | 0.003 | 0.017 | 0.002 | 0.093 | 0.038 | 0.007 | 0.006 | 0.042 | 0.012 | 0.0018 | 332 | 1067 | 461 | 575 |
| 0.081 | 0.26 | 1.46 | 0.012 | 0.003 | 0.034 | 0.001 | 0.042 | 0.034 | 0.007 | 0.040 | 0.038 | 0.012 | 0.0005 | 394 | 1057 | 481 | 590 |
| 0.082 | 0.25 | 1.46 | 0.011 | 0.003 | 0.040 | 0.001 | 0.012 | 0.042 | 0.001 | 0.047 | 0.038 | 0.012 | 0.0004 | 454 | 1181 | 484 | 591 |
| 0.077 | 0.30 | 1.52 | 0.013 | 0.003 | 0.114 | 0.002 | 0.066 | 0.066 | 0.005 | 0.030 | 0.033 | 0.011 | 0.0025 | 376 | 1139 | 483 | 589 |
| 0.077 | 0.26 | 1.48 | 0.011 | 0.003 | 0.038 | 0.001 | 0.016 | 0.037 | 0.000 | 0.046 | 0.036 | 0.011 | 0.0005 | 467 | 1180 | 484 | 591 |
| 0.078 | 0.25 | 1.44 | 0.013 | 0.003 | 0.023 | 0.001 | 0.013 | 0.028 | 0.001 | 0.047 | 0.037 | 0.011 | 0.0005 | 463 | 1182 | 481 | 592 |
| 0.074 | 0.27 | 1.42 | 0.008 | 0.004 | 0.041 | 0.001 | 0.012 | 0.039 | 0.001 | 0.043 | 0.038 | 0.011 | 0.0005 | 470 | 1150 | 488 | 594 |
| 0.081 | 0.26 | 1.52 | 0.014 | 0.003 | 0.036 | 0.002 | 0.044 | 0.040 | 0.003 | 0.043 | 0.037 | 0.012 | 0.0005 | 472 | 1178 | 482 | 592 |
|  |  |  |  |  |  |  |  |  |  |  |  |  |  |  |  |  |  |
| **GPR** | | | | | | | | | | | | | | | | | |
| C | Si | Mn | P | S | Cu | Sn | Ni | Cr | Mo | V | Nb | Ti | Ca | Time | Temp | YS | UTS |
| (wt%) | (wt%) | (wt%) | (wt%) | (wt%) | (wt%) | (wt%) | (wt%) | (wt%) | (wt%) | (wt%) | (wt%) | (wt%) | (wt%) | (s) | (℃) | (Mpa) | (Mpa) |
| 0.074 | 0.25 | 1.38 | 0.012 | 0.002 | 0.035 | 0.002 | 0.029 | 0.037 | 0.004 | 0.041 | 0.036 | 0.013 | 0.0005 | 371 | 1161 | 486 | 576 |
| 0.074 | 0.25 | 1.40 | 0.012 | 0.002 | 0.033 | 0.002 | 0.022 | 0.038 | 0.004 | 0.042 | 0.035 | 0.013 | 0.0005 | 374 | 1150 | 485 | 580 |
| 0.075 | 0.26 | 1.40 | 0.012 | 0.003 | 0.037 | 0.002 | 0.026 | 0.043 | 0.003 | 0.041 | 0.035 | 0.013 | 0.0005 | 369 | 1148 | 483 | 580 |
| 0.075 | 0.25 | 1.40 | 0.012 | 0.002 | 0.036 | 0.001 | 0.028 | 0.037 | 0.003 | 0.042 | 0.035 | 0.012 | 0.0004 | 376 | 1148 | 483 | 582 |
| 0.074 | 0.26 | 1.40 | 0.012 | 0.002 | 0.037 | 0.002 | 0.028 | 0.040 | 0.003 | 0.041 | 0.035 | 0.013 | 0.0006 | 365 | 1147 | 484 | 579 |
| 0.074 | 0.26 | 1.39 | 0.012 | 0.002 | 0.039 | 0.002 | 0.027 | 0.039 | 0.003 | 0.040 | 0.035 | 0.013 | 0.0005 | 370 | 1145 | 484 | 578 |
| 0.074 | 0.25 | 1.39 | 0.012 | 0.002 | 0.034 | 0.001 | 0.026 | 0.039 | 0.004 | 0.041 | 0.035 | 0.013 | 0.0005 | 373 | 1140 | 485 | 578 |
| 0.075 | 0.25 | 1.39 | 0.012 | 0.003 | 0.035 | 0.001 | 0.023 | 0.044 | 0.003 | 0.040 | 0.037 | 0.013 | 0.0005 | 380 | 1152 | 482 | 580 |
| 0.075 | 0.25 | 1.40 | 0.012 | 0.002 | 0.035 | 0.001 | 0.021 | 0.036 | 0.003 | 0.041 | 0.035 | 0.012 | 0.0005 | 375 | 1149 | 484 | 581 |
| 0.074 | 0.25 | 1.39 | 0.012 | 0.002 | 0.042 | 0.001 | 0.033 | 0.045 | 0.004 | 0.041 | 0.037 | 0.013 | 0.0006 | 376 | 1152 | 484 | 576 |
| 0.074 | 0.26 | 1.40 | 0.012 | 0.003 | 0.038 | 0.002 | 0.026 | 0.039 | 0.003 | 0.041 | 0.037 | 0.013 | 0.0005 | 355 | 1150 | 484 | 578 |
| 0.074 | 0.25 | 1.40 | 0.012 | 0.002 | 0.034 | 0.002 | 0.025 | 0.037 | 0.003 | 0.041 | 0.035 | 0.013 | 0.0005 | 372 | 1150 | 484 | 580 |
| 0.075 | 0.25 | 1.41 | 0.012 | 0.003 | 0.031 | 0.001 | 0.022 | 0.040 | 0.003 | 0.041 | 0.035 | 0.013 | 0.0005 | 366 | 1150 | 484 | 581 |
| 0.074 | 0.25 | 1.40 | 0.012 | 0.003 | 0.034 | 0.001 | 0.022 | 0.038 | 0.004 | 0.041 | 0.035 | 0.013 | 0.0005 | 373 | 1159 | 485 | 579 |
| 0.074 | 0.25 | 1.39 | 0.012 | 0.002 | 0.033 | 0.002 | 0.025 | 0.036 | 0.003 | 0.040 | 0.036 | 0.013 | 0.0005 | 370 | 1148 | 485 | 580 |
| 0.073 | 0.25 | 1.39 | 0.012 | 0.002 | 0.038 | 0.002 | 0.032 | 0.040 | 0.004 | 0.038 | 0.034 | 0.013 | 0.0005 | 371 | 1162 | 485 | 574 |
| 0.074 | 0.25 | 1.40 | 0.012 | 0.002 | 0.034 | 0.002 | 0.029 | 0.040 | 0.004 | 0.040 | 0.035 | 0.013 | 0.0005 | 369 | 1154 | 485 | 578 |
| 0.076 | 0.25 | 1.40 | 0.012 | 0.003 | 0.032 | 0.001 | 0.022 | 0.039 | 0.003 | 0.042 | 0.035 | 0.012 | 0.0004 | 379 | 1145 | 482 | 585 |
| 0.074 | 0.25 | 1.39 | 0.012 | 0.002 | 0.033 | 0.002 | 0.024 | 0.037 | 0.004 | 0.041 | 0.035 | 0.013 | 0.0006 | 375 | 1152 | 485 | 579 |
| 0.074 | 0.25 | 1.41 | 0.012 | 0.002 | 0.032 | 0.001 | 0.028 | 0.038 | 0.004 | 0.041 | 0.035 | 0.012 | 0.0005 | 373 | 1138 | 485 | 579 |
| 0.074 | 0.26 | 1.39 | 0.012 | 0.002 | 0.035 | 0.002 | 0.030 | 0.039 | 0.003 | 0.040 | 0.036 | 0.013 | 0.0005 | 373 | 1160 | 484 | 579 |
| 0.074 | 0.25 | 1.39 | 0.012 | 0.003 | 0.028 | 0.001 | 0.019 | 0.035 | 0.004 | 0.042 | 0.035 | 0.013 | 0.0005 | 376 | 1150 | 484 | 581 |
| 0.076 | 0.25 | 1.40 | 0.013 | 0.003 | 0.031 | 0.001 | 0.021 | 0.036 | 0.003 | 0.041 | 0.036 | 0.012 | 0.0004 | 377 | 1137 | 482 | 584 |
| 0.074 | 0.25 | 1.39 | 0.012 | 0.002 | 0.035 | 0.001 | 0.025 | 0.037 | 0.004 | 0.042 | 0.035 | 0.012 | 0.0004 | 374 | 1136 | 484 | 580 |
| 0.074 | 0.25 | 1.39 | 0.012 | 0.002 | 0.032 | 0.002 | 0.020 | 0.038 | 0.004 | 0.041 | 0.035 | 0.013 | 0.0005 | 371 | 1150 | 485 | 580 |
| 0.074 | 0.25 | 1.40 | 0.012 | 0.002 | 0.038 | 0.001 | 0.025 | 0.041 | 0.003 | 0.038 | 0.035 | 0.013 | 0.0005 | 369 | 1149 | 484 | 579 |
| 0.076 | 0.25 | 1.40 | 0.012 | 0.003 | 0.033 | 0.001 | 0.024 | 0.038 | 0.003 | 0.042 | 0.036 | 0.013 | 0.0004 | 373 | 1141 | 483 | 583 |
| 0.074 | 0.25 | 1.40 | 0.012 | 0.002 | 0.033 | 0.002 | 0.025 | 0.038 | 0.003 | 0.042 | 0.035 | 0.013 | 0.0005 | 371 | 1152 | 485 | 579 |
| 0.072 | 0.25 | 1.39 | 0.012 | 0.002 | 0.034 | 0.002 | 0.028 | 0.036 | 0.003 | 0.039 | 0.035 | 0.013 | 0.0005 | 374 | 1146 | 485 | 577 |
| 0.074 | 0.25 | 1.39 | 0.012 | 0.002 | 0.034 | 0.001 | 0.026 | 0.037 | 0.004 | 0.041 | 0.035 | 0.012 | 0.0006 | 379 | 1144 | 485 | 579 |
| 0.076 | 0.26 | 1.41 | 0.012 | 0.003 | 0.031 | 0.001 | 0.023 | 0.042 | 0.003 | 0.041 | 0.036 | 0.013 | 0.0005 | 376 | 1144 | 482 | 584 |
| 0.074 | 0.25 | 1.40 | 0.012 | 0.002 | 0.033 | 0.002 | 0.022 | 0.035 | 0.003 | 0.040 | 0.035 | 0.013 | 0.0005 | 374 | 1157 | 485 | 579 |
| 0.074 | 0.25 | 1.40 | 0.012 | 0.002 | 0.034 | 0.002 | 0.027 | 0.037 | 0.003 | 0.041 | 0.035 | 0.013 | 0.0005 | 372 | 1145 | 484 | 580 |
| 0.074 | 0.25 | 1.39 | 0.012 | 0.002 | 0.037 | 0.002 | 0.022 | 0.037 | 0.003 | 0.041 | 0.035 | 0.013 | 0.0005 | 384 | 1144 | 484 | 579 |
| 0.074 | 0.25 | 1.37 | 0.012 | 0.002 | 0.035 | 0.002 | 0.026 | 0.040 | 0.003 | 0.041 | 0.035 | 0.013 | 0.0005 | 356 | 1149 | 484 | 576 |
| 0.075 | 0.26 | 1.40 | 0.012 | 0.003 | 0.033 | 0.002 | 0.023 | 0.037 | 0.003 | 0.042 | 0.034 | 0.013 | 0.0004 | 382 | 1148 | 483 | 581 |
| 0.075 | 0.25 | 1.40 | 0.012 | 0.003 | 0.036 | 0.001 | 0.034 | 0.038 | 0.003 | 0.041 | 0.036 | 0.013 | 0.0005 | 375 | 1153 | 484 | 580 |
| 0.074 | 0.25 | 1.39 | 0.012 | 0.002 | 0.037 | 0.001 | 0.026 | 0.035 | 0.004 | 0.039 | 0.035 | 0.013 | 0.0005 | 373 | 1157 | 484 | 578 |
| 0.074 | 0.25 | 1.39 | 0.012 | 0.003 | 0.035 | 0.001 | 0.024 | 0.036 | 0.003 | 0.041 | 0.036 | 0.013 | 0.0005 | 379 | 1147 | 484 | 581 |
| 0.074 | 0.25 | 1.40 | 0.012 | 0.002 | 0.036 | 0.001 | 0.021 | 0.038 | 0.004 | 0.042 | 0.035 | 0.012 | 0.0005 | 370 | 1143 | 484 | 581 |
| 0.074 | 0.25 | 1.39 | 0.012 | 0.002 | 0.038 | 0.002 | 0.024 | 0.036 | 0.004 | 0.040 | 0.036 | 0.013 | 0.0004 | 364 | 1150 | 485 | 578 |
| 0.074 | 0.25 | 1.39 | 0.012 | 0.002 | 0.034 | 0.001 | 0.020 | 0.039 | 0.004 | 0.041 | 0.035 | 0.013 | 0.0006 | 372 | 1163 | 485 | 578 |
| 0.075 | 0.25 | 1.41 | 0.012 | 0.003 | 0.035 | 0.001 | 0.027 | 0.038 | 0.003 | 0.040 | 0.036 | 0.013 | 0.0005 | 373 | 1151 | 484 | 581 |
| 0.076 | 0.25 | 1.39 | 0.012 | 0.003 | 0.034 | 0.001 | 0.023 | 0.037 | 0.003 | 0.041 | 0.036 | 0.013 | 0.0005 | 372 | 1133 | 483 | 582 |
| 0.074 | 0.25 | 1.39 | 0.012 | 0.003 | 0.034 | 0.002 | 0.031 | 0.037 | 0.003 | 0.041 | 0.036 | 0.012 | 0.0004 | 380 | 1145 | 484 | 580 |
| 0.075 | 0.25 | 1.41 | 0.012 | 0.003 | 0.032 | 0.001 | 0.022 | 0.037 | 0.003 | 0.042 | 0.035 | 0.013 | 0.0005 | 366 | 1145 | 484 | 582 |
| 0.075 | 0.25 | 1.40 | 0.012 | 0.003 | 0.031 | 0.001 | 0.028 | 0.038 | 0.003 | 0.041 | 0.035 | 0.012 | 0.0004 | 368 | 1140 | 483 | 582 |
| 0.074 | 0.25 | 1.40 | 0.012 | 0.002 | 0.035 | 0.002 | 0.023 | 0.040 | 0.004 | 0.041 | 0.035 | 0.013 | 0.0005 | 371 | 1152 | 486 | 578 |
| 0.074 | 0.25 | 1.40 | 0.012 | 0.003 | 0.039 | 0.001 | 0.024 | 0.039 | 0.003 | 0.042 | 0.036 | 0.013 | 0.0004 | 374 | 1157 | 484 | 581 |
| 0.074 | 0.25 | 1.40 | 0.012 | 0.003 | 0.037 | 0.001 | 0.026 | 0.039 | 0.004 | 0.041 | 0.034 | 0.013 | 0.0004 | 375 | 1153 | 485 | 579 |
| 0.074 | 0.25 | 1.39 | 0.012 | 0.003 | 0.032 | 0.001 | 0.019 | 0.040 | 0.004 | 0.042 | 0.035 | 0.013 | 0.0004 | 380 | 1150 | 484 | 581 |
| 0.075 | 0.25 | 1.39 | 0.012 | 0.003 | 0.032 | 0.002 | 0.025 | 0.039 | 0.003 | 0.041 | 0.035 | 0.012 | 0.0004 | 369 | 1136 | 483 | 581 |
| 0.074 | 0.25 | 1.40 | 0.012 | 0.003 | 0.037 | 0.001 | 0.024 | 0.039 | 0.004 | 0.041 | 0.035 | 0.013 | 0.0006 | 368 | 1150 | 484 | 579 |
| 0.075 | 0.25 | 1.39 | 0.012 | 0.003 | 0.033 | 0.001 | 0.025 | 0.036 | 0.003 | 0.043 | 0.036 | 0.013 | 0.0005 | 374 | 1138 | 483 | 582 |
| 0.074 | 0.26 | 1.39 | 0.012 | 0.002 | 0.035 | 0.002 | 0.028 | 0.037 | 0.003 | 0.040 | 0.035 | 0.013 | 0.0005 | 364 | 1158 | 484 | 578 |
| 0.074 | 0.25 | 1.40 | 0.012 | 0.002 | 0.035 | 0.001 | 0.025 | 0.038 | 0.004 | 0.041 | 0.035 | 0.013 | 0.0005 | 378 | 1149 | 485 | 579 |
| 0.074 | 0.25 | 1.40 | 0.012 | 0.002 | 0.034 | 0.001 | 0.025 | 0.038 | 0.004 | 0.041 | 0.035 | 0.013 | 0.0005 | 370 | 1152 | 485 | 579 |
| 0.074 | 0.25 | 1.39 | 0.012 | 0.002 | 0.036 | 0.001 | 0.024 | 0.040 | 0.004 | 0.041 | 0.035 | 0.012 | 0.0005 | 377 | 1152 | 485 | 578 |
| 0.074 | 0.26 | 1.41 | 0.012 | 0.003 | 0.033 | 0.002 | 0.024 | 0.037 | 0.003 | 0.040 | 0.035 | 0.013 | 0.0005 | 366 | 1147 | 484 | 580 |
| 0.074 | 0.25 | 1.38 | 0.012 | 0.002 | 0.034 | 0.002 | 0.021 | 0.039 | 0.003 | 0.041 | 0.036 | 0.012 | 0.0005 | 378 | 1148 | 485 | 579 |
| 0.075 | 0.25 | 1.41 | 0.012 | 0.003 | 0.037 | 0.001 | 0.021 | 0.041 | 0.003 | 0.040 | 0.035 | 0.013 | 0.0005 | 376 | 1157 | 484 | 581 |
| 0.075 | 0.26 | 1.41 | 0.012 | 0.003 | 0.031 | 0.001 | 0.022 | 0.036 | 0.003 | 0.042 | 0.036 | 0.013 | 0.0005 | 374 | 1155 | 484 | 582 |
| 0.076 | 0.26 | 1.42 | 0.012 | 0.003 | 0.039 | 0.001 | 0.017 | 0.038 | 0.003 | 0.042 | 0.035 | 0.012 | 0.0004 | 375 | 1143 | 482 | 584 |
| 0.074 | 0.26 | 1.37 | 0.012 | 0.002 | 0.036 | 0.002 | 0.027 | 0.038 | 0.003 | 0.042 | 0.036 | 0.013 | 0.0005 | 374 | 1153 | 483 | 579 |
| 0.073 | 0.25 | 1.40 | 0.012 | 0.002 | 0.036 | 0.001 | 0.034 | 0.040 | 0.004 | 0.041 | 0.035 | 0.013 | 0.0005 | 367 | 1166 | 485 | 577 |
| 0.075 | 0.25 | 1.40 | 0.012 | 0.003 | 0.032 | 0.001 | 0.024 | 0.036 | 0.004 | 0.041 | 0.037 | 0.013 | 0.0005 | 369 | 1147 | 484 | 581 |
| 0.075 | 0.26 | 1.41 | 0.012 | 0.003 | 0.032 | 0.001 | 0.021 | 0.037 | 0.003 | 0.041 | 0.035 | 0.013 | 0.0005 | 374 | 1142 | 483 | 583 |
| 0.073 | 0.25 | 1.37 | 0.011 | 0.002 | 0.040 | 0.002 | 0.035 | 0.042 | 0.003 | 0.039 | 0.036 | 0.013 | 0.0007 | 353 | 1166 | 483 | 570 |
| 0.074 | 0.25 | 1.40 | 0.012 | 0.002 | 0.038 | 0.002 | 0.027 | 0.040 | 0.003 | 0.041 | 0.035 | 0.013 | 0.0005 | 378 | 1152 | 484 | 579 |
| 0.075 | 0.25 | 1.40 | 0.012 | 0.003 | 0.035 | 0.001 | 0.025 | 0.037 | 0.004 | 0.042 | 0.036 | 0.012 | 0.0005 | 377 | 1142 | 484 | 582 |
| 0.074 | 0.25 | 1.37 | 0.012 | 0.002 | 0.043 | 0.001 | 0.025 | 0.038 | 0.003 | 0.040 | 0.035 | 0.013 | 0.0005 | 369 | 1156 | 483 | 577 |
| 0.074 | 0.25 | 1.39 | 0.012 | 0.002 | 0.040 | 0.001 | 0.026 | 0.041 | 0.004 | 0.040 | 0.035 | 0.013 | 0.0005 | 370 | 1156 | 485 | 577 |
| 0.075 | 0.25 | 1.41 | 0.012 | 0.003 | 0.031 | 0.001 | 0.027 | 0.038 | 0.003 | 0.043 | 0.036 | 0.013 | 0.0005 | 368 | 1138 | 482 | 584 |
| 0.075 | 0.25 | 1.40 | 0.012 | 0.003 | 0.038 | 0.001 | 0.024 | 0.037 | 0.003 | 0.042 | 0.035 | 0.013 | 0.0005 | 375 | 1143 | 483 | 582 |
| 0.075 | 0.25 | 1.40 | 0.012 | 0.002 | 0.029 | 0.001 | 0.023 | 0.039 | 0.004 | 0.042 | 0.035 | 0.012 | 0.0005 | 376 | 1154 | 484 | 581 |
| 0.075 | 0.25 | 1.40 | 0.012 | 0.003 | 0.032 | 0.002 | 0.021 | 0.035 | 0.003 | 0.042 | 0.037 | 0.013 | 0.0004 | 371 | 1150 | 484 | 581 |
| 0.075 | 0.25 | 1.39 | 0.012 | 0.003 | 0.036 | 0.001 | 0.025 | 0.039 | 0.003 | 0.041 | 0.035 | 0.013 | 0.0005 | 374 | 1151 | 484 | 580 |
| 0.074 | 0.25 | 1.39 | 0.012 | 0.002 | 0.038 | 0.002 | 0.028 | 0.038 | 0.004 | 0.041 | 0.034 | 0.013 | 0.0005 | 366 | 1160 | 485 | 577 |
| 0.074 | 0.25 | 1.39 | 0.012 | 0.002 | 0.036 | 0.001 | 0.026 | 0.042 | 0.004 | 0.041 | 0.036 | 0.013 | 0.0005 | 376 | 1156 | 485 | 577 |
| 0.074 | 0.25 | 1.39 | 0.012 | 0.002 | 0.035 | 0.002 | 0.036 | 0.037 | 0.003 | 0.041 | 0.036 | 0.013 | 0.0005 | 368 | 1155 | 485 | 577 |
| 0.075 | 0.25 | 1.38 | 0.012 | 0.003 | 0.033 | 0.001 | 0.024 | 0.039 | 0.003 | 0.041 | 0.037 | 0.013 | 0.0005 | 376 | 1152 | 483 | 581 |
| 0.075 | 0.26 | 1.41 | 0.012 | 0.003 | 0.034 | 0.002 | 0.023 | 0.040 | 0.003 | 0.042 | 0.035 | 0.012 | 0.0005 | 370 | 1147 | 483 | 582 |
| 0.075 | 0.25 | 1.41 | 0.013 | 0.003 | 0.032 | 0.001 | 0.021 | 0.038 | 0.003 | 0.043 | 0.035 | 0.013 | 0.0004 | 377 | 1139 | 482 | 584 |
| 0.075 | 0.25 | 1.39 | 0.013 | 0.003 | 0.037 | 0.001 | 0.022 | 0.037 | 0.003 | 0.042 | 0.036 | 0.012 | 0.0004 | 373 | 1143 | 483 | 582 |
| 0.075 | 0.25 | 1.39 | 0.012 | 0.002 | 0.034 | 0.001 | 0.022 | 0.039 | 0.003 | 0.042 | 0.036 | 0.013 | 0.0005 | 368 | 1144 | 484 | 581 |
| 0.074 | 0.25 | 1.40 | 0.012 | 0.002 | 0.036 | 0.001 | 0.023 | 0.037 | 0.003 | 0.042 | 0.035 | 0.013 | 0.0006 | 368 | 1148 | 484 | 579 |
| 0.074 | 0.25 | 1.40 | 0.012 | 0.003 | 0.036 | 0.002 | 0.027 | 0.038 | 0.004 | 0.040 | 0.035 | 0.013 | 0.0005 | 374 | 1150 | 484 | 579 |
| 0.075 | 0.25 | 1.39 | 0.012 | 0.002 | 0.035 | 0.001 | 0.027 | 0.042 | 0.003 | 0.042 | 0.035 | 0.013 | 0.0005 | 376 | 1146 | 483 | 580 |
| 0.075 | 0.25 | 1.40 | 0.012 | 0.003 | 0.041 | 0.001 | 0.024 | 0.037 | 0.004 | 0.040 | 0.036 | 0.013 | 0.0004 | 374 | 1144 | 483 | 581 |
| 0.075 | 0.26 | 1.40 | 0.012 | 0.003 | 0.035 | 0.002 | 0.020 | 0.037 | 0.003 | 0.042 | 0.036 | 0.013 | 0.0005 | 373 | 1150 | 483 | 582 |
| 0.075 | 0.25 | 1.41 | 0.012 | 0.003 | 0.038 | 0.001 | 0.025 | 0.036 | 0.003 | 0.041 | 0.035 | 0.013 | 0.0005 | 367 | 1145 | 484 | 581 |
| 0.075 | 0.25 | 1.41 | 0.012 | 0.002 | 0.031 | 0.002 | 0.021 | 0.036 | 0.003 | 0.040 | 0.035 | 0.012 | 0.0005 | 376 | 1143 | 484 | 580 |
| 0.074 | 0.25 | 1.39 | 0.012 | 0.002 | 0.033 | 0.001 | 0.023 | 0.037 | 0.004 | 0.040 | 0.035 | 0.013 | 0.0005 | 375 | 1151 | 485 | 579 |
| 0.075 | 0.25 | 1.39 | 0.012 | 0.003 | 0.033 | 0.001 | 0.026 | 0.038 | 0.004 | 0.042 | 0.035 | 0.013 | 0.0005 | 377 | 1151 | 484 | 581 |
| 0.074 | 0.26 | 1.39 | 0.012 | 0.002 | 0.041 | 0.002 | 0.032 | 0.038 | 0.003 | 0.041 | 0.035 | 0.013 | 0.0005 | 374 | 1154 | 484 | 577 |
| 0.074 | 0.25 | 1.41 | 0.012 | 0.003 | 0.034 | 0.001 | 0.023 | 0.040 | 0.003 | 0.041 | 0.035 | 0.013 | 0.0005 | 371 | 1155 | 484 | 580 |
| 0.074 | 0.25 | 1.40 | 0.012 | 0.002 | 0.035 | 0.002 | 0.028 | 0.040 | 0.003 | 0.041 | 0.035 | 0.013 | 0.0006 | 372 | 1153 | 485 | 577 |
| 0.074 | 0.25 | 1.38 | 0.012 | 0.002 | 0.034 | 0.002 | 0.021 | 0.037 | 0.003 | 0.041 | 0.036 | 0.013 | 0.0005 | 373 | 1156 | 485 | 579 |
| 0.075 | 0.26 | 1.40 | 0.012 | 0.003 | 0.035 | 0.001 | 0.022 | 0.036 | 0.003 | 0.042 | 0.036 | 0.013 | 0.0004 | 379 | 1146 | 484 | 582 |
| 0.075 | 0.25 | 1.40 | 0.012 | 0.003 | 0.031 | 0.001 | 0.027 | 0.040 | 0.004 | 0.042 | 0.035 | 0.013 | 0.0005 | 379 | 1130 | 483 | 582 |
| 0.074 | 0.25 | 1.39 | 0.012 | 0.003 | 0.036 | 0.001 | 0.025 | 0.038 | 0.004 | 0.042 | 0.036 | 0.012 | 0.0004 | 375 | 1143 | 484 | 580 |
| 0.075 | 0.25 | 1.39 | 0.012 | 0.003 | 0.032 | 0.001 | 0.020 | 0.041 | 0.004 | 0.041 | 0.035 | 0.013 | 0.0005 | 372 | 1157 | 484 | 580 |
| 0.076 | 0.25 | 1.41 | 0.012 | 0.003 | 0.035 | 0.001 | 0.024 | 0.039 | 0.003 | 0.042 | 0.035 | 0.013 | 0.0005 | 373 | 1143 | 482 | 584 |
| 0.074 | 0.25 | 1.40 | 0.012 | 0.002 | 0.038 | 0.001 | 0.022 | 0.038 | 0.004 | 0.038 | 0.035 | 0.013 | 0.0005 | 371 | 1160 | 485 | 577 |
| 0.074 | 0.25 | 1.40 | 0.012 | 0.002 | 0.039 | 0.002 | 0.025 | 0.037 | 0.004 | 0.040 | 0.035 | 0.013 | 0.0005 | 375 | 1155 | 485 | 577 |
| 0.074 | 0.25 | 1.38 | 0.012 | 0.002 | 0.033 | 0.002 | 0.027 | 0.039 | 0.004 | 0.041 | 0.036 | 0.013 | 0.0005 | 371 | 1153 | 485 | 577 |
| 0.075 | 0.25 | 1.40 | 0.012 | 0.003 | 0.032 | 0.001 | 0.020 | 0.036 | 0.003 | 0.043 | 0.036 | 0.012 | 0.0005 | 381 | 1130 | 482 | 584 |
| 0.075 | 0.25 | 1.39 | 0.012 | 0.003 | 0.033 | 0.001 | 0.025 | 0.038 | 0.003 | 0.041 | 0.035 | 0.013 | 0.0005 | 374 | 1143 | 483 | 581 |
| 0.074 | 0.25 | 1.41 | 0.012 | 0.002 | 0.036 | 0.001 | 0.025 | 0.037 | 0.004 | 0.041 | 0.035 | 0.013 | 0.0005 | 373 | 1154 | 485 | 579 |
| 0.075 | 0.26 | 1.40 | 0.012 | 0.003 | 0.038 | 0.001 | 0.025 | 0.037 | 0.003 | 0.040 | 0.035 | 0.013 | 0.0005 | 373 | 1146 | 483 | 582 |
| 0.074 | 0.26 | 1.40 | 0.012 | 0.002 | 0.038 | 0.001 | 0.035 | 0.040 | 0.003 | 0.040 | 0.036 | 0.013 | 0.0005 | 364 | 1158 | 484 | 578 |
| 0.074 | 0.25 | 1.41 | 0.012 | 0.003 | 0.033 | 0.001 | 0.027 | 0.039 | 0.003 | 0.042 | 0.035 | 0.013 | 0.0005 | 371 | 1151 | 484 | 581 |
| 0.075 | 0.25 | 1.40 | 0.012 | 0.003 | 0.035 | 0.002 | 0.021 | 0.039 | 0.003 | 0.042 | 0.036 | 0.013 | 0.0005 | 368 | 1147 | 484 | 581 |
| 0.076 | 0.25 | 1.40 | 0.012 | 0.003 | 0.030 | 0.001 | 0.018 | 0.038 | 0.003 | 0.042 | 0.035 | 0.012 | 0.0005 | 377 | 1146 | 483 | 583 |
| 0.074 | 0.25 | 1.39 | 0.012 | 0.002 | 0.037 | 0.001 | 0.027 | 0.036 | 0.004 | 0.041 | 0.035 | 0.013 | 0.0005 | 366 | 1147 | 484 | 579 |
| 0.074 | 0.26 | 1.38 | 0.012 | 0.002 | 0.034 | 0.001 | 0.024 | 0.037 | 0.004 | 0.041 | 0.035 | 0.013 | 0.0005 | 374 | 1143 | 484 | 579 |
| 0.074 | 0.25 | 1.40 | 0.012 | 0.002 | 0.033 | 0.002 | 0.022 | 0.036 | 0.003 | 0.041 | 0.035 | 0.013 | 0.0005 | 369 | 1146 | 485 | 580 |
| 0.074 | 0.25 | 1.39 | 0.012 | 0.002 | 0.036 | 0.001 | 0.025 | 0.039 | 0.003 | 0.039 | 0.034 | 0.013 | 0.0005 | 377 | 1153 | 484 | 578 |
| 0.074 | 0.25 | 1.40 | 0.012 | 0.003 | 0.032 | 0.001 | 0.027 | 0.037 | 0.003 | 0.042 | 0.036 | 0.013 | 0.0004 | 374 | 1141 | 484 | 582 |
| 0.076 | 0.25 | 1.40 | 0.013 | 0.003 | 0.036 | 0.001 | 0.021 | 0.037 | 0.003 | 0.042 | 0.035 | 0.013 | 0.0005 | 369 | 1138 | 482 | 584 |
| 0.074 | 0.25 | 1.40 | 0.012 | 0.002 | 0.032 | 0.002 | 0.026 | 0.036 | 0.003 | 0.040 | 0.036 | 0.013 | 0.0005 | 370 | 1148 | 485 | 579 |
| 0.073 | 0.25 | 1.39 | 0.012 | 0.002 | 0.034 | 0.001 | 0.022 | 0.040 | 0.004 | 0.041 | 0.036 | 0.013 | 0.0005 | 376 | 1152 | 486 | 577 |
| 0.074 | 0.25 | 1.41 | 0.012 | 0.002 | 0.030 | 0.001 | 0.025 | 0.040 | 0.004 | 0.041 | 0.035 | 0.013 | 0.0004 | 367 | 1153 | 485 | 578 |
| 0.074 | 0.26 | 1.40 | 0.012 | 0.003 | 0.034 | 0.002 | 0.020 | 0.036 | 0.003 | 0.042 | 0.035 | 0.013 | 0.0005 | 371 | 1153 | 484 | 580 |
| 0.072 | 0.25 | 1.40 | 0.012 | 0.002 | 0.034 | 0.002 | 0.031 | 0.040 | 0.004 | 0.039 | 0.035 | 0.013 | 0.0005 | 372 | 1159 | 486 | 575 |
| 0.075 | 0.25 | 1.40 | 0.012 | 0.003 | 0.031 | 0.001 | 0.025 | 0.037 | 0.003 | 0.042 | 0.036 | 0.013 | 0.0005 | 376 | 1150 | 483 | 583 |
| 0.075 | 0.25 | 1.42 | 0.012 | 0.003 | 0.035 | 0.001 | 0.023 | 0.036 | 0.004 | 0.042 | 0.035 | 0.013 | 0.0005 | 370 | 1153 | 485 | 581 |
| 0.077 | 0.25 | 1.42 | 0.013 | 0.003 | 0.029 | 0.001 | 0.024 | 0.041 | 0.002 | 0.044 | 0.036 | 0.012 | 0.0004 | 387 | 1141 | 479 | 591 |
| 0.075 | 0.26 | 1.40 | 0.012 | 0.002 | 0.033 | 0.002 | 0.024 | 0.037 | 0.004 | 0.042 | 0.035 | 0.012 | 0.0005 | 360 | 1136 | 484 | 579 |
| 0.076 | 0.25 | 1.41 | 0.012 | 0.002 | 0.031 | 0.001 | 0.021 | 0.035 | 0.003 | 0.041 | 0.035 | 0.012 | 0.0004 | 374 | 1140 | 483 | 583 |
| 0.075 | 0.25 | 1.40 | 0.013 | 0.003 | 0.036 | 0.001 | 0.023 | 0.037 | 0.002 | 0.042 | 0.036 | 0.013 | 0.0005 | 373 | 1133 | 481 | 585 |
| 0.074 | 0.25 | 1.40 | 0.012 | 0.002 | 0.032 | 0.002 | 0.025 | 0.039 | 0.004 | 0.040 | 0.034 | 0.013 | 0.0005 | 370 | 1155 | 485 | 577 |
| 0.075 | 0.26 | 1.40 | 0.012 | 0.003 | 0.038 | 0.002 | 0.029 | 0.039 | 0.003 | 0.040 | 0.035 | 0.013 | 0.0005 | 376 | 1152 | 483 | 580 |
| 0.074 | 0.25 | 1.39 | 0.012 | 0.002 | 0.036 | 0.001 | 0.025 | 0.041 | 0.004 | 0.039 | 0.034 | 0.013 | 0.0005 | 364 | 1150 | 484 | 578 |
| 0.074 | 0.25 | 1.40 | 0.012 | 0.002 | 0.035 | 0.002 | 0.025 | 0.037 | 0.004 | 0.041 | 0.035 | 0.013 | 0.0005 | 374 | 1149 | 485 | 579 |
| 0.074 | 0.25 | 1.40 | 0.012 | 0.002 | 0.036 | 0.001 | 0.025 | 0.039 | 0.004 | 0.040 | 0.037 | 0.013 | 0.0005 | 379 | 1156 | 485 | 578 |
| 0.075 | 0.25 | 1.39 | 0.012 | 0.003 | 0.032 | 0.001 | 0.025 | 0.038 | 0.003 | 0.043 | 0.035 | 0.013 | 0.0005 | 370 | 1146 | 483 | 582 |
| 0.076 | 0.25 | 1.40 | 0.012 | 0.003 | 0.032 | 0.002 | 0.025 | 0.040 | 0.003 | 0.043 | 0.036 | 0.013 | 0.0005 | 374 | 1143 | 483 | 582 |
| 0.074 | 0.26 | 1.39 | 0.012 | 0.002 | 0.037 | 0.002 | 0.025 | 0.041 | 0.004 | 0.040 | 0.035 | 0.013 | 0.0005 | 373 | 1142 | 484 | 579 |
| 0.074 | 0.26 | 1.38 | 0.012 | 0.002 | 0.034 | 0.002 | 0.020 | 0.038 | 0.003 | 0.040 | 0.035 | 0.013 | 0.0005 | 376 | 1155 | 484 | 579 |
| 0.075 | 0.25 | 1.39 | 0.012 | 0.003 | 0.038 | 0.001 | 0.023 | 0.040 | 0.003 | 0.042 | 0.035 | 0.013 | 0.0005 | 368 | 1155 | 484 | 580 |
| 0.074 | 0.25 | 1.40 | 0.012 | 0.003 | 0.033 | 0.001 | 0.025 | 0.036 | 0.004 | 0.041 | 0.035 | 0.013 | 0.0004 | 368 | 1146 | 485 | 579 |
| 0.074 | 0.25 | 1.40 | 0.012 | 0.002 | 0.034 | 0.001 | 0.026 | 0.038 | 0.004 | 0.041 | 0.035 | 0.013 | 0.0005 | 372 | 1160 | 486 | 578 |
| 0.074 | 0.25 | 1.40 | 0.012 | 0.002 | 0.034 | 0.001 | 0.023 | 0.037 | 0.004 | 0.040 | 0.035 | 0.013 | 0.0004 | 375 | 1155 | 485 | 578 |
| 0.074 | 0.25 | 1.40 | 0.012 | 0.002 | 0.035 | 0.002 | 0.027 | 0.039 | 0.004 | 0.039 | 0.035 | 0.013 | 0.0004 | 376 | 1155 | 484 | 579 |
| 0.072 | 0.25 | 1.39 | 0.012 | 0.002 | 0.038 | 0.002 | 0.024 | 0.041 | 0.004 | 0.040 | 0.035 | 0.013 | 0.0005 | 374 | 1157 | 485 | 576 |
| 0.075 | 0.25 | 1.40 | 0.012 | 0.003 | 0.033 | 0.001 | 0.022 | 0.036 | 0.003 | 0.041 | 0.036 | 0.013 | 0.0005 | 372 | 1143 | 484 | 581 |
| 0.074 | 0.25 | 1.40 | 0.012 | 0.002 | 0.033 | 0.001 | 0.028 | 0.041 | 0.004 | 0.041 | 0.035 | 0.013 | 0.0005 | 374 | 1152 | 485 | 579 |
| 0.076 | 0.25 | 1.40 | 0.012 | 0.002 | 0.033 | 0.001 | 0.022 | 0.037 | 0.003 | 0.040 | 0.036 | 0.013 | 0.0005 | 371 | 1146 | 484 | 581 |
| 0.075 | 0.26 | 1.41 | 0.012 | 0.003 | 0.033 | 0.001 | 0.021 | 0.036 | 0.003 | 0.042 | 0.035 | 0.012 | 0.0005 | 380 | 1149 | 483 | 583 |
| 0.073 | 0.25 | 1.36 | 0.012 | 0.002 | 0.038 | 0.002 | 0.026 | 0.039 | 0.004 | 0.039 | 0.035 | 0.013 | 0.0006 | 361 | 1161 | 485 | 573 |
| 0.074 | 0.25 | 1.39 | 0.012 | 0.002 | 0.034 | 0.001 | 0.027 | 0.038 | 0.004 | 0.040 | 0.035 | 0.013 | 0.0005 | 369 | 1162 | 485 | 577 |
| 0.076 | 0.25 | 1.40 | 0.012 | 0.003 | 0.030 | 0.001 | 0.021 | 0.033 | 0.003 | 0.041 | 0.035 | 0.012 | 0.0005 | 372 | 1142 | 483 | 582 |
| 0.075 | 0.25 | 1.40 | 0.012 | 0.003 | 0.033 | 0.001 | 0.028 | 0.036 | 0.003 | 0.042 | 0.035 | 0.013 | 0.0005 | 370 | 1146 | 484 | 581 |
| 0.074 | 0.26 | 1.40 | 0.012 | 0.003 | 0.032 | 0.002 | 0.020 | 0.037 | 0.003 | 0.041 | 0.035 | 0.013 | 0.0005 | 369 | 1151 | 484 | 581 |
| 0.074 | 0.25 | 1.40 | 0.012 | 0.003 | 0.041 | 0.001 | 0.022 | 0.037 | 0.004 | 0.041 | 0.035 | 0.013 | 0.0005 | 373 | 1153 | 485 | 579 |
| 0.074 | 0.26 | 1.39 | 0.012 | 0.002 | 0.037 | 0.001 | 0.023 | 0.044 | 0.003 | 0.040 | 0.035 | 0.013 | 0.0006 | 367 | 1153 | 484 | 578 |
| 0.074 | 0.25 | 1.39 | 0.012 | 0.002 | 0.035 | 0.001 | 0.021 | 0.036 | 0.004 | 0.042 | 0.035 | 0.012 | 0.0005 | 378 | 1150 | 485 | 579 |
| 0.074 | 0.25 | 1.39 | 0.012 | 0.003 | 0.033 | 0.002 | 0.026 | 0.036 | 0.004 | 0.041 | 0.035 | 0.013 | 0.0005 | 369 | 1146 | 485 | 578 |
| 0.074 | 0.25 | 1.40 | 0.012 | 0.003 | 0.035 | 0.001 | 0.022 | 0.041 | 0.003 | 0.041 | 0.035 | 0.013 | 0.0006 | 366 | 1145 | 484 | 579 |
| 0.076 | 0.25 | 1.41 | 0.012 | 0.003 | 0.033 | 0.001 | 0.022 | 0.037 | 0.003 | 0.041 | 0.036 | 0.012 | 0.0005 | 371 | 1152 | 484 | 583 |
| 0.074 | 0.25 | 1.39 | 0.011 | 0.002 | 0.036 | 0.001 | 0.021 | 0.038 | 0.004 | 0.041 | 0.035 | 0.013 | 0.0005 | 377 | 1153 | 486 | 578 |
| 0.075 | 0.26 | 1.40 | 0.012 | 0.003 | 0.038 | 0.002 | 0.028 | 0.035 | 0.003 | 0.041 | 0.036 | 0.012 | 0.0005 | 361 | 1148 | 484 | 579 |
| 0.074 | 0.25 | 1.41 | 0.012 | 0.002 | 0.036 | 0.001 | 0.024 | 0.041 | 0.003 | 0.042 | 0.036 | 0.013 | 0.0005 | 373 | 1149 | 484 | 580 |
| 0.076 | 0.26 | 1.40 | 0.012 | 0.002 | 0.035 | 0.002 | 0.022 | 0.037 | 0.003 | 0.041 | 0.036 | 0.013 | 0.0005 | 372 | 1146 | 483 | 582 |
| 0.075 | 0.25 | 1.39 | 0.012 | 0.002 | 0.036 | 0.002 | 0.023 | 0.040 | 0.004 | 0.040 | 0.035 | 0.013 | 0.0005 | 374 | 1157 | 485 | 577 |
| 0.074 | 0.25 | 1.39 | 0.012 | 0.002 | 0.038 | 0.002 | 0.024 | 0.037 | 0.003 | 0.040 | 0.036 | 0.013 | 0.0005 | 370 | 1151 | 485 | 577 |
| 0.074 | 0.26 | 1.40 | 0.012 | 0.002 | 0.042 | 0.002 | 0.030 | 0.038 | 0.003 | 0.041 | 0.034 | 0.013 | 0.0004 | 372 | 1152 | 484 | 578 |
| 0.074 | 0.26 | 1.40 | 0.012 | 0.002 | 0.041 | 0.002 | 0.025 | 0.039 | 0.003 | 0.040 | 0.036 | 0.013 | 0.0005 | 373 | 1156 | 485 | 577 |
| 0.075 | 0.25 | 1.41 | 0.012 | 0.003 | 0.033 | 0.001 | 0.023 | 0.037 | 0.003 | 0.042 | 0.036 | 0.012 | 0.0005 | 378 | 1145 | 483 | 584 |
| 0.075 | 0.26 | 1.39 | 0.012 | 0.002 | 0.032 | 0.002 | 0.019 | 0.039 | 0.003 | 0.042 | 0.035 | 0.013 | 0.0004 | 370 | 1143 | 483 | 581 |
| 0.074 | 0.25 | 1.39 | 0.012 | 0.002 | 0.037 | 0.001 | 0.023 | 0.038 | 0.004 | 0.041 | 0.035 | 0.013 | 0.0005 | 376 | 1146 | 485 | 579 |
| 0.074 | 0.25 | 1.41 | 0.012 | 0.003 | 0.036 | 0.001 | 0.032 | 0.039 | 0.004 | 0.041 | 0.035 | 0.013 | 0.0005 | 372 | 1143 | 483 | 580 |
| 0.074 | 0.25 | 1.40 | 0.012 | 0.002 | 0.034 | 0.001 | 0.026 | 0.038 | 0.004 | 0.041 | 0.035 | 0.013 | 0.0005 | 375 | 1153 | 485 | 579 |
| 0.073 | 0.26 | 1.39 | 0.012 | 0.002 | 0.034 | 0.001 | 0.035 | 0.042 | 0.004 | 0.040 | 0.035 | 0.013 | 0.0007 | 363 | 1160 | 484 | 574 |
| 0.074 | 0.25 | 1.40 | 0.012 | 0.003 | 0.037 | 0.001 | 0.024 | 0.038 | 0.004 | 0.041 | 0.035 | 0.013 | 0.0005 | 373 | 1148 | 483 | 578 |
| 0.075 | 0.25 | 1.40 | 0.012 | 0.003 | 0.033 | 0.001 | 0.020 | 0.037 | 0.003 | 0.042 | 0.036 | 0.013 | 0.0005 | 378 | 1148 | 484 | 583 |
| 0.074 | 0.25 | 1.38 | 0.012 | 0.002 | 0.035 | 0.002 | 0.028 | 0.038 | 0.004 | 0.040 | 0.035 | 0.013 | 0.0005 | 372 | 1152 | 485 | 577 |
| 0.075 | 0.25 | 1.39 | 0.012 | 0.003 | 0.031 | 0.002 | 0.026 | 0.038 | 0.003 | 0.041 | 0.036 | 0.013 | 0.0006 | 379 | 1141 | 484 | 580 |
| 0.074 | 0.26 | 1.40 | 0.012 | 0.003 | 0.034 | 0.002 | 0.027 | 0.037 | 0.003 | 0.041 | 0.035 | 0.013 | 0.0005 | 364 | 1152 | 483 | 579 |
| 0.074 | 0.25 | 1.40 | 0.012 | 0.003 | 0.032 | 0.002 | 0.021 | 0.036 | 0.003 | 0.040 | 0.036 | 0.013 | 0.0005 | 370 | 1143 | 484 | 581 |
| 0.075 | 0.26 | 1.40 | 0.012 | 0.002 | 0.034 | 0.002 | 0.025 | 0.039 | 0.003 | 0.040 | 0.034 | 0.013 | 0.0005 | 373 | 1153 | 484 | 579 |
| 0.075 | 0.25 | 1.40 | 0.012 | 0.003 | 0.035 | 0.002 | 0.025 | 0.036 | 0.003 | 0.041 | 0.036 | 0.013 | 0.0004 | 368 | 1155 | 485 | 579 |
| 0.073 | 0.25 | 1.39 | 0.012 | 0.002 | 0.042 | 0.002 | 0.029 | 0.041 | 0.004 | 0.040 | 0.035 | 0.013 | 0.0005 | 367 | 1149 | 484 | 576 |
| 0.075 | 0.25 | 1.40 | 0.012 | 0.003 | 0.033 | 0.001 | 0.030 | 0.038 | 0.003 | 0.042 | 0.035 | 0.013 | 0.0005 | 373 | 1147 | 484 | 581 |
| 0.074 | 0.26 | 1.41 | 0.012 | 0.002 | 0.036 | 0.002 | 0.028 | 0.038 | 0.004 | 0.040 | 0.034 | 0.013 | 0.0006 | 366 | 1154 | 484 | 577 |
| 0.074 | 0.26 | 1.39 | 0.012 | 0.002 | 0.037 | 0.002 | 0.020 | 0.041 | 0.003 | 0.041 | 0.035 | 0.013 | 0.0005 | 361 | 1146 | 484 | 579 |
| 0.075 | 0.25 | 1.40 | 0.012 | 0.003 | 0.038 | 0.001 | 0.026 | 0.038 | 0.003 | 0.042 | 0.036 | 0.013 | 0.0005 | 357 | 1146 | 484 | 580 |
| 0.075 | 0.25 | 1.39 | 0.012 | 0.003 | 0.038 | 0.001 | 0.021 | 0.039 | 0.003 | 0.042 | 0.035 | 0.012 | 0.0004 | 374 | 1150 | 483 | 581 |
| 0.074 | 0.25 | 1.40 | 0.012 | 0.003 | 0.037 | 0.001 | 0.025 | 0.038 | 0.003 | 0.042 | 0.035 | 0.013 | 0.0005 | 373 | 1147 | 484 | 580 |
| 0.075 | 0.25 | 1.40 | 0.012 | 0.002 | 0.032 | 0.002 | 0.020 | 0.035 | 0.003 | 0.040 | 0.035 | 0.013 | 0.0005 | 377 | 1143 | 484 | 581 |
| 0.075 | 0.25 | 1.41 | 0.012 | 0.003 | 0.030 | 0.001 | 0.025 | 0.038 | 0.003 | 0.043 | 0.037 | 0.012 | 0.0004 | 366 | 1141 | 484 | 583 |
| 0.075 | 0.26 | 1.40 | 0.012 | 0.003 | 0.037 | 0.001 | 0.025 | 0.038 | 0.003 | 0.041 | 0.036 | 0.012 | 0.0005 | 381 | 1137 | 483 | 582 |
| 0.076 | 0.26 | 1.40 | 0.012 | 0.003 | 0.032 | 0.001 | 0.024 | 0.039 | 0.004 | 0.042 | 0.035 | 0.012 | 0.0004 | 376 | 1136 | 483 | 582 |
| 0.075 | 0.25 | 1.41 | 0.012 | 0.003 | 0.032 | 0.002 | 0.023 | 0.035 | 0.003 | 0.041 | 0.035 | 0.012 | 0.0004 | 375 | 1133 | 483 | 583 |
| 0.075 | 0.25 | 1.40 | 0.012 | 0.002 | 0.032 | 0.002 | 0.024 | 0.037 | 0.003 | 0.042 | 0.036 | 0.013 | 0.0005 | 377 | 1143 | 484 | 581 |
| 0.073 | 0.25 | 1.40 | 0.012 | 0.002 | 0.032 | 0.002 | 0.024 | 0.039 | 0.004 | 0.040 | 0.035 | 0.013 | 0.0004 | 368 | 1150 | 485 | 579 |
| 0.074 | 0.25 | 1.40 | 0.012 | 0.002 | 0.040 | 0.001 | 0.026 | 0.037 | 0.004 | 0.040 | 0.035 | 0.013 | 0.0005 | 370 | 1157 | 485 | 578 |
| 0.075 | 0.25 | 1.39 | 0.012 | 0.003 | 0.033 | 0.001 | 0.028 | 0.038 | 0.004 | 0.041 | 0.036 | 0.012 | 0.0005 | 383 | 1142 | 484 | 581 |
| 0.075 | 0.25 | 1.40 | 0.012 | 0.003 | 0.034 | 0.001 | 0.022 | 0.038 | 0.003 | 0.039 | 0.036 | 0.013 | 0.0005 | 371 | 1142 | 483 | 581 |

**Table S2**

| Ridge | | | | |
| --- | --- | --- | --- | --- |
| hyper parameter | Regression result | | | |
| alpha | MSE (training) | R^2^ (training) | MSE (validation) | R^2^ (validation) |
| 0.001 | 0.0105398 | 0.506191891 | 0.010621482 | 0.501938445 |
| 0.005 | 0.0105398 | 0.506191887 | 0.010621474 | 0.501938864 |
| 0.01 | 0.010539801 | 0.506191875 | 0.010621464 | 0.501939369 |
| 0.05 | 0.010539808 | 0.506191524 | 0.010621388 | 0.50194315 |
| 0.1 | 0.010539829 | 0.506190448 | 0.010621307 | 0.501947186 |
| **0.5** | **0.01054049** | **0.506159578** | **0.010621174** | **0.501955249** |
| 1 | 0.01054223 | 0.506078013 | 0.010622011 | 0.501918098 |

| Lasso | | | | |
| --- | --- | --- | --- | --- |
| hyper parameter | Regression result | | | |
| alpha | MSE (training) | R^2^ (training) | MSE (validation) | R^2^ (validation) |
| **0.00001** | **0.010540078** | **0.506178802** | **0.010621472** | **0.501940147** |
| 0.00005 | 0.01054681 | 0.505863391 | 0.010627074 | 0.501682221 |
| 0.0001 | 0.010566355 | 0.504947849 | 0.010644118 | 0.500886975 |
| 0.0005 | 0.010708498 | 0.498287703 | 0.010772037 | 0.494911898 |
| 0.001 | 0.01091288 | 0.488712302 | 0.010960182 | 0.486105309 |
| 0.005 | 0.012239908 | 0.426536005 | 0.012267238 | 0.424884955 |
| 0.01 | 0.013602754 | 0.362682066 | 0.013627623 | 0.361171088 |
| 0.05 | - | - | - | - |
| 0.1 | - | - | - | - |
| 0.5 | - | - | - | - |
| 1 | - | - | - | - |

| LARS | | | | |
| --- | --- | --- | --- | --- |
| hyper parameter | Regression result | | | |
| alpha | MSE (training) | R^2^ (training) | MSE (validation) | R^2^ (validation) |
| **0.0001** | **0.011165114** | **0.4768952** | **0.011204067** | **0.474682548** |
| 0.001 | 0.018527571 | 0.131939424 | 0.018543996 | 0.130612991 |
| 0.01 | - | - | - | - |
| 0.1 | - | - | - | - |
| 1 | - | - | - | - |

| ENR | | | | | |
| --- | --- | --- | --- | --- | --- |
| hyper parameter | | Regression result | | | |
| l1_ratio | alpha | MSE (training) | R^2^ (training) | MSE (validation) | R^2^ (validation) |
| **0.3** | **0.001** | **0.01066372** | **0.5003858** | **0.010727897** | **0.496974337** |
| 0.3 | 0.005 | 0.011186199 | 0.47590706 | 0.011225592 | 0.473671378 |
| 0.3 | 0.01 | 0.011756991 | 0.449163193 | 0.011782429 | 0.447585078 |
| 0.3 | 0.05 | 0.015866522 | 0.256619662 | 0.01588242 | 0.255463339 |
| 0.3 | 0.1 | 0.020378313 | 0.045239751 | 0.02038843 | 0.044065346 |
| 0.3 | 0.5 | - | - | - | - |
| 0.3 | 1 | - | - | - | - |
| 0.5 | 0.001 | 0.01072533 | 0.497499279 | 0.010786658 | 0.494228441 |
| 0.5 | 0.005 | 0.011543201 | 0.459180181 | 0.011572508 | 0.457412779 |
| 0.5 | 0.01 | 0.012312849 | 0.423118527 | 0.012338324 | 0.421553541 |
| 0.5 | 0.05 | 0.019009709 | 0.109352406 | 0.019024991 | 0.108090971 |
| 0.5 | 0.1 | - | - | - | - |
| 0.5 | 0.5 | - | - | - | - |
| 0.5 | 1 | - | - | - | - |
| 0.7 | 0.001 | 0.010798565 | 0.494067965 | 0.010853971 | 0.491079756 |
| 0.7 | 0.005 | 0.011810947 | 0.446634807 | 0.011838614 | 0.444953478 |
| 0.7 | 0.01 | 0.012841697 | 0.398341295 | 0.012866838 | 0.396796029 |
| 0.7 | 0.05 | 0.02107897 | 0.012409603 | 0.021090102 | 0.01114997 |
| 0.7 | 0.1 | - | - | - | - |
| 0.7 | 0.5 | - | - | - | - |
| 0.7 | 1 | - | - | - | - |

| KRR | | | | | | | | | |
| --- | --- | --- | --- | --- | --- | --- | --- | --- | --- |
| hyper parameter | | | | | | Regression result | | | |
| kernel | alpha | degree | coef0 | nu | length | MSE | R^2^ | MSE | R^2^ |
|  |  |  |  |  | _scale | (training) | (training) | (validation) | (validation) |
| matern | 0.001 | x | x | 0.5 | 1 | 9.44E-06 | 0.9995575 | 0.00721832 | 0.66151067 |
| matern | 0.001 | x | x | 0.5 | 10 | 0.000287 | 0.9865731 | 0.00687036 | 0.67782014 |
| matern | 0.001 | x | x | 0.5 | 100 | 0.001958 | 0.9082462 | 0.00618464 | 0.70996728 |
| matern | 0.001 | x | x | 1.5 | 1 | 0.001863 | 0.9127309 | 0.00777948 | 0.63520576 |
| matern | 0.001 | x | x | 1.5 | 10 | 0.005996 | 0.7190916 | 0.00691577 | 0.67570669 |
| matern | 0.001 | x | x | 1.5 | 100 | 0.010047 | 0.5292647 | 0.01014656 | 0.52423342 |
| matern | 0.001 | x | x | 2.5 | 1 | 0.003197 | 0.8502005 | 0.00720353 | 0.66211909 |
| matern | 0.001 | x | x | 2.5 | 10 | 0.007746 | 0.6370721 | 0.00809442 | 0.62042822 |
| matern | 0.001 | x | x | 2.5 | 100 | 0.010559 | 0.505292 | 0.01063305 | 0.50141371 |
| matern | 0.01 | x | x | 0.5 | 1 | 0.000287 | 0.9865577 | 0.00685206 | 0.67867648 |
| matern | 0.01 | x | x | 0.5 | 10 | 0.001958 | 0.9082442 | 0.00618386 | 0.71000373 |
| matern | 0.01 | x | x | 0.5 | 100 | 0.004752 | 0.7773633 | 0.00645542 | 0.69730606 |
| matern | 0.01 | x | x | 1.5 | 1 | 0.00316 | 0.8519541 | 0.00650237 | 0.69504022 |
| matern | 0.01 | x | x | 1.5 | 10 | 0.007468 | 0.6501242 | 0.00787825 | 0.63058994 |
| matern | 0.01 | x | x | 1.5 | 100 | 0.010668 | 0.5001971 | 0.0107294 | 0.49692373 |
| matern | 0.01 | x | x | 2.5 | 1 | 0.00422 | 0.8022901 | 0.0065007 | 0.69512869 |
| matern | 0.01 | x | x | 2.5 | 10 | 0.008776 | 0.5888245 | 0.00896353 | 0.5797084 |
| matern | 0.01 | x | x | 2.5 | 100 | 0.010868 | 0.4908162 | 0.0109199 | 0.48800272 |
| **matern** | **0.1** | **x** | **x** | **0.5** | **1** | **0.00196** | **0.90795** | **0.0061725** | **0.710536** |
| matern | 0.1 | x | x | 0.5 | 10 | 0.004753 | 0.7773195 | 0.00645584 | 0.6972865 |
| matern | 0.1 | x | x | 0.5 | 100 | 0.007534 | 0.6470313 | 0.00805241 | 0.62246192 |
| matern | 0.1 | x | x | 1.5 | 1 | 0.004664 | 0.7814852 | 0.00636987 | 0.70129071 |
| matern | 0.1 | x | x | 1.5 | 10 | 0.008943 | 0.5809869 | 0.00912021 | 0.57237715 |
| matern | 0.1 | x | x | 1.5 | 100 | 0.011775 | 0.4483074 | 0.01180736 | 0.44646323 |
| matern | 0.1 | x | x | 2.5 | 1 | 0.005435 | 0.7453798 | 0.00666246 | 0.68757824 |
| matern | 0.1 | x | x | 2.5 | 10 | 0.00993 | 0.534772 | 0.0100311 | 0.5296562 |
| matern | 0.1 | x | x | 2.5 | 100 | 0.012437 | 0.417326 | 0.01246298 | 0.41575534 |
| matern | 1 | x | x | 0.5 | 1 | 0.004814 | 0.7744589 | 0.00650775 | 0.69486375 |
| matern | 1 | x | x | 0.5 | 10 | 0.007549 | 0.6463091 | 0.00806775 | 0.62174566 |
| matern | 1 | x | x | 0.5 | 100 | 0.010195 | 0.5223256 | 0.01032886 | 0.51576301 |
| matern | 1 | x | x | 1.5 | 1 | 0.006385 | 0.7008402 | 0.00714217 | 0.66511739 |
| matern | 1 | x | x | 1.5 | 10 | 0.010335 | 0.515807 | 0.01041208 | 0.51181667 |
| matern | 1 | x | x | 1.5 | 100 | 0.016179 | 0.2419673 | 0.01619473 | 0.24080456 |
| matern | 1 | x | x | 2.5 | 1 | 0.006893 | 0.6770665 | 0.00747563 | 0.64947918 |
| matern | 1 | x | x | 2.5 | 10 | 0.010768 | 0.4955077 | 0.01082384 | 0.49251149 |
| matern | 1 | x | x | 2.5 | 100 | 0.017586 | 0.1760547 | 0.01759928 | 0.17491701 |
| linear | 0.001 | x | x | x | x | 0.011102 | 0.4798278 | 0.0111986 | 0.47486578 |
| linear | 0.01 | x | x | x | x | 0.010853 | 0.4915162 | 0.0109335 | 0.48729357 |
| linear | 0.1 | x | x | x | x | 0.010849 | 0.4917007 | 0.01092995 | 0.48746136 |
| linear | 1 | x | x | x | x | 0.010851 | 0.4916004 | 0.01093044 | 0.48744207 |
| poly | 0.001 | 2 | 1 | x | x | 0.007595 | 0.6441734 | 0.00803019 | 0.62337727 |
| poly | 0.001 | 3 | 1 | x | x | 0.006786 | 0.6820853 | 0.00746293 | 0.65000578 |
| poly | 0.001 | 4 | 1 | x | x | 0.006349 | 0.7025553 | 0.00724128 | 0.66039737 |
| poly | 0.01 | 2 | 1 | x | x | 0.007962 | 0.6269515 | 0.00827738 | 0.61182768 |
| poly | 0.01 | 3 | 1 | x | x | 0.007437 | 0.6515401 | 0.00787068 | 0.63089437 |
| poly | 0.01 | 4 | 1 | x | x | 0.007009 | 0.6716151 | 0.00756884 | 0.64504899 |
| poly | 0.1 | 2 | 1 | x | x | 0.008762 | 0.5895028 | 0.00894997 | 0.58033564 |
| poly | 0.1 | 3 | 1 | x | x | 0.008762 | 0.5895028 | 0.00894997 | 0.58033564 |
| poly | 0.1 | 4 | 1 | x | x | 0.007798 | 0.6346335 | 0.00813353 | 0.61858948 |
| poly | 1 | 2 | 1 | x | x | 0.009832 | 0.539345 | 0.00993637 | 0.53409852 |
| poly | 1 | 3 | 1 | x | x | 0.009219 | 0.5680841 | 0.00936309 | 0.56097832 |
| poly | 1 | 4 | 1 | x | x | 0.008742 | 0.5904242 | 0.00892673 | 0.58143148 |
| rbf | 0.001 | x | x | x | x | 0.006728 | 0.6847697 | 0.00740136 | 0.65290094 |
| rbf | 0.01 | x | x | x | x | 0.007537 | 0.64686 | 0.00794053 | 0.62763083 |
| rbf | 0.1 | x | x | x | x | 0.008441 | 0.6045108 | 0.00866788 | 0.59356173 |
| rbf | 1 | x | x | x | x | 0.009652 | 0.5477893 | 0.00977015 | 0.54190451 |
| sigmoid | 0.001 | x | 1 | x | x | 0.008624 | 0.5959629 | 0.00912046 | 0.5723115 |
| sigmoid | 0.01 | x | 1 | x | x | 0.018706 | 0.1234618 | 0.01926842 | 0.0962237 |
| sigmoid | 0.1 | x | 1 | x | x | 0.019862 | 0.0693521 | 0.01993236 | 0.06505429 |
| sigmoid | 1 | x | 1 | x | x | 0.012614 | 0.4089973 | 0.01263001 | 0.40772992 |

| BRR | | | | |
| --- | --- | --- | --- | --- |
| hyper parameter | Regression result | | | |
| tol | MSE (training) | R^2^ (training) | MSE (validation) | R^2^ (validation) |
| 0.00001 | 0.010542411 | 0.506069654 | 0.010622457 | 0.50189721 |
| 0.0001 | 0.010542411 | 0.506069655 | 0.010622457 | 0.501897212 |
| 0.001 | 0.010542411 | 0.506069679 | 0.010622457 | 0.501897227 |
| 0.01 | 0.010542405 | 0.506069945 | 0.010622454 | 0.501897375 |
| **0.1** | **0.010542356** | **0.506072247** | **0.010622424** | **0.501898755** |

| ARD | | | | | | | |
| --- | --- | --- | --- | --- | --- | --- | --- |
| hyper parameter | | | | Regression result | | | |
| alpha_1 | alpha_2 | lambda_1 | lambda_2 | MSE  (training) | R^2^  (training) | MSE  (validation) | R^2^  (validation) |
| 1.00E-04 | 1.00E-04 | 1.00E-04 | 1.00E-04 | 0.010542 | 0.506094 | 0.0106239 | 0.5018275 |
| 1.00E-04 | 1.00E-04 | 1.00E-04 | 1.00E-06 | 0.010551 | 0.505665 | 0.0106356 | 0.501277 |
| 1.00E-04 | 1.00E-04 | 1.00E-04 | 1.00E-08 | 0.010551 | 0.505661 | 0.0106357 | 0.5012728 |
| 1.00E-04 | 1.00E-04 | 1.00E-06 | 1.00E-04 | 0.010542 | 0.506094 | 0.0106239 | 0.5018275 |
| 1.00E-04 | 1.00E-04 | 1.00E-06 | 1.00E-06 | 0.010551 | 0.505665 | 0.0106356 | 0.5012771 |
| 1.00E-04 | 1.00E-04 | 1.00E-06 | 1.00E-08 | 0.010551 | 0.505661 | 0.0106357 | 0.5012729 |
| 1.00E-04 | 1.00E-04 | 1.00E-08 | 1.00E-04 | 0.010542 | 0.506094 | 0.0106239 | 0.5018275 |
| 1.00E-04 | 1.00E-04 | 1.00E-08 | 1.00E-06 | 0.010551 | 0.505665 | 0.0106356 | 0.5012771 |
| 1.00E-04 | 1.00E-04 | 1.00E-08 | 1.00E-08 | 0.010551 | 0.505661 | 0.0106357 | 0.5012729 |
| 1.00E-04 | 1.00E-06 | 1.00E-04 | 1.00E-04 | 0.010542 | 0.506094 | 0.0106239 | 0.5018275 |
| 1.00E-04 | 1.00E-06 | 1.00E-04 | 1.00E-06 | 0.010551 | 0.505665 | 0.0106356 | 0.501277 |
| 1.00E-04 | 1.00E-06 | 1.00E-04 | 1.00E-08 | 0.010551 | 0.505661 | 0.0106357 | 0.5012728 |
| 1.00E-04 | 1.00E-06 | 1.00E-06 | 1.00E-04 | 0.010542 | 0.506094 | 0.0106239 | 0.5018275 |
| 1.00E-04 | 1.00E-06 | 1.00E-06 | 1.00E-06 | 0.010551 | 0.505665 | 0.0106356 | 0.5012771 |
| 1.00E-04 | 1.00E-06 | 1.00E-06 | 1.00E-08 | 0.010551 | 0.505661 | 0.0106357 | 0.5012729 |
| 1.00E-04 | 1.00E-06 | 1.00E-08 | 1.00E-04 | 0.010542 | 0.506094 | 0.0106239 | 0.5018275 |
| 1.00E-04 | 1.00E-06 | 1.00E-08 | 1.00E-06 | 0.010551 | 0.505665 | 0.0106356 | 0.5012771 |
| 1.00E-04 | 1.00E-06 | 1.00E-08 | 1.00E-08 | 0.010551 | 0.505661 | 0.0106357 | 0.5012729 |
| 1.00E-04 | 1.00E-08 | 1.00E-04 | 1.00E-04 | 0.010542 | 0.506094 | 0.0106239 | 0.5018275 |
| 1.00E-04 | 1.00E-08 | 1.00E-04 | 1.00E-06 | 0.010551 | 0.505665 | 0.0106356 | 0.501277 |
| 1.00E-04 | 1.00E-08 | 1.00E-04 | 1.00E-08 | 0.010551 | 0.505661 | 0.0106357 | 0.5012728 |
| 1.00E-04 | 1.00E-08 | 1.00E-06 | 1.00E-04 | 0.010542 | 0.506094 | 0.0106239 | 0.5018275 |
| 1.00E-04 | 1.00E-08 | 1.00E-06 | 1.00E-06 | 0.010551 | 0.505665 | 0.0106356 | 0.5012771 |
| 1.00E-04 | 1.00E-08 | 1.00E-06 | 1.00E-08 | 0.010551 | 0.505661 | 0.0106357 | 0.5012729 |
| **1.00E-04** | **1.00E-08** | **1.00E-08** | **1.00E-04** | **0.01054** | **0.50609** | **0.010624** | **0.501828** |
| 1.00E-04 | 1.00E-08 | 1.00E-08 | 1.00E-06 | 0.010551 | 0.505665 | 0.0106356 | 0.5012771 |
| 1.00E-04 | 1.00E-08 | 1.00E-08 | 1.00E-08 | 0.010551 | 0.505661 | 0.0106357 | 0.5012729 |
| 1.00E-06 | 1.00E-04 | 1.00E-04 | 1.00E-04 | 0.010542 | 0.506094 | 0.0106239 | 0.5018275 |
| 1.00E-06 | 1.00E-04 | 1.00E-04 | 1.00E-06 | 0.010551 | 0.505665 | 0.0106356 | 0.501277 |
| 1.00E-06 | 1.00E-04 | 1.00E-04 | 1.00E-08 | 0.010551 | 0.505661 | 0.0106357 | 0.5012728 |
| 1.00E-06 | 1.00E-04 | 1.00E-06 | 1.00E-04 | 0.010542 | 0.506094 | 0.0106239 | 0.5018275 |
| 1.00E-06 | 1.00E-04 | 1.00E-06 | 1.00E-06 | 0.010551 | 0.505665 | 0.0106356 | 0.5012771 |
| 1.00E-06 | 1.00E-04 | 1.00E-06 | 1.00E-08 | 0.010551 | 0.505661 | 0.0106357 | 0.5012729 |
| 1.00E-06 | 1.00E-04 | 1.00E-08 | 1.00E-04 | 0.010542 | 0.506094 | 0.0106239 | 0.5018275 |
| 1.00E-06 | 1.00E-04 | 1.00E-08 | 1.00E-06 | 0.010551 | 0.505665 | 0.0106356 | 0.5012771 |
| 1.00E-06 | 1.00E-04 | 1.00E-08 | 1.00E-08 | 0.010551 | 0.505661 | 0.0106357 | 0.5012729 |
| 1.00E-06 | 1.00E-06 | 1.00E-04 | 1.00E-04 | 0.010542 | 0.506094 | 0.0106239 | 0.5018275 |
| 1.00E-06 | 1.00E-06 | 1.00E-04 | 1.00E-06 | 0.010551 | 0.505665 | 0.0106356 | 0.501277 |
| 1.00E-06 | 1.00E-06 | 1.00E-04 | 1.00E-08 | 0.010551 | 0.505661 | 0.0106357 | 0.5012728 |
| 1.00E-06 | 1.00E-06 | 1.00E-06 | 1.00E-04 | 0.010542 | 0.506094 | 0.0106239 | 0.5018275 |
| 1.00E-06 | 1.00E-06 | 1.00E-06 | 1.00E-06 | 0.010551 | 0.505665 | 0.0106356 | 0.5012771 |
| 1.00E-06 | 1.00E-06 | 1.00E-06 | 1.00E-08 | 0.010551 | 0.505661 | 0.0106357 | 0.5012729 |
| 1.00E-06 | 1.00E-06 | 1.00E-08 | 1.00E-04 | 0.010542 | 0.506094 | 0.0106239 | 0.5018275 |
| 1.00E-06 | 1.00E-06 | 1.00E-08 | 1.00E-06 | 0.010551 | 0.505665 | 0.0106356 | 0.5012771 |
| 1.00E-06 | 1.00E-06 | 1.00E-08 | 1.00E-08 | 0.010551 | 0.505661 | 0.0106357 | 0.5012729 |
| 1.00E-06 | 1.00E-08 | 1.00E-04 | 1.00E-04 | 0.010542 | 0.506094 | 0.0106239 | 0.5018275 |
| 1.00E-06 | 1.00E-08 | 1.00E-04 | 1.00E-06 | 0.010551 | 0.505665 | 0.0106356 | 0.501277 |
| 1.00E-06 | 1.00E-08 | 1.00E-04 | 1.00E-08 | 0.010551 | 0.505661 | 0.0106357 | 0.5012728 |
| 1.00E-06 | 1.00E-08 | 1.00E-06 | 1.00E-04 | 0.010542 | 0.506094 | 0.0106239 | 0.5018275 |
| 1.00E-06 | 1.00E-08 | 1.00E-06 | 1.00E-06 | 0.010551 | 0.505665 | 0.0106356 | 0.5012771 |
| 1.00E-06 | 1.00E-08 | 1.00E-06 | 1.00E-08 | 0.010551 | 0.505661 | 0.0106357 | 0.5012729 |
| 1.00E-06 | 1.00E-08 | 1.00E-08 | 1.00E-04 | 0.010542 | 0.506094 | 0.0106239 | 0.5018275 |
| 1.00E-06 | 1.00E-08 | 1.00E-08 | 1.00E-06 | 0.010551 | 0.505665 | 0.0106356 | 0.5012771 |
| 1.00E-06 | 1.00E-08 | 1.00E-08 | 1.00E-08 | 0.010551 | 0.505661 | 0.0106357 | 0.5012729 |
| 1.00E-08 | 1.00E-04 | 1.00E-04 | 1.00E-04 | 0.010542 | 0.506094 | 0.0106239 | 0.5018275 |
| 1.00E-08 | 1.00E-04 | 1.00E-04 | 1.00E-06 | 0.010551 | 0.505665 | 0.0106356 | 0.501277 |
| 1.00E-08 | 1.00E-04 | 1.00E-04 | 1.00E-08 | 0.010551 | 0.505661 | 0.0106357 | 0.5012728 |
| 1.00E-08 | 1.00E-04 | 1.00E-06 | 1.00E-04 | 0.010542 | 0.506094 | 0.0106239 | 0.5018275 |
| 1.00E-08 | 1.00E-04 | 1.00E-06 | 1.00E-06 | 0.010551 | 0.505665 | 0.0106356 | 0.5012771 |
| 1.00E-08 | 1.00E-04 | 1.00E-06 | 1.00E-08 | 0.010551 | 0.505661 | 0.0106357 | 0.5012729 |
| 1.00E-08 | 1.00E-04 | 1.00E-08 | 1.00E-04 | 0.010542 | 0.506094 | 0.0106239 | 0.5018275 |
| 1.00E-08 | 1.00E-04 | 1.00E-08 | 1.00E-06 | 0.010551 | 0.505665 | 0.0106356 | 0.5012771 |
| 1.00E-08 | 1.00E-04 | 1.00E-08 | 1.00E-08 | 0.010551 | 0.505661 | 0.0106357 | 0.5012729 |
| 1.00E-08 | 1.00E-06 | 1.00E-04 | 1.00E-04 | 0.010542 | 0.506094 | 0.0106239 | 0.5018275 |
| 1.00E-08 | 1.00E-06 | 1.00E-04 | 1.00E-06 | 0.010551 | 0.505665 | 0.0106356 | 0.501277 |
| 1.00E-08 | 1.00E-06 | 1.00E-04 | 1.00E-08 | 0.010551 | 0.505661 | 0.0106357 | 0.5012728 |
| 1.00E-08 | 1.00E-06 | 1.00E-06 | 1.00E-04 | 0.010542 | 0.506094 | 0.0106239 | 0.5018275 |
| 1.00E-08 | 1.00E-06 | 1.00E-06 | 1.00E-06 | 0.010551 | 0.505665 | 0.0106356 | 0.5012771 |
| 1.00E-08 | 1.00E-06 | 1.00E-06 | 1.00E-08 | 0.010551 | 0.505661 | 0.0106357 | 0.5012729 |
| 1.00E-08 | 1.00E-06 | 1.00E-08 | 1.00E-04 | 0.010542 | 0.506094 | 0.0106239 | 0.5018275 |
| 1.00E-08 | 1.00E-06 | 1.00E-08 | 1.00E-06 | 0.010551 | 0.505665 | 0.0106356 | 0.5012771 |
| 1.00E-08 | 1.00E-06 | 1.00E-08 | 1.00E-08 | 0.010551 | 0.505661 | 0.0106357 | 0.5012729 |
| 1.00E-08 | 1.00E-08 | 1.00E-04 | 1.00E-04 | 0.010542 | 0.506094 | 0.0106239 | 0.5018275 |
| 1.00E-08 | 1.00E-08 | 1.00E-04 | 1.00E-06 | 0.010551 | 0.505665 | 0.0106356 | 0.501277 |
| 1.00E-08 | 1.00E-08 | 1.00E-04 | 1.00E-08 | 0.010551 | 0.505661 | 0.0106357 | 0.5012728 |
| 1.00E-08 | 1.00E-08 | 1.00E-06 | 1.00E-04 | 0.010542 | 0.506094 | 0.0106239 | 0.5018275 |
| 1.00E-08 | 1.00E-08 | 1.00E-06 | 1.00E-06 | 0.010551 | 0.505665 | 0.0106356 | 0.5012771 |
| 1.00E-08 | 1.00E-08 | 1.00E-06 | 1.00E-08 | 0.010551 | 0.505661 | 0.0106357 | 0.5012729 |
| 1.00E-08 | 1.00E-08 | 1.00E-08 | 1.00E-04 | 0.010542 | 0.506094 | 0.0106239 | 0.5018275 |
| 1.00E-08 | 1.00E-08 | 1.00E-08 | 1.00E-06 | 0.010551 | 0.505665 | 0.0106356 | 0.5012771 |
| 1.00E-08 | 1.00E-08 | 1.00E-08 | 1.00E-08 | 0.010551 | 0.505661 | 0.0106357 | 0.5012729 |

| RF | | | | | | |
| --- | --- | --- | --- | --- | --- | --- |
| hyper parameter | | | Regression result | | | |
| max_features | n_estimators | max_depth | MSE | R^2^ | MSE | R^2^ |
|  |  |  | (training) | (training) | (validation) | (validation) |
| auto | 50 | 5 | 0.00814 | 0.61861857 | 0.008674336 | 0.59331883 |
| auto | 50 | 10 | 0.003582 | 0.83216837 | 0.00637197 | 0.70126131 |
| auto | 50 | 15 | 0.0014136 | 0.93376608 | 0.006061889 | 0.71573841 |
| auto | 100 | 5 | 0.0081197 | 0.6195704 | 0.008654815 | 0.59422296 |
| auto | 100 | 10 | 0.0035525 | 0.8335497 | 0.006338265 | 0.70284766 |
| auto | 100 | 15 | 0.0013805 | 0.93531445 | 0.006014241 | 0.71798222 |
| auto | 150 | 5 | 0.0081111 | 0.61997469 | 0.008650215 | 0.59443241 |
| auto | 150 | 10 | 0.0035416 | 0.8340606 | 0.006327492 | 0.70334912 |
| auto | 150 | 15 | 0.0013656 | 0.93601326 | 0.006000872 | 0.71861257 |
| log2 | 50 | 5 | 0.0082686 | 0.61259498 | 0.00868208 | 0.59295025 |
| log2 | 50 | 10 | 0.0039388 | 0.81545455 | 0.006253112 | 0.70678238 |
| log2 | 50 | 15 | 0.0014691 | 0.9311684 | 0.00597113 | 0.71995056 |
| log2 | 100 | 5 | 0.0082396 | 0.61395829 | 0.008646107 | 0.59461691 |
| log2 | 100 | 10 | 0.0039118 | 0.81671971 | 0.006220008 | 0.70834649 |
| log2 | 100 | 15 | 0.0014355 | 0.93274163 | 0.005923921 | 0.72219698 |
| log2 | 150 | 5 | 0.0082316 | 0.61433167 | 0.0086381 | 0.594989 |
| log2 | 150 | 10 | 0.0039041 | 0.8170799 | 0.006212636 | 0.70869306 |
| log2 | 150 | 15 | 0.0014188 | 0.93352538 | 0.005911763 | 0.7227668 |
| sqrt | 50 | 5 | 0.0082686 | 0.61259498 | 0.00868208 | 0.59295025 |
| sqrt | 50 | 10 | 0.0039388 | 0.81545455 | 0.006253112 | 0.70678238 |
| sqrt | 50 | 15 | 0.0014691 | 0.9311684 | 0.00597113 | 0.71995056 |
| sqrt | 100 | 5 | 0.0082396 | 0.61395829 | 0.008646107 | 0.59461691 |
| sqrt | 100 | 10 | 0.0039118 | 0.81671971 | 0.006220008 | 0.70834649 |
| sqrt | 100 | 15 | 0.0014355 | 0.93274163 | 0.005923921 | 0.72219698 |
| sqrt | 150 | 5 | 0.0082316 | 0.61433167 | 0.0086381 | 0.594989 |
| sqrt | 150 | 10 | 0.0039041 | 0.8170799 | 0.006212636 | 0.70869306 |
| **sqrt** | **150** | **15** | **0.001419** | **0.9335254** | **0.0059118** | **0.722767** |

| Ada Boost | | | | | | |
| --- | --- | --- | --- | --- | --- | --- |
| hyper parameter | | | Regression result | | | |
| loss | learning_rate | n_estimators | MSE (training) | R^2^ (training) | MSE (validation) | R^2^ (validation) |
| exponential | 0.001 | 50 | 0.0103958 | 0.5129404 | 0.010604587 | 0.502718919 |
| exponential | 0.001 | 100 | 0.0103823 | 0.5135735 | 0.010587939 | 0.503483937 |
| exponential | 0.001 | 150 | 0.0103767 | 0.5138343 | 0.010581744 | 0.503774545 |
| exponential | 0.01 | 50 | 0.0103523 | 0.5149793 | 0.01055242 | 0.505140883 |
| exponential | 0.01 | 100 | 0.0102929 | 0.5177603 | 0.010491333 | 0.508009136 |
| exponential | 0.01 | 150 | 0.0102025 | 0.5219904 | 0.010399558 | 0.512326857 |
| exponential | 0.1 | 50 | 0.0096824 | 0.5463607 | 0.009898309 | 0.535811652 |
| **exponential** | **0.1** | **100** | **0.009603** | **0.55009** | **0.0098722** | **0.536968** |
| exponential | 0.1 | 150 | 0.0096703 | 0.5469275 | 0.009977401 | 0.531960397 |
| exponential | 1 | 50 | 0.0108574 | 0.4913164 | 0.011257949 | 0.471748846 |
| exponential | 1 | 100 | 0.0124463 | 0.4169187 | 0.012870767 | 0.395769306 |
| exponential | 1 | 150 | 0.0133855 | 0.3729272 | 0.013803636 | 0.351899525 |
| linear | 0.001 | 50 | 0.0103938 | 0.5130351 | 0.010605964 | 0.502645547 |
| linear | 0.001 | 100 | 0.0103822 | 0.5135764 | 0.010592529 | 0.503265556 |
| linear | 0.001 | 150 | 0.0103798 | 0.5136893 | 0.010587792 | 0.503487529 |
| linear | 0.01 | 50 | 0.0103569 | 0.5147601 | 0.010557466 | 0.504910125 |
| linear | 0.01 | 100 | 0.0102596 | 0.5193227 | 0.010457384 | 0.509600284 |
| linear | 0.01 | 150 | 0.0101299 | 0.525392 | 0.010324309 | 0.515856245 |
| linear | 0.1 | 50 | 0.009705 | 0.5452994 | 0.009934771 | 0.534088334 |
| linear | 0.1 | 100 | 0.0097165 | 0.544763 | 0.009993929 | 0.531229069 |
| linear | 0.1 | 150 | 0.0097857 | 0.5415209 | 0.010085044 | 0.526893894 |
| linear | 1 | 50 | 0.0101499 | 0.5244745 | 0.010523299 | 0.506218267 |
| linear | 1 | 100 | 0.0102771 | 0.5185196 | 0.010657014 | 0.499907945 |
| linear | 1 | 150 | 0.0103253 | 0.5162643 | 0.010708038 | 0.497487315 |
| square | 0.001 | 50 | 0.0103939 | 0.5130287 | 0.010601514 | 0.502850543 |
| square | 0.001 | 100 | 0.0103895 | 0.5132385 | 0.010591855 | 0.503299957 |
| square | 0.001 | 150 | 0.0103812 | 0.513625 | 0.010585659 | 0.503583671 |
| square | 0.01 | 50 | 0.0103373 | 0.5156802 | 0.010539112 | 0.505756878 |
| square | 0.01 | 100 | 0.0101795 | 0.523069 | 0.0103782 | 0.513310662 |
| square | 0.01 | 150 | 0.0099615 | 0.5332814 | 0.010157965 | 0.523651154 |
| square | 0.1 | 50 | 0.0099448 | 0.534063 | 0.010204033 | 0.521367787 |
| square | 0.1 | 100 | 0.0101216 | 0.5257847 | 0.010446465 | 0.509879849 |
| square | 0.1 | 150 | 0.0103832 | 0.5135371 | 0.010742645 | 0.495894853 |
| square | 1 | 50 | 0.0121419 | 0.4311891 | 0.012568705 | 0.409917215 |
| square | 1 | 100 | 0.0126466 | 0.4075425 | 0.013084452 | 0.385719112 |
| square | 1 | 150 | 0.0128501 | 0.3980052 | 0.013302022 | 0.375544186 |

| Gradient Boost | | | | | | | |
| --- | --- | --- | --- | --- | --- | --- | --- |
| hyper parameter | | | | Regression result | | | |
| loss | learning | n_estimators | sub | MSE | R^2^ | MSE | R^2^ |
|  | _rate |  | sample | (training) | (training) | (validation) | (validation) |
| huber | 0.001 | 50 | 0.4 | 0.020401 | 0.04417 | 0.02041823 | 0.04267848 |
| huber | 0.001 | 50 | 0.7 | 0.0204 | 0.04423 | 0.02041771 | 0.04270259 |
| huber | 0.001 | 50 | 1 | 0.020398 | 0.04433 | 0.02041674 | 0.0427479 |
| huber | 0.001 | 100 | 0.4 | 0.019483 | 0.0872 | 0.01950862 | 0.08535816 |
| huber | 0.001 | 100 | 0.7 | 0.019483 | 0.08721 | 0.01950974 | 0.08530457 |
| huber | 0.001 | 100 | 1 | 0.019479 | 0.08739 | 0.01950892 | 0.08534221 |
| huber | 0.001 | 150 | 0.4 | 0.018648 | 0.12632 | 0.01868207 | 0.12413789 |
| huber | 0.001 | 150 | 0.7 | 0.018649 | 0.12628 | 0.01868536 | 0.12398405 |
| huber | 0.001 | 150 | 1 | 0.018647 | 0.12638 | 0.01868696 | 0.12390672 |
| huber | 0.01 | 50 | 0.4 | 0.014515 | 0.31994 | 0.01460379 | 0.31545282 |
| huber | 0.01 | 50 | 0.7 | 0.014536 | 0.31896 | 0.01463008 | 0.31422158 |
| huber | 0.01 | 50 | 1 | 0.014553 | 0.31817 | 0.01465879 | 0.31288886 |
| huber | 0.01 | 100 | 0.4 | 0.011592 | 0.45688 | 0.01175019 | 0.44926214 |
| huber | 0.01 | 100 | 0.7 | 0.011636 | 0.45484 | 0.01180343 | 0.44677031 |
| huber | 0.01 | 100 | 1 | 0.011651 | 0.45411 | 0.01183113 | 0.44546765 |
| huber | 0.01 | 150 | 0.4 | 0.010137 | 0.52508 | 0.0103542 | 0.51468107 |
| huber | 0.01 | 150 | 0.7 | 0.01019 | 0.52259 | 0.01041702 | 0.51173178 |
| huber | 0.01 | 150 | 1 | 0.010223 | 0.52105 | 0.01046727 | 0.50937368 |
| huber | 0.1 | 50 | 0.4 | 0.00746 | 0.65051 | 0.00798994 | 0.62538962 |
| huber | 0.1 | 50 | 0.7 | 0.007479 | 0.64958 | 0.00802316 | 0.62385033 |
| huber | 0.1 | 50 | 1 | 0.007545 | 0.64649 | 0.00811919 | 0.61935685 |
| huber | 0.1 | 100 | 0.4 | 0.006432 | 0.69863 | 0.00726672 | 0.6592822 |
| huber | 0.1 | 100 | 0.7 | 0.006425 | 0.69898 | 0.00726759 | 0.65923508 |
| huber | 0.1 | 100 | 1 | 0.006533 | 0.69391 | 0.0073949 | 0.6533055 |
| huber | 0.1 | 150 | 0.4 | 0.005889 | 0.72407 | 0.00694723 | 0.67426198 |
| huber | 0.1 | 150 | 0.7 | 0.005849 | 0.72598 | 0.00692075 | 0.67548806 |
| huber | 0.1 | 150 | 1 | 0.005975 | 0.72007 | 0.00704874 | 0.66952572 |
| huber | 1 | 50 | 0.4 | 0.006542 | 0.69352 | 0.00914846 | 0.57080779 |
| huber | 1 | 50 | 0.7 | 0.005342 | 0.74972 | 0.0078507 | 0.63179483 |
| huber | 1 | 50 | 1 | 0.004905 | 0.7702 | 0.00747855 | 0.64930686 |
| huber | 1 | 100 | 0.4 | 0.006154 | 0.71168 | 0.01028348 | 0.51759642 |
| huber | 1 | 100 | 0.7 | 0.00446 | 0.79105 | 0.00817563 | 0.61654959 |
| huber | 1 | 100 | 1 | 0.003872 | 0.81859 | 0.00762791 | 0.64220441 |
| huber | 1 | 150 | 0.4 | 0.005933 | 0.72201 | 0.01130204 | 0.46986968 |
| huber | 1 | 150 | 0.7 | 0.003918 | 0.81645 | 0.00839941 | 0.60603952 |
| huber | 1 | 150 | 1 | 0.003262 | 0.84717 | 0.00779662 | 0.63435819 |
| lad | 0.001 | 50 | 0.4 | 0.020512 | 0.03897 | 0.02052695 | 0.03757949 |
| lad | 0.001 | 50 | 0.7 | 0.020495 | 0.03978 | 0.02050939 | 0.03840363 |
| lad | 0.001 | 50 | 1 | 0.020481 | 0.04045 | 0.02049376 | 0.03913515 |
| lad | 0.001 | 100 | 0.4 | 0.01969 | 0.07747 | 0.01971192 | 0.07582329 |
| lad | 0.001 | 100 | 0.7 | 0.019661 | 0.07884 | 0.01968236 | 0.0772105 |
| lad | 0.001 | 100 | 1 | 0.019636 | 0.08004 | 0.01965397 | 0.07853951 |
| lad | 0.001 | 150 | 0.4 | 0.018941 | 0.1126 | 0.01896881 | 0.11069265 |
| lad | 0.001 | 150 | 0.7 | 0.018905 | 0.11427 | 0.01893275 | 0.11238544 |
| lad | 0.001 | 150 | 1 | 0.018875 | 0.11566 | 0.01889948 | 0.11394069 |
| lad | 0.01 | 50 | 0.4 | 0.015172 | 0.28914 | 0.01524339 | 0.28548702 |
| lad | 0.01 | 50 | 0.7 | 0.015128 | 0.29122 | 0.01520293 | 0.28738824 |
| lad | 0.01 | 50 | 1 | 0.015136 | 0.29086 | 0.01521128 | 0.28699303 |
| lad | 0.01 | 100 | 0.4 | 0.012452 | 0.41658 | 0.01257285 | 0.41073908 |
| lad | 0.01 | 100 | 0.7 | 0.01242 | 0.41809 | 0.01254464 | 0.41206188 |
| lad | 0.01 | 100 | 1 | 0.012424 | 0.4179 | 0.01254719 | 0.41194412 |
| lad | 0.01 | 150 | 0.4 | 0.011112 | 0.4794 | 0.01127006 | 0.47181196 |
| lad | 0.01 | 150 | 0.7 | 0.011109 | 0.47955 | 0.01126896 | 0.47186523 |
| lad | 0.01 | 150 | 1 | 0.011135 | 0.47832 | 0.01129014 | 0.47087169 |
| lad | 0.1 | 50 | 0.4 | 0.008357 | 0.60848 | 0.00875135 | 0.58978492 |
| lad | 0.1 | 50 | 0.7 | 0.008346 | 0.60898 | 0.00877073 | 0.58888781 |
| lad | 0.1 | 50 | 1 | 0.008439 | 0.60459 | 0.00887029 | 0.58419519 |
| lad | 0.1 | 100 | 0.4 | 0.007347 | 0.65577 | 0.00796543 | 0.62660181 |
| lad | 0.1 | 100 | 0.7 | 0.007308 | 0.65762 | 0.00795398 | 0.62714401 |
| lad | 0.1 | 100 | 1 | 0.007413 | 0.65268 | 0.0080638 | 0.62196815 |
| lad | 0.1 | 150 | 0.4 | 0.006846 | 0.67923 | 0.00759936 | 0.64374791 |
| lad | 0.1 | 150 | 0.7 | 0.006782 | 0.68224 | 0.00757159 | 0.64502813 |
| lad | 0.1 | 150 | 1 | 0.006913 | 0.67612 | 0.00769772 | 0.63911173 |
| lad | 1 | 50 | 0.4 | 0.007083 | 0.66813 | 0.00875474 | 0.58944635 |
| lad | 1 | 50 | 0.7 | 0.006377 | 0.70125 | 0.00799294 | 0.62510748 |
| lad | 1 | 50 | 1 | 0.006209 | 0.70907 | 0.0077691 | 0.63565072 |
| lad | 1 | 100 | 0.4 | 0.006578 | 0.69184 | 0.00898542 | 0.57856686 |
| lad | 1 | 100 | 0.7 | 0.005715 | 0.73225 | 0.00789763 | 0.6295634 |
| lad | 1 | 100 | 1 | 0.005848 | 0.72603 | 0.0076859 | 0.63957346 |
| lad | 1 | 150 | 0.4 | 0.006286 | 0.70548 | 0.00930891 | 0.56338504 |
| lad | 1 | 150 | 0.7 | 0.005327 | 0.7504 | 0.00790194 | 0.62934319 |
| lad | 1 | 150 | 1 | 0.005784 | 0.72899 | 0.00767823 | 0.63993198 |
| ls | 0.001 | 50 | 0.4 | 0.020318 | 0.04805 | 0.02033789 | 0.04640739 |
| ls | 0.001 | 50 | 0.7 | 0.020318 | 0.04806 | 0.02033703 | 0.04644681 |
| ls | 0.001 | 50 | 1 | 0.020318 | 0.04806 | 0.02033676 | 0.04645872 |
| ls | 0.001 | 100 | 0.4 | 0.019384 | 0.09181 | 0.01941379 | 0.08976467 |
| ls | 0.001 | 100 | 0.7 | 0.019387 | 0.09168 | 0.01941612 | 0.08965446 |
| ls | 0.001 | 100 | 1 | 0.019389 | 0.0916 | 0.01941671 | 0.08962747 |
| ls | 0.001 | 150 | 0.4 | 0.018534 | 0.13164 | 0.01857374 | 0.12917739 |
| ls | 0.001 | 150 | 0.7 | 0.018541 | 0.13132 | 0.01858014 | 0.12887627 |
| ls | 0.001 | 150 | 1 | 0.018546 | 0.13109 | 0.0185837 | 0.12871021 |
| ls | 0.01 | 50 | 0.4 | 0.014298 | 0.33011 | 0.01440064 | 0.32493207 |
| ls | 0.01 | 50 | 0.7 | 0.01436 | 0.32721 | 0.01446224 | 0.32204879 |
| ls | 0.01 | 50 | 1 | 0.014398 | 0.32541 | 0.01450183 | 0.32019021 |
| ls | 0.01 | 100 | 0.4 | 0.011287 | 0.47117 | 0.01145881 | 0.46287047 |
| ls | 0.01 | 100 | 0.7 | 0.011379 | 0.46686 | 0.01155342 | 0.45843556 |
| ls | 0.01 | 100 | 1 | 0.011434 | 0.46429 | 0.01160831 | 0.45586629 |
| ls | 0.01 | 150 | 0.4 | 0.009863 | 0.5379 | 0.0100943 | 0.52681486 |
| ls | 0.01 | 150 | 0.7 | 0.009936 | 0.53449 | 0.0101654 | 0.52347827 |
| ls | 0.01 | 150 | 1 | 0.009963 | 0.53322 | 0.01019229 | 0.52222028 |
| ls | 0.1 | 50 | 0.4 | 0.007297 | 0.6581 | 0.00788237 | 0.63040321 |
| ls | 0.1 | 50 | 0.7 | 0.007312 | 0.6574 | 0.00790319 | 0.6294497 |
| ls | 0.1 | 50 | 1 | 0.007391 | 0.6537 | 0.00799513 | 0.62516614 |
| ls | 0.1 | 100 | 0.4 | 0.006244 | 0.70743 | 0.00718758 | 0.66295786 |
| ls | 0.1 | 100 | 0.7 | 0.006223 | 0.70842 | 0.00715727 | 0.6643936 |
| ls | 0.1 | 100 | 1 | 0.006342 | 0.70284 | 0.00725819 | 0.6596882 |
| ls | 0.1 | 150 | 0.4 | 0.005677 | 0.73404 | 0.00688989 | 0.67692493 |
| **ls** | **0.1** | **150** | **0.7** | **0.00561** | **0.7372** | **0.0068239** | **0.6800149** |
| ls | 0.1 | 150 | 1 | 0.005773 | 0.72954 | 0.00692636 | 0.67523646 |
| ls | 1 | 50 | 0.4 | 0.006734 | 0.68452 | 0.01025992 | 0.51858685 |
| ls | 1 | 50 | 0.7 | 0.005093 | 0.76139 | 0.00824433 | 0.61324829 |
| ls | 1 | 50 | 1 | 0.004557 | 0.78652 | 0.00759144 | 0.6438416 |
| ls | 1 | 100 | 0.4 | 0.006332 | 0.70335 | 0.0126147 | 0.40799026 |
| ls | 1 | 100 | 0.7 | 0.00414 | 0.80604 | 0.00874179 | 0.5898925 |
| ls | 1 | 100 | 1 | 0.00341 | 0.84022 | 0.00783083 | 0.63261132 |
| ls | 1 | 150 | 0.4 | 0.006096 | 0.71438 | 0.01465261 | 0.31228093 |
| ls | 1 | 150 | 0.7 | 0.003555 | 0.83344 | 0.00913514 | 0.57142583 |
| ls | 1 | 150 | 1 | 0.002746 | 0.87132 | 0.00805164 | 0.62227024 |
| quantile | 0.001 | 50 | 0.4 | - | - | - | - |
| quantile | 0.001 | 50 | 0.7 | - | - | - | - |
| quantile | 0.001 | 50 | 1 | - | - | - | - |
| quantile | 0.001 | 100 | 0.4 | - | - | - | - |
| quantile | 0.001 | 100 | 0.7 | - | - | - | - |
| quantile | 0.001 | 100 | 1 | - | - | - | - |
| quantile | 0.001 | 150 | 0.4 | - | - | - | - |
| quantile | 0.001 | 150 | 0.7 | - | - | - | - |
| quantile | 0.001 | 150 | 1 | - | - | - | - |
| quantile | 0.01 | 50 | 0.4 | - | - | - | - |
| quantile | 0.01 | 50 | 0.7 | - | - | - | - |
| quantile | 0.01 | 50 | 1 | - | - | - | - |
| quantile | 0.01 | 100 | 0.4 | - | - | - | - |
| quantile | 0.01 | 100 | 0.7 | - | - | - | - |
| quantile | 0.01 | 100 | 1 | - | - | - | - |
| quantile | 0.01 | 150 | 0.4 | - | - | - | - |
| quantile | 0.01 | 150 | 0.7 | - | - | - | - |
| quantile | 0.01 | 150 | 1 | - | - | - | - |
| quantile | 0.1 | 50 | 0.4 | - | - | - | - |
| quantile | 0.1 | 50 | 0.7 | - | - | - | - |
| quantile | 0.1 | 50 | 1 | - | - | - | - |
| quantile | 0.1 | 100 | 0.4 | 0.020602 | 0.03476 | 0.02125846 | 0.00207996 |
| quantile | 0.1 | 100 | 0.7 | 0.020605 | 0.0346 | 0.02125191 | 0.00273213 |
| quantile | 0.1 | 100 | 1 | - | - | - | - |
| quantile | 0.1 | 150 | 0.4 | 0.019553 | 0.08391 | 0.02037671 | 0.04318099 |
| quantile | 0.1 | 150 | 0.7 | 0.019452 | 0.08864 | 0.02022392 | 0.05043498 |
| quantile | 0.1 | 150 | 1 | 0.020149 | 0.05598 | 0.02084444 | 0.02116641 |
| quantile | 1 | 50 | 0.4 | - | - | - | - |
| quantile | 1 | 50 | 0.7 | 0.018951 | 0.1121 | 0.02068589 | 0.02891698 |
| quantile | 1 | 50 | 1 | 0.018871 | 0.11588 | 0.02034092 | 0.04478427 |
| quantile | 1 | 100 | 0.4 | - | - | - | - |
| quantile | 1 | 100 | 0.7 | 0.016921 | 0.20723 | 0.01908237 | 0.10402237 |
| quantile | 1 | 100 | 1 | 0.017678 | 0.17179 | 0.01930237 | 0.09365094 |
| quantile | 1 | 150 | 0.4 | - | - | - | - |
| quantile | 1 | 150 | 0.7 | 0.015956 | 0.25245 | 0.01839743 | 0.13626776 |
| quantile | 1 | 150 | 1 | 0.017345 | 0.18737 | 0.0190588 | 0.10516447 |

| XG Boost | | | | | | |
| --- | --- | --- | --- | --- | --- | --- |
| hyper parameter | | | Regression result | | | |
| learning_rate | sub sample | max_depth | MSE (training) | R^2^  (training) | MSE (validation) | R^2^ (validation) |
| 0.001 | 0.4 | 4 | - | - | - | - |
| 0.001 | 0.4 | 6 | - | - | - | - |
| 0.001 | 0.4 | 8 | - | - | - | - |
| 0.001 | 0.7 | 4 | - | - | - | - |
| 0.001 | 0.7 | 6 | - | - | - | - |
| 0.001 | 0.7 | 8 | - | - | - | - |
| 0.001 | 1 | 4 | - | - | - | - |
| 0.001 | 1 | 6 | - | - | - | - |
| 0.001 | 1 | 8 | - | - | - | - |
| 0.01 | 0.4 | 4 | 0.0123785 | 0.4200336 | 0.01266665 | 0.4058983 |
| 0.01 | 0.4 | 6 | 0.0109497 | 0.486974 | 0.01161918 | 0.45503696 |
| 0.01 | 0.4 | 8 | 0.0098876 | 0.5367387 | 0.01103696 | 0.48234632 |
| 0.01 | 0.7 | 4 | 0.0124275 | 0.4177403 | 0.01272275 | 0.40325874 |
| 0.01 | 0.7 | 6 | 0.010832 | 0.4924901 | 0.01154058 | 0.45872316 |
| 0.01 | 0.7 | 8 | 0.009509 | 0.554477 | 0.01084393 | 0.49140563 |
| 0.01 | 1 | 4 | 0.0124997 | 0.4143564 | 0.01279766 | 0.3997457 |
| 0.01 | 1 | 6 | 0.010857 | 0.4913187 | 0.01158015 | 0.45686129 |
| 0.01 | 1 | 8 | 0.0094264 | 0.5583482 | 0.01087714 | 0.48984301 |
| 0.1 | 0.4 | 4 | 0.0052293 | 0.7549927 | 0.00666016 | 0.68770131 |
| 0.1 | 0.4 | 6 | 0.0033776 | 0.8417492 | 0.00620469 | 0.70906581 |
| 0.1 | 0.4 | 8 | 0.0019893 | 0.9067973 | 0.00615445 | 0.71141746 |
| 0.1 | 0.7 | 4 | 0.0051143 | 0.7603842 | 0.00659799 | 0.69059999 |
| 0.1 | 0.7 | 6 | 0.0029814 | 0.8603162 | 0.00608509 | 0.71470159 |
| **0.1** | **0.7** | **8** | **0.001361** | **0.936243** | **0.0060843** | **0.714739** |
| 0.1 | 1 | 4 | 0.0052546 | 0.7538135 | 0.00668287 | 0.68664406 |
| 0.1 | 1 | 6 | 0.0031711 | 0.8514286 | 0.00618291 | 0.71012014 |
| 0.1 | 1 | 8 | 0.0014574 | 0.9317188 | 0.00616133 | 0.71110775 |
| 1 | 0.4 | 4 | 0.0049303 | 0.7689982 | 0.01225737 | 0.42477362 |
| 1 | 0.4 | 6 | 0.0034596 | 0.8379185 | 0.01891677 | 0.11238337 |
| 1 | 0.4 | 8 | - | - | - | - |
| 1 | 0.7 | 4 | 0.0026393 | 0.8763497 | 0.00897535 | 0.57908594 |
| 1 | 0.7 | 6 | 0.0005686 | 0.9733618 | 0.01072566 | 0.49672996 |
| 1 | 0.7 | 8 | 2.07E-05 | 0.9990293 | 0.01127219 | 0.47106949 |
| 1 | 1 | 4 | 0.0018882 | 0.911536 | 0.00798238 | 0.62555297 |
| 1 | 1 | 6 | 0.0001941 | 0.990904 | 0.00896972 | 0.57936541 |
| 1 | 1 | 8 | 1.66E-06 | 0.9999222 | 0.00912632 | 0.57184644 |

| SVR | | | | | | | | |
| --- | --- | --- | --- | --- | --- | --- | --- | --- |
| hyper parameter | | | | | Regression result | | | |
| kernel | C | degree | nu | length | MSE | R^2^ | MSE | R^2^ |
|  |  |  |  | _scale | (training) | (training) | (validation) | (validation) |
| matern | 0.001 | x | 0.5 | 1 | 0.013123 | 0.3851508 | 0.01319443 | 0.38149163 |
| matern | 0.001 | x | 0.5 | 10 | 0.016054 | 0.2478473 | 0.01607551 | 0.24643005 |
| matern | 0.001 | x | 0.5 | 100 | 0.020333 | 0.0473483 | 0.02034421 | 0.04618098 |
| matern | 0.001 | x | 1.5 | 1 | 0.012608 | 0.4092745 | 0.01266069 | 0.40648855 |
| matern | 0.001 | x | 1.5 | 10 | 0.018533 | 0.1316787 | 0.01854417 | 0.13063233 |
| matern | 0.001 | x | 1.5 | 100 | - | - | - | - |
| matern | 0.001 | x | 2.5 | 1 | 0.012535 | 0.4126994 | 0.01258301 | 0.41012753 |
| matern | 0.001 | x | 2.5 | 10 | 0.019301 | 0.0957218 | 0.01931243 | 0.09460006 |
| matern | 0.001 | x | 2.5 | 100 | - | - | - | - |
| matern | 0.01 | x | 0.5 | 1 | 0.009151 | 0.5712416 | 0.00945416 | 0.5567699 |
| matern | 0.01 | x | 0.5 | 10 | 0.01149 | 0.4616809 | 0.01157342 | 0.45744123 |
| matern | 0.01 | x | 0.5 | 100 | 0.015792 | 0.2601281 | 0.01581307 | 0.25873032 |
| matern | 0.01 | x | 1.5 | 1 | 0.009396 | 0.559801 | 0.00958003 | 0.55084974 |
| matern | 0.01 | x | 1.5 | 10 | 0.013417 | 0.3713918 | 0.01344079 | 0.36992776 |
| matern | 0.01 | x | 1.5 | 100 | 0.021018 | 0.0152573 | 0.0210281 | 0.01409665 |
| matern | 0.01 | x | 2.5 | 1 | 0.009535 | 0.5532763 | 0.00969054 | 0.54566299 |
| matern | 0.01 | x | 2.5 | 10 | 0.014165 | 0.3363288 | 0.01418525 | 0.33504093 |
| matern | 0.01 | x | 2.5 | 100 | 0.021231 | 0.0053053 | 0.02124041 | 0.00413422 |
| matern | 0.1 | x | 0.5 | 1 | 0.006541 | 0.6935632 | 0.00754704 | 0.64607469 |
| matern | 0.1 | x | 0.5 | 10 | 0.00868 | 0.5933385 | 0.00899179 | 0.578413 |
| matern | 0.1 | x | 0.5 | 100 | 0.01137 | 0.4672813 | 0.01145448 | 0.46301361 |
| matern | 0.1 | x | 1.5 | 1 | 0.007423 | 0.6522252 | 0.00792561 | 0.62834172 |
| matern | 0.1 | x | 1.5 | 10 | 0.01089 | 0.4897866 | 0.01095313 | 0.48646651 |
| matern | 0.1 | x | 1.5 | 100 | 0.018075 | 0.1531713 | 0.01808483 | 0.15218055 |
| matern | 0.1 | x | 2.5 | 1 | 0.007748 | 0.6369784 | 0.00815103 | 0.61777333 |
| matern | 0.1 | x | 2.5 | 10 | 0.011299 | 0.4706377 | 0.01134867 | 0.46792375 |
| matern | 0.1 | x | 2.5 | 100 | 0.01922 | 0.0995348 | 0.01923072 | 0.09843092 |
| **matern** | **1** | **x** | **0.5** | **1** | **0.00493** | **0.769096** | **0.0071564** | **0.66437** |
| matern | 1 | x | 0.5 | 10 | 0.006435 | 0.6984845 | 0.00745335 | 0.65045859 |
| matern | 1 | x | 0.5 | 100 | 0.008649 | 0.5947905 | 0.00896164 | 0.5798256 |
| matern | 1 | x | 1.5 | 1 | 0.006138 | 0.7124114 | 0.00724355 | 0.6602711 |
| matern | 1 | x | 1.5 | 10 | 0.009497 | 0.555025 | 0.00963378 | 0.54828598 |
| matern | 1 | x | 1.5 | 100 | 0.013099 | 0.3862813 | 0.01312341 | 0.3848005 |
| matern | 1 | x | 2.5 | 1 | 0.006574 | 0.6919804 | 0.00743748 | 0.65117848 |
| matern | 1 | x | 2.5 | 10 | 0.010271 | 0.518807 | 0.01036147 | 0.51417253 |
| matern | 1 | x | 2.5 | 100 | 0.014101 | 0.3393262 | 0.01412029 | 0.3380802 |
| linear | 0.001 | x | x | x | 0.011705 | 0.4515779 | 0.01174612 | 0.44930774 |
| linear | 0.01 | x | x | x | 0.010728 | 0.4973828 | 0.01079806 | 0.49370908 |
| linear | 0.1 | x | x | x | 0.010601 | 0.503303 | 0.01070281 | 0.49814311 |
| linear | 1 | x | x | x | 0.010615 | 0.502675 | 0.01071489 | 0.49756139 |
| poly | 0.001 | 2 | x | x | 0.01057 | 0.5047807 | 0.01063406 | 0.50144708 |
| poly | 0.001 | 3 | x | x | 0.009553 | 0.5524296 | 0.00967228 | 0.54648899 |
| poly | 0.001 | 4 | x | x | 0.008774 | 0.588906 | 0.00896433 | 0.57965762 |
| poly | 0.01 | 2 | x | x | 0.009318 | 0.5634184 | 0.00945184 | 0.55678486 |
| poly | 0.01 | 3 | x | x | 0.008459 | 0.6036685 | 0.00870478 | 0.5917778 |
| poly | 0.01 | 4 | x | x | 0.007832 | 0.6330654 | 0.00823906 | 0.61359717 |
| poly | 0.1 | 2 | x | x | 0.008526 | 0.6005304 | 0.00877408 | 0.58849841 |
| poly | 0.1 | 3 | x | x | 0.007703 | 0.6391103 | 0.00815055 | 0.61774699 |
| poly | 0.1 | 4 | x | x | 0.007137 | 0.6655955 | 0.00786321 | 0.63119828 |
| poly | 1 | 2 | x | x | 0.008146 | 0.6183207 | 0.00852675 | 0.60006925 |
| poly | 1 | 3 | x | x | 0.0071 | 0.6673412 | 0.00785497 | 0.63157862 |
| poly | 1 | 4 | x | x | 0.006567 | 0.6923368 | 0.00772916 | 0.63745742 |
| rbf | 0.001 | x | x | x | 0.012412 | 0.4184705 | 0.01245725 | 0.4160163 |
| rbf | 0.01 | x | x | x | 0.009664 | 0.5472369 | 0.00979542 | 0.54073755 |
| rbf | 0.1 | x | x | x | 0.007703 | 0.6391103 | 0.00815055 | 0.61774699 |
| rbf | 1 | x | x | x | 0.00709 | 0.6678177 | 0.00773002 | 0.63746549 |
| sigmoid | 0.001 | x | x | x | 0.016409 | 0.2312201 | 0.01641812 | 0.23025646 |
| sigmoid | 0.01 | x | x | x | - | - | - | - |
| sigmoid | 0.1 | x | x | x | - | - | - | - |
| sigmoid | 1 | x | x | x | - | - | - | - |

| KNN | | | | | | |
| --- | --- | --- | --- | --- | --- | --- |
| hyper parameter | | | Regression result | | | |
| weights | n_neighbors | p | MSE | R^2^ | MSE | R^2^ |
|  |  |  | (training) | (training) | (validation) | (validation) |
| distance | 3 | 1 | 0 | 1 | 0.006949182 | 0.674062543 |
| distance | 3 | 2 | 0 | 1 | 0.007150935 | 0.664583143 |
| distance | 5 | 1 | 0 | 1 | 0.006477858 | 0.696188619 |
| distance | 5 | 2 | 0 | 1 | 0.006674789 | 0.686930548 |
| distance | 7 | 1 | 0 | 1 | 0.006315903 | 0.703790885 |
| distance | 7 | 2 | 0 | 1 | 0.006515809 | 0.694398698 |
| distance | 11 | 1 | 0 | 1 | 0.006246539 | 0.707032496 |
| distance | 11 | 2 | 0 | 1 | 0.006408039 | 0.699455665 |
| **distance** | **13** | **1** | **0** | **1** | **0.006240216** | **0.707334234** |
| distance | 13 | 2 | 0 | 1 | 0.00641358 | 0.699210096 |
| uniform | 3 | 1 | 0.0033045 | 0.845177201 | 0.006784389 | 0.681748944 |
| uniform | 3 | 2 | 0.0033899 | 0.841173428 | 0.007004894 | 0.671395121 |
| uniform | 5 | 1 | 0.004164 | 0.804904971 | 0.006443894 | 0.697763619 |
| uniform | 5 | 2 | 0.0043158 | 0.797796254 | 0.006715678 | 0.684949094 |
| uniform | 7 | 1 | 0.0047257 | 0.778592803 | 0.006488609 | 0.695664943 |
| uniform | 7 | 2 | 0.0049523 | 0.767978214 | 0.006798189 | 0.681101186 |
| uniform | 11 | 1 | 0.0055401 | 0.740437638 | 0.006797877 | 0.681130473 |
| uniform | 11 | 2 | 0.0057469 | 0.730747943 | 0.007041538 | 0.669705173 |
| uniform | 13 | 1 | 0.0058426 | 0.726261739 | 0.006937152 | 0.674625505 |
| uniform | 13 | 2 | 0.0060543 | 0.716343611 | 0.007190231 | 0.662755298 |

| PLS | | | | |
| --- | --- | --- | --- | --- |
| hyper parameter | Regression result | | | |
| n_components | MSE (training) | R^2^ (training) | MSE (validation) | R^2^ (validation) |
| 1 | 0.01362685 | 0.361558217 | 0.013647799 | 0.360114499 |
| **2** | **0.011709902** | **0.45137136** | **0.011741524** | **0.449379353** |

| GPR | | | | | | | |
| --- | --- | --- | --- | --- | --- | --- | --- |
| hyper parameter | | | | Regression result | | | |
| kernel | length_scale | nu | alpha | MSE | R^2^ | MSE | R^2^ |
|  |  |  |  | (training) | (training) | (validation) | (validation) |
| matern | 1 | 0.5 | 1.E-01 | 0.009115 | 0.5729631 | 0.00935315 | 0.5615118 |
| matern | 1 | 1.5 | 1.E-01 | 0.009394 | 0.5598853 | 0.0095382 | 0.5528082 |
| matern | 1 | 2.5 | 1.E-01 | 0.009489 | 0.5554288 | 0.00962471 | 0.5487502 |
| matern | 10 | 0.5 | 1.E-01 | 0.009115 | 0.5729625 | 0.00935316 | 0.5615113 |
| matern | 10 | 1.5 | 1.E-01 | 0.009394 | 0.5598851 | 0.0095382 | 0.5528082 |
| matern | 10 | 2.5 | 1.E-01 | 0.009489 | 0.5554291 | 0.0096247 | 0.5487506 |
| matern | 100 | 0.5 | 1.E-01 | 0.009115 | 0.5729636 | 0.00935314 | 0.5615123 |
| matern | 100 | 1.5 | 1.E-01 | 0.009394 | 0.5598856 | 0.00953819 | 0.5528086 |
| matern | 100 | 2.5 | 1.E-01 | 0.009489 | 0.555429 | 0.0096247 | 0.5487505 |
| matern | 1 | 0.5 | 1.00E-04 | 5.06E-06 | 0.9997628 | 0.00724202 | 0.6603939 |
| matern | 1 | 1.5 | 1.00E-04 | 1.71E-05 | 0.9991982 | 0.02098273 | 0.0158985 |
| matern | 1 | 2.5 | 1.00E-04 | - | - | - | - |
| **matern** | **10** | **0.5** | **1.00E-04** | **5.05E-06** | **0.999763** | **0.007241** | **0.660421** |
| matern | 10 | 1.5 | 1.00E-04 | - | - | - | - |
| matern | 10 | 2.5 | 1.00E-04 | - | - | - | - |
| matern | 100 | 0.5 | 1.00E-04 | 5.10E-06 | 0.9997609 | 0.00724589 | 0.6602121 |
| matern | 100 | 1.5 | 1.00E-04 | - | - | - | - |
| matern | 100 | 2.5 | 1.00E-04 | - | - | - | - |
| matern | 1 | 0.5 | 1.00E-07 | 5.25E-12 | 1 | 0.00729847 | 0.6577491 |
| matern | 1 | 1.5 | 1.00E-07 | - | - | - | - |
| matern | 1 | 2.5 | 1.00E-07 | - | - | - | - |
| matern | 10 | 0.5 | 1.00E-07 | 5.25E-12 | 1 | 0.00729836 | 0.6577537 |
| matern | 10 | 1.5 | 1.00E-07 | - | - | - | - |
| matern | 10 | 2.5 | 1.00E-07 | - | - | - | - |
| matern | 100 | 0.5 | 1.00E-07 | 5.31E-12 | 1 | 0.00730184 | 0.6575886 |
| matern | 100 | 1.5 | 1.00E-07 | - | - | - | - |
| matern | 100 | 2.5 | 1.00E-07 | - | - | - | - |
| matern | 1 | 0.5 | 1.00E-10 | 5.25E-18 | 1 | 0.00729851 | 0.6577474 |
| matern | 1 | 1.5 | 1.00E-10 | - | - | - | - |
| matern | 1 | 2.5 | 1.00E-10 | - | - | - | - |
| matern | 10 | 0.5 | 1.00E-10 | 5.25E-18 | 1 | 0.00729909 | 0.6577216 |
| matern | 10 | 1.5 | 1.00E-10 | - | - | - | - |
| matern | 10 | 2.5 | 1.00E-10 | - | - | - | - |
| matern | 100 | 0.5 | 1.00E-10 | 5.32E-18 | 1 | 0.00729997 | 0.6576814 |
| matern | 100 | 1.5 | 1.00E-10 | - | - | - | - |
| matern | 100 | 2.5 | 1.00E-10 | - | - | - | - |
